# Comparative Diagnostic Efficacy of Swept-Source OCT and Scheimpflug Imaging in Clinically Unaffected Eyes of Very Asymmetric Ectasia

**DOI:** 10.1016/j.xops.2026.101285

**Published:** 2026-06-15

**Authors:** Robert Herber, Tadas Naujokaitis, Renato Ambrósio, Lisa Ramm, Janine Lenk, Frederik Raiskup, Ramin Khoramnia

**Affiliations:** 1 Department of Ophthalmology, Faculty of Medicine and University Hospital Carl Gustav Carus, TU Dresden, Dresden, Germany; 2 Department of Ophthalmology, Federal University of the State of Rio de Janeiro, Rio de Janeiro, Brazil

**Keywords:** Corneal tomography, Ectasia screening, Keratoconus, OCT, Scheimpflug imaging

## Abstract

**Purpose:**

To determine the diagnostic ability of corneal tomography indices for detecting corneal ectasia using a swept-source OCT system (SS-OCT; ANTERION), a rotating Scheimpflug camera system (Pentacam HR), and a dynamic ultra-high-speed Scheimpflug noncontact tonometry and cornea analyzer (Corvis ST).

**Design:**

A prospective cross-sectional study.

**Subjects:**

Participants were assigned to a healthy group (N = 228), a bilateral keratoconus group (N = 308), with 1 eye randomly selected, or a very asymmetric ectasia group with no clinical ectasia signs (VAE-NES, n = 91), with the unoperated fellow eye having clinical ectasia (VAE-E, n = 75).

**Methods:**

All participants underwent examination with all devices. The following indices were analyzed: Screening Corneal Objective Risk of Ectasia (SCORE), tomographical and biomechanical index (TBI, version 2), Pentacam random forest index (PRFI), Belin/Ambrósio total deviation value (BAD-D, version 3), and Corvis biomechanical index.

**Main Outcome Measures:**

Comparison of the area under the curve (AUC) from receiver operating characteristic (ROC) curve analyses.

**Results:**

When differentiating VAE-NES from healthy eyes, ROC analysis showed a significantly higher AUC for SCORE (0.888) compared with PRFI (0.765; *P* < 0.001) and BAD-D (0.686; *P* < 0.001), but not TBI (0.847; *P* = 0.06). Sensitivity and specificity were 75% and 85% for SCORE, 58%/86% for PRFI, 41%/90% for BAD-D, and 77%/81% for TBI, respectively. In distinguishing healthy eyes from all ectatic eyes, AUC values were 0.978 for SCORE, 0.955 for PRFI, and 0.936 for BAD-D (all *P* < 0.001). Tomographical and biomechanical index (AUC 0.968) and SCORE were comparable (*P* = 0.06).

**Conclusions:**

Screening Corneal Objective Risk of Ectasia derived from SS-OCT and TBI from integrated Scheimpflug imaging demonstrated the highest and comparable diagnostic performance for detecting clinically unaffected fellow eyes without clinical ectasia signs but with ectasia susceptibility (VAE-NES).

**Financial Disclosures:**

Proprietary or commercial disclosure may be found in the Footnotes and Disclosures at the end of this article.

Keratoconus (KC) is a progressive corneal disorder that may lead to significant vision loss if not identified early. Early detection is critical, ideally before the onset of clinical symptoms and before consideration of laser vision correction procedures. Iatrogenic ectasia can develop regardless of the type of laser refractive surgery performed, particularly in cases involving corneas with suspicious topographic features or subclinical KC.^1^

Therefore, corneal topography alone is insufficient for ectasia risk assessment in refractive candidates.^2^ To enhance diagnostic accuracy, assessing the posterior corneal curvature and corneal thickness has become essential. Rotating Scheimpflug camera (RSC) has proven reliable for this purpose over the past 2 decades. However, it has a significant limitation: blue light scattering in scarred corneas can be substantial, rendering the technique unsuitable for certain cases, such as advanced KC. Consequently, imaging modalities employing longer wavelengths and technologies such as OCT have emerged as viable alternatives.

Previous studies demonstrated that swept-source OCT (SS-OCT) provides superior repeatability in measuring corneal curvature parameters compared to RSC, particularly in moderate to advanced KC.^3^ This suggests that OCT is a more appropriate approach for diagnosing and monitoring KC in these cases. Another study revealed a comparable diagnostic accuracy of SS-OCT to RSC, although it lacked information regarding the detection of very early KC.^4^

A further critical factor in early ectasia screening is corneal biomechanics, as it is hypothesized that the cornea undergoes alterations in its microstructure and biomechanical properties, which ultimately manifest in changes in topography or tomography.^5^^,^^6^ Recently, Brillouin spectroscopy and air-puff tonometry using Scheimpflug imaging have demonstrated biomechanical alterations in very early ectasia.^7^, ^8^, ^9^ Furthermore, integrating artificial intelligence with biomechanical and tomographic parameters improves the prediction of susceptible corneas that may develop ectasia.^10^

Recent technological advances have led to the development of new indices for screening for corneal ectasia. This study aimed to compare the diagnostic performance of corneal tomography parameters obtained with a novel optical SS-OCT system and combined biomechanical and tomographic indices from Scheimpflug imaging for detecting ectasia susceptibility.

## Methods

This prospective, cross-sectional study was conducted at the Department of Ophthalmology, Carl Gustav Carus Faculty of Medicine, and the University Hospital, TU Dresden, Germany, between December 2021 and April 2025. The study protocol adhered to the principles outlined in the Declaration of Helsinki, was approved by the Ethics Committee of the TU Dresden, and registered as a clinical study (NCT04251143). Exclusion criteria for all study participants encompassed preexisting corneal or ocular pathologies (excluding KC), ocular surgery or trauma in the past, diabetes mellitus, and pregnancy. Participants were required to be ≥18 years old. The requirement for individual informed patient consent was waived by the Ethics Committee for this study. Notably, only 1 eye of each healthy and KC subject was randomly selected for the study, except for very asymmetric ectasia (VAE) cases.

Participants, who were either refractive surgery candidates or KC patients, received a routine ophthalmic examination including slit lamp biomicroscopy of both the anterior and posterior eye segments, RSC, air-puff tonometry, and SS-OCT. Additionally, participants were asked to discontinue wearing contact lenses ≥10 days prior to their consultation. This protocol was applied to all lens types, including soft and rigid gas permeable lenses, and to all participant subgroups, such as those with KC and VAE. While a longer washout period is recommended for rigid gas permeable lenses to minimize residual corneal warpage, a shorter period was adopted in this study to balance the need for accurate diagnostic imaging with the preservation of patient visual function, consistent with our clinical workflow.

### Corneal Classification for Study Groups

Corneas of participants, classified using Scheimpflug tomography, were defined as healthy when anterior corneal surface maps were within conventional clinical limits, without evidence of inferior steepening (e.g., inferior–superior keratometry difference >1.45 D) or a distorted, asymmetrical bowtie pattern (e.g., skewed radial axis >22°). Corneas were also required to have sufficient thickness for refractive laser surgery, defined as a residual stromal bed >250 μm and a percentage of tissue altered <40%. Abnormal elevation on the posterior surface was assessed qualitatively, and only corneas without central posterior elevation were included. For these participants, corneal tomography was performed to assess the eyes for clinical suitability for laser refractive surgery.

Patients with KC were stratified into 2 subgroups: bilateral KC and VAE. Bilateral KC cases exhibited ≥2 characteristic tomographic features, such as inferior corneal steepening, a skewed asymmetric bowtie pattern, abnormal corneal thickness distribution, or posterior corneal elevation, in both eyes. Secondary clinical signs, including Fleischer rings or Vogt’s striae, were identified during slit-lamp examination.

The VAE subgroup comprised individuals with clinically evident KC in 1 eye (very asymmetric ectasia with ectasia [VAE-E]), excluding those who had undergone prior interventions, such as corneal cross-linking. The contralateral eye, designated as VAE group with no clinical ectasia signs (VAE-NES), exhibited no clinical or topographic signs of ectasia, defined by the following criteria: best-corrected visual acuity of 20/20, and absence of inferior steepening, inferior–superior asymmetry, or a skewed bow-tie pattern on corneal topography. Importantly, the VAE-NES classification was based exclusively on the anterior corneal surface (topography) and was defined by topographic parameters (e.g., simulated keratometry values) remaining within conventional clinical limits. The representative device displays of patient measurements are shown in Supplement 1 (available at www.ophthalmologyscience.org).

### Swept-Source OCT-Based Tomography

The technical properties of the anterior segment SS-OCT device (ANTERION, Heidelberg Engineering GmbH) have been described elsewhere.^4^^,^^11^^,^^12^ Parameters describing the anterior and posterior corneal curvature, as well as corneal pachymetry, were used in this study. Additionally, the following parameters were analyzed: the difference between the maximum keratometry value and the opposite keratometry value of the anterior corneal surface (Kmax – opposite K), difference between the mean inferior curvature and the mean superior value of the corneal curvature (inferior–superior K mean), irregularity of the axial curvature in the central 3-mm ring (anterior irregularity [3 mm]), and posterior elevation (best-fit sphere as the reference surface) at the thinnest point of the cornea (posterior elevation of thinnest point).^4^ The Screening Corneal Objective Risk of Ectasia (SCORE), a machine learning–derived discriminant parameter, ranges from –4 to 20 on a color scale. Values below 0 indicate low risk of ectasia, whereas values above 0 indicate higher risk. Screening Corneal Objective Risk of Ectasia integrates 5 parameters: vertical position of maximum posterior keratometry (PostKmax Y), inferior–superior K mean, Kmax – opposite K, percentage thickness increase at 2 mm from the thinnest pachymetry (PTI2), and the ratio of posterior to anterior corneal radius (Pr/Ar).^12^

Before measurements were taken, patients were asked to blink and fixate the target light. Only measurements classified by the device as ‘pass’ or ‘borderline’ were included.

### Corneal Biomechanical and Tomography Using Scheimpflug Imaging

In this study, an RSC (Pentacam HR, Oculus Optikgeraete GmbH) and a dynamic ultra-high-speed Scheimpflug noncontact tonometry and cornea analyzer (CST, Corvis ST, Oculus Optikgeraete GmbH) were used to assess corneal tomography and biomechanics. Both devices can be connected to calculate not only individual parameters but also the tomographical and biomechanical index (TBI, version 2).^10^ Various parameters and indices were calculated; the following were analyzed in this study: the Ambrosio relational thickness (ART max),^13^ the Belin/Ambrósio total deviation value (BAD-D, version 3),^13^^,^^14^ the inferior–superior asymmetry value (IS-value), the KC percentage index score (KISA %),^15^ the averaged pachymetric progression (RPI Avg), and the Pentacam random forest index (PRFI).^16^ Among biomechanical parameters, the Corvis biomechanical index (CBI) was used.^17^

### Statistical Analysis

Statistical analyses were performed using SPSS version 27 (IBM Corp), MedCalc (MedCalc Software Ltd), and R (R Foundation for Statistical Computing). Data distribution was assessed for normality using the Kolmogorov–Smirnov test and Q–Q plots. As data were non-normally distributed, results are presented as medians with interquartile ranges.

Comparisons among multiple groups were conducted using the Kruskal–Wallis test, with post hoc adjustment for multiple comparisons (Dunn’s test). Receiver operating characteristic (ROC) curve analysis was performed to evaluate the diagnostic performance of each parameter in distinguishing normal eyes from ectatic eyes and to determine sensitivity and specificity. Areas under the ROC curves were statistically compared using the DeLong test.

Sensitivity and specificity were calculated using literature-based (lb) cutoff values. In addition, optimized (optm) cutoff values were determined using the Youden index to maximize the combined sensitivity and specificity. Diagnostic odds ratios (DORs) were calculated as the ratio of true positives to false negatives divided by the ratio of false positives to true negatives, according to the following formula^18^:

(1) $$DOR=\frac{TP}{FN}/\;\frac{FP}{TN}$$

Based on the calculated DORs, the numbers of true positives, false negatives, false positives, and true negatives were derived and compared among SCORE, TBI, and BAD-D.

Sample size calculation was based on an anticipated area under the curve (AUC) of 0.80 for differentiating healthy eyes from ectatic eyes using the SCORE parameter. This threshold was considered clinically meaningful, as a comparable tomographic parameter, the BAD-D, has previously demonstrated an AUC of 0.887 for differentiating healthy eyes from subclinical KC.^19^ With an α level of 0.05 and a power of 80%, a minimum sample size of 150 eyes was required. A *P* value <0.05 was considered statistically significant. Owing to multiple comparisons, only *P* values comparing normal eyes with each ectatic subgroup are reported.

## Results

### Demographics

This study included 1 eye randomly selected from 228 normal healthy participants, and from 308 bilateral KC patients, along with 91 eyes with VAE-NES of patients presenting with ectasia in the fellow eye, along with the 75 unoperated ectatic eyes (VAE-E). Sixteen of the VAE-E group were excluded due to previous corneal cross-linking. The demographic data are presented in Table 1.

**Table 1:** Demographic Data of the Study Groups

	Healthy	Bilateral KC	VAE-NES	VAE-E
No. of eyes	228	308	91	75
Age (years)	29 (23–37)	35 (26.5 – 41)	32 (25 – 38)	32 (25.3 – 37)
Gender (male, %)	50	75	71	68
Eyes (right, %)	50	48	57	43
BCVA (logMAR)	0.0 (0.0–0.05)	0.2 (0.1–0.3)	0.0 (0.0–0.0)	0.2 (0.1–0.4)

BCVA = best-corrected visual acuity; E = ectasia; KC = keratoconus; No = number; NES = nonectatic signs; VAE = very asymmetric ectasia; logMAR = logarithm of the minimum angle of resolution.

Metric data are presented as median and interquartile range.

### Corneal Tomography Parameters of SS-OCT and RSC

All analyzed parameters from both devices are listed in Tables 2 and 3 as well as in Supplements 2 and 3 (available at www.ophthalmologyscience.org). As expected, these parameters differed statistically significantly between normal eyes and KC eyes (bilateral KC and VAE-E; all *P* < 0.001). Similarly, eyes from patients with bilateral KC and VAE-E did not differ statistically significantly (all *P* > 0.05, data not shown).

**Table 2:** Comparison of Tomographic Parameters Obtained by SS-OCT among Healthy Eyes, Bilateral KC, and VAE-NES and VAE-E

	Healthy	Bilateral KC	*P* Value∗	VAE-NES	*P* Value∗	VAE-E	*P* Value∗
Kmax – opposite K (D)	0.52 (0.21–0.92)	7.97 (4.23–12.2)	**<0.001**	0.73 (0.36–1.19)	1.0	7.5 (3.95–11.91)	**<0.001**
Inferior – superior K mean (D)	–0.04 (–0.37 to 0.27)	5.5 (3.37–8)	**<0.001**	0.44 (0.11–0.68)	**0.003**	5.52 (3.77–7.79)	**<0.001**
Anterior irregularity [3 mm] (D)	0.75 (0.5–1.2)	3.3 (2.4–4.6)	**<0.001**	0.7 (0.5–0.9)	1.0	3.34 (2.7–4.9)	**<0.001**
Posterior elevation of thinnest point (μm)	5.5 (3–8)	56.0 (38.0–79.5)	**<0.001**	7.0 (4–11)	0.794	56.0 (41.3–80.8)	**<0.001**
PostKmax Y (mm)	1.41 (1.08–1.65)	–1.2 (–1.93 to [–0.68])	**<0.001**	–0.93 (–1.94 to 0.51)	**<0.001**	–0.96 (–1.79 to [–0.6])	**<0.001**
PTI2	1.2 (1.1–1.3)	3.7 (2.8–4.9)	**<0.001**	1.3 (1.1–1.5)	0.943	3.8 (2.9–4.8)	**<0.001**
Pr/Ar	0.84 (0.83–0.85)	0.81 (0.78–0.84)	**<0.001**	0.85 (0.84–0.86)	**0.025**	0.81 (0.78–0.83)	**<0.001**
SCORE	–1.600 (–2.1 to [–1])	16.4 (10.3–23)	**<0.001**	0.4 (–0.7 to 1.1)	**<0.001**	15.4 (11.1–22.9)	**<0.001**

E = ectasia; KC = keratoconus; NES = nonectatic signs; PostKmax Y = vertical position of maximum posterior keratometry; Pr/Ar = the ratio of posterior to anterior corneal radius; PTI2 = percentage thickness increase at 2 mm from the thinnest pachymetry; SCORE = Screening Corneal Objective Risk of Ectasia; SS-OCT = swept-source OCT; VAE = very asymmetric ectasia.

Data are presented as median and interquartile range.

∗ Compared to healthy using Kruskal–Wallis test. Significances are marked in bold.

**Table 3:** Comparison of Tomographic Parameters Obtained by the Rotating Scheimpflug Camera System and Scheimpflug-Based Air-Puff Tonometry among Healthy Eyes, Bilateral KC, and VAE-NES and VAE-E

	Healthy	Bilateral KC	*P* Value∗	VAE-NES	*P* Value∗	VAE-E	*P* Value∗
I-S value (D)	–0.04 (–0.5 to 0.4)	6.05 (3.7–8.5)	**<0.001**	0.43 (0.03–0.82)	**0.016**	5.41 (3.94–7.51)	**<0.001**
KISA(%)	3.8 (1.9–6.8)	408 (149 – 1158)	**<0.001**	6.8 (2.7–13.8)	0.334	331 (172 – 1482)	**<0.001**
RPI Avg.	0.99 (0.92–1.06)	1.82 (1.54–2.29)	**<0.001**	1.04 (0.97–1.14)	0.262	1.80 (1.59–2.13)	**<0.001**
ART Max. (μm)	453 (402 – 495)	175 (133 – 211)	**<0.001**	386 (337 – 424)	**0.003**	176 (147 – 207)	**<0.001**
BAD D	1.03 (0.59–1.43)	7.62 (5.15–10.26)	**<0.001**	1.35 (0.98–1.94)	0.090	6.97 (5.29–9.36)	**<0.001**
PRFI	0.05 (0.02–0.13)	0.99 (0.96–1)	**<0.001**	0.22 (0.07–0.44)	**0.004**	0.99 (0.96–1)	**<0.001**
TBI	0.08 (0.02–0.24)	1.0 (1–1)	**<0.001**	0.63 (0.31–0.86)	**<0.001**	1 (1–1)	**<0.001**
CBI	0.17 (0.07–0.34)	0.99 (0.91–1)	**<0.001**	0.55 (0.23–0.74)	**<0.001**	0.98 (0.94–1)	**<0.001**

ART max = Ambrosio relational thickness; BAD D = Belin/Ambrosio total deviation value; CBI = Corvis biomechanical index; E = ectasia; I-S = inferior–superior asymmetry value; KC = keratoconus; KISA% = keratoconus percentage index score; NES = nonectatic signs; PRFI = Pentacam random forest index; RPI Avg = averaged pachymetric progression; TBI = tomographical and biomechanical index (version 2); VAE = very asymmetric ectasia.

Data are presented as median and interquartile range.

∗ Compared to healthy using Kruskal–Wallis test. Significances are marked in bold.

**Figure 1:**
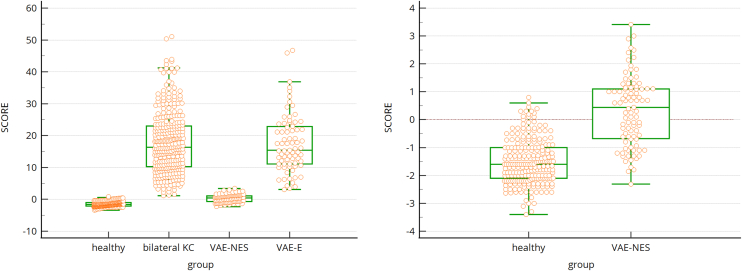
Comparison of the SCORE index between healthy eyes, bilateral keratoconus, and VAE-NES and VAE-E (left). Detailed comparison between healthy eyes and VAE-NES (right). Orange dots represent the individual data points. SCORE = Screening Corneal Objective Risk of Ectasia; VAE-E = very asymmetric ectasia with ectasia; VAE-NES = very asymmetric ectasia with nonectatic signs.

Four parameters obtained from the SS-OCT, which were inferior–superior K mean, PostKmax Y, Pr/Ar, and SCORE, showed statistically significantly altered values in the VAE-NES group compared with normal eyes. Interestingly, Pr/Ar values were elevated in VAE-NES eyes, even though early KC typically presents with corneal steepening and therefore lower Pr/Ar values. The most highly statistically significant single parameter was PostKmax Y, with median (interquartile range) values of 1.41 (1.08–1.65) and –0.93 (–1.94 to 0.51) for normal and VAE-NES eyes (*P* < 0.001), respectively. Additionally, SCORE was statistically significantly higher in VAE-NES eyes than in normal eyes (Fig 1, *P* < 0.001).

Similarly, 5 parameters differed statistically significantly between the healthy and VAE-NES groups from RSC. The I-S value was slightly higher in VAE-NES eyes compared with healthy eyes (*P* = 0.016), although the maximum values in both groups were comparable (1.33 and 1.35 D, respectively) and not clinically relevant (see Supplement 3). Ambrosio relational thickness was lower in VAE-NES eyes than in normal eyes (*P* = 0.003, Table 3); notably, this parameter is analogous to PTI2 in SS-OCT, for which no difference was observed (*P* = 0.94, Table 2). Additionally, PRFI, TBI, and CBI were all statistically significantly elevated in VAE-NES compared with normal eyes.

### Diagnostic Ability of the Parameters

Table 4 presents the results of the ROC curve analysis, including the respective AUC, Youden index, literature-based (lb) and optimized (optm) cutoff values, sensitivity, specificity, and positive/negative likelihood ratios. As expected, all parameters achieved an AUC greater than 0.9 when distinguishing normal eyes from all ectatic cases, indicating that they are sufficient to detect predominantly clinical ectasia. Using a lb-cutoff of >0, the SCORE parameter showed a sensitivity and specificity of 92% and 94.7%, respectively. An optm-cutoff of >0.6 was determined for detecting all ectasia, yielding sensitivity and specificity values of 90.1% and 99.6%, respectively. The lb- and optm-cutoffs of TBI were very similar (>0.65 vs. >0.622), resulting in sensitivities and specificities of approximately 90% and 97%, respectively. These values can be considered clinically equivalent between SCORE and TBI. Although the specificity of PRFI, BAD-D, and CBI exceeded 90%, their sensitivity values were between 80% and 90%. Consequently, the AUC values for SCORE and TBI were statistically significantly higher compared to those for PRFI, BAD-D, and CBI, with no significant difference between the 2 (Table 5, Fig 3).

**Table 4:** Receiver Operating Characteristic (ROC) Analysis of Machine Learning and Artificial Intelligence Parameters Derived from SS-OCT and Scheimpflug Imaging Systems

Parameter	Group	AUC (95% CI)	YI	Cutoff	Sn (%)	Sp (%)	+LR	-LR
SCORE	NE vs. all ectasia	0.978 (0.965–0.988)	-	>0^12^	91.98	94.74	17.48	0.085
	NE vs. all ectasia		0.8965	>0.6	90.08	99.56	205.39	0.1
	NE vs. VAE-NES	0.888 (0.848–0.920)	-	>0^12^	58.24	94.74	11.07	0.44
	NE vs. VAE-NES		0.5981	>-0.6147	74.73	85.09	5.01	0.30
TBI	NE vs. all ectasia	0.968 (0.953–0.980)	-	>0.65^10^	89.66	97.32	33.47	0.11
	NE vs. all ectasia		0.8717	>0.622	90.30	96.87	28.89	0.1
	NE vs. VAE-NES	0.847 (0.802–0.885)	-	>0.43^10^	61.54	87.95	5.11	0.44
	NE vs. VAE-NES		0.5817	>0.295	76.92	81.25	4.1	0.28
PRFI	NE vs. all ectasia	0.955 (0.937–0.969)	-	>0.265^16^	88.61	94.64	16.54	0.12
	NE vs. all ectasia		0.8466	>0.334	87.34	97.32	32.61	0.13
	NE vs. VAE-NES	0.765 (0.715–0.811)	-	>0.125^16^	65.93	75	2.64	0.45
	NE vs. VAE-NES		0.4440	>0.18	58.24	86.16	4.21	0.48
BAD-D	NE vs. all ectasia	0.936 (0.915–0.953)	-	>2.60^20^	80.59	100	-	0.19
	NE vs. all ectasia		0.8286	>2.01	84.18	98.68	63.97	0.16
	NE vs. VAE-NES	0.686 (0.632–0.736)	-	>1.6^21^	41.76	87.28	3.28	0.67
	NE vs. VAE-NES		0.3101	>1.64	40.66	90.35	4.21	0.66
CBI	NE vs. all ectasia	0.935 (0.914–0.952)	-	>0.5^17^	86.68	89.24	8.05	0.15
	NE vs. all ectasia		0.7979	>0.537	85.62	94.17	14.69	0.15
	NE vs. VAE-NES	0.778 (0.728–0.823)	-	>0.2^9^	81.32	57.85	1.93	0.32
	NE vs. VAE-NES		0.4544	>0.403	59.34	86.10	4.27	0.47

AUC = area under curve; BAD-D = Belin/Ambrosio total deviation value; CBI = Corvis biomechanical index; CI = confidence interval; +LR = positive likelihood ratio; -LR = negative likelihood ratio; NE = normal eyes; NES = nonectatic signs; PRFI = Pentacam random forest index; SCORE = Screening Corneal Objective Risk of Ectasia; Sn = sensitivity; Sp = specificity; SS-OCT = swept-source OCT; TBI = tomographical and biomechanical index (version 2); VAE = very asymmetric ectasia; YI = Youden index.

**Table 5:** Comparison of Area under the Curve (AUC) Values between the Parameters Using DeLong Test

Parameter	Group	TBI	PRFI	BAD-D	CBI
SCORE	NE vs. all ectasia (AUC = 0.978)	0.06	**<0.001**	**<0.001**	**<0.001**
	NE vs. VAE-NES (AUC = 0.888)	0.06	**<0.001**	**<0.001**	**<0.001**
TBI	NE vs. all ectasia (AUC = 0.968)	-	**<0.001**	**<0.001**	**<0.001**
	NE vs. VAE-NES (AUC = 0.847)	–	**<0.001**	**<0.001**	**0.016**
PRFI	NE vs. all ectasia (AUC = 0.955)	–	–	**<0.001**	**0.027**
	NE vs. VAE-NES (AUC = 0.765)	–	–	**<0.001**	0.776
BAD-D	NE vs. all ectasia (AUC = 0.936)	–	–	–	0.931
	NE vs. VAE-NES (AUC = 0.686)	–	–	–	**0.005**
CBI	NE vs. all ectasia (AUC = 0.935)	–	–	–	–
	NE vs. VAE-NES (AUC = 0.778)	–	–	–	–

BAD-D = Belin/Ambrosio total deviation value; CBI = Corvis biomechanical index; NE = normal eyes; NES = nonectatic signs; PRFI = Pentacam random forest index; SCORE = Screening Corneal Objective Risk of Ectasia; TBI = tomographical and biomechanical index (version 2); VAE = very asymmetric ectasia.

**Figure 2:**
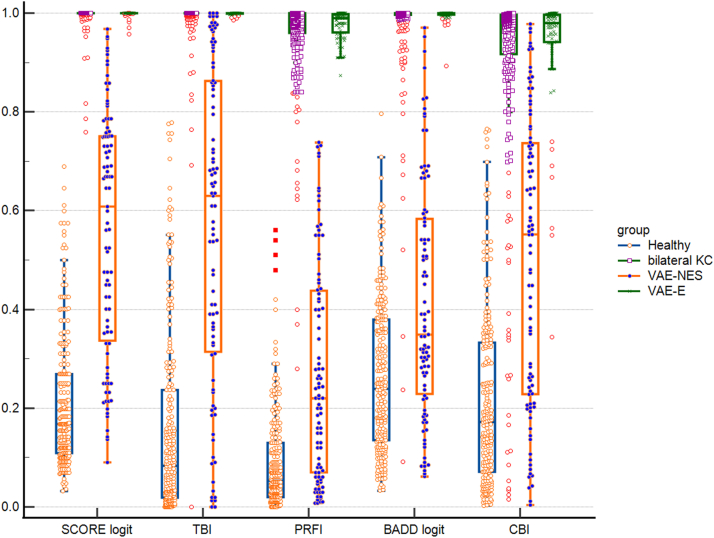
All parameters are shown as boxplots on a scale between 0 and 1 for each group. Screening Corneal Objective Risk of Ectasia and BAD-D were converted using the standard logistic (sigmoid) function to place them on a scale between 0 and 1. BAD-D = Belin/Ambrósio total deviation value; SCORE = Screening Corneal Objective Risk of Ectasia; VAE-E = very asymmetric ectasia with ectasia; VAE-NES = very asymmetric ectasia with nonectatic signs.

**Figure 3:**
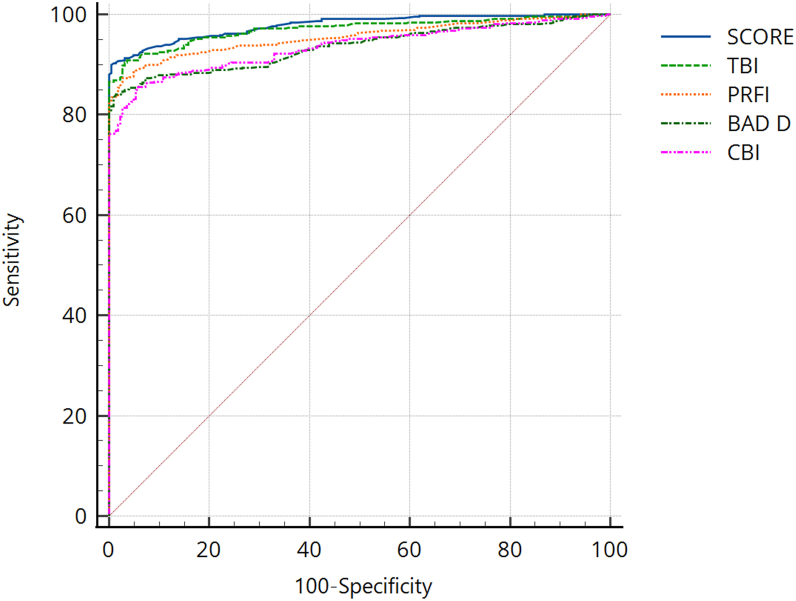
Receiver operating characteristic curves for SCORE, TBI, PRFI, BAD-D, and CBI distinguishing healthy from all ectasia eyes.BAD-D = Belin/Ambrósio total deviation value; CBI = Corvis biomechanical index; PRFI = Pentacam random forest index; SCORE = Screening Corneal Objective Risk of Ectasia; TBI = tomographical and biomechanical index (version 2).

**Figure 4:**
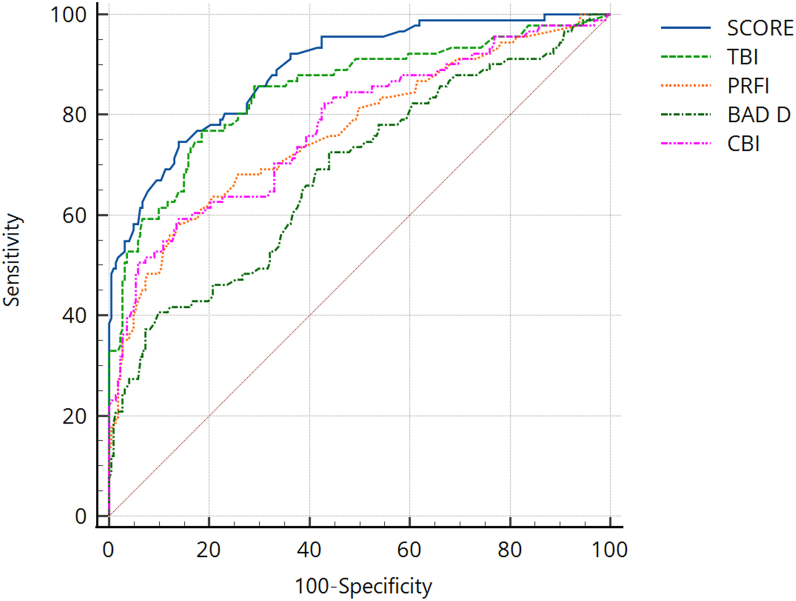
Receiver operating characteristic curves for SCORE, TBI, PRFI, BAD-D, and CBI distinguishing healthy from VAE-NES eyes. BAD-D = Belin/Ambrósio total deviation value; CBI = Corvis biomechanical index; SCORE = Screening Corneal Objective Risk of Ectasia; PRFI = Pentacam random forest index; TBI = tomographical and biomechanical index (version 2); VAE-NES = very asymmetric ectasia with nonectatic signs.

When distinguishing between normal eyes and VAE-NES, the AUC, as well as sensitivity and specificity, decreased for all parameters. For SCORE, the AUC was 0.888, and using the lb-cutoff of >0 yielded a sensitivity of 58% and a specificity of 95%. Using the optm-cutoff of >–0.6147 increased sensitivity to 74.7%, though it reduced specificity to 85.1%. Similarly, the lb- and optm-cutoffs for TBI were different (>0.43 vs. >0.295), and the optm-cutoff increased sensitivity from 61.5% to 77% at the expense of decreasing specificity from 88% to 81.2%. SCORE showed slightly lower sensitivity and slightly higher specificity than TBI, with no statistically significant difference in the AUC (*P* = 0.06; see Table 5). Both SCORE and TBI markedly outperformed PRFI (0.765), BAD-D (0.686), and CBI (0.778, all *P* < 0.001). The PRFI and CBI had similar AUCs (*P* = 0.776) and were superior to BAD-D (*P* < 0.05, Fig 4).

The ROC analysis for the additional parameters is shown in Supplement 4 (available at www.ophthalmologyscience.org). Among these parameters, ART Max, RPI Avg, KISA%, I–S value (D), PTI2, PostKmax Y, and the posterior elevation at the thinnest point achieved AUC values above 0.9 for detecting all ectatic eyes. However, PostKmax Y was the only parameter to achieve a sufficient AUC value of 0.819, with sensitivities and specificities of 78% and 90%, respectively, for detecting VAE-NES (cutoff ≤0.67).

### Diagnostic Odds Ratios and Quantitative Assessment of Improved Case Classification

**Figure 5:**
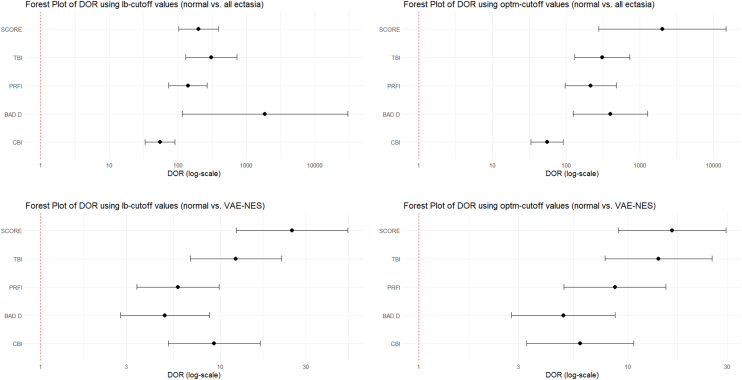
Forest plot of DOR for literature-based (lb) and optimized (optm) cutoff values for distinguishing healthy eyes from ectasia. BAD-D = Belin/Ambrósio total deviation value; CBI = Corvis biomechanical index; DOR = diagnostic odds ratio; PRFI = Pentacam random forest index; SCORE = Screening Corneal Objective Risk of Ectasia; TBI = tomographical and biomechanical index (version 2); VAE-NES = very asymmetric ectasia with nonectatic signs.

The diagnostic odds ratio (DOR) was calculated using lb- and optm-cutoffs for detecting all ectasia and VAE-NES and presented in Figure 5 as a forest plot with log-scale. The predicted outcome (yes or no ectasia) was compared with the actual condition (healthy or ectasia). The SCORE achieved the highest DOR separating normal eyes from all ectasia (except for lb-cutoff values) and VAE-NES (Fig 5). In particular, SCORE and TBI demonstrated a significantly higher DOR using optm-cutoffs compared to BAD-D considering their 95% confidence intervals, when normal eyes were differentiated from VAE-NES.

For further analysis, the SCORE and BAD-D values were transformed using the standard logistic (sigmoid) function to place them on a scale between 0 and 1 (Fig 2). Because the BAD-D is a commonly used clinical parameter, it was used as the reference standard and compared with the most powerful parameters identified in this study (SCORE and TBI). Considering all ectasias, SCORE identified 6 additional KC cases and 1 additional healthy case per 100 patients compared to BAD-D. However, when focusing on VAE-NES, SCORE classified 25 more patients as having ectasia and 17 more patients as healthy compared to BAD-D. TBI classified 6 additional eyes as ectatic and 4 healthy eyes as ectatic, per 100 patients, compared with normal eyes. With VAE-NES, an additional 13 ectatic and 19 healthy eyes per 100 patients were correctly identified compared with BAD-D. The detection of ectatic eyes was similar between SCORE and TBI. However, SCORE classified 4 more eyes as normal than TBI did. Additionally, SCORE identified 3 and 2 additional ectatic and healthy eyes, respectively, per 100 patients when comparing normal eyes with all ectasia and VAE-NES. It should be noted that this analysis is highly dependent on the study sample composition and the specific cutoff values applied for classification.

## Discussion

Early detection of ectasia is clinically essential for 2 critical reasons. First, it prevents iatrogenic ectasia following laser vision correction procedures, which, despite being widely used, is a rare complication. Second, early identification allows for management and treatment before ectasia progresses to clinical KC, which is associated with vision loss.^22^ New corneal imaging technologies generate extensive parameter sets that, when analyzed with artificial intelligence algorithms, provide clinicians with access to expert-level diagnostic knowledge. This study investigated 2 novel ectasia probability indices and compared their diagnostic performance to that of commonly used indices. The primary findings were: (1) SCORE, a machine learning–derived parameter from SS-OCT, demonstrated comparable diagnostic ability to an artificial intelligence–based parameter that integrates both tomographic and biomechanical measurements, and (2) both SCORE and TBI (version 2) substantially outperformed the standard clinical parameter, BAD-D (version 3), as well as PRFI and CBI.

The strength of this study lies in its analysis of new diagnostic techniques, for which identical inclusion and exclusion criteria were applied to all subjects. However, comparisons with earlier studies are limited because inconsistent definitions and terms, such as "subclinical keratoconus" and "forme fruste keratoconus,” have been used.^23^ Nevertheless, this stage of the disease is typically used to evaluate the performance of established and new indices for detecting early ectatic changes. Ideally, the analysis would include cases in which there is manifest clinical KC in 1 eye and no obvious abnormalities in the other (VAE-NES). This would allow subtle structural changes to be identified and analyzed. These analyses focus on such cases because it is assumed that the probability of early KC in the apparently normal fellow eye is high, as KC is generally considered a bilateral disease.^24^ The challenge lies in defining the typical appearance of a VAE eye as normal. However, relying solely on subjective inclusion criteria in studies may not be the most effective approach to achieve comparability. Henriquez et al reviewed the definitions of subclinical and forme fruste KC and found that, in over 70% of studies, the definition was established when the contralateral eye was diagnosed with KC. This was followed by the detection of nonkeratoconic clinical findings via biomicroscopy (>50%) and normal topography (approximately 60%).^25^ However, since the definition of a normal cornea can vary widely, objective criteria would be more appropriate. Recently, studies have used the KC percentage index (KISA%) as an inclusion criterion. A KISA% value exceeding 100 indicates a clinical KC, while a value between 60 and 100 suggests a suspect KC. Conversely, a KISA% below 60 indicates a normal cornea. However, the cutoff value of 60 has been a subject of controversy regarding its diagnostic accuracy. Hammoud et al found that some KC patients had values lower than 60 and showed progression.^26^ Therefore, the parameter appears to be a less adequate inclusion criterion for objectively separating normal from KC. The authors conclude that the parameter should not be used alone or automatically calculated without subjectively assessing topographies.^26^

In the present study, the VAE-NES group was within conventional clinical limits for topographic and tomographic indices obtained from Scheimpflug imaging, although statistically significant differences were observed compared to the healthy group. Specifically, the median (interquartile range) I-S values were –0.04 (–0.50 to 0.40) and 0.43 (0.03 to 0.82), respectively, suggesting that, despite a statistically significant difference between the groups, the clinical characteristics were similar. A 75th-percentile value of 0.82 D indicates that clinically relevant inferior corneal steepening was present in most cases, with maximum values not exceeding 1.5 D.

Similarly, KISA% values were comparable between the 2 groups, with a 75th-percentile value of 13.8 at its maximum, and no values exceeding 60 in either group. Overall, therefore, the healthy and VAE-NES groups can be considered clinically comparable with respect to anterior surface properties.

Although BAD-D (version 3) has become more clinically relevant in recent years, helping to prevent many suspect eyes from undergoing laser refractive surgery and aiding in the detection of early KC, the present findings suggest otherwise. Specifically, the discriminatory power of the diagnosis between normal corneas and VAE-NES appears limited when stricter inclusion criteria are applied, and topographic characteristics are incorporated into the assessment. These findings stand in contrast to previous reports in which this parameter demonstrated superior performance, varying by study design.^19^^,^^27^, ^28^, ^29^, ^30^ Nevertheless, our results are consistent with those of other studies, indicating reduced diagnostic accuracy. For example, Steinberg et al reported an AUC of 0.712 (with 66% sensitivity and 66% specificity), while Koh et al noted an AUC of 0.67 (with 61% sensitivity and 86% specificity).^31^^,^^32^ One probable explanation for this discrepancy is that the parameter was initially developed without incorporating early KC cases. Conversely, subsequent refinements to BAD-D (version 4) incorporated such data, resulting in improved performance.^33^ However, as BAD-D (version 4) was unavailable at the time of this study, further research is needed to confirm the effectiveness of this improvement.

Moreover, our study revealed that the PRFI, a parameter based solely on tomography and artificial intelligence algorithms, was less effective at distinguishing healthy eyes from VAE-NES than SCORE and TBI.^16^ The highest discriminative power was observed for TBI, a tomographic and biomechanical index derived from Scheimpflug imaging, compared with the other parameters. The TBI was recently optimized to version 2.0, which was used in the present study. During the initial validation, TBI (version 2) demonstrated an AUC value of 0.945, with a sensitivity of 84.4% and a specificity of 90.1% at a cutoff value of 0.43, distinguishing normal eyes from VAE with normal topography. This condition is comparable to current VAE-NES definition. However, the diagnostic performance observed in the current cohort was lower. With the lb-cutoff, sensitivity was 62%, and specificity was 88%. Using the optm-cutoff increased sensitivity to 77% while slightly decreasing specificity to 81%.

Swept-source OCT is a relatively novel device that has rarely been investigated for the detection of ectasia. In a previous study, our group examined the diagnostic ability of new parameters in a cohort of patients with KC. One of the key findings was that SCORE, mean inferior–superior keratometry, and posterior elevation at the thinnest point exhibited the highest discriminatory power in distinguishing healthy eyes from eyes with KC.^4^ In the current study, these values remained comparable (both AUC > 0.9) when considering all ectasia cases. However, when comparing only VAE-NES and healthy eyes, the respective AUC values decreased to 0.767 and 0.612.^4^ Only the SCORE parameter maintained a high level of discriminatory power, though it decreased as well when considering only VAE-NES cases. Another important finding of our previous study was that, despite statistical differences between healthy eyes and several stages of KC, epithelium thickness parameters did not demonstrate high discriminatory power, as the best parameter, the standard deviation of epithelial thickness, yielded a sensitivity and specificity of 80% and 91%, respectively, with an AUC of 0.92.^4^ The clinical utility of standard epithelial thickness parameters for the detection of early ectasia therefore remains to be seen. For this reason, it may not have been included in the SCORE parameters developed by Saad et al, although it is believed that it could offer advantages. On the other hand, analyzing the epithelium in sectors and combining epithelial parameters with tomographical and biomechanical data could be a better approach for detecting very early ectasia.^34^

The SCORE was the most valuable parameter for estimating ectasia risk probability using the SS-OCT device. In contrast, none of the parameters derived from the radar map were sensitive or specific enough to detect VAE-NES cases. This result is consistent with expectations, as these parameters primarily characterize the morphology of the anterior corneal surface, which remains unaffected in the early stages of ectasia. Additionally, maximum keratometry and thinnest point thickness were excluded from the evaluation because they are clinically insensitive. Other variables from the SCORE calculation were analyzed separately. Interestingly, the Y-position of the posterior Kmax reached high statistical significance. This likely indicates that subtle changes initially occur on the posterior corneal surface, consistent with the theory of ectasia development. This observation is consistent with the results of Saad et al, though other studies have been unable to confirm this finding.^12^^,^^21^^,^^35^ Except for the inferior–superior K-mean, the Pr/Ar ratio, and the SCORE itself, none of the examined parameters showed statistically significant differences between healthy control subjects and VAE-NES eyes. This contrasts with previous reports.^12^^,^^21^ These discrepancies likely reflect differences in cohort composition and disease stage distribution. In particular, the VAE-NES cases analyzed in the pilot study by Saad et al showed significant differences in curvature and pachymetry on both the anterior and posterior corneal surfaces.^12^ The absence of such differences in the present cohort, combined with SCORE's performance, suggests that this index can identify eyes with apparently normal corneas that are at high risk of developing KC.

In ROC analysis, the SCORE index achieved an AUC of 0.888 for distinguishing healthy eyes from VAE-NES. The index had a sensitivity of 75% and a specificity of 85% (optm cutoff value: >–0.6147). However, these results were inferior to those of the pilot study, which reported an AUC value of 0.95, a sensitivity of 75%, and a specificity of 99% (cutoff: 0).^12^ In contrast, Kim et al reported a sensitivity of 82% and a specificity of 78% at a cutoff value of 0.25.^21^ Furthermore, Naujokaitis et al evaluated SS-OCT parameters in Scheimpflug tomography-normal eyes of KC patients (BAD-D <1.6) and identified an optimized cutoff value of –1.1. This value yielded a sensitivity and specificity of 72%. This relatively low cutoff value attributable to the inclusion criteria; however, these criteria do not permit conclusions about the clinical situation. Nevertheless, this preselection allows an evaluation of how many eyes could be identified as ectatic using SS-OCT despite normal Scheimpflug imaging.^36^ The PostKmaxPosY showed a comparable AUC value and sensitivity in the current study but lower specificity. The inferior–superior K mean was another parameter of the pilot study that showed sufficient separating ability between cohorts in the pilot study; however, this could not be confirmed in the current study.

Overall, the SCORE index demonstrated the greatest ability to discriminate between healthy eyes and ectasia, outperforming purely tomographic Scheimpflug parameters, such as PRFI and BAD-D. The SCORE achieved comparable performance to TBI, which combines biomechanical and tomographic data; neither method was clinically superior. The DOR analysis confirmed this, as it is a prevalence-independent measure of test performance that indicates the magnitude by which a positive result is more likely in diseased individuals than in healthy individuals. Higher DOR values reflect better cohort separation. In this study, both SCORE and TBI achieved the highest DOR values of approximately 15 for detecting VAE-NES eyes, indicating that diseased individuals were 15 times more likely to test positive than healthy individuals. This surpassed the results of other parameters and was higher than reported by Naujokaitis et al.^36^

The limitations of this study are its monocentric design and the predominance of a Caucasian population. Since this population likely overlaps with that of the pilot study, the findings may not apply to other populations. Additionally, as previously noted, defining VAE-NES is a diverse group with complex and inconsistent in the absence of objective criteria. Therefore, other clinicians might grade the cases differently. At the time of the study, the optimized BAD-D version 4 index was not available and therefore could not be integrated into the analysis.^33^ However, this parameter will be evaluated in future studies. Moreover, all subjects were evaluated based on RSC, which might introduce an inherent circularity bias that may affect the study findings in relation to RSC indices. On the other hand, this circularity strengthens the outcome of SS-OCT indices, which were independent of group classification. A further limitation is the use of a 10-day minimum contact lens washout period, which is shorter than the 3–4 weeks recommended for rigid gas permeable lenses to minimize residual corneal warpage; consequently, residual warpage may have influenced topographic and tomographic indices in a subset of participants. Future studies should therefore incorporate longer washout periods or employ serial topographic and tomographic assessments where feasible.

In conclusion, SCORE derived from SS-OCT and TBI, which combines Scheimpflug-based tomographic data with air-puff tonometry, demonstrated the highest diagnostic performance for detecting clinically unaffected fellow eyes without clinical ectasia signs but with ectasia susceptibility compared with PRFI, BAD-D, CBI, and individual device parameters. Both can therefore be considered clinically comparable. Future work should test the integration of epithelial thickness data with tomography and biomechanical data to assess ectasia risk or susceptibility.
