# Supplementary material for: Comparative Diagnostic Efficacy of Swept-Source OCT and Scheimpflug Imaging in Clinically Unaffected Eyes of Very Asymmetric Ectasia
Source: Ophthalmol Sci. 2026 Jun 15;6(8):101285. doi: 10.1016/j.xops.2026.101285 (PMC13383214; doi:10.1016/j.xops.2026.101285)
Supplement: Supplement 1 [file mmc1.pdf]

Supplement 1 Representative device displays of patient measurements (VAE-NES group) for the right eye (left side) and the left eye (right side).

CASE #1

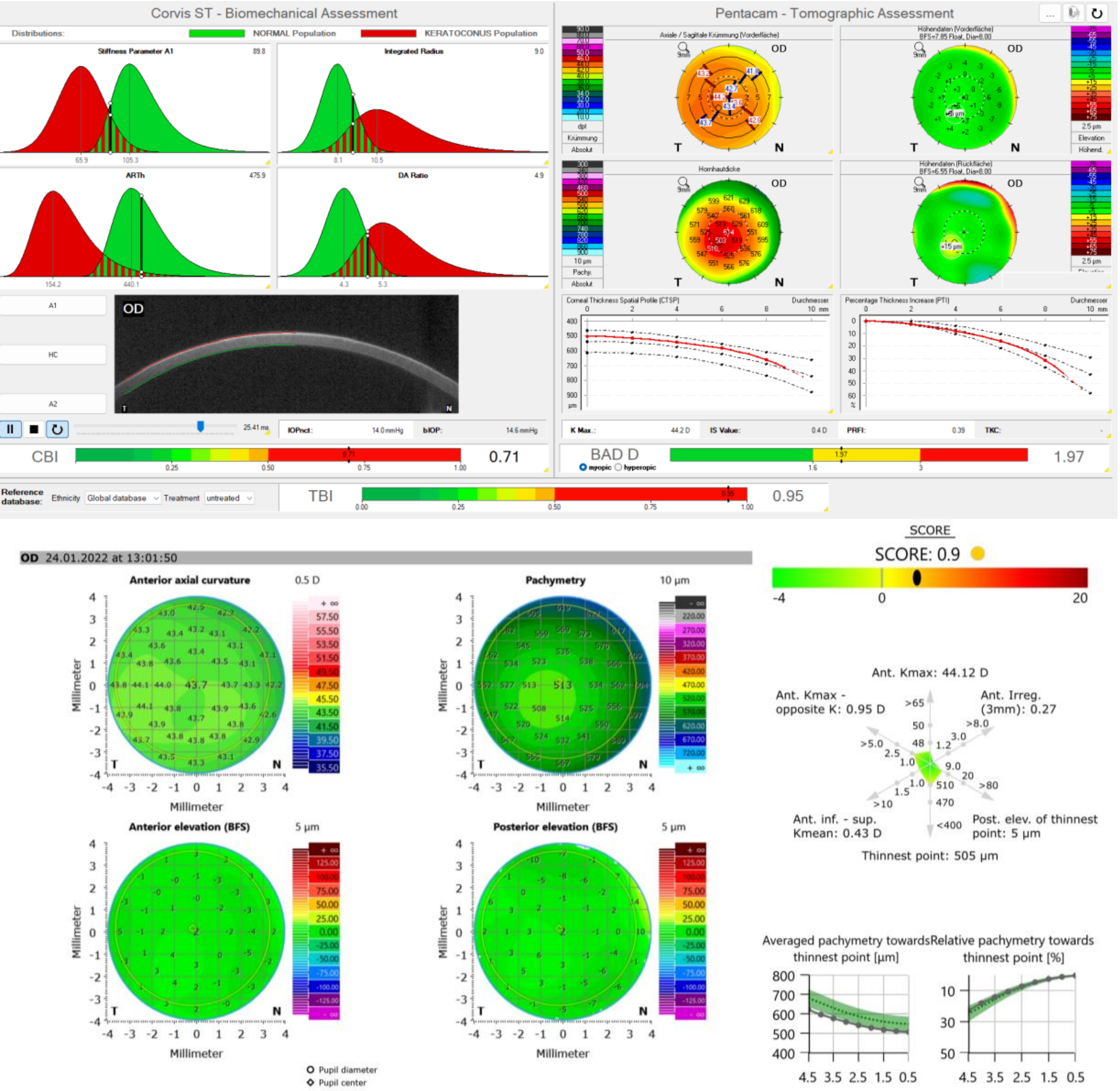

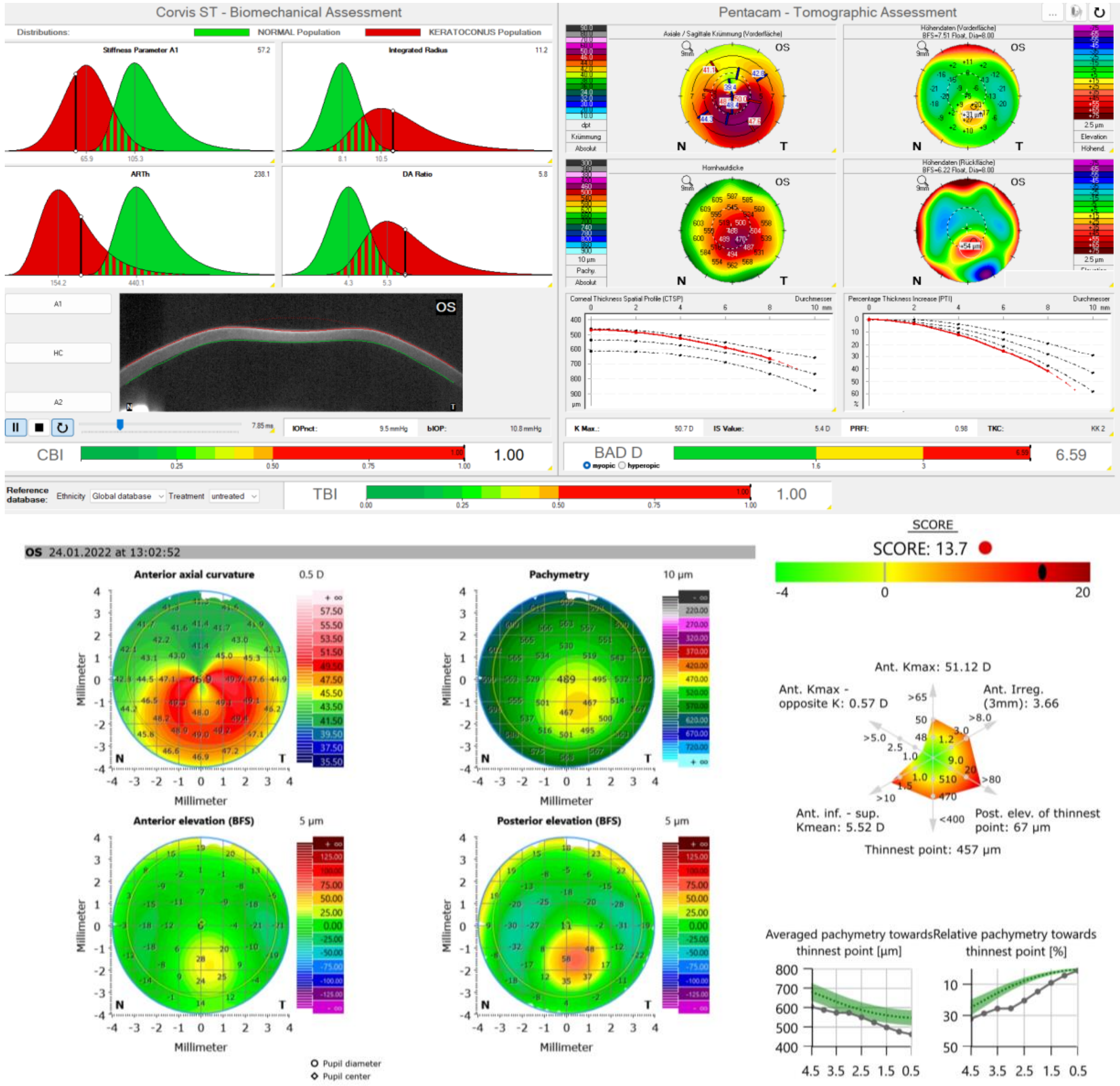

CASE #2

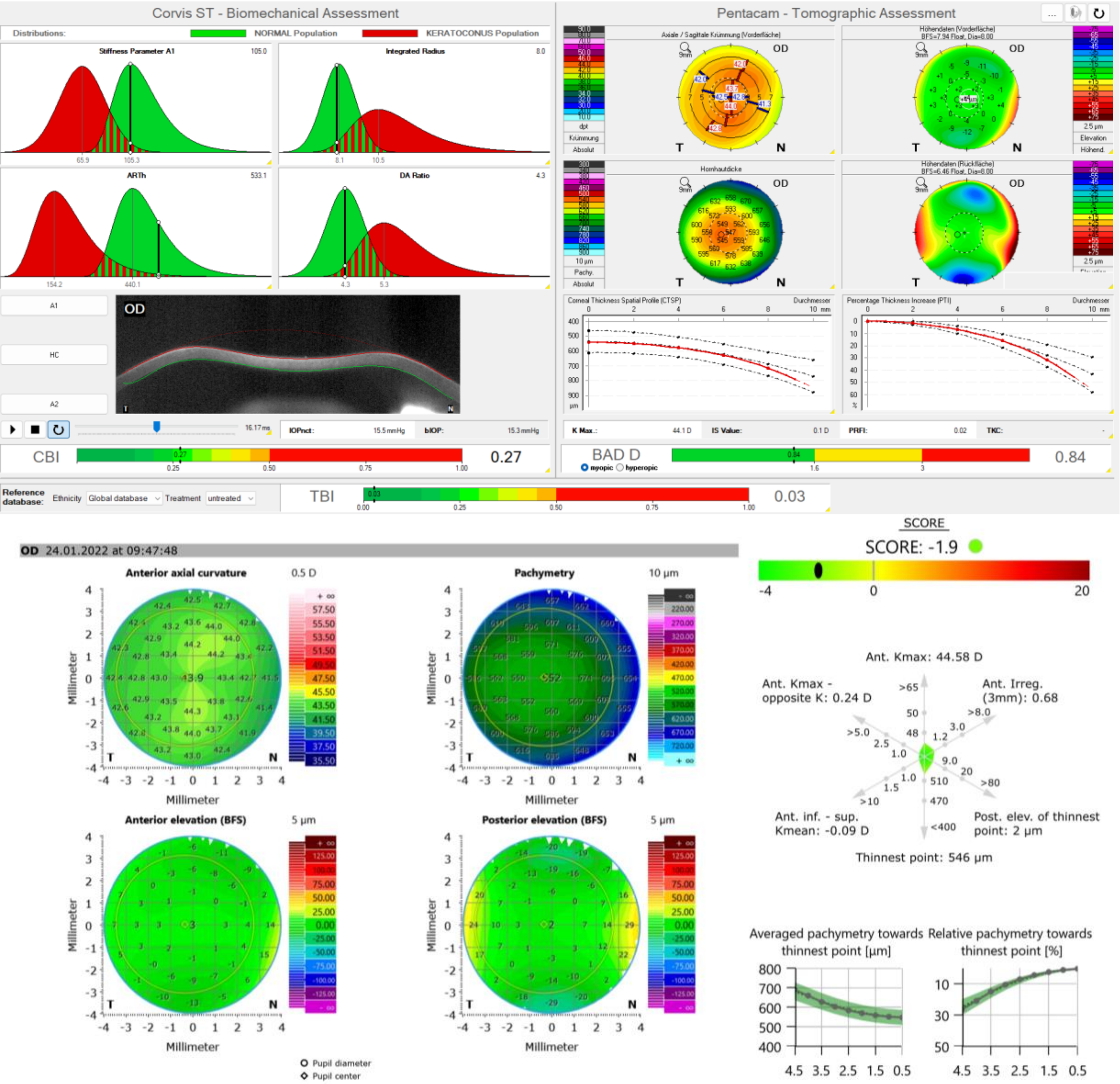

Corvis ST - Biomechanical Assessment
Pentacam - Tomographic Assessment
NORMAL Population
KERATOCONUS Population
Stiffness Parameter A1
Integrated Radius
ARTh
DA Ratio
OD
CBI
0.27
BAD D
0.84
TBI
0.03
OD 24.01.2022 at 09:47:48
Anterior axial curvature
Pachymetry
Anterior elevation (BFS)
Posterior elevation (BFS)
Millimeter
SCORE
SCORE: -1.9
Ant. Kmax: 44.58 D
Ant. Kmax - opposite K: 0.24 D
Ant. Irreg. (3mm): 0.68
Ant. inf. - sup. Kmean: -0.09 D
Post. elev. of thinnest point: 2 µm
Thinnest point: 546 µm
Averaged pachymetry towards thinnest point [µm]
Relative pachymetry towards thinnest point [%]
Pupil diameter
Pupil center

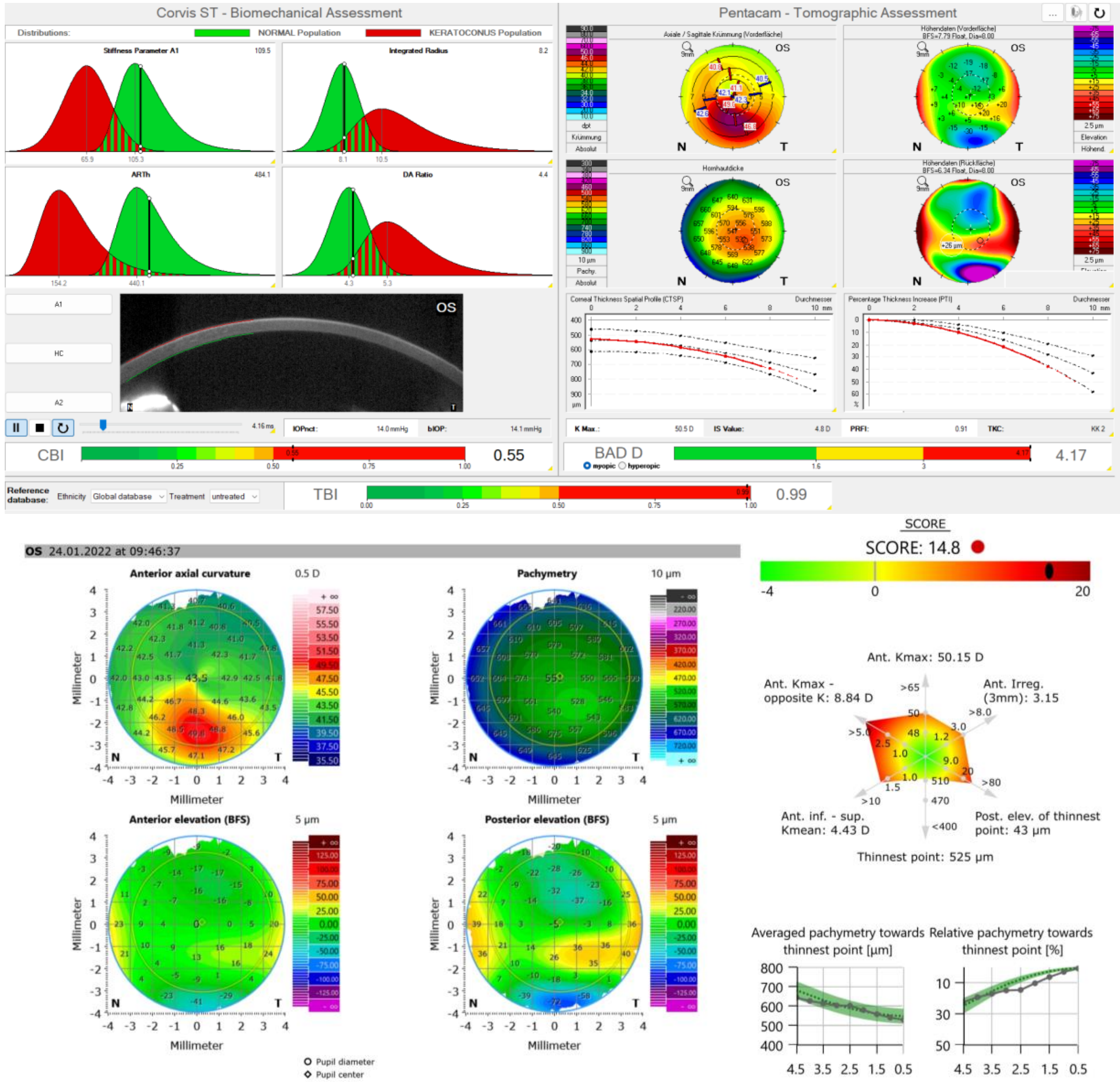

Corvis ST - Biomechanical Assessment
Pentacam - Tomographic Assessment
NORMAL Population
KERATOCONUS Population
Stiffness Parameter A1
Integrated Radius
ARTh
DA Ratio
OS
CBI
0.55
BAD D
4.17
TBI
0.99
OS 24.01.2022 at 09:46:37
Anterior axial curvature
Pachymetry
Anterior elevation (BFS)
Posterior elevation (BFS)
Millimeter
SCORE
SCORE: 14.8
Ant. Kmax: 50.15 D
Ant. Kmax - opposite K: 8.84 D
Ant. Irreg. (3mm): 3.15
Ant. inf. - sup. Kmean: 4.43 D
Post. elev. of thinnest point: 43 µm
Thinnest point: 525 µm
Averaged pachymetry towards thinnest point [µm]
Relative pachymetry towards thinnest point [%]
Pupil diameter
Pupil center

CASE #3

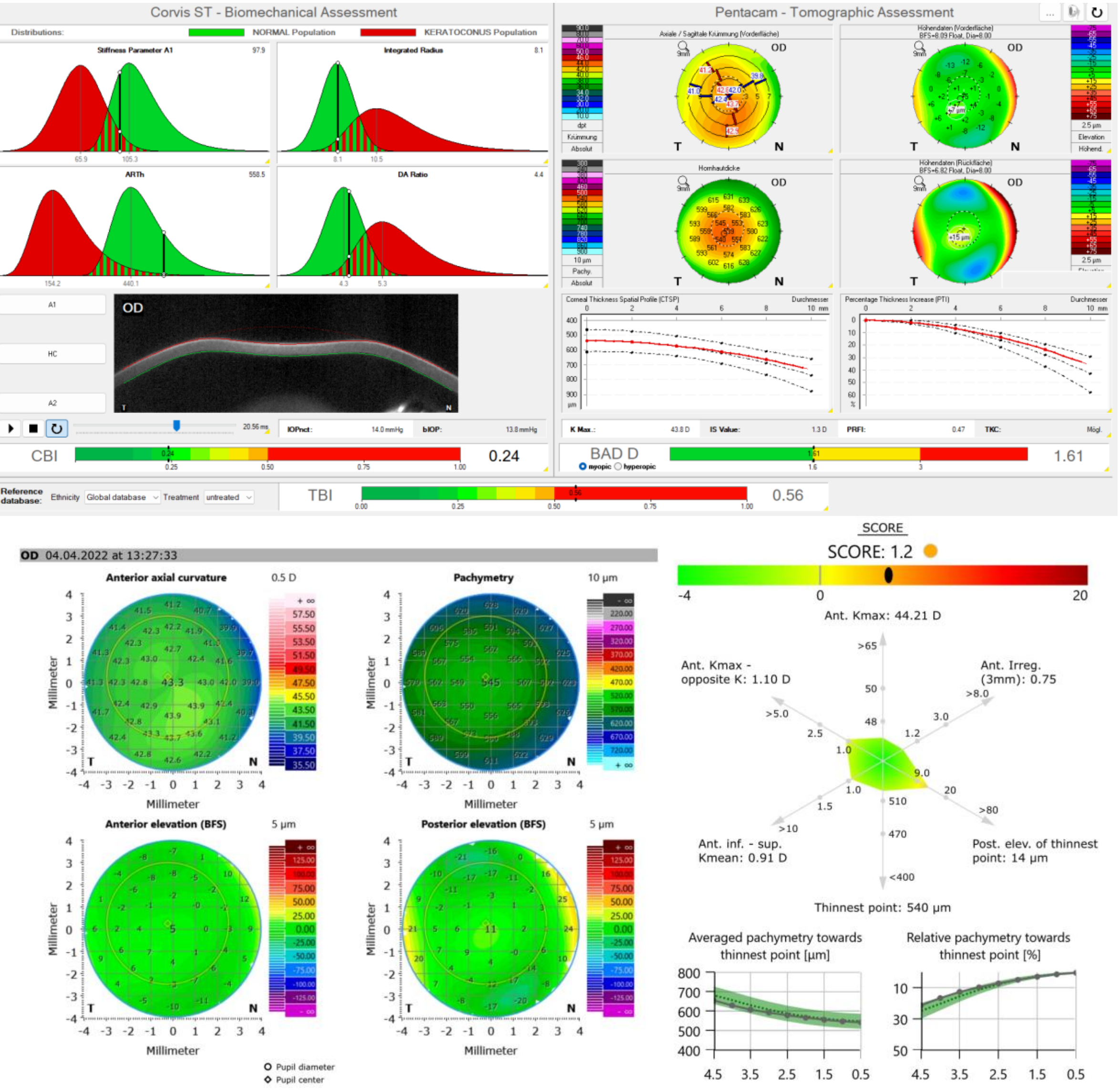

Corvis ST - Biomechanical Assessment
Pentacam - Tomographic Assessment
NORMAL Population
KERATOCONUS Population
OD
CBI 0.24
BAD D 1.61
TBI 0.56
SCORE
OD 04.04.2022 at 13:27:33
Anterior axial curvature
Pachymetry
Anterior elevation (BFS)
Posterior elevation (BFS)
Millimeter
SCORE: 1.2
Ant. Kmax: 44.21 D
Ant. Kmax - opposite K: 1.10 D
Ant. Irreg. (3mm): 0.75
Ant. inf. - sup. Kmean: 0.91 D
Post. elev. of thinnest point: 14 µm
Thinnest point: 540 µm
Averaged pachymetry towards thinnest point [µm]
Relative pachymetry towards thinnest point [%]
Pupil diameter
Pupil center

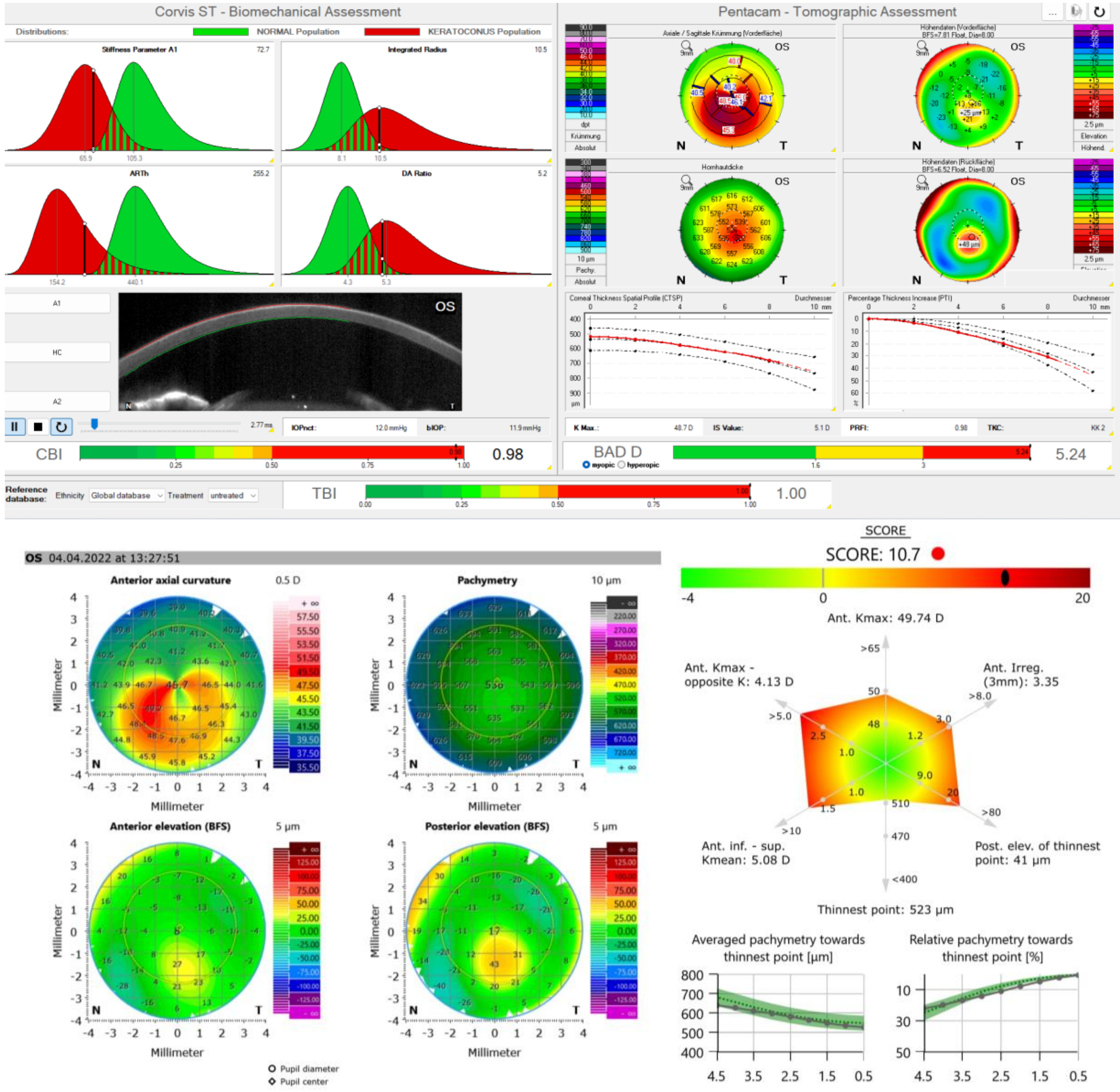

Corvis ST - Biomechanical Assessment
Pentacam - Tomographic Assessment
NORMAL Population
KERATOCONUS Population
OS
CBI 0.98
BAD D 5.24
TBI 1.00
SCORE
OS 04.04.2022 at 13:27:51
Anterior axial curvature
Pachymetry
Anterior elevation (BFS)
Posterior elevation (BFS)
Millimeter
SCORE: 10.7
Ant. Kmax: 49.74 D
Ant. Kmax - opposite K: 4.13 D
Ant. Irreg. (3mm): 3.35
Ant. inf. - sup. Kmean: 5.08 D
Post. elev. of thinnest point: 41 µm
Thinnest point: 523 µm
Averaged pachymetry towards thinnest point [µm]
Relative pachymetry towards thinnest point [%]
Pupil diameter
Pupil center

CASE #4

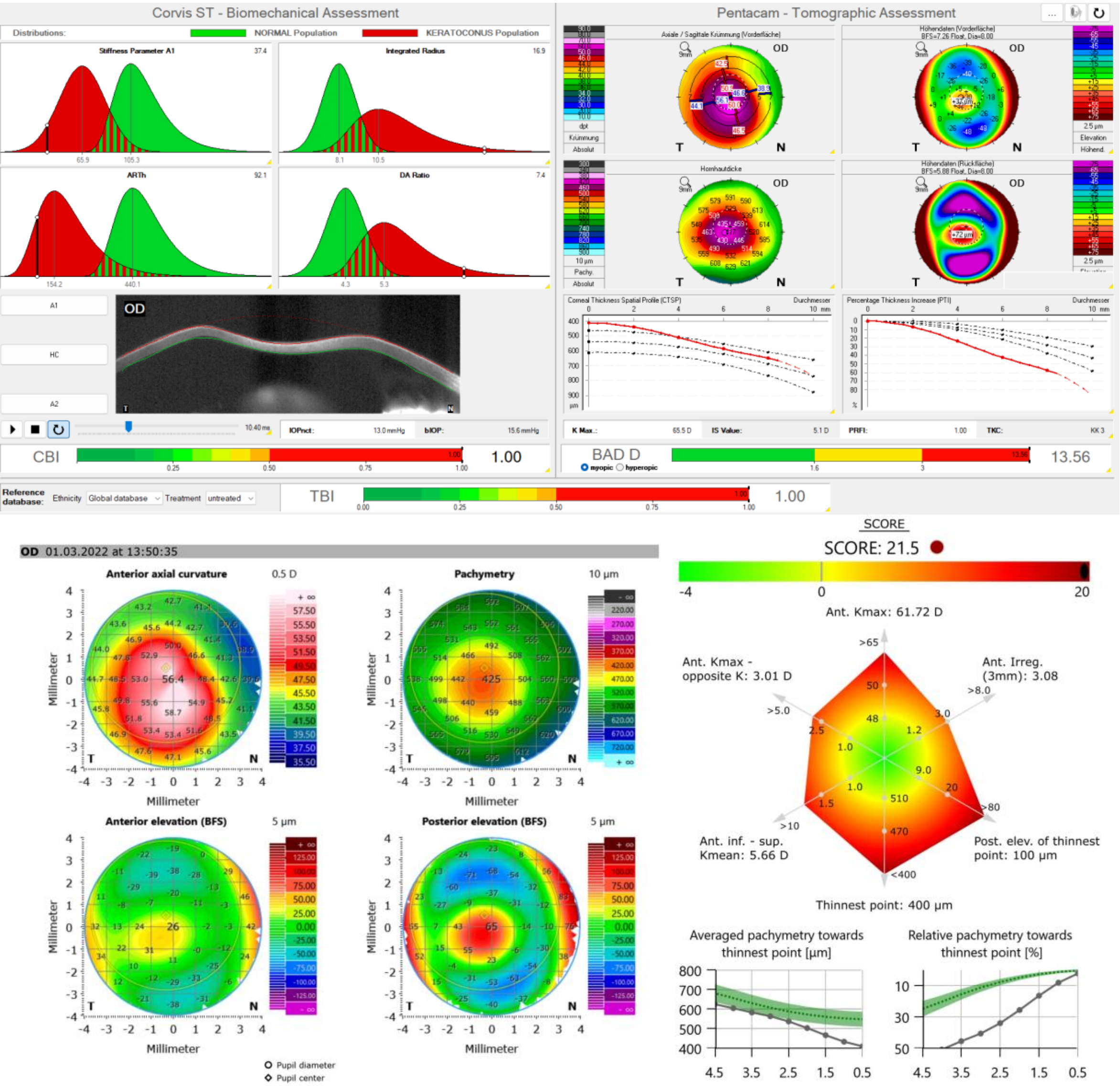

Corvis ST - Biomechanical Assessment
Pentacam - Tomographic Assessment
NORMAL Population
KERATOCONUS Population
Stiffness Parameter A1
Integrated Radius
ARTh
DA Ratio
OD
CBI
1.00
BAD D
13.56
TBI
1.00
OD 01.03.2022 at 13:50:35
Anterior axial curvature
Pachymetry
Anterior elevation (BFS)
Posterior elevation (BFS)
Millimeter
SCORE
SCORE: 21.5
Ant. Kmax: 61.72 D
Ant. Kmax - opposite K: 3.01 D
Ant. Irreg. (3mm): 3.08
Ant. inf. - sup. Kmean: 5.66 D
Post. elev. of thinnest point: 100 µm
Thinnest point: 400 µm
Averaged pachymetry towards thinnest point [µm]
Relative pachymetry towards thinnest point [%]
Pupil diameter
Pupil center

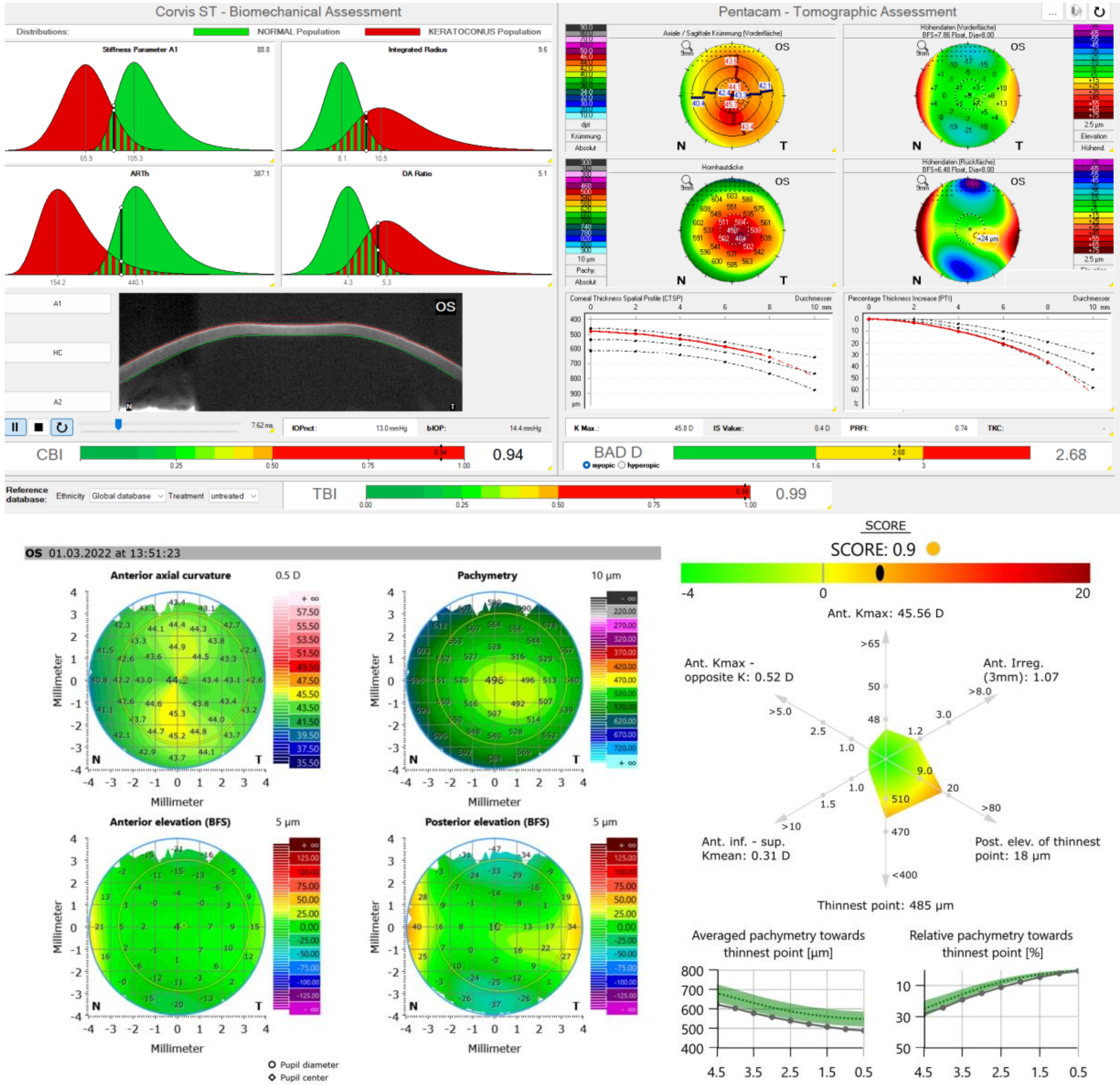

Corvis ST - Biomechanical Assessment
Pentacam - Tomographic Assessment
NORMAL Population
KERATOCONUS Population
Stiffness Parameter A1
Integrated Radius
ARTh
DA Ratio
OS
CBI
0.94
BAD D
2.68
TBI
0.99
OS 01.03.2022 at 13:51:23
Anterior axial curvature
Pachymetry
Anterior elevation (BFS)
Posterior elevation (BFS)
Millimeter
SCORE
SCORE: 0.9
Ant. Kmax: 45.56 D
Ant. Kmax - opposite K: 0.52 D
Ant. Irreg. (3mm): 1.07
Ant. inf. - sup. Kmean: 0.31 D
Post. elev. of thinnest point: 18 µm
Thinnest point: 485 µm
Averaged pachymetry towards thinnest point [µm]
Relative pachymetry towards thinnest point [%]
Pupil diameter
Pupil center

CASE #5

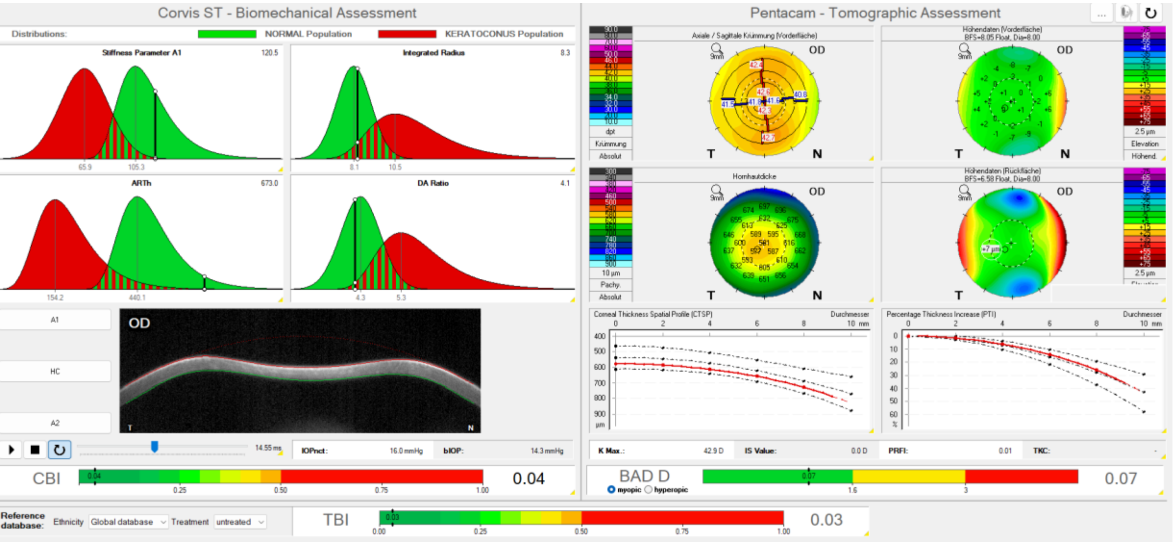

Corvis ST - Biomechanical Assessment
Pentacam - Tomographic Assessment
NORMAL Population
KERATOCONUS Population
Stiffness Parameter A1
Integrated Radius
ARTh
DA Ratio
OD
CBI
0.04
BAD D
0.07
TBI
0.03

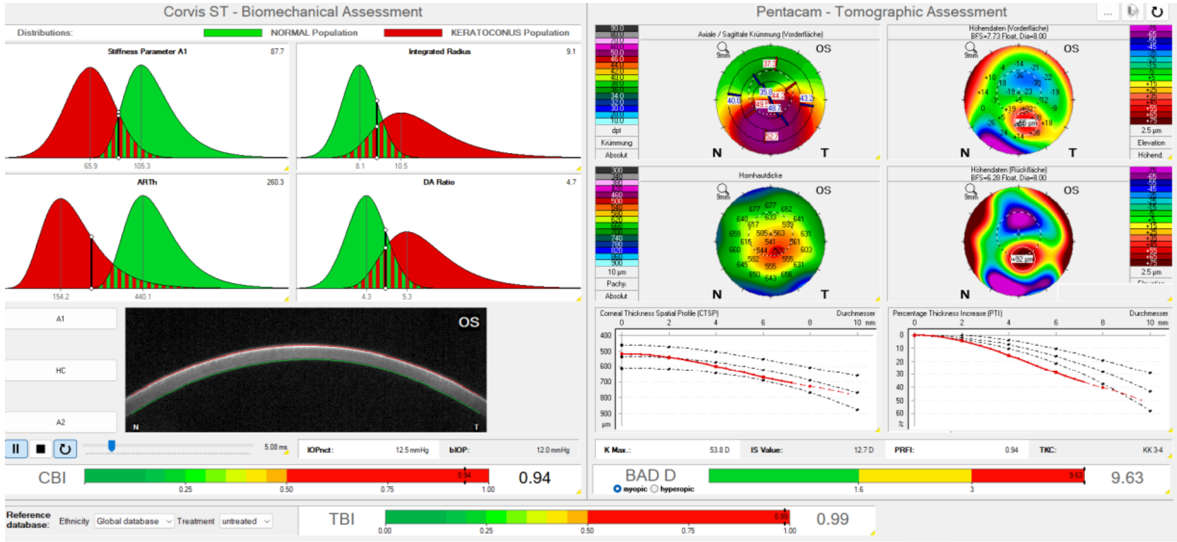

Corvis ST - Biomechanical Assessment
Pentacam - Tomographic Assessment
NORMAL Population
KERATOCONUS Population
Stiffness Parameter A1
Integrated Radius
ARTh
DA Ratio
OS
CBI
0.94
BAD D
9.63
TBI
0.99

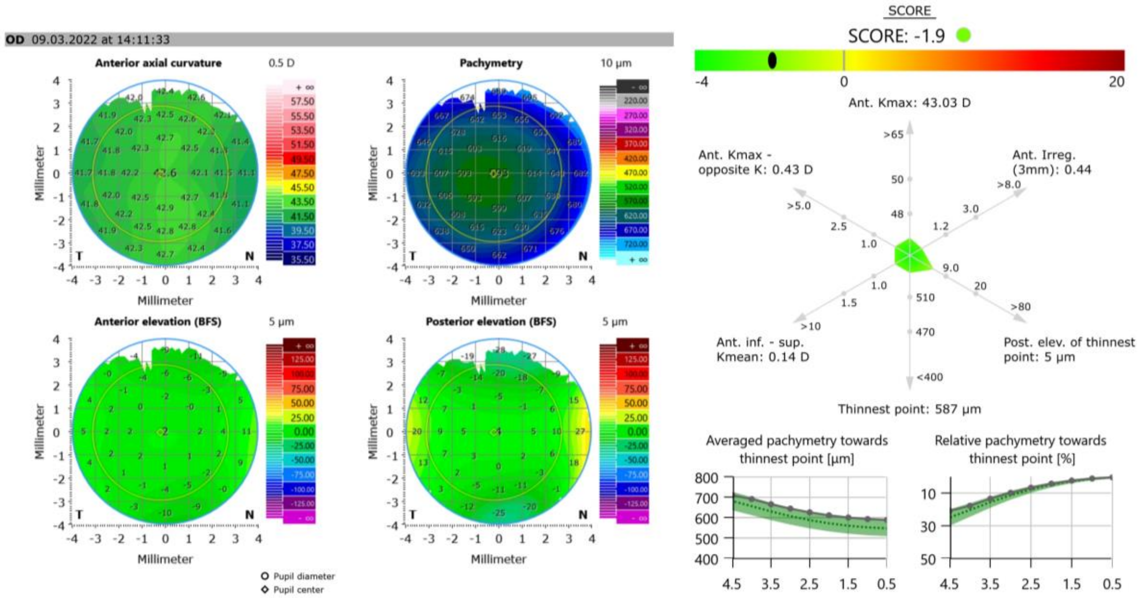

OD 09.03.2022 at 14:11:33
Anterior axial curvature
Pachymetry
Anterior elevation (BFS)
Posterior elevation (BFS)
Millimeter
SCORE
SCORE: -1.9
Ant. Kmax: 43.03 D
Ant. Kmax - opposite K: 0.43 D
Ant. Irreg. (3mm): 0.44
Ant. inf. - sup. Kmean: 0.14 D
Post. elev. of thinnest point: 5 μm
Thinnest point: 587 μm
Averaged pachymetry towards thinnest point [μm]
Relative pachymetry towards thinnest point [%]
Pupil diameter
Pupil center

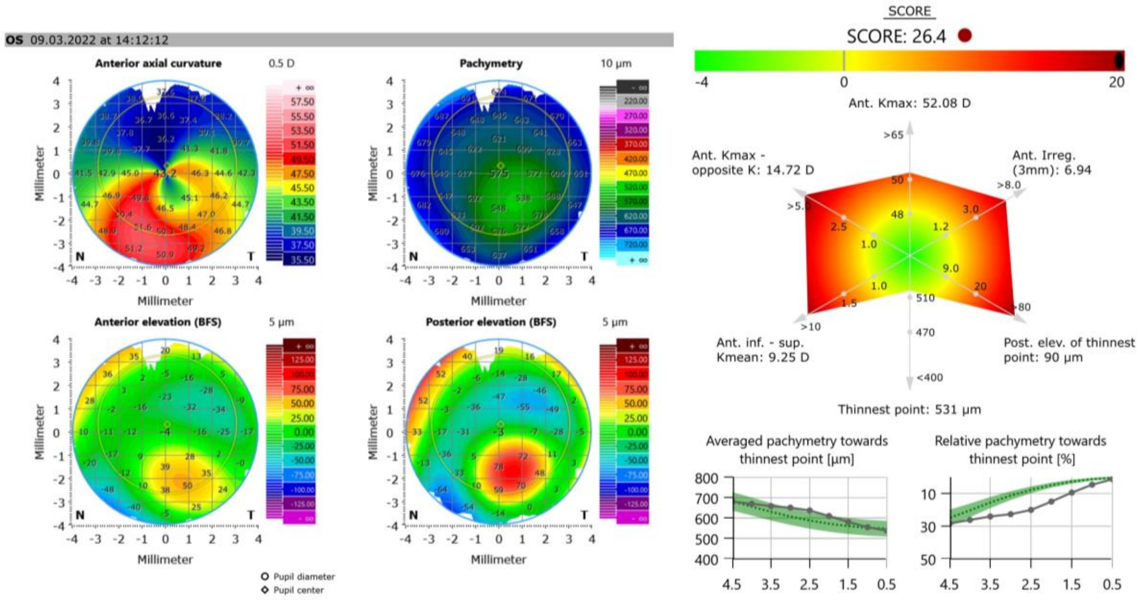

OS 09.03.2022 at 14:12:12
Anterior axial curvature
Pachymetry
Anterior elevation (BFS)
Posterior elevation (BFS)
Millimeter
SCORE
SCORE: 26.4
Ant. Kmax: 52.08 D
Ant. Kmax - opposite K: 14.72 D
Ant. Irreg. (3mm): 6.94
Ant. inf. - sup. Kmean: 9.25 D
Post. elev. of thinnest point: 90 μm
Thinnest point: 531 μm
Averaged pachymetry towards thinnest point [μm]
Relative pachymetry towards thinnest point [%]
Pupil diameter
Pupil center

CASE #6

Excluded for analysis due to prior CXL

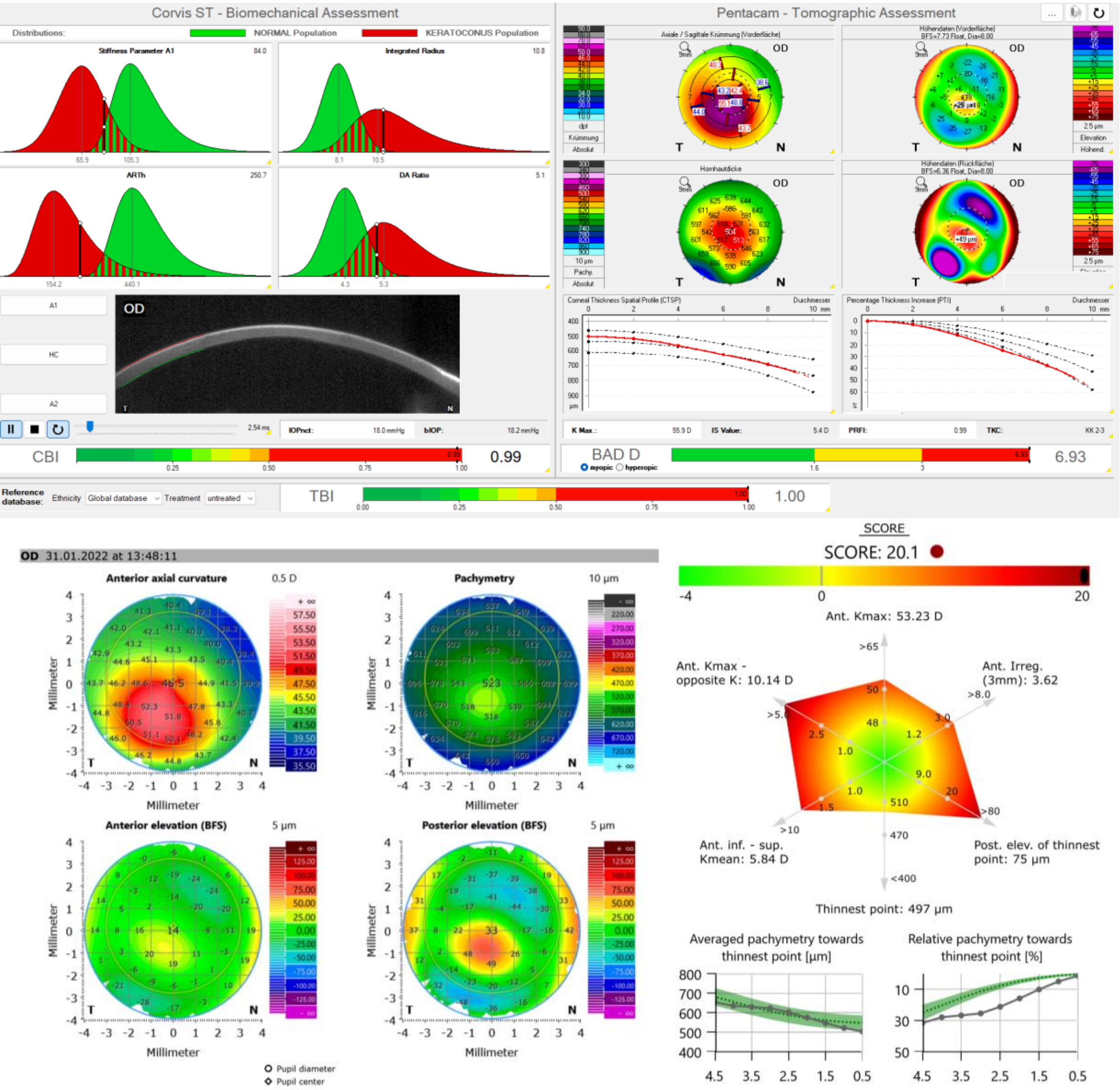

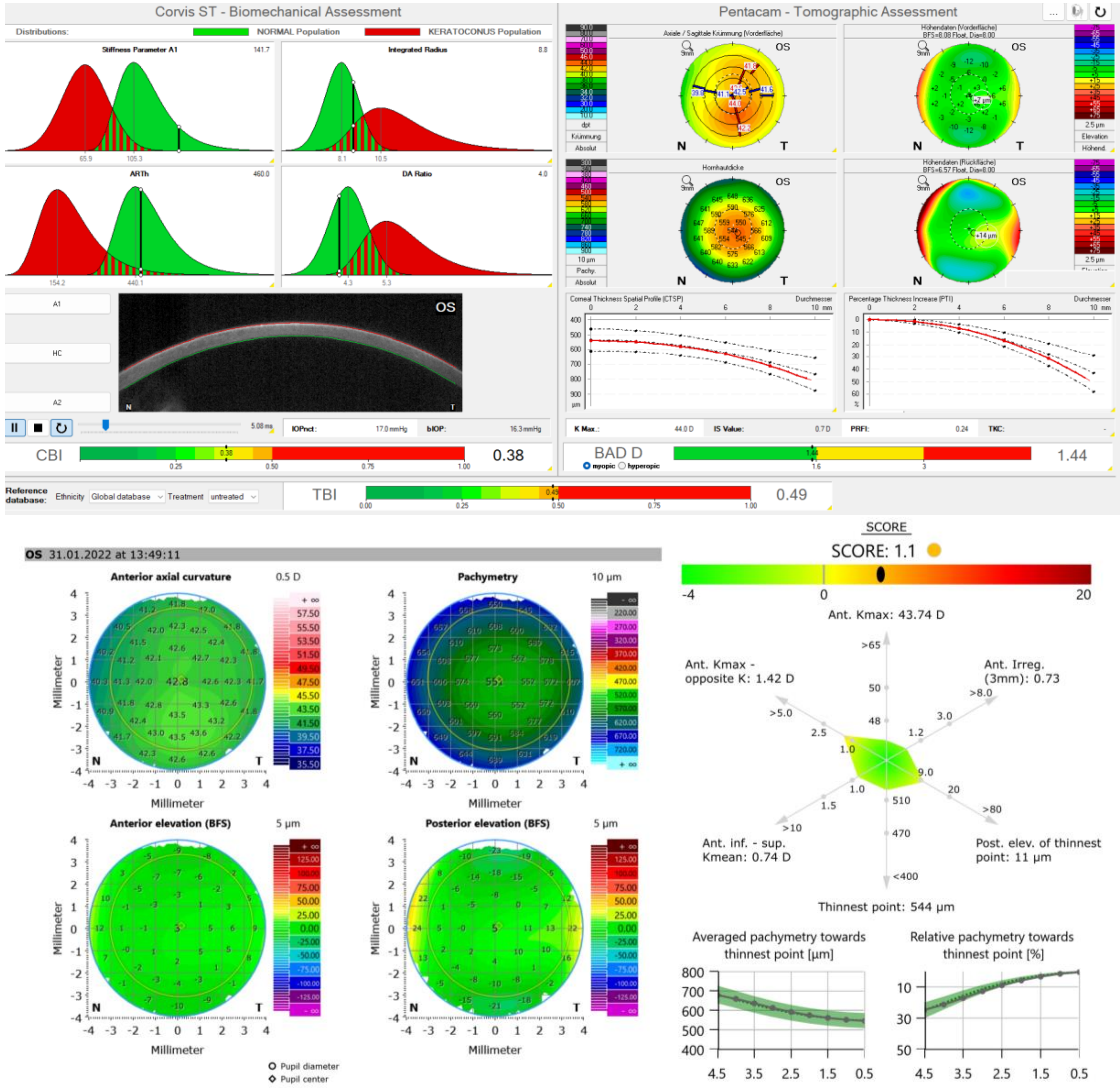

CASE #7

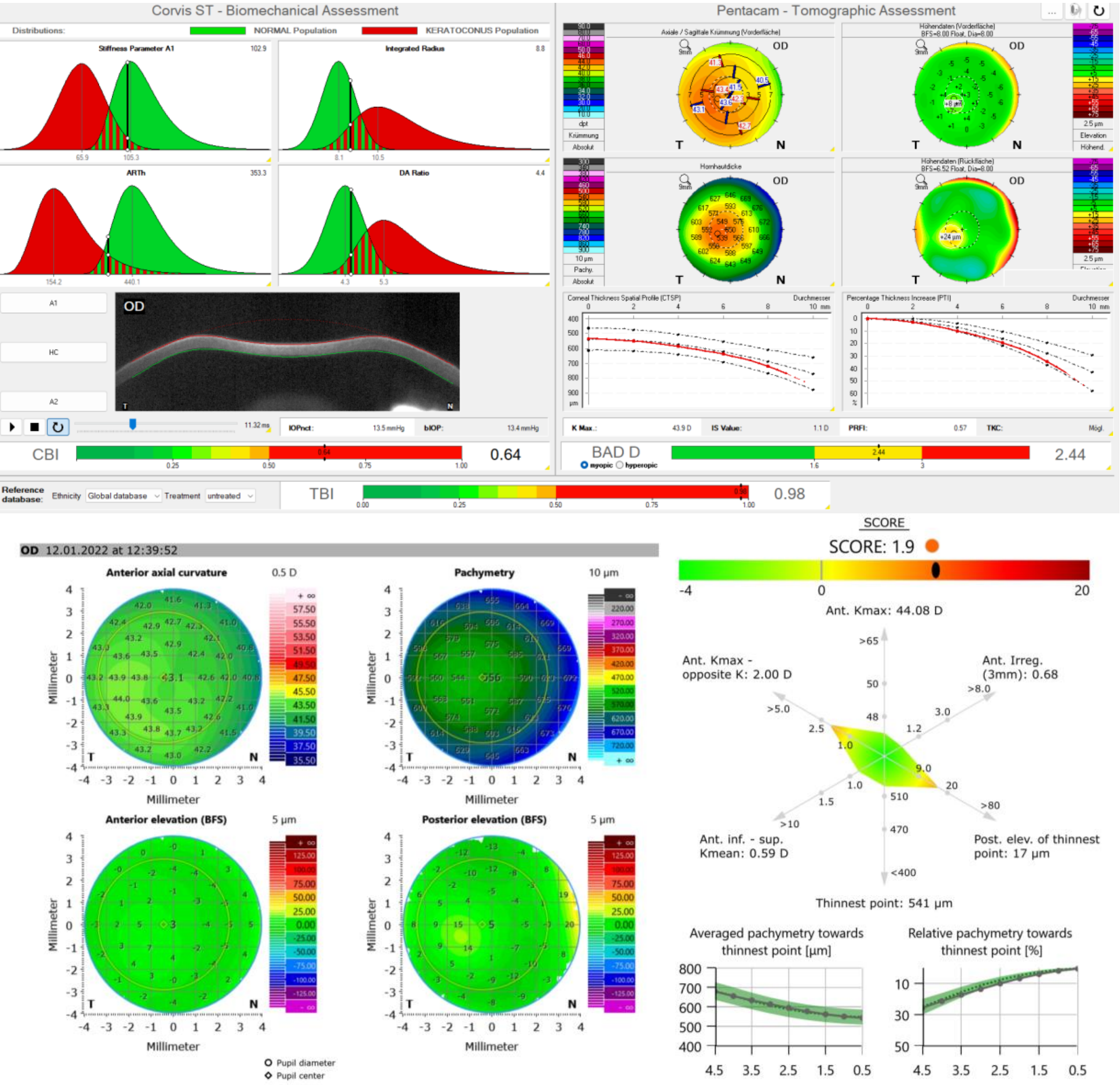

Corvis ST - Biomechanical Assessment
Pentacam - Tomographic Assessment
Distributions: NORMAL Population KERATOCONUS Population
Stiffness Parameter A1
Integrated Radius
ARTh
DA Ratio
OD
CBI 0.64
BAD D 2.44
TBI 0.98
Reference database: Ethnicity Global database Treatment untreated
OD 12.01.2022 at 12:39:52
Anterior axial curvature 0.5 D
Pachymetry 10 µm
Anterior elevation (BFS) 5 µm
Posterior elevation (BFS) 5 µm
Millimeter
Pupil diameter
Pupil center
SCORE
SCORE: 1.9
Ant. Kmax: 44.08 D
Ant. Kmax - opposite K: 2.00 D
Ant. Irreg. (3mm): 0.68
Ant. inf. - sup. Kmean: 0.59 D
Post. elev. of thinnest point: 17 µm
Thinnest point: 541 µm
Averaged pachymetry towards thinnest point [µm]
Relative pachymetry towards thinnest point [%]

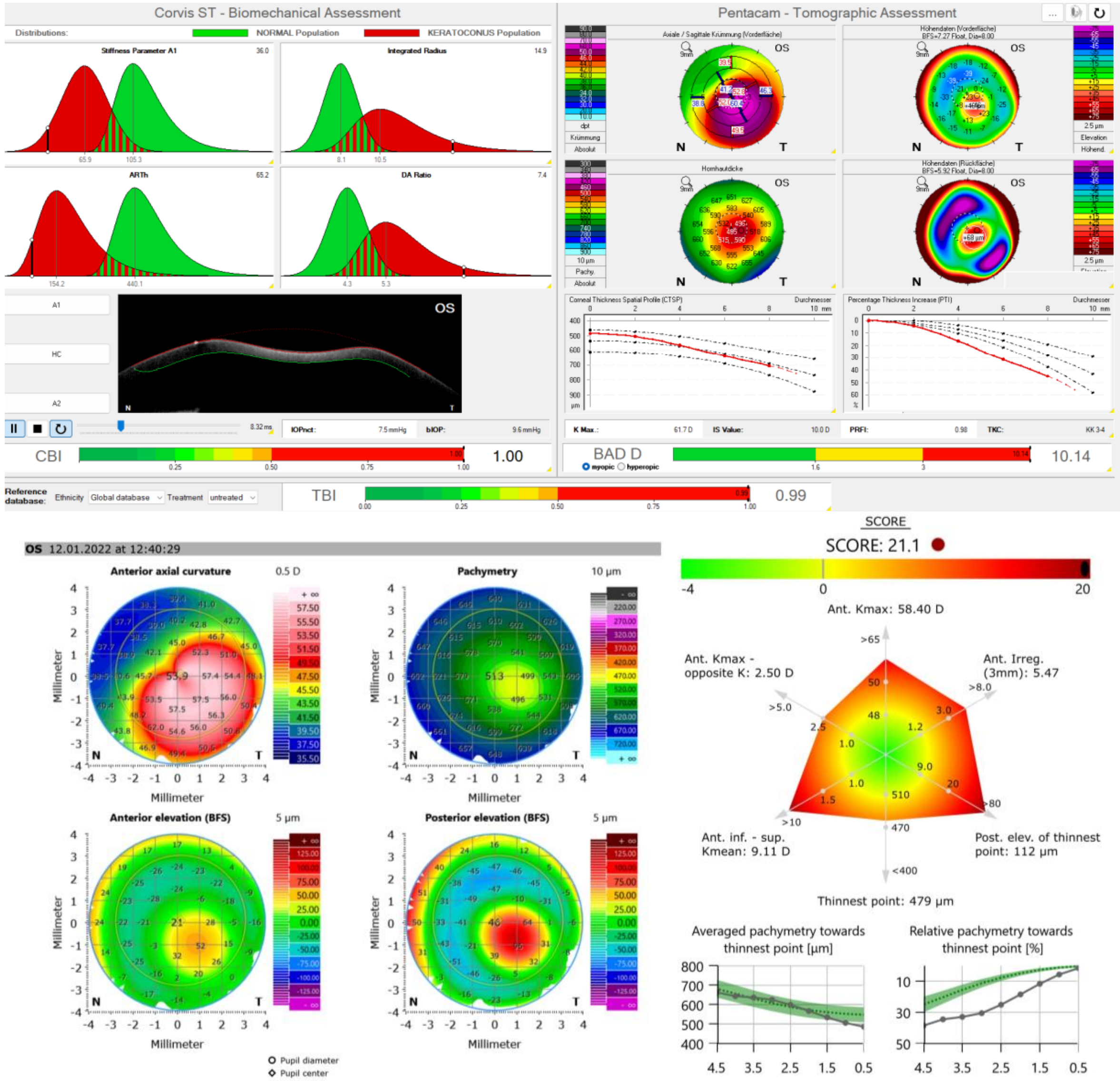

Corvis ST - Biomechanical Assessment
Pentacam - Tomographic Assessment
Distributions: NORMAL Population KERATOCONUS Population
Stiffness Parameter A1
Integrated Radius
ARTh
DA Ratio
OS
CBI 1.00
BAD D 10.14
TBI 0.99
Reference database: Ethnicity Global database Treatment untreated
OS 12.01.2022 at 12:40:29
Anterior axial curvature 0.5 D
Pachymetry 10 µm
Anterior elevation (BFS) 5 µm
Posterior elevation (BFS) 5 µm
Millimeter
Pupil diameter
Pupil center
SCORE
SCORE: 21.1
Ant. Kmax: 58.40 D
Ant. Kmax - opposite K: 2.50 D
Ant. Irreg. (3mm): 5.47
Ant. inf. - sup. Kmean: 9.11 D
Post. elev. of thinnest point: 112 µm
Thinnest point: 479 µm
Averaged pachymetry towards thinnest point [µm]
Relative pachymetry towards thinnest point [%]

CASE #8

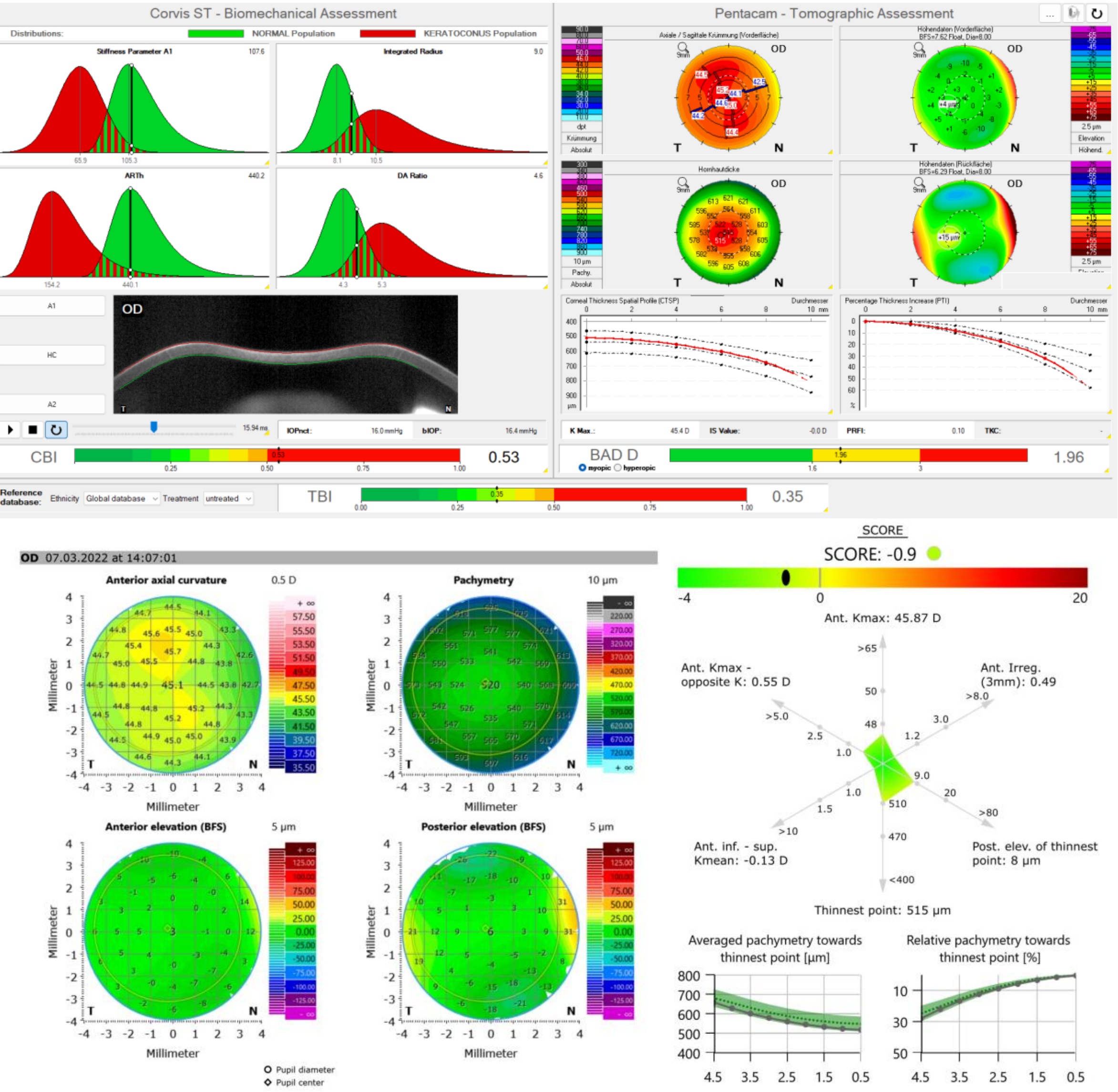

Corvis ST - Biomechanical Assessment
Pentacam - Tomographic Assessment
NORMAL Population
KERATOCONUS Population
Stiffness Parameter A1
Integrated Radius
ARTh
DA Ratio
OD
CBI 0.53
BAD D 1.96
TBI 0.35
SCORE
OD 07.03.2022 at 14:07:01
Anterior axial curvature
Pachymetry
Anterior elevation (BFS)
Posterior elevation (BFS)
Millimeter
SCORE: -0.9
Ant. Kmax: 45.87 D
Ant. Kmax - opposite K: 0.55 D
Ant. Irreg. (3mm): 0.49
Ant. inf. - sup. Kmean: -0.13 D
Post. elev. of thinnest point: 8 μm
Thinnest point: 515 μm
Averaged pachymetry towards thinnest point [μm]
Relative pachymetry towards thinnest point [%]
Pupil diameter
Pupil center

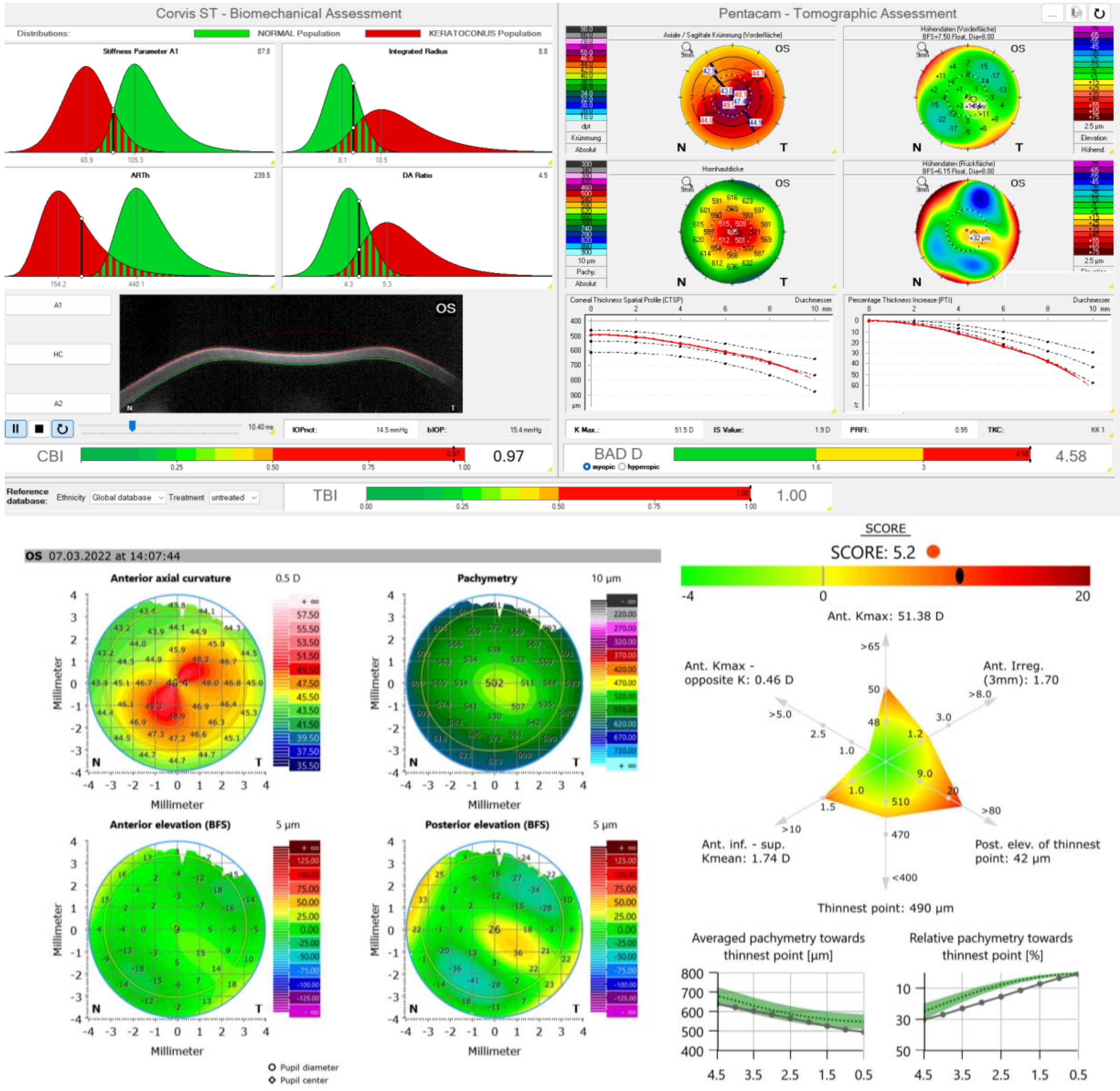

Corvis ST - Biomechanical Assessment
Pentacam - Tomographic Assessment
NORMAL Population
KERATOCONUS Population
Stiffness Parameter A1
Integrated Radius
ARTh
DA Ratio
OS
CBI 0.97
BAD D 4.58
TBI 1.00
SCORE
OS 07.03.2022 at 14:07:44
Anterior axial curvature
Pachymetry
Anterior elevation (BFS)
Posterior elevation (BFS)
Millimeter
SCORE: 5.2
Ant. Kmax: 51.38 D
Ant. Kmax - opposite K: 0.46 D
Ant. Irreg. (3mm): 1.70
Ant. inf. - sup. Kmean: 1.74 D
Post. elev. of thinnest point: 42 μm
Thinnest point: 490 μm
Averaged pachymetry towards thinnest point [μm]
Relative pachymetry towards thinnest point [%]
Pupil diameter
Pupil center

CASE #9

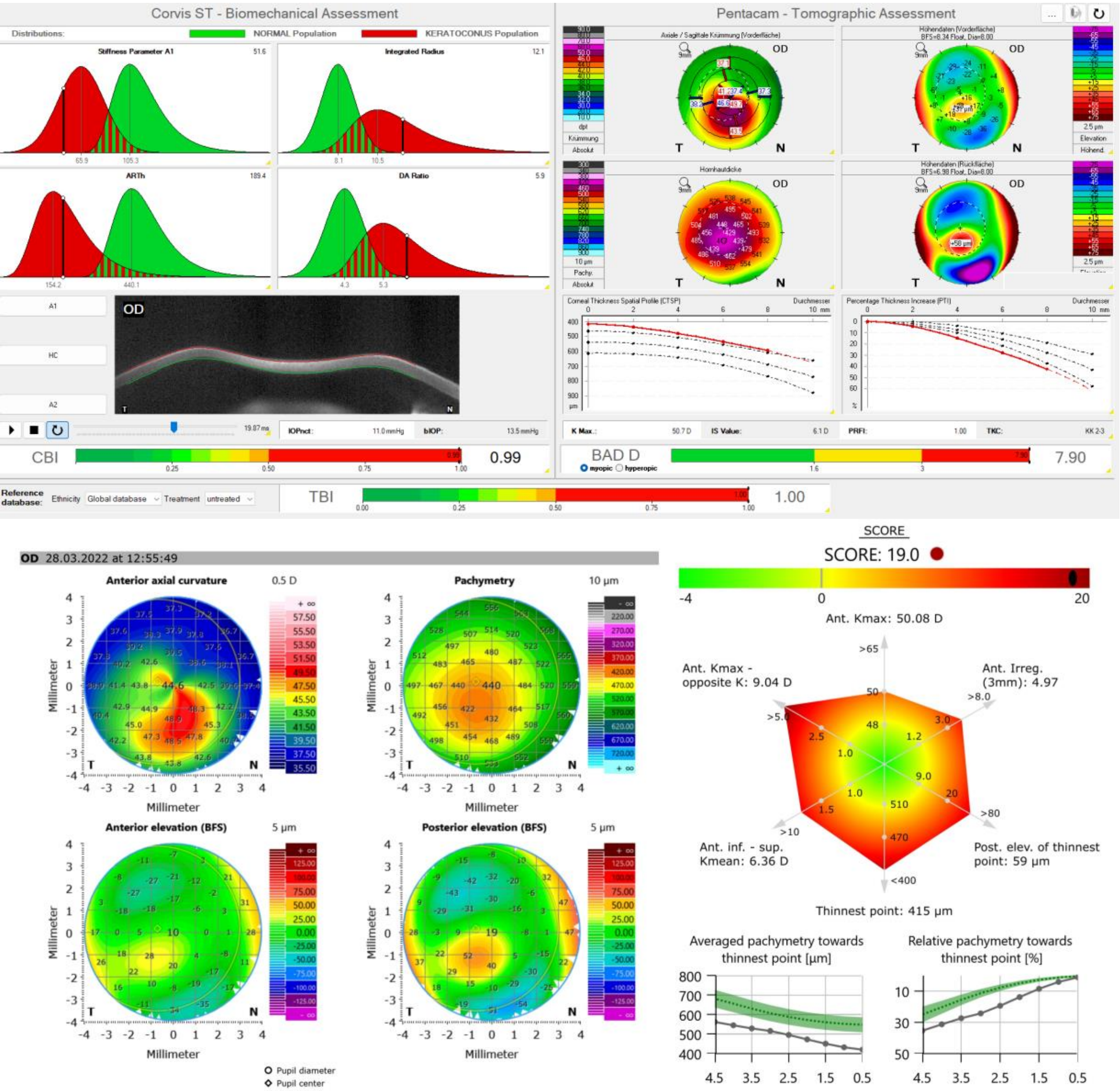
Corvis ST - Biomechanical Assessment
Pentacam - Tomographic Assessment
OD
CBI 0.99
BAD D 7.90
TBI 1.00
SCORE
OD 28.03.2022 at 12:55:49
Anterior axial curvature
Pachymetry
Anterior elevation (BFS)
Posterior elevation (BFS)
SCORE: 19.0
Ant. Kmax: 50.08 D
Ant. Kmax - opposite K: 9.04 D
Ant. Irreg. (3mm): 4.97
Ant. inf. - sup. Kmean: 6.36 D
Post. elev. of thinnest point: 59 µm
Thinnest point: 415 µm
Averaged pachymetry towards thinnest point [µm]
Relative pachymetry towards thinnest point [%]
Millimeter
Pupil diameter
Pupil center

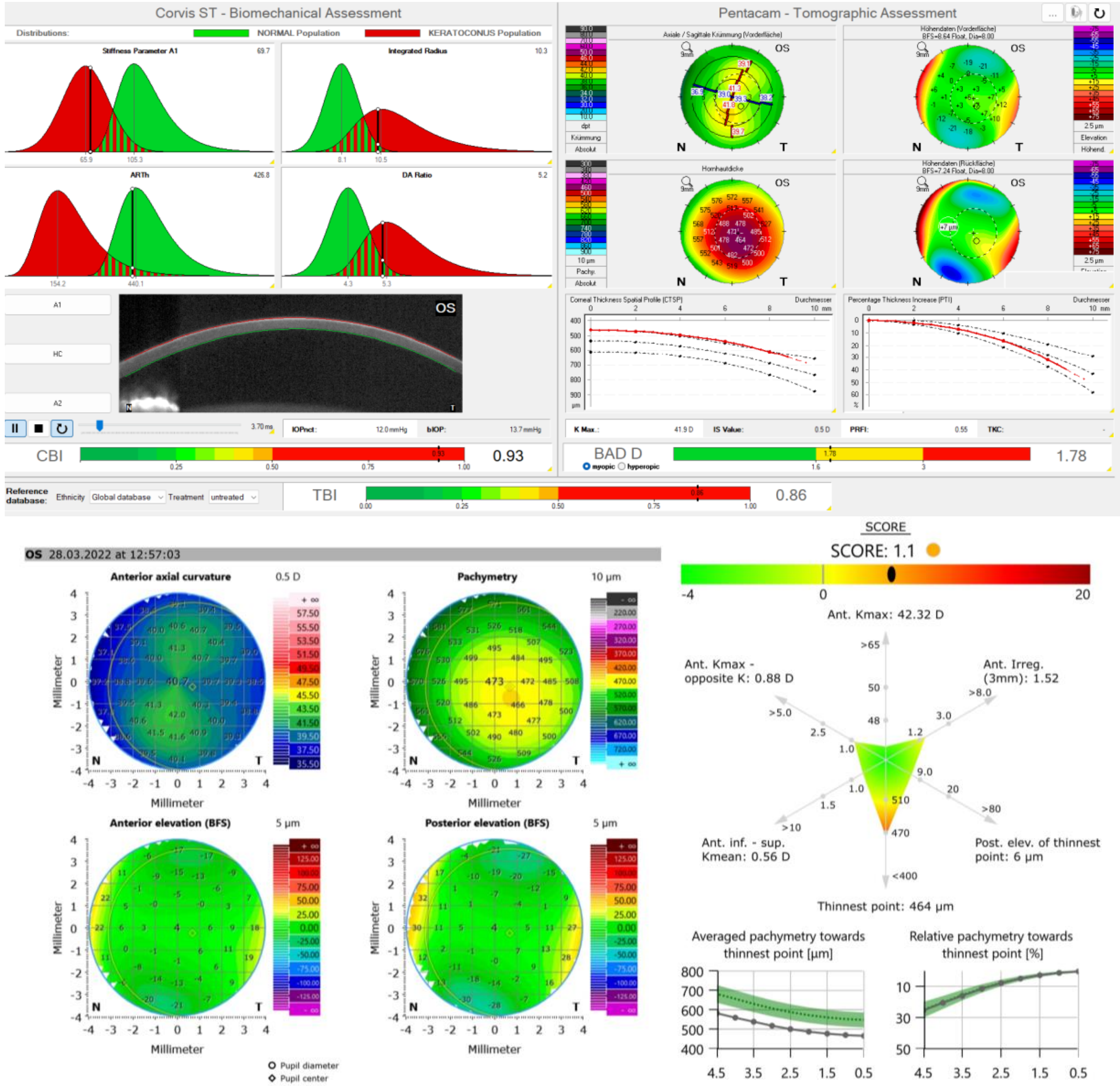
Corvis ST - Biomechanical Assessment
Pentacam - Tomographic Assessment
OS
CBI 0.93
BAD D 1.78
TBI 0.86
SCORE
OS 28.03.2022 at 12:57:03
Anterior axial curvature
Pachymetry
Anterior elevation (BFS)
Posterior elevation (BFS)
SCORE: 1.1
Ant. Kmax: 42.32 D
Ant. Kmax - opposite K: 0.88 D
Ant. Irreg. (3mm): 1.52
Ant. inf. - sup. Kmean: 0.56 D
Post. elev. of thinnest point: 6 µm
Thinnest point: 464 µm
Averaged pachymetry towards thinnest point [µm]
Relative pachymetry towards thinnest point [%]
Millimeter
Pupil diameter
Pupil center

CASE #10

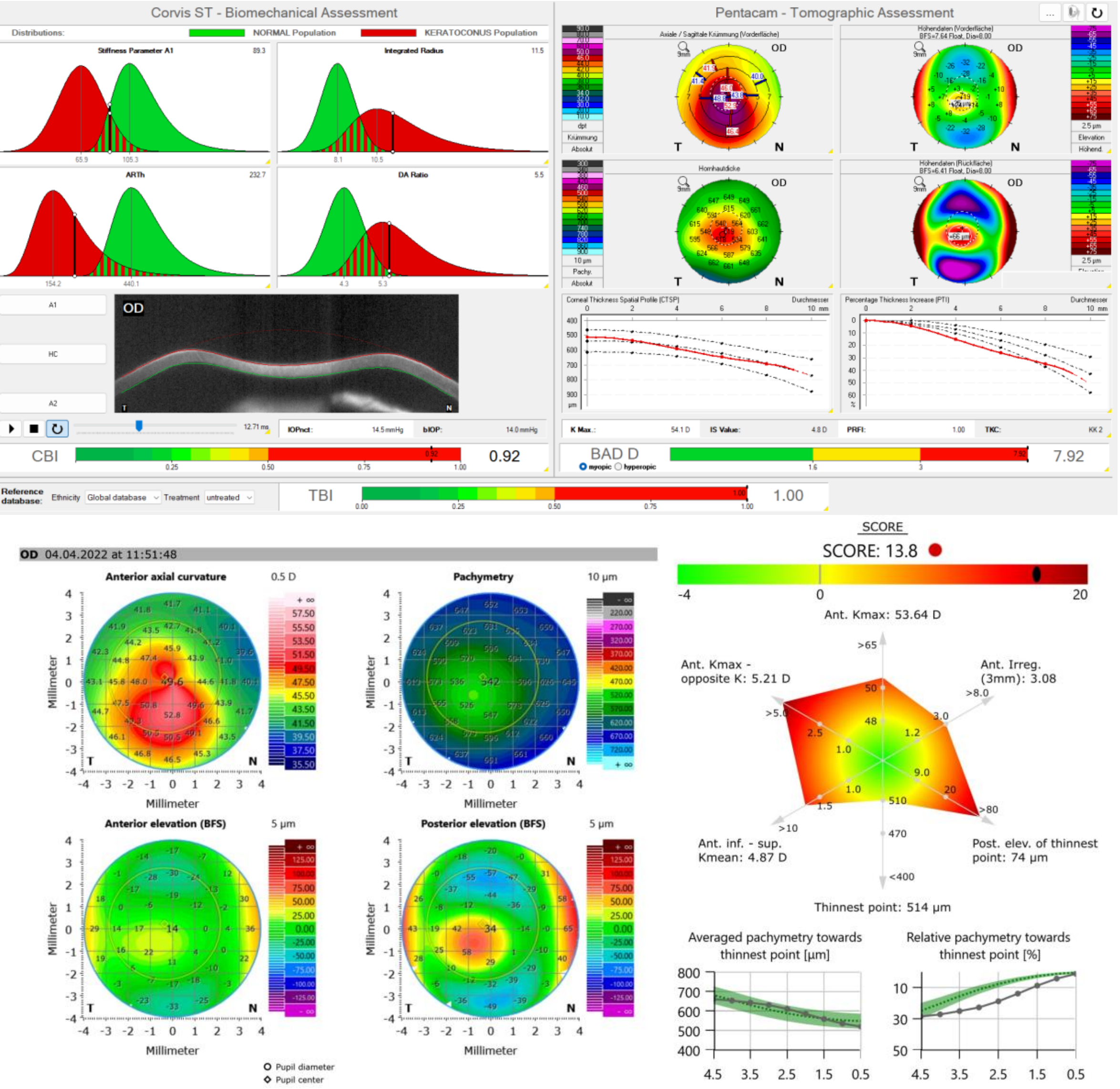
Corvis ST - Biomechanical Assessment
Pentacam - Tomographic Assessment
NORMAL Population
KERATOCONUS Population
Stiffness Parameter A1
Integrated Radius
ARTh
DA Ratio
OD
CBI
0.92
BAD D
7.92
TBI
1.00
SCORE
OD 04.04.2022 at 11:51:48
Anterior axial curvature
Pachymetry
Anterior elevation (BFS)
Posterior elevation (BFS)
Millimeter
SCORE: 13.8
Ant. Kmax: 53.64 D
Ant. Kmax - opposite K: 5.21 D
Ant. Irreg. (3mm): 3.08
Ant. inf. - sup. Kmean: 4.87 D
Post. elev. of thinnest point: 74 µm
Thinnest point: 514 µm
Averaged pachymetry towards thinnest point [µm]
Relative pachymetry towards thinnest point [%]
Pupil diameter
Pupil center

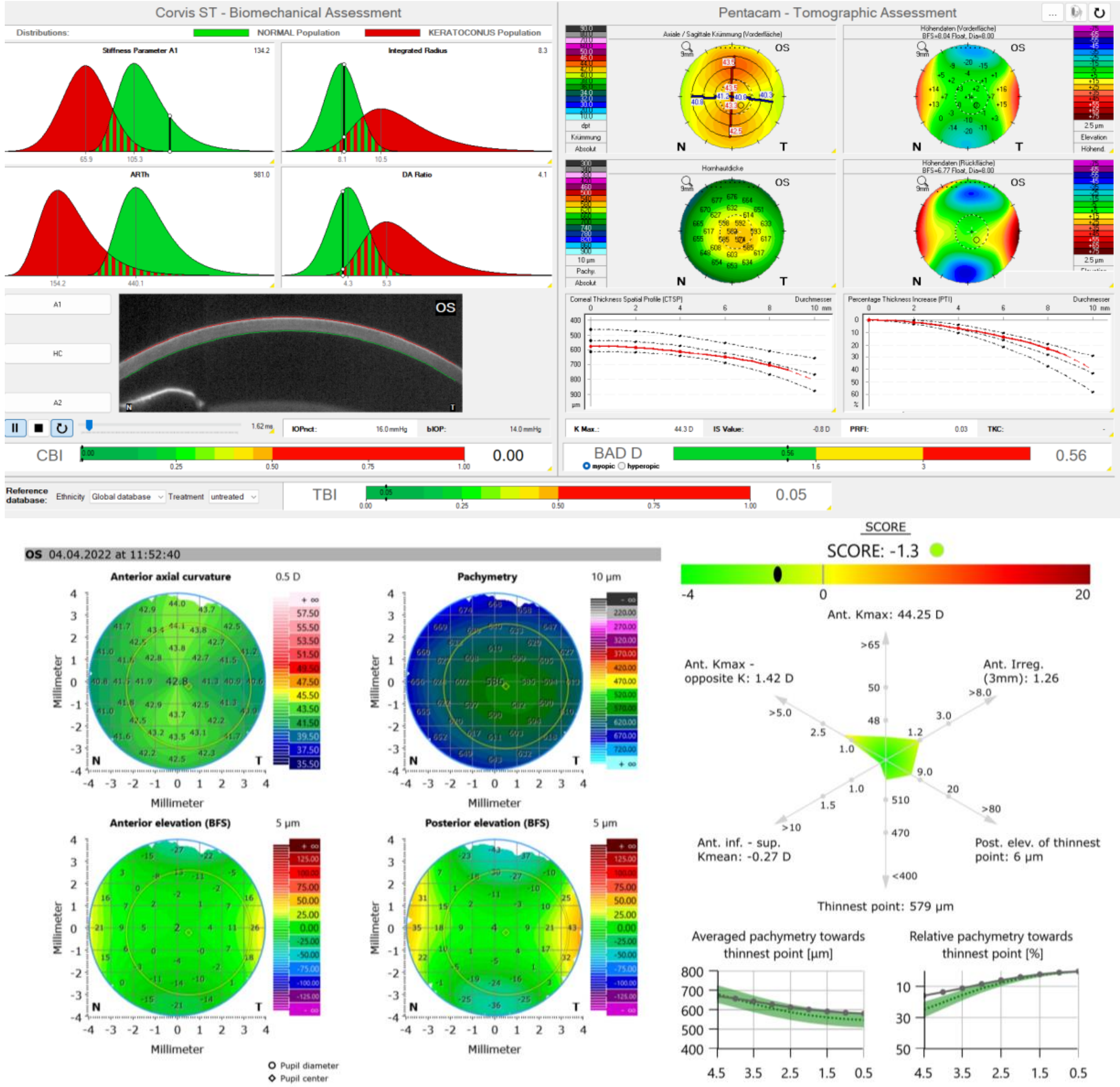
Corvis ST - Biomechanical Assessment
Pentacam - Tomographic Assessment
NORMAL Population
KERATOCONUS Population
Stiffness Parameter A1
Integrated Radius
ARTh
DA Ratio
OS
CBI
0.00
BAD D
0.56
TBI
0.05
SCORE
OS 04.04.2022 at 11:52:40
Anterior axial curvature
Pachymetry
Anterior elevation (BFS)
Posterior elevation (BFS)
Millimeter
SCORE: -1.3
Ant. Kmax: 44.25 D
Ant. Kmax - opposite K: 1.42 D
Ant. Irreg. (3mm): 1.26
Ant. inf. - sup. Kmean: -0.27 D
Post. elev. of thinnest point: 6 µm
Thinnest point: 579 µm
Averaged pachymetry towards thinnest point [µm]
Relative pachymetry towards thinnest point [%]
Pupil diameter
Pupil center

CASE #11

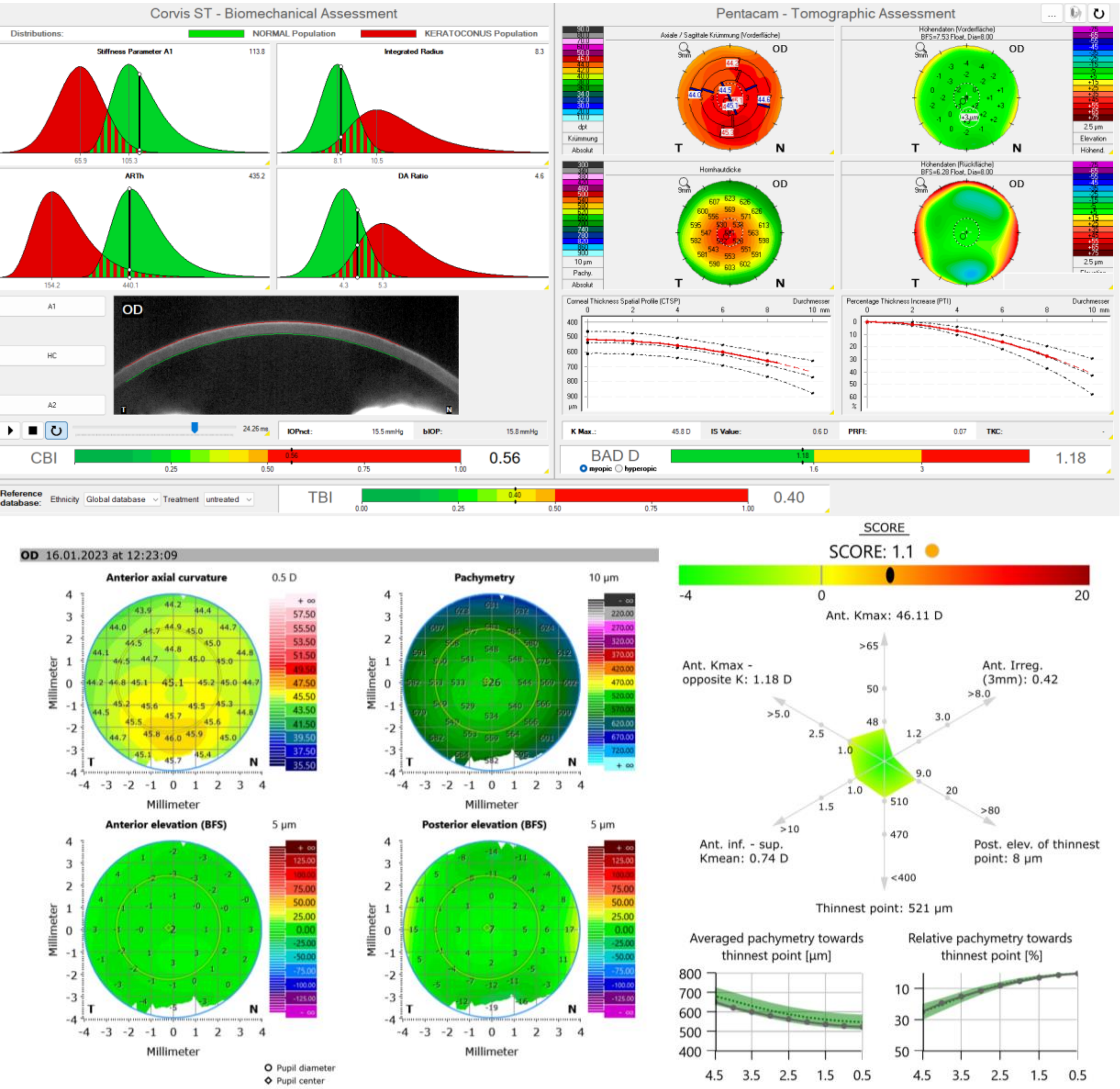

Corvis ST - Biomechanical Assessment
Pentacam - Tomographic Assessment
OD
CBI 0.56
BAD D 1.18
TBI 0.40
OD 16.01.2023 at 12:23:09
Anterior axial curvature
Pachymetry
Anterior elevation (BFS)
Posterior elevation (BFS)
Millimeter
SCORE
SCORE: 1.1
Ant. Kmax: 46.11 D
Ant. Kmax - opposite K: 1.18 D
Ant. Irreg. (3mm): 0.42
Ant. inf. - sup. Kmean: 0.74 D
Post. elev. of thinnest point: 8 µm
Thinnest point: 521 µm
Averaged pachymetry towards thinnest point [µm]
Relative pachymetry towards thinnest point [%]
Pupil diameter
Pupil center

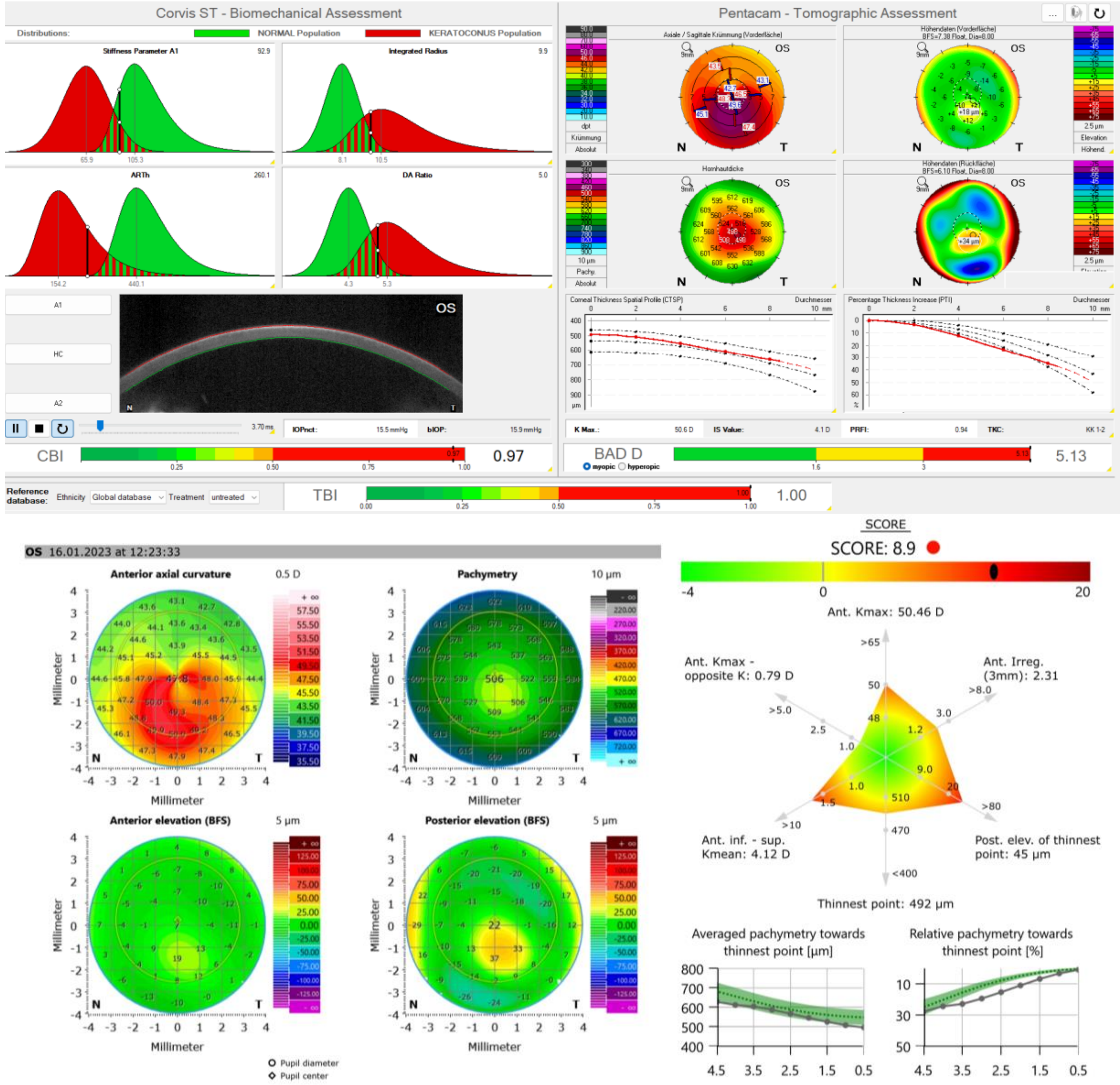

Corvis ST - Biomechanical Assessment
Pentacam - Tomographic Assessment
OS
CBI 0.97
BAD D 5.13
TBI 1.00
OS 16.01.2023 at 12:23:33
Anterior axial curvature
Pachymetry
Anterior elevation (BFS)
Posterior elevation (BFS)
Millimeter
SCORE
SCORE: 8.9
Ant. Kmax: 50.46 D
Ant. Kmax - opposite K: 0.79 D
Ant. Irreg. (3mm): 2.31
Ant. inf. - sup. Kmean: 4.12 D
Post. elev. of thinnest point: 45 µm
Thinnest point: 492 µm
Averaged pachymetry towards thinnest point [µm]
Relative pachymetry towards thinnest point [%]
Pupil diameter
Pupil center

CASE #12

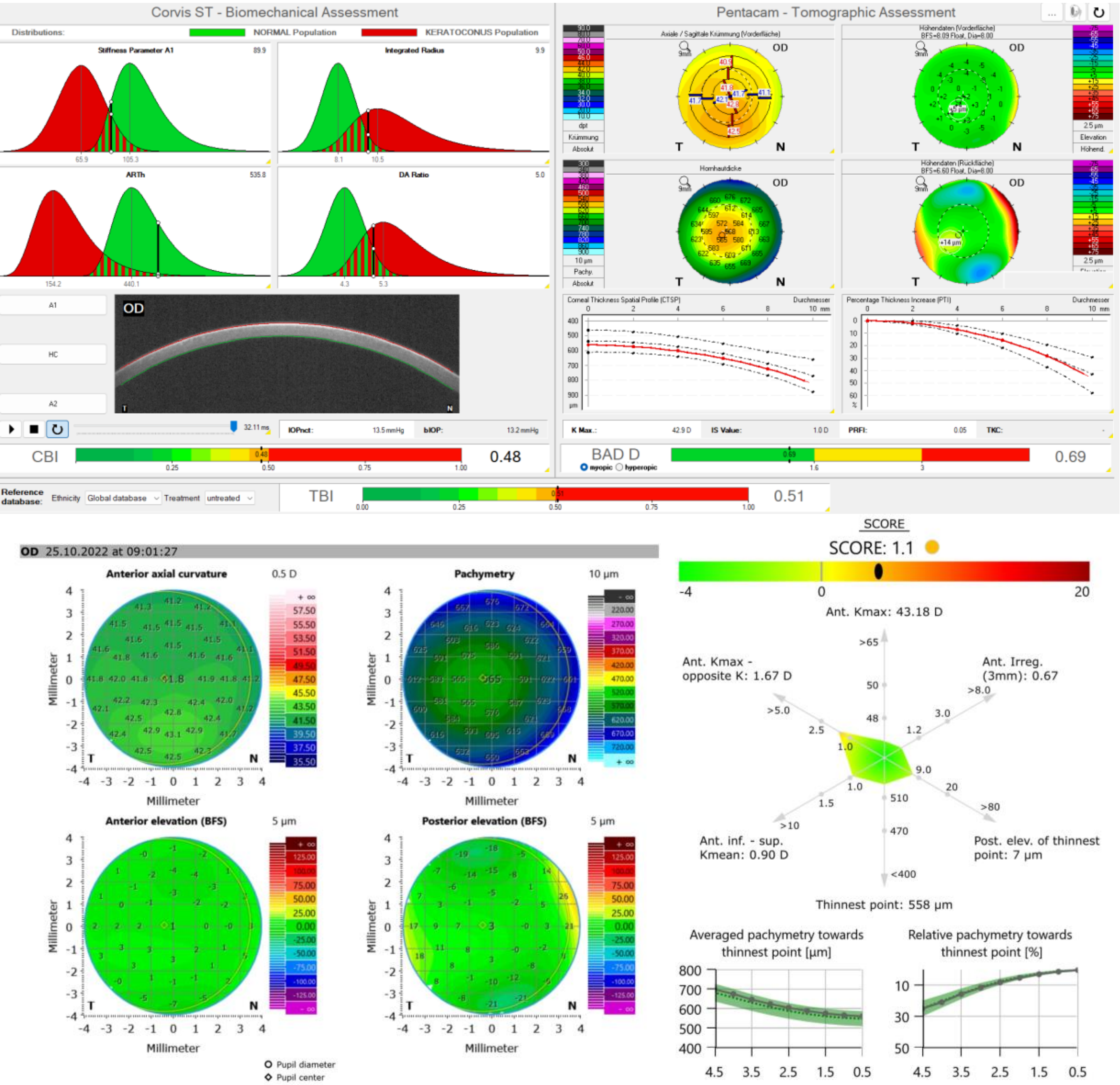
Corvis ST - Biomechanical Assessment
Pentacam - Tomographic Assessment
OD
CBI
0.48
BAD D
0.69
TBI
0.51
OD 25.10.2022 at 09:01:27
Anterior axial curvature
Pachymetry
Anterior elevation (BFS)
Posterior elevation (BFS)
Millimeter
SCORE
SCORE: 1.1
Ant. Kmax: 43.18 D
Ant. Kmax - opposite K: 1.67 D
Ant. Irreg. (3mm): 0.67
Ant. inf. - sup. Kmean: 0.90 D
Post. elev. of thinnest point: 7 µm
Thinnest point: 558 µm
Averaged pachymetry towards thinnest point [µm]
Relative pachymetry towards thinnest point [%]
Pupil diameter
Pupil center

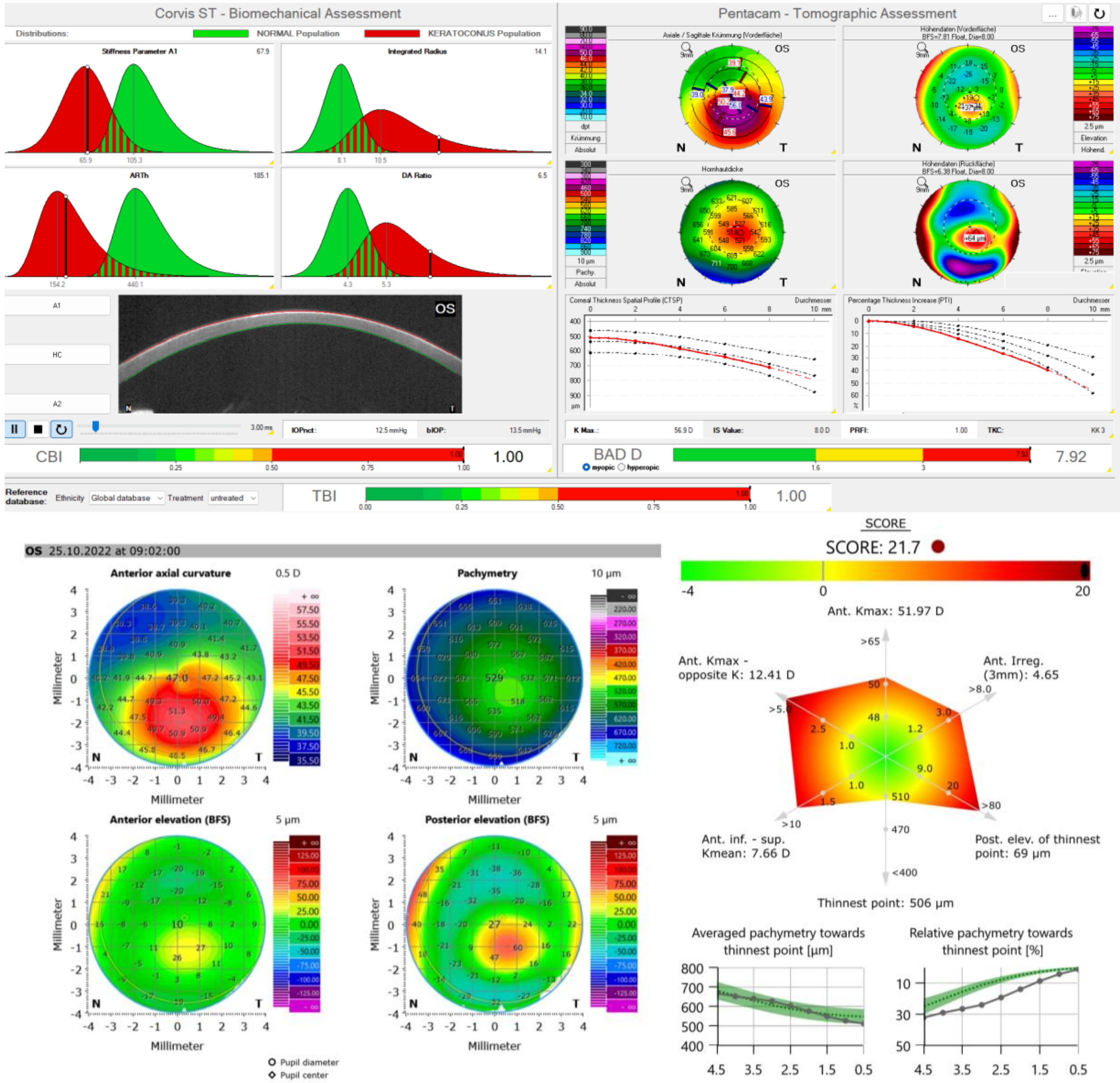
Corvis ST - Biomechanical Assessment
Pentacam - Tomographic Assessment
OS
CBI
1.00
BAD D
7.92
TBI
1.00
OS 25.10.2022 at 09:02:00
Anterior axial curvature
Pachymetry
Anterior elevation (BFS)
Posterior elevation (BFS)
Millimeter
SCORE
SCORE: 21.7
Ant. Kmax: 51.97 D
Ant. Kmax - opposite K: 12.41 D
Ant. Irreg. (3mm): 4.65
Ant. inf. - sup. Kmean: 7.66 D
Post. elev. of thinnest point: 69 µm
Thinnest point: 506 µm
Averaged pachymetry towards thinnest point [µm]
Relative pachymetry towards thinnest point [%]
Pupil diameter
Pupil center

CASE #13

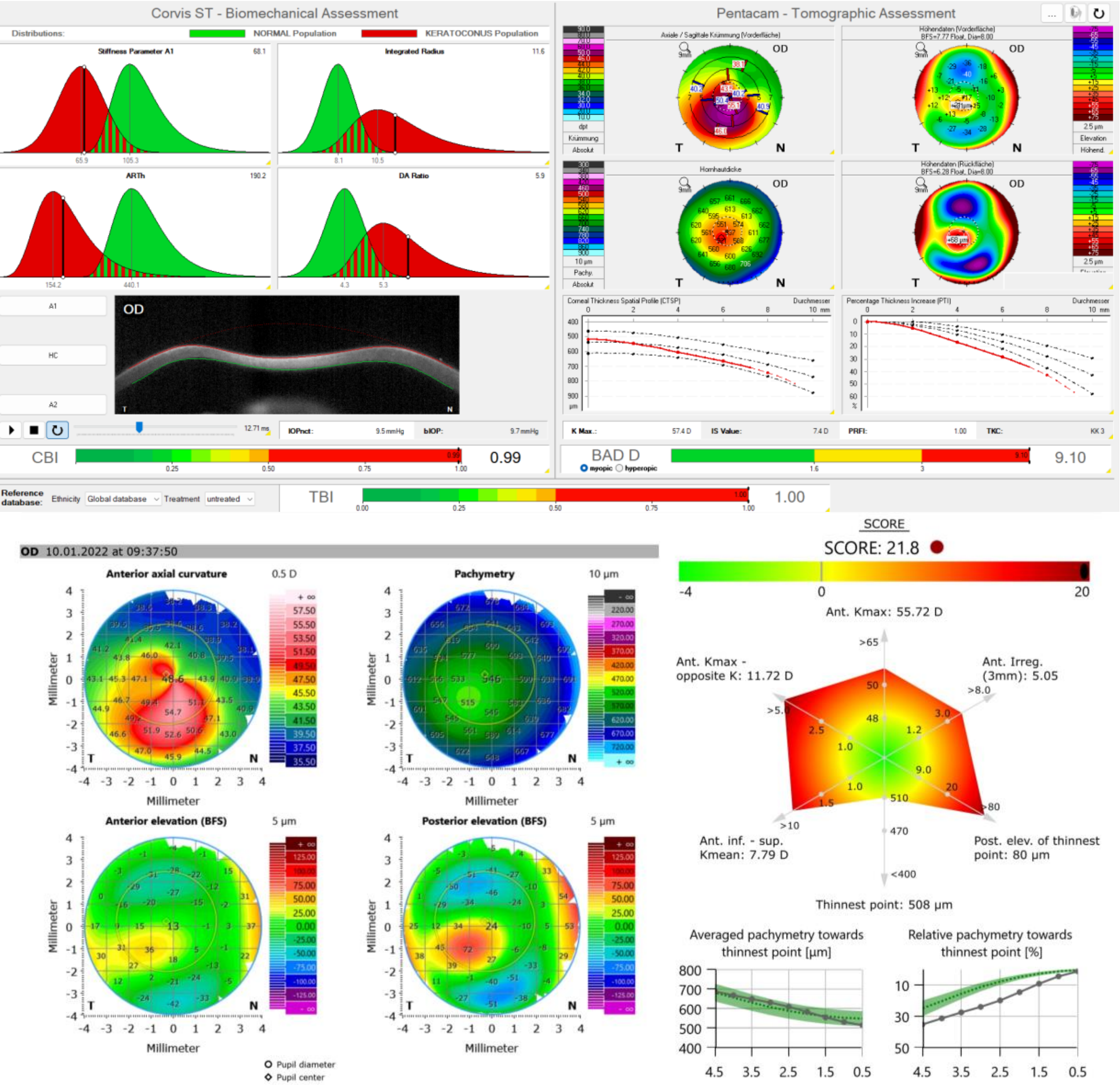

Corvis ST - Biomechanical Assessment
Pentacam - Tomographic Assessment
NORMAL Population
KERATOCONUS Population
OD
CBI 0.99
BAD D 9.10
TBI 1.00
OD 10.01.2022 at 09:37:50
Anterior axial curvature
Pachymetry
Anterior elevation (BFS)
Posterior elevation (BFS)
Millimeter
SCORE
SCORE: 21.8
Ant. Kmax: 55.72 D
Ant. Kmax - opposite K: 11.72 D
Ant. Irreg. (3mm): 5.05
Ant. inf. - sup. Kmean: 7.79 D
Post. elev. of thinnest point: 80 µm
Thinnest point: 508 µm
Averaged pachymetry towards thinnest point [µm]
Relative pachymetry towards thinnest point [%]
Pupil diameter
Pupil center

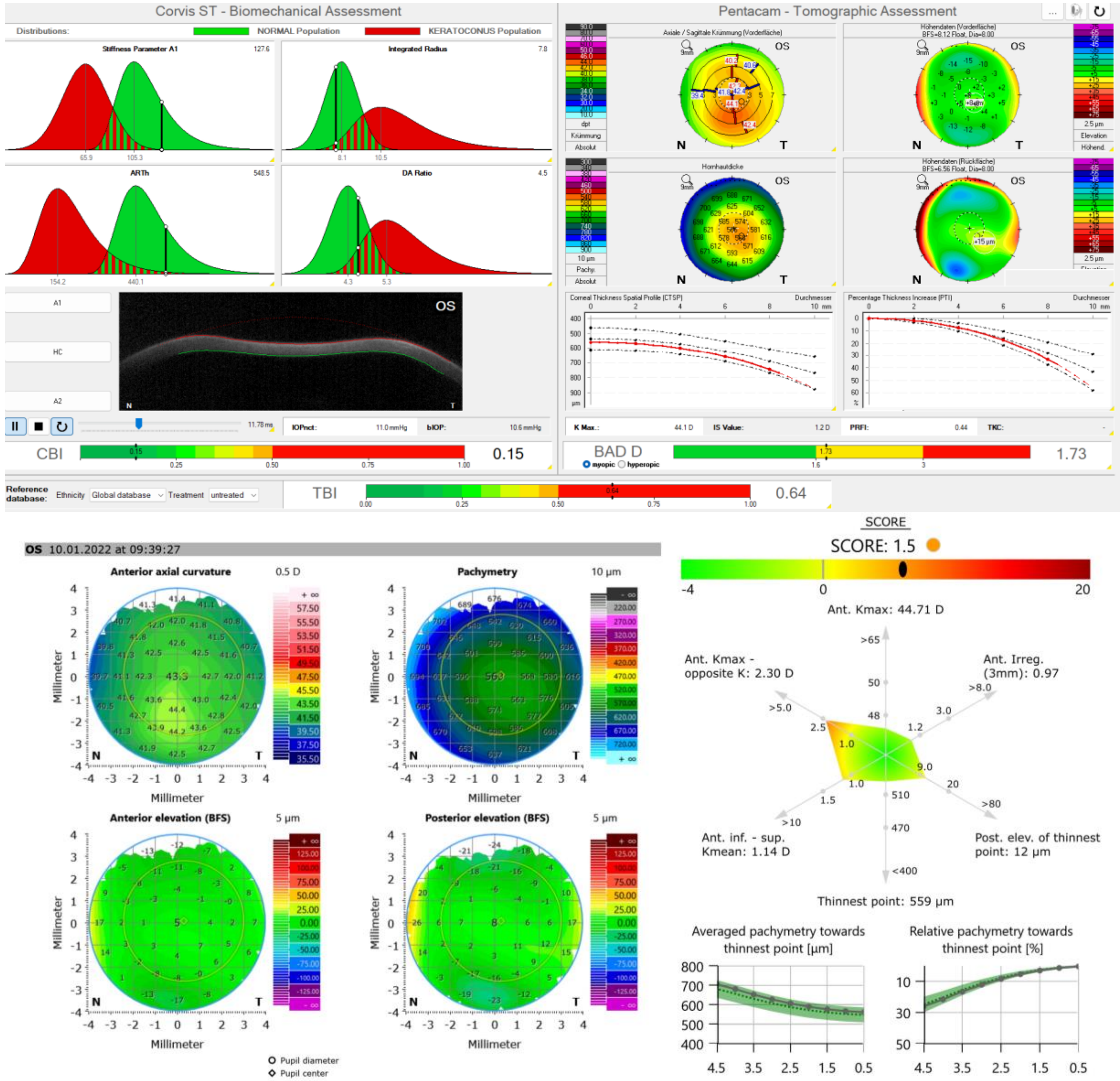

Corvis ST - Biomechanical Assessment
Pentacam - Tomographic Assessment
NORMAL Population
KERATOCONUS Population
OS
CBI 0.15
BAD D 1.73
TBI 0.64
OS 10.01.2022 at 09:39:27
Anterior axial curvature
Pachymetry
Anterior elevation (BFS)
Posterior elevation (BFS)
Millimeter
SCORE
SCORE: 1.5
Ant. Kmax: 44.71 D
Ant. Kmax - opposite K: 2.30 D
Ant. Irreg. (3mm): 0.97
Ant. inf. - sup. Kmean: 1.14 D
Post. elev. of thinnest point: 12 µm
Thinnest point: 559 µm
Averaged pachymetry towards thinnest point [µm]
Relative pachymetry towards thinnest point [%]
Pupil diameter
Pupil center

CASE #14

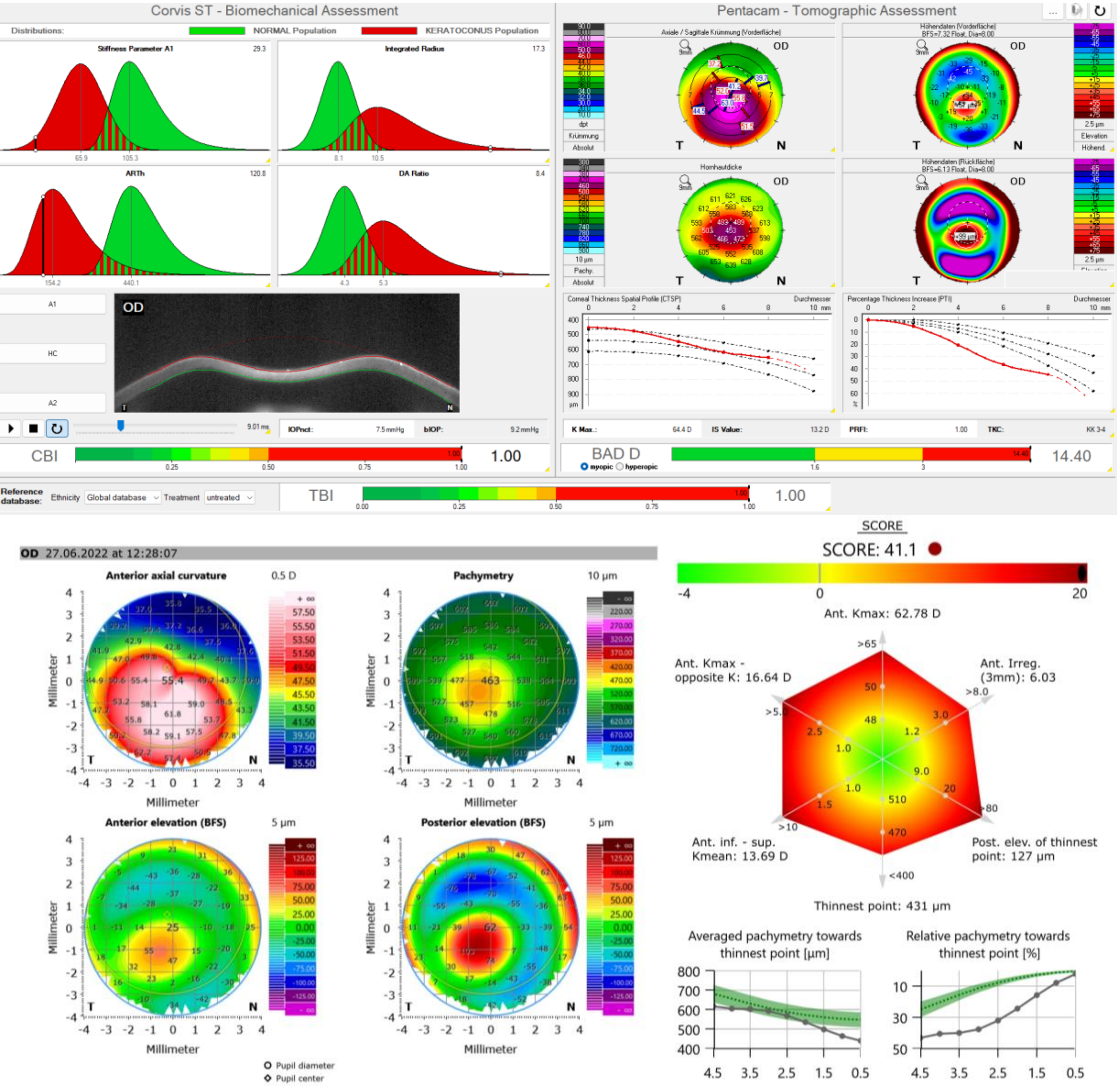

Corvis ST - Biomechanical Assessment
Pentacam - Tomographic Assessment
NORMAL Population
KERATOCONUS Population
OD
CBI 1.00
BAD D 14.40
TBI 1.00
OD 27.06.2022 at 12:28:07
Anterior axial curvature
Pachymetry
Anterior elevation (BFS)
Posterior elevation (BFS)
Millimeter
SCORE
SCORE: 41.1
Ant. Kmax: 62.78 D
Ant. Kmax - opposite K: 16.64 D
Ant. Irreg. (3mm): 6.03
Ant. inf. - sup. Kmean: 13.69 D
Post. elev. of thinnest point: 127 µm
Thinnest point: 431 µm
Averaged pachymetry towards thinnest point [µm]
Relative pachymetry towards thinnest point [%]
Pupil diameter
Pupil center

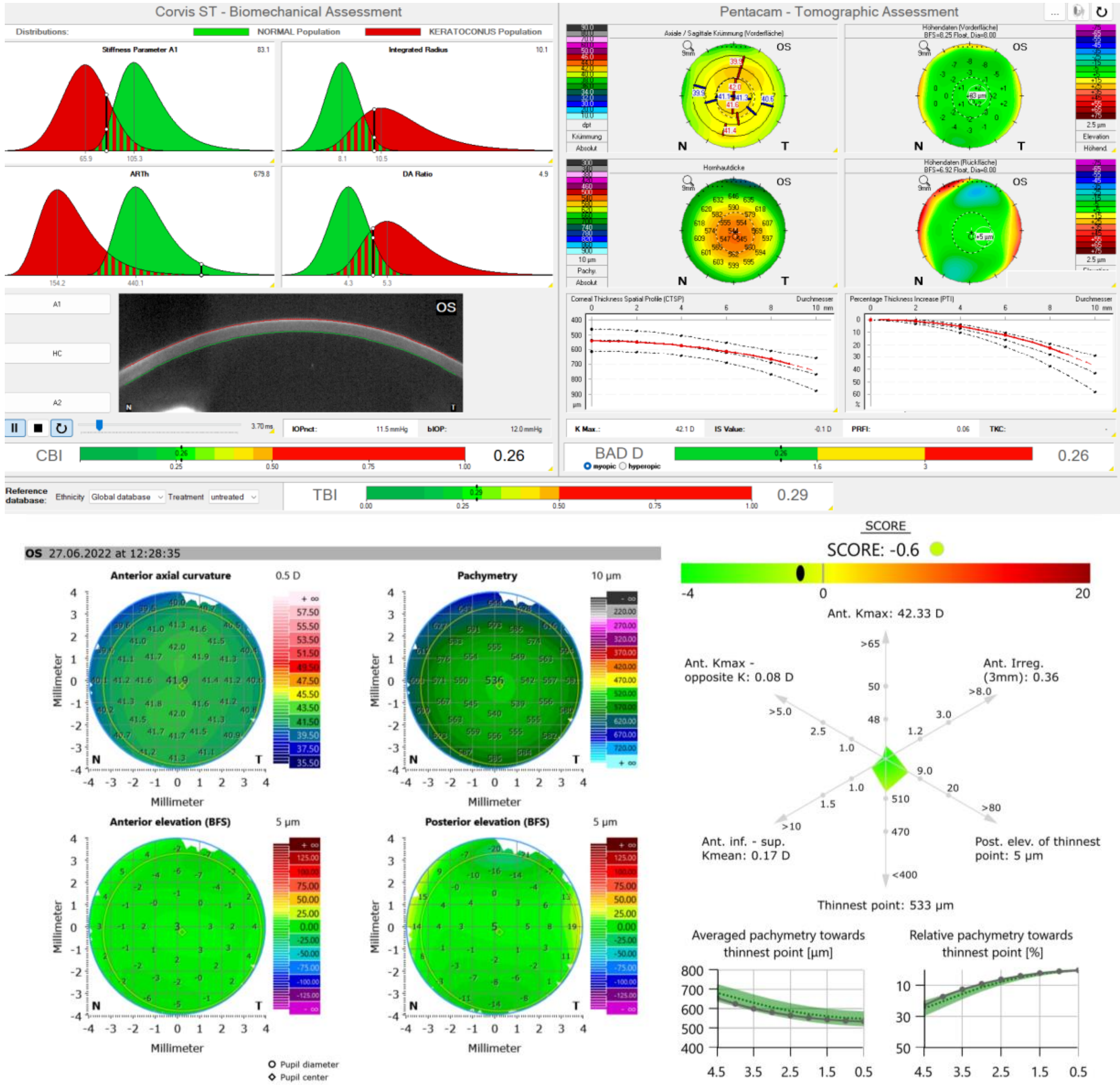

Corvis ST - Biomechanical Assessment
Pentacam - Tomographic Assessment
NORMAL Population
KERATOCONUS Population
OS
CBI 0.26
BAD D 0.26
TBI 0.29
OS 27.06.2022 at 12:28:35
Anterior axial curvature
Pachymetry
Anterior elevation (BFS)
Posterior elevation (BFS)
Millimeter
SCORE
SCORE: -0.6
Ant. Kmax: 42.33 D
Ant. Kmax - opposite K: 0.08 D
Ant. Irreg. (3mm): 0.36
Ant. inf. - sup. Kmean: 0.17 D
Post. elev. of thinnest point: 5 µm
Thinnest point: 533 µm
Averaged pachymetry towards thinnest point [µm]
Relative pachymetry towards thinnest point [%]
Pupil diameter
Pupil center

CASE #15

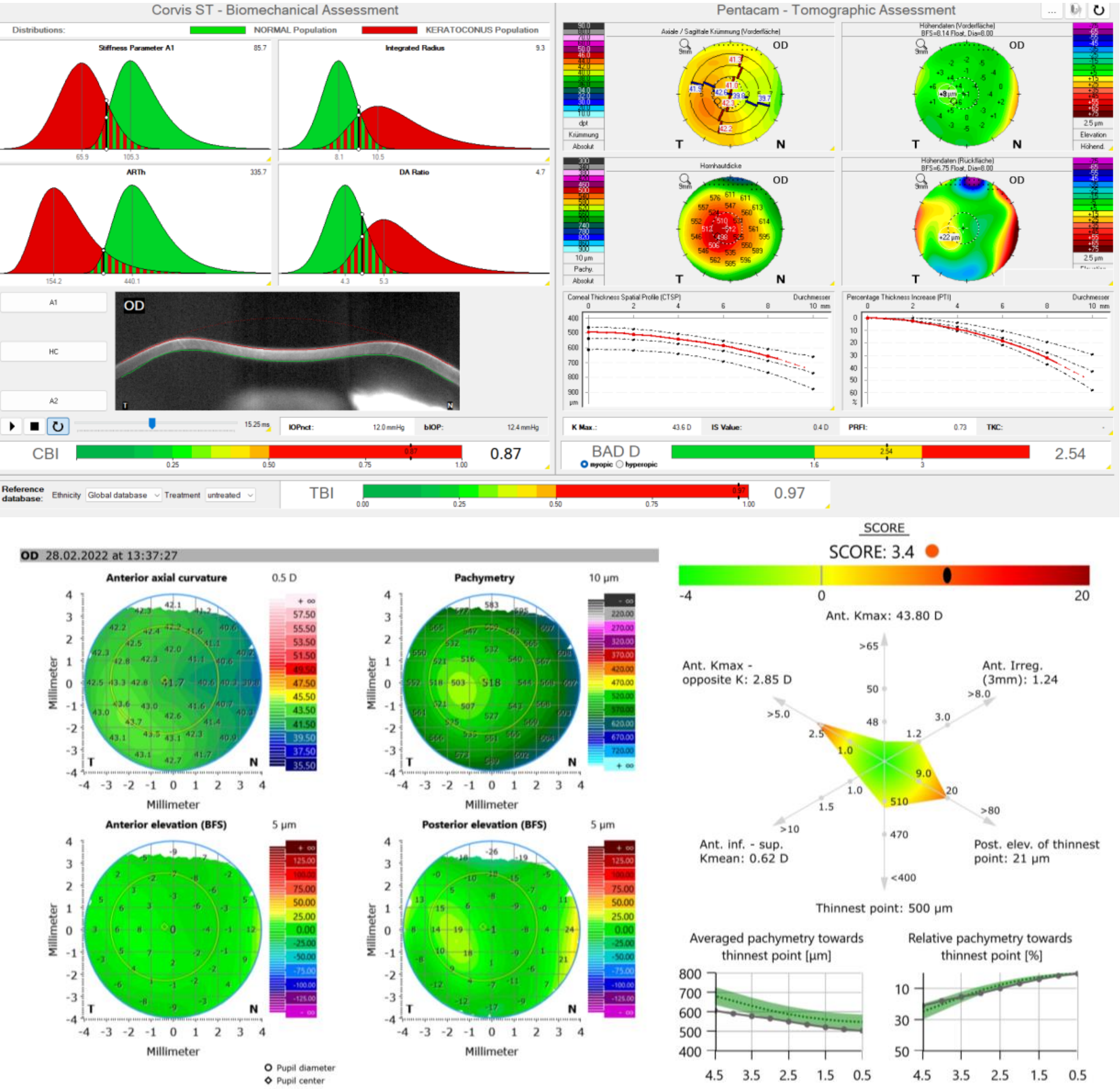
Corvis ST - Biomechanical Assessment
Pentacam - Tomographic Assessment
NORMAL Population
KERATOCONUS Population
Stiffness Parameter A1
Integrated Radius
ARTh
DA Ratio
OD
CBI
0.87
BAD D
2.54
TBI
0.97
SCORE
OD 28.02.2022 at 13:37:27
Anterior axial curvature
Pachymetry
Anterior elevation (BFS)
Posterior elevation (BFS)
Millimeter
SCORE: 3.4
Ant. Kmax: 43.80 D
Ant. Kmax - opposite K: 2.85 D
Ant. Irreg. (3mm): 1.24
Ant. inf. - sup. Kmean: 0.62 D
Post. elev. of thinnest point: 21 µm
Thinnest point: 500 µm
Averaged pachymetry towards thinnest point [µm]
Relative pachymetry towards thinnest point [%]
Pupil diameter
Pupil center

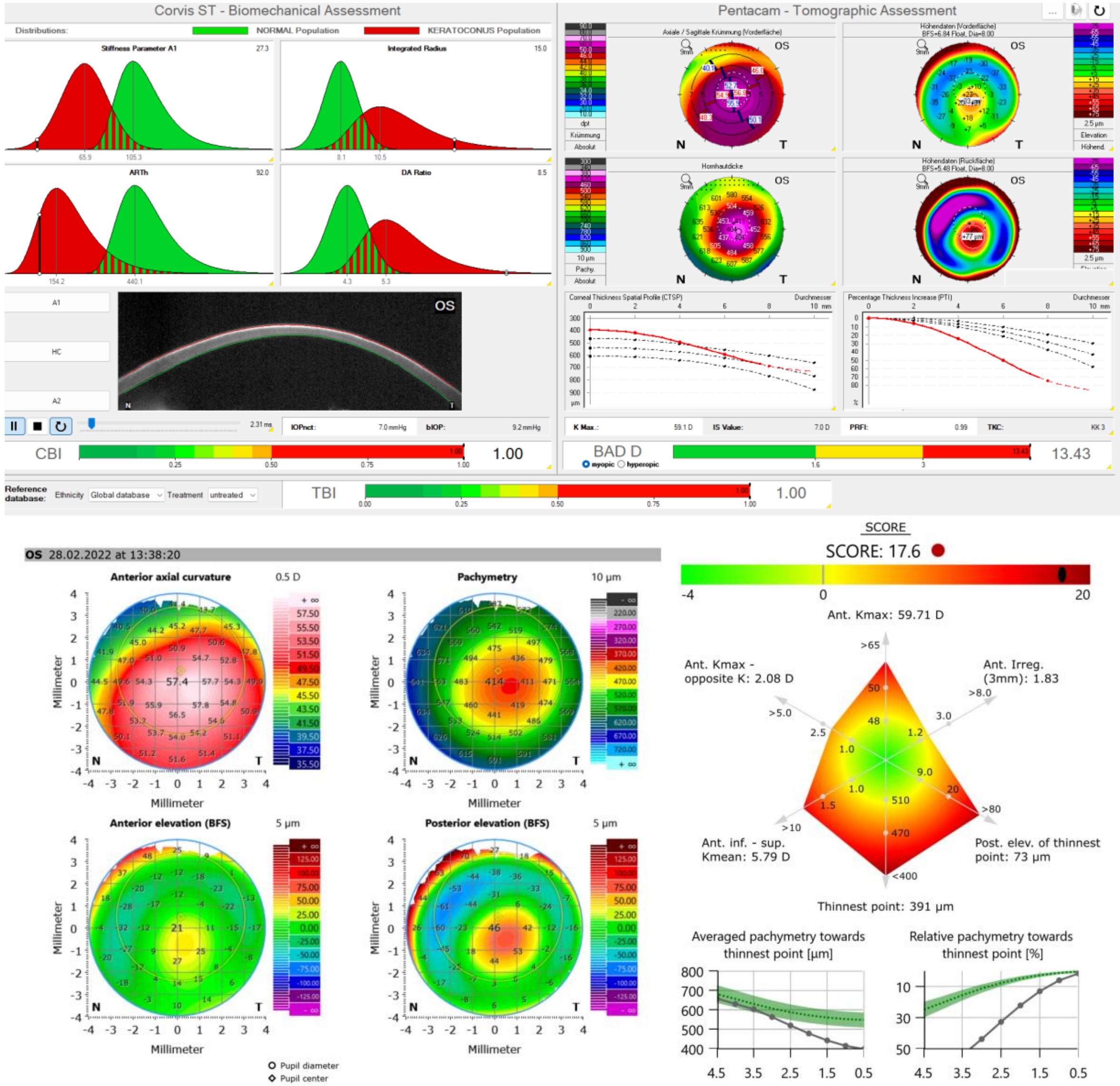
Corvis ST - Biomechanical Assessment
Pentacam - Tomographic Assessment
NORMAL Population
KERATOCONUS Population
Stiffness Parameter A1
Integrated Radius
ARTh
DA Ratio
OS
CBI
1.00
BAD D
13.43
TBI
1.00
SCORE
OS 28.02.2022 at 13:38:20
Anterior axial curvature
Pachymetry
Anterior elevation (BFS)
Posterior elevation (BFS)
Millimeter
SCORE: 17.6
Ant. Kmax: 59.71 D
Ant. Kmax - opposite K: 2.08 D
Ant. Irreg. (3mm): 1.83
Ant. inf. - sup. Kmean: 5.79 D
Post. elev. of thinnest point: 73 µm
Thinnest point: 391 µm
Averaged pachymetry towards thinnest point [µm]
Relative pachymetry towards thinnest point [%]
Pupil diameter
Pupil center

CASE #16

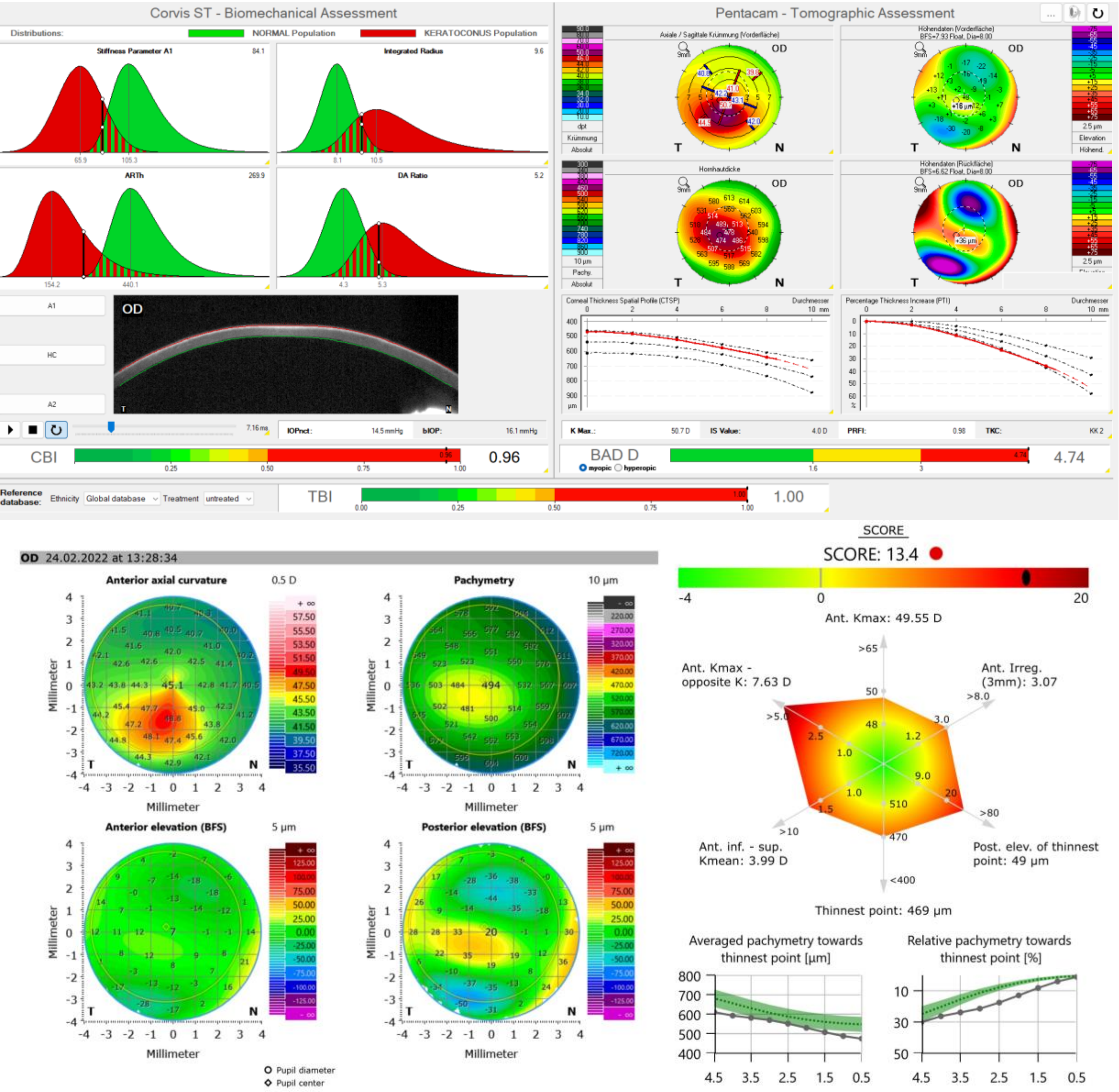
Corvis ST - Biomechanical Assessment
Pentacam - Tomographic Assessment
NORMAL Population
KERATOCONUS Population
Stiffness Parameter A1
Integrated Radius
ARTh
DA Ratio
OD
CBI 0.96
BAD D 4.74
TBI 1.00
OD 24.02.2022 at 13:28:34
Anterior axial curvature
Pachymetry
Anterior elevation (BFS)
Posterior elevation (BFS)
Millimeter
SCORE
SCORE: 13.4
Ant. Kmax: 49.55 D
Ant. Kmax - opposite K: 7.63 D
Ant. Irreg. (3mm): 3.07
Ant. inf. - sup. Kmean: 3.99 D
Post. elev. of thinnest point: 49 µm
Thinnest point: 469 µm
Averaged pachymetry towards thinnest point [µm]
Relative pachymetry towards thinnest point [%]
Pupil diameter
Pupil center

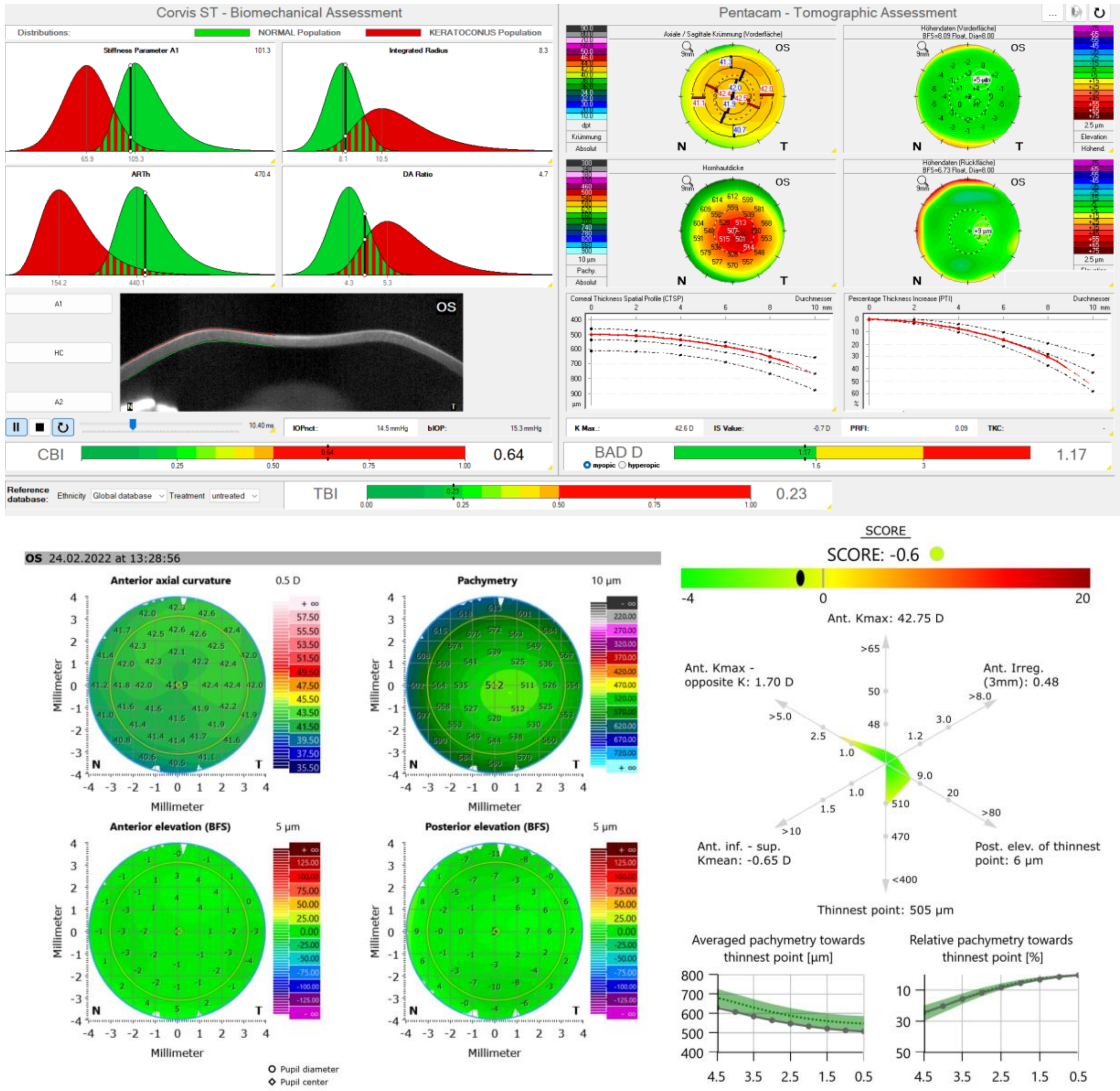
Corvis ST - Biomechanical Assessment
Pentacam - Tomographic Assessment
NORMAL Population
KERATOCONUS Population
Stiffness Parameter A1
Integrated Radius
ARTh
DA Ratio
OS
CBI 0.64
BAD D 1.17
TBI 0.23
OS 24.02.2022 at 13:28:56
Anterior axial curvature
Pachymetry
Anterior elevation (BFS)
Posterior elevation (BFS)
Millimeter
SCORE
SCORE: -0.6
Ant. Kmax: 42.75 D
Ant. Kmax - opposite K: 1.70 D
Ant. Irreg. (3mm): 0.48
Ant. inf. - sup. Kmean: -0.65 D
Post. elev. of thinnest point: 6 µm
Thinnest point: 505 µm
Averaged pachymetry towards thinnest point [µm]
Relative pachymetry towards thinnest point [%]
Pupil diameter
Pupil center

CASE #17

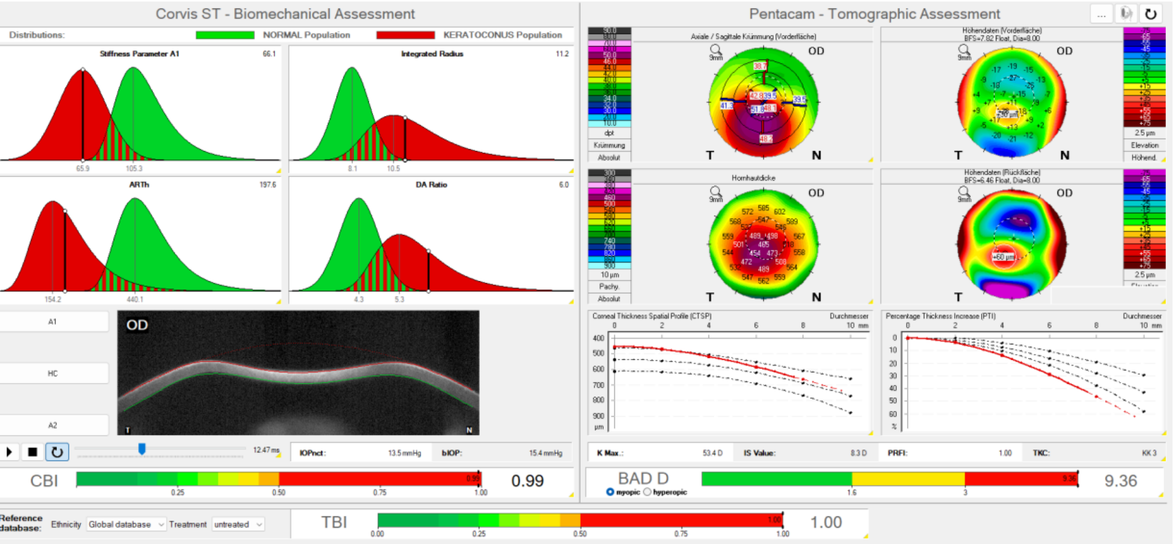

Corvis ST - Biomechanical Assessment
Pentacam - Tomographic Assessment
NORMAL Population
KERATOCONUS Population
Stiffness Parameter A1
Integrated Radius
ARTh
DA Ratio
OD
CBI
0.99
BAD D
9.36
TBI
1.00

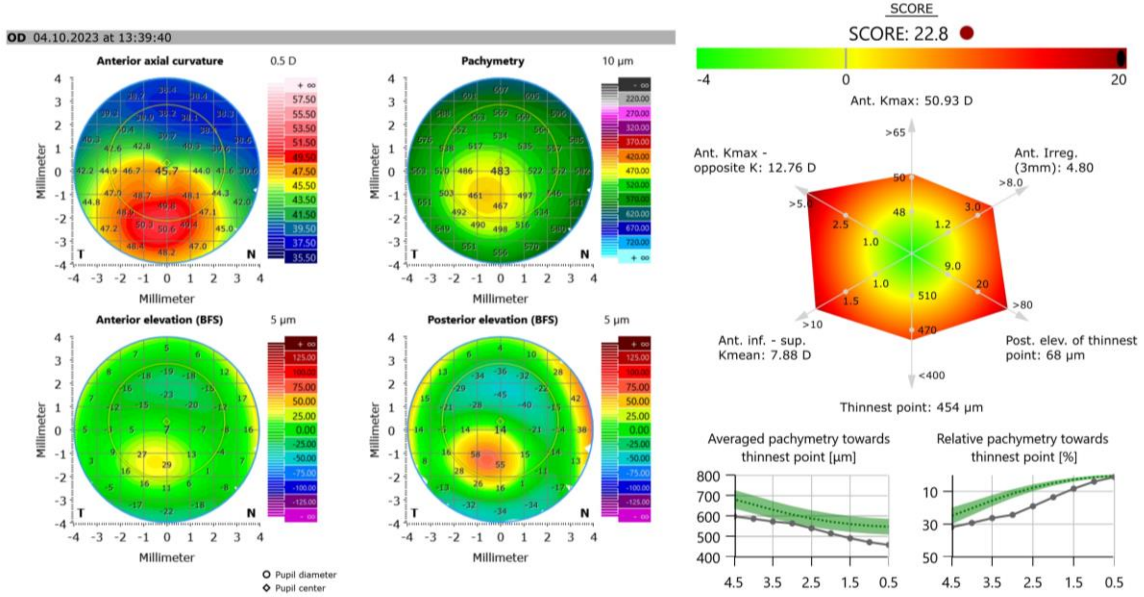

OD 04.10.2023 at 13:39:40
Anterior axial curvature
Pachymetry
Anterior elevation (BFS)
Posterior elevation (BFS)
Millimeter
SCORE
SCORE: 22.8
Ant. Kmax: 50.93 D
Ant. Kmax - opposite K: 12.76 D
Ant. Irreg. (3mm): 4.80
Ant. inf. - sup. Kmean: 7.88 D
Post. elev. of thinnest point: 68 µm
Thinnest point: 454 µm
Averaged pachymetry towards thinnest point [µm]
Relative pachymetry towards thinnest point [%]
Pupil diameter
Pupil center

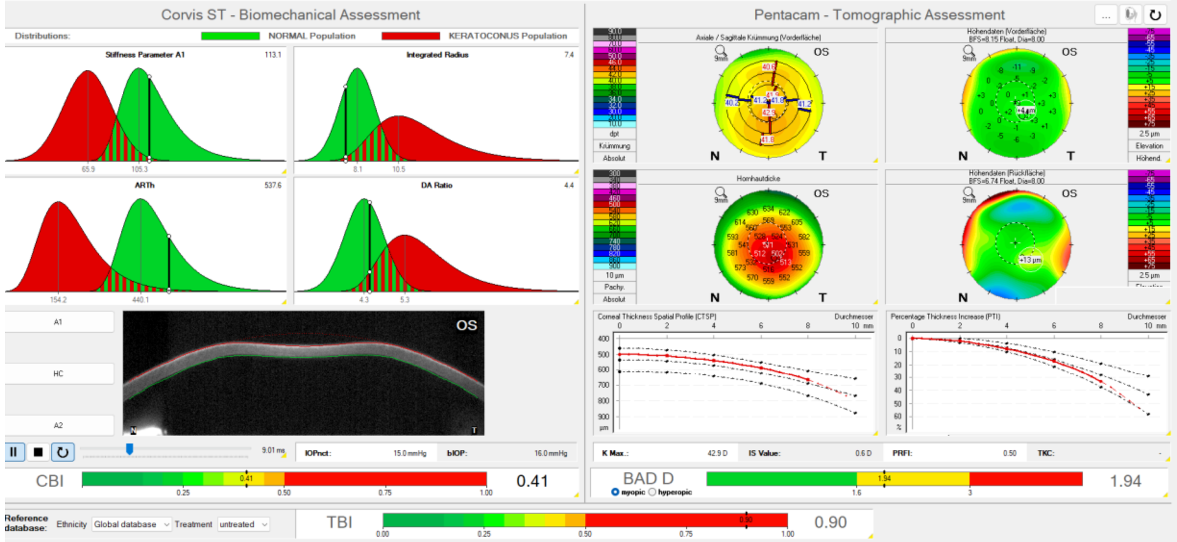

Corvis ST - Biomechanical Assessment
Pentacam - Tomographic Assessment
NORMAL Population
KERATOCONUS Population
Stiffness Parameter A1
Integrated Radius
ARTh
DA Ratio
OS
CBI
0.41
BAD D
1.94
TBI
0.90

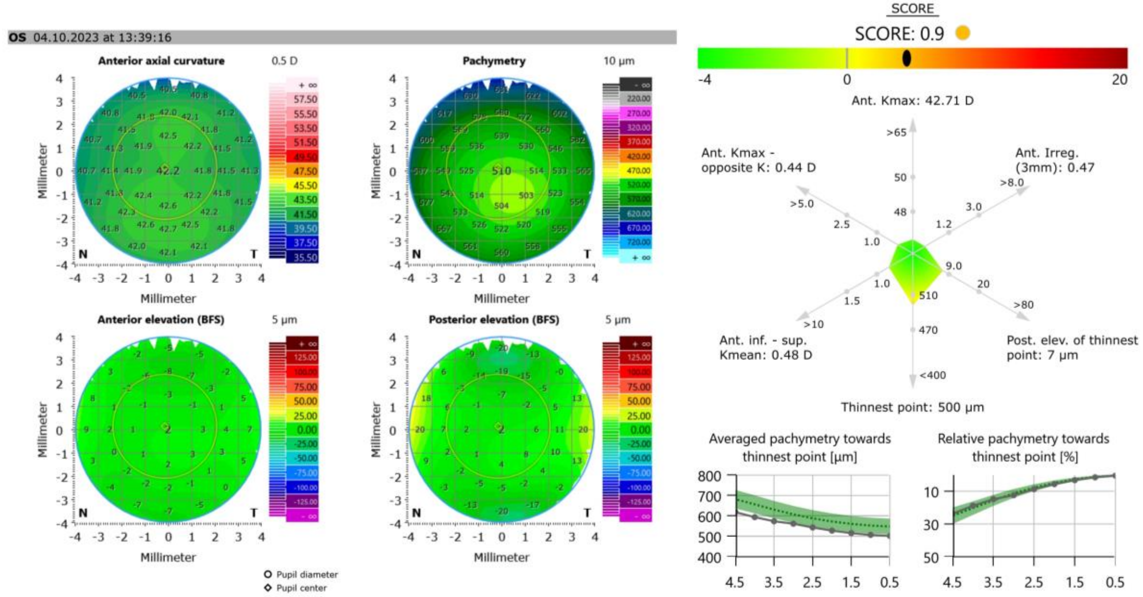

OS 04.10.2023 at 13:39:16
Anterior axial curvature
Pachymetry
Anterior elevation (BFS)
Posterior elevation (BFS)
Millimeter
SCORE
SCORE: 0.9
Ant. Kmax: 42.71 D
Ant. Kmax - opposite K: 0.44 D
Ant. Irreg. (3mm): 0.47
Ant. inf. - sup. Kmean: 0.48 D
Post. elev. of thinnest point: 7 µm
Thinnest point: 500 µm
Averaged pachymetry towards thinnest point [µm]
Relative pachymetry towards thinnest point [%]
Pupil diameter
Pupil center

CASE #18

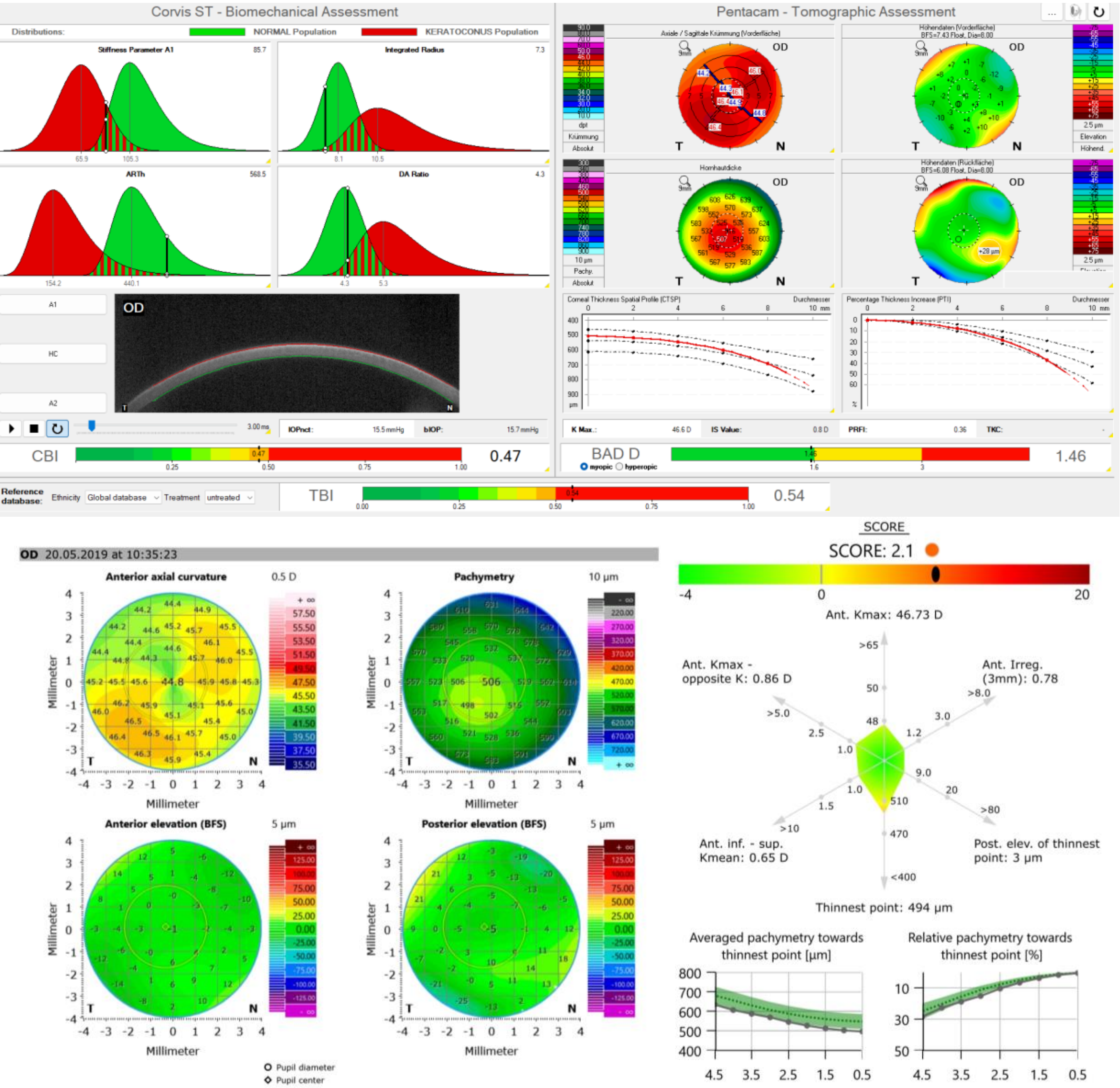

Corvis ST - Biomechanical Assessment
Pentacam - Tomographic Assessment
NORMAL Population
KERATOCONUS Population
Stiffness Parameter A1
Integrated Radius
ARTh
DA Ratio
OD
CBI 0.47
BAD D 1.46
TBI 0.54
Reference database:
OD 20.05.2019 at 10:35:23
Anterior axial curvature
Pachymetry
Anterior elevation (BFS)
Posterior elevation (BFS)
Millimeter
SCORE
SCORE: 2.1
Ant. Kmax: 46.73 D
Ant. Kmax - opposite K: 0.86 D
Ant. Irreg. (3mm): 0.78
Ant. inf. - sup. Kmean: 0.65 D
Post. elev. of thinnest point: 3 µm
Thinnest point: 494 µm
Averaged pachymetry towards thinnest point [µm]
Relative pachymetry towards thinnest point [%]
Pupil diameter
Pupil center

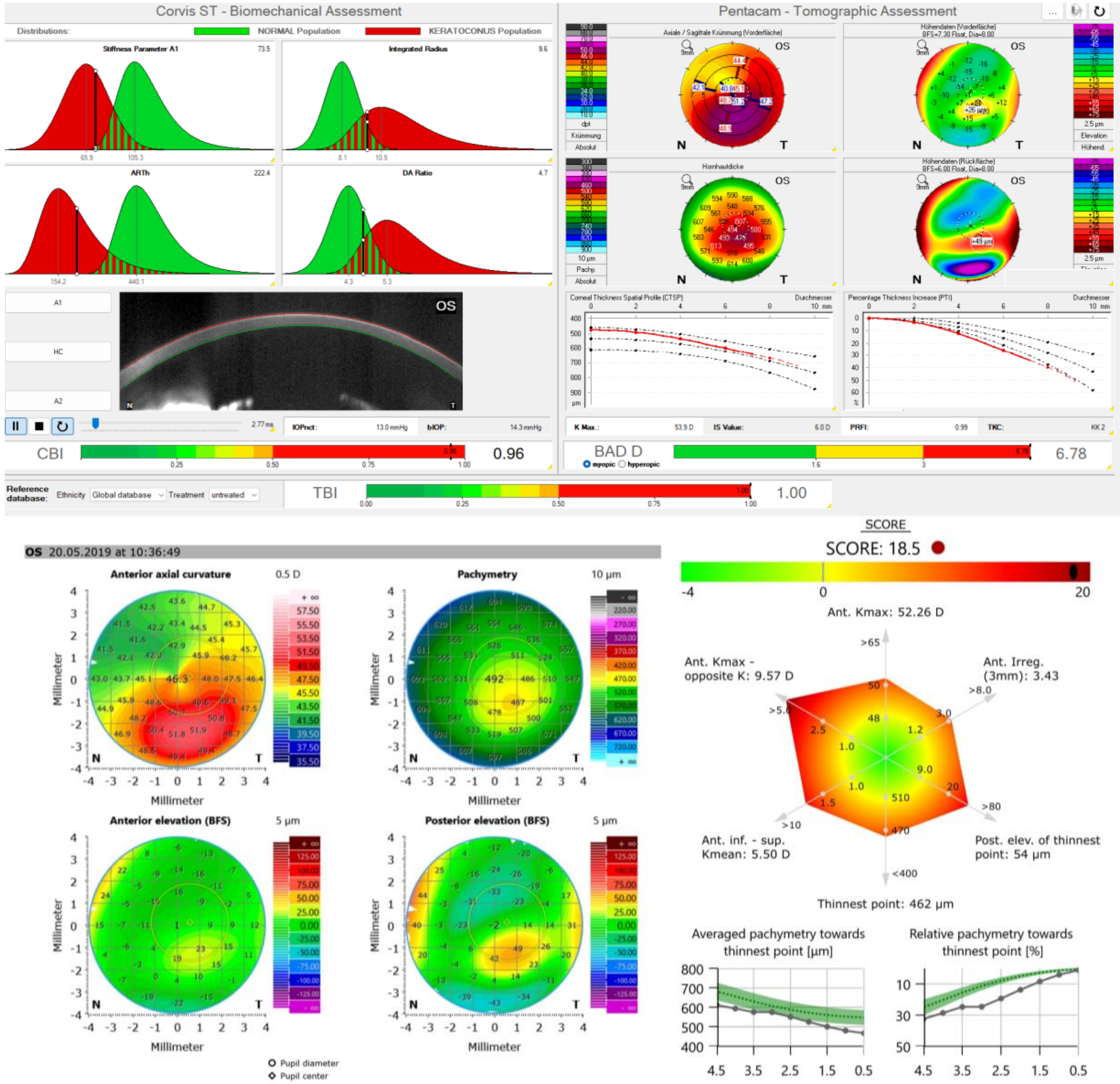

Corvis ST - Biomechanical Assessment
Pentacam - Tomographic Assessment
NORMAL Population
KERATOCONUS Population
Stiffness Parameter A1
Integrated Radius
ARTh
DA Ratio
OS
CBI 0.96
BAD D 6.78
TBI 1.00
Reference database:
OS 20.05.2019 at 10:36:49
Anterior axial curvature
Pachymetry
Anterior elevation (BFS)
Posterior elevation (BFS)
Millimeter
SCORE
SCORE: 18.5
Ant. Kmax: 52.26 D
Ant. Kmax - opposite K: 9.57 D
Ant. Irreg. (3mm): 3.43
Ant. inf. - sup. Kmean: 5.50 D
Post. elev. of thinnest point: 54 µm
Thinnest point: 462 µm
Averaged pachymetry towards thinnest point [µm]
Relative pachymetry towards thinnest point [%]
Pupil diameter
Pupil center

CASE #19

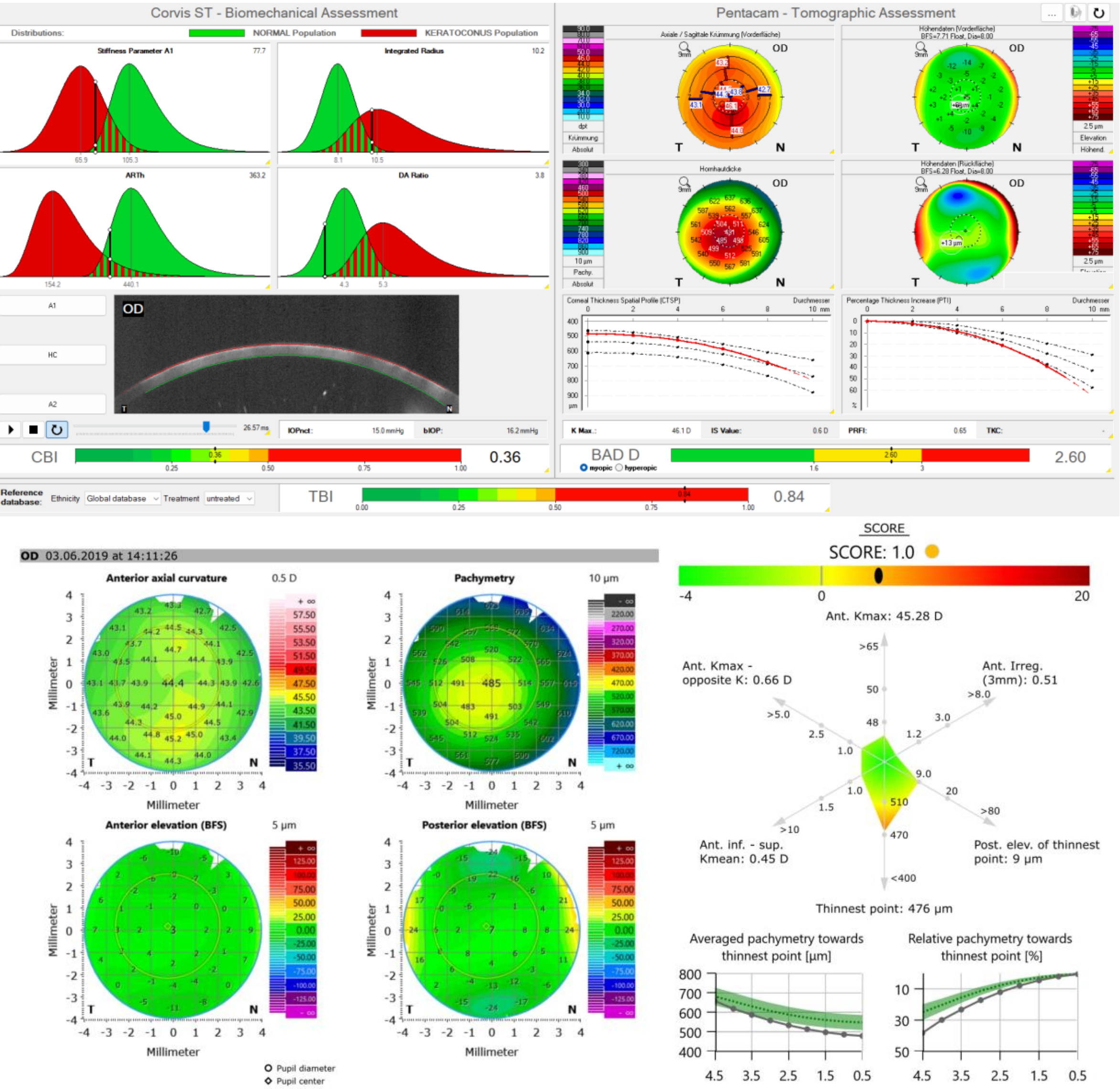

Excluded for analysis due to prior CXL

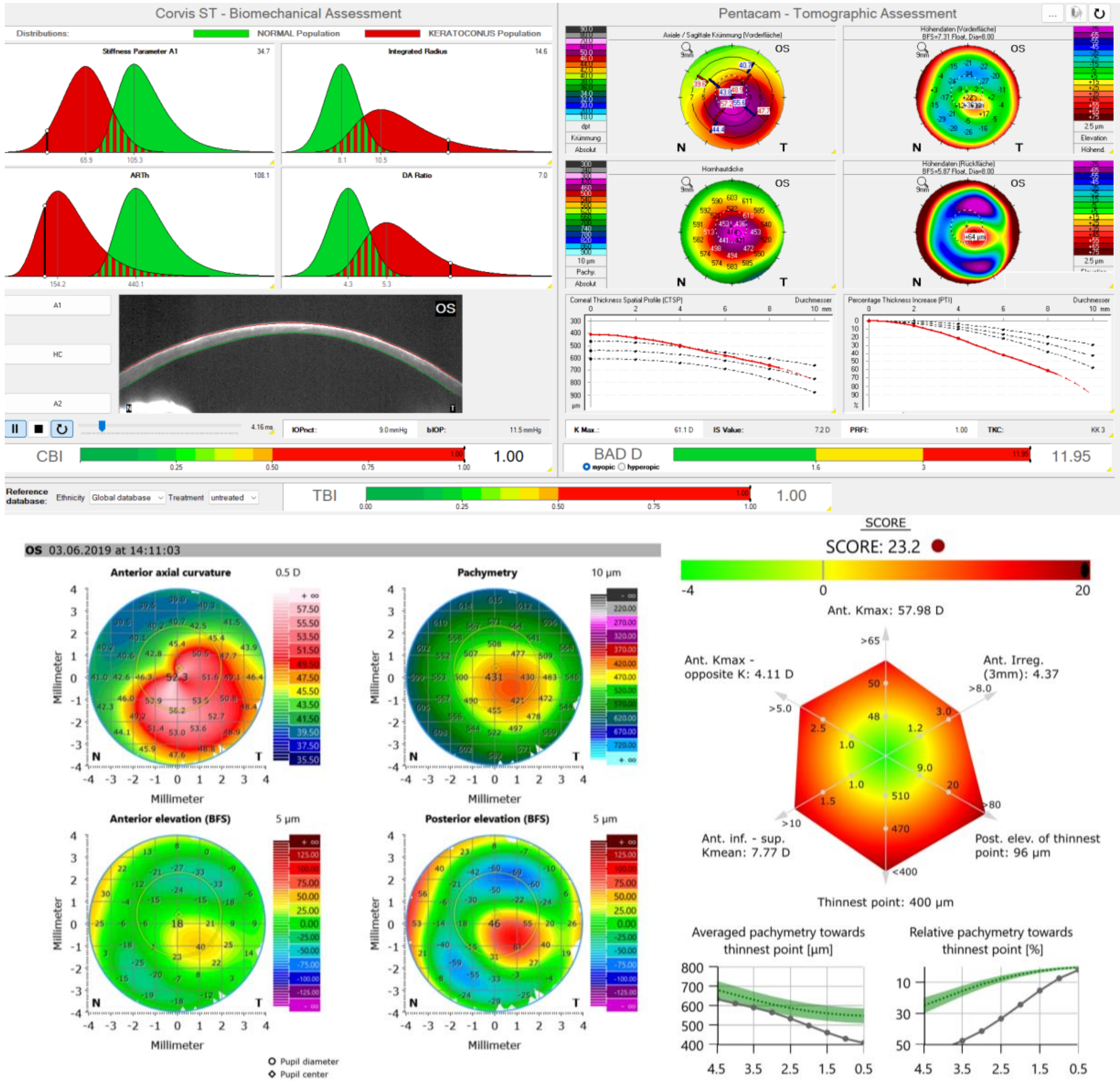

CASE #20

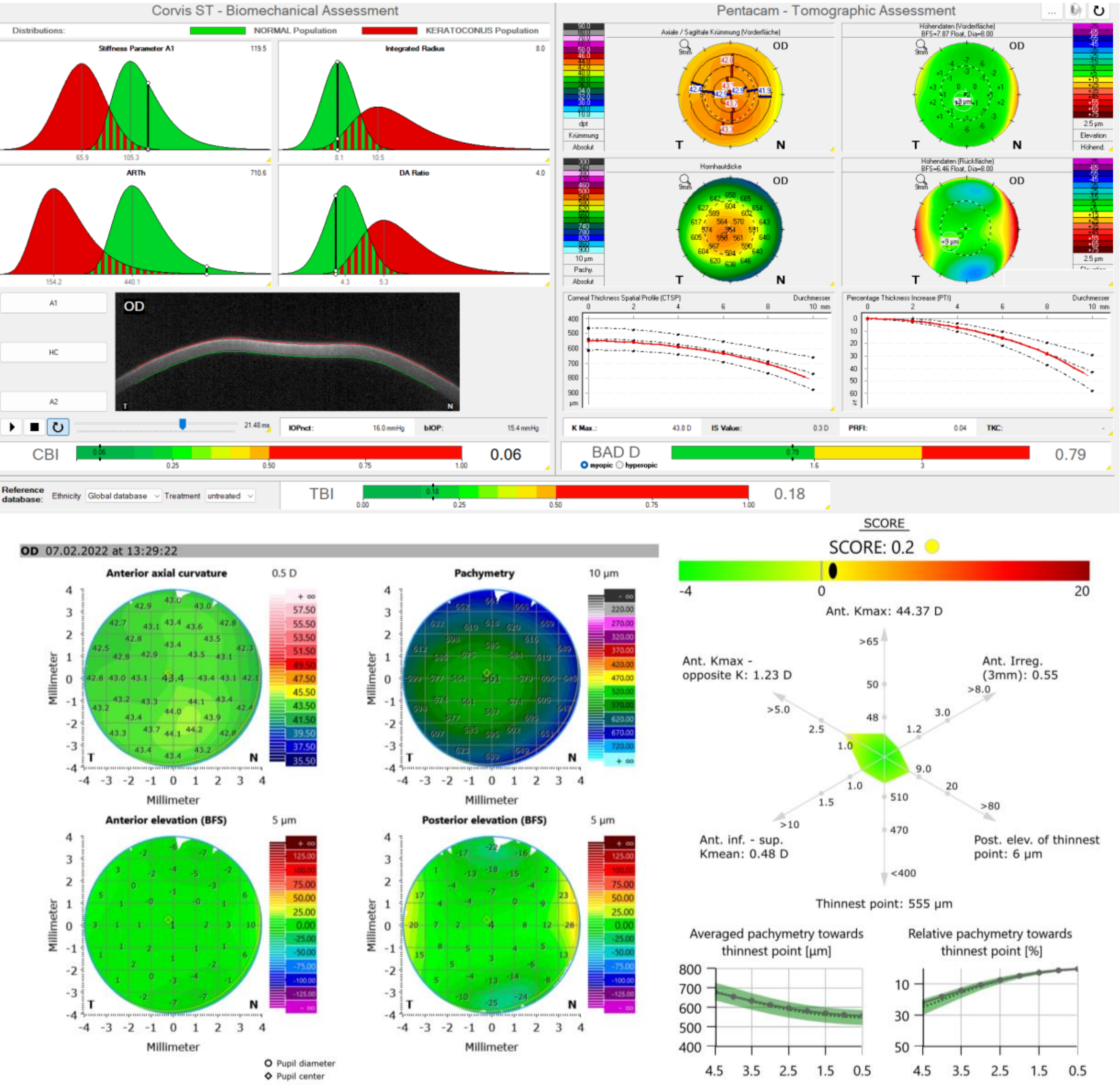
Corvis ST - Biomechanical Assessment
Pentacam - Tomographic Assessment
Distributions: NORMAL Population KERATOCONUS Population
Stiffness Parameter A1
Integrated Radius
ARTh
DA Ratio
OD
CBI 0.06
BAD D 0.79
TBI 0.18
Reference database: Ethnicity Global database Treatment untreated
OD 07.02.2022 at 13:29:22
Anterior axial curvature
Pachymetry
Anterior elevation (BFS)
Posterior elevation (BFS)
Millimeter
Pupil diameter
Pupil center
SCORE
SCORE: 0.2
Ant. Kmax: 44.37 D
Ant. Kmax - opposite K: 1.23 D
Ant. Irreg. (3mm): 0.55
Ant. inf. - sup. Kmean: 0.48 D
Post. elev. of thinnest point: 6 µm
Thinnest point: 555 µm
Averaged pachymetry towards thinnest point [µm]
Relative pachymetry towards thinnest point [%]

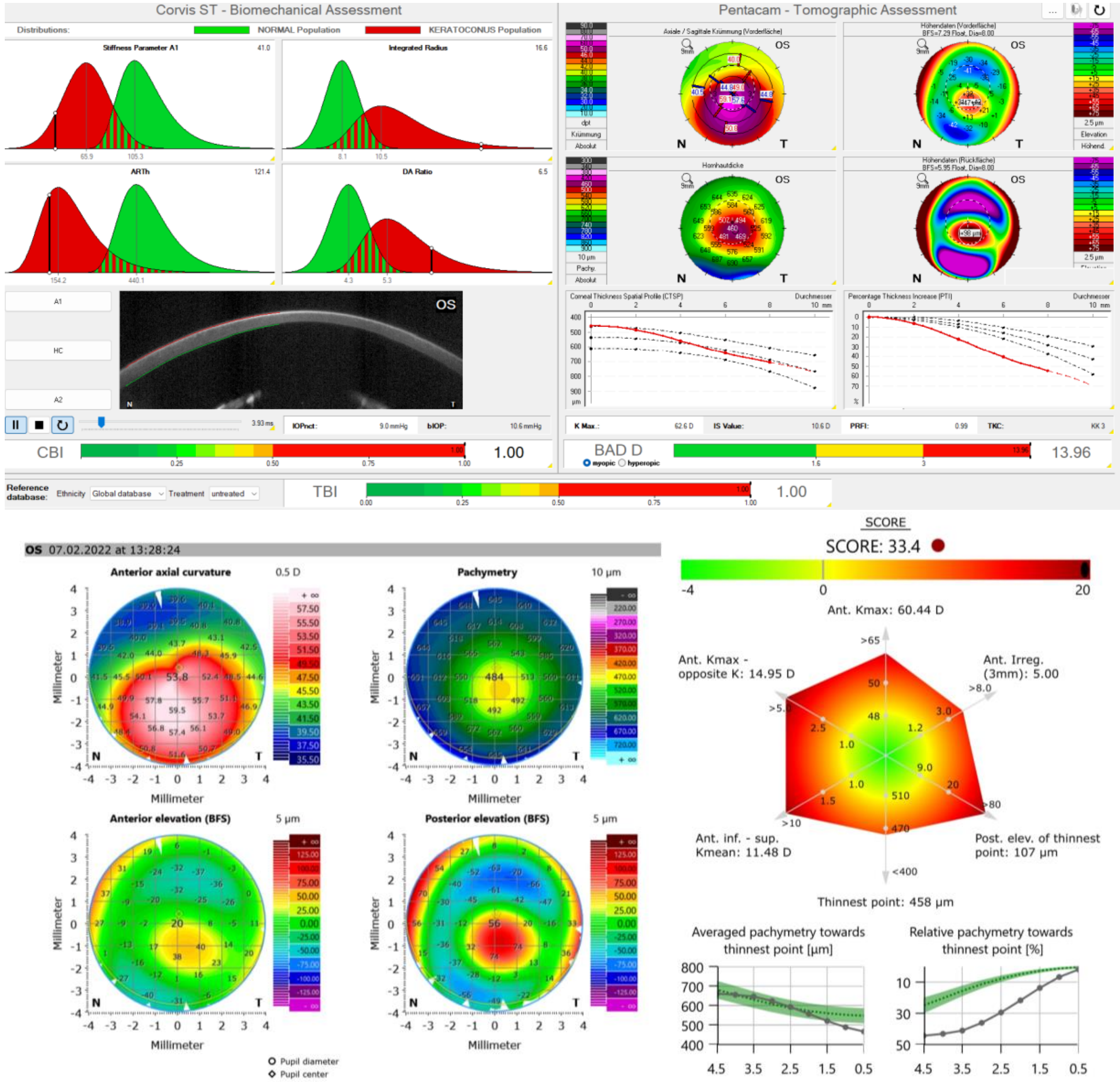
Corvis ST - Biomechanical Assessment
Pentacam - Tomographic Assessment
Distributions: NORMAL Population KERATOCONUS Population
Stiffness Parameter A1
Integrated Radius
ARTh
DA Ratio
OS
CBI 1.00
BAD D 13.96
TBI 1.00
Reference database: Ethnicity Global database Treatment untreated
OS 07.02.2022 at 13:28:24
Anterior axial curvature
Pachymetry
Anterior elevation (BFS)
Posterior elevation (BFS)
Millimeter
Pupil diameter
Pupil center
SCORE
SCORE: 33.4
Ant. Kmax: 60.44 D
Ant. Kmax - opposite K: 14.95 D
Ant. Irreg. (3mm): 5.00
Ant. inf. - sup. Kmean: 11.48 D
Post. elev. of thinnest point: 107 µm
Thinnest point: 458 µm
Averaged pachymetry towards thinnest point [µm]
Relative pachymetry towards thinnest point [%]

CASE #21

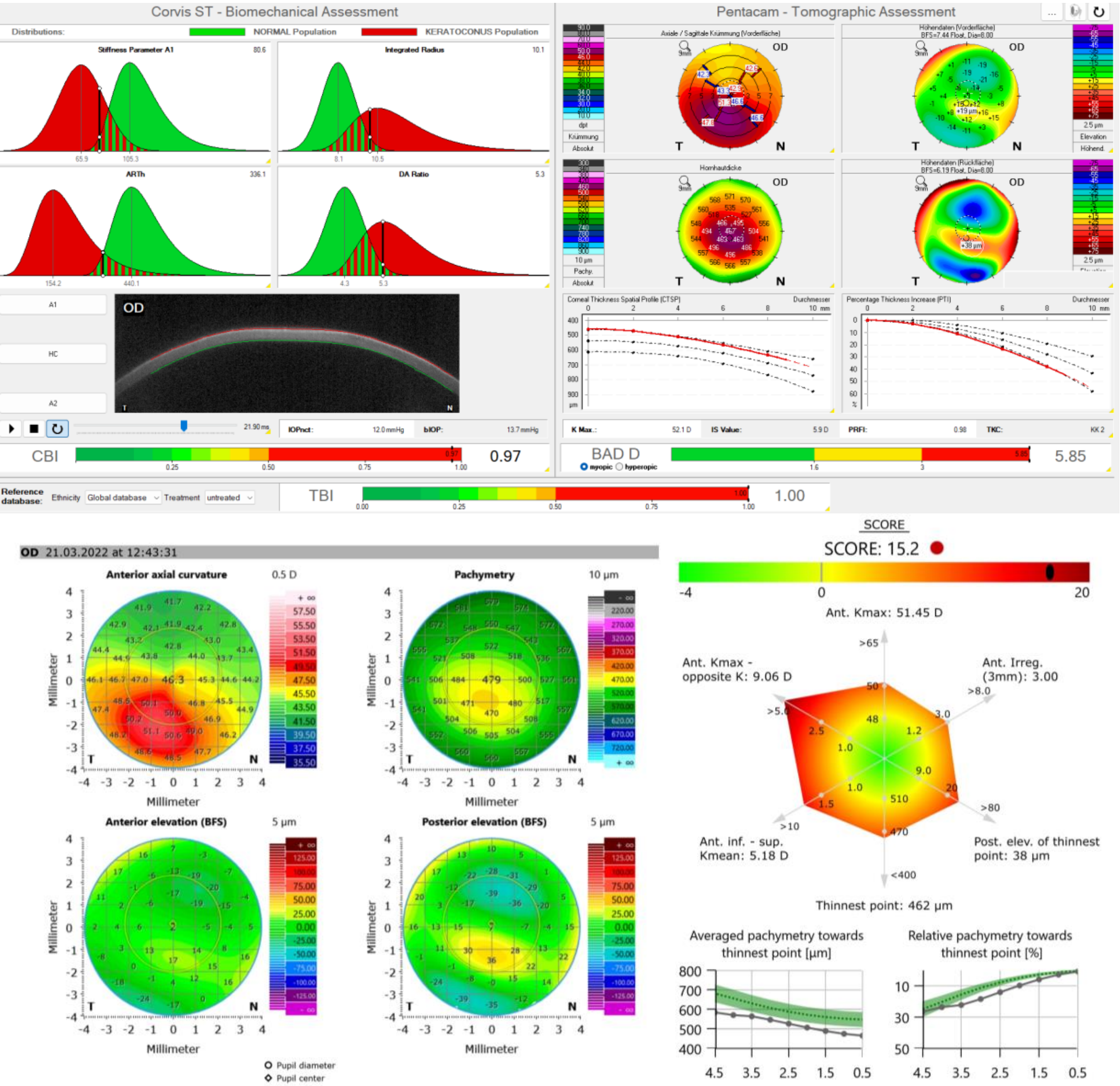

Corvis ST - Biomechanical Assessment
Pentacam - Tomographic Assessment
NORMAL Population
KERATOCONUS Population
Stiffness Parameter A1
Integrated Radius
ARTh
DA Ratio
OD
CBI
0.97
BAD D
5.85
TBI
1.00
OD 21.03.2022 at 12:43:31
Anterior axial curvature
Pachymetry
Anterior elevation (BFS)
Posterior elevation (BFS)
Millimeter
SCORE
SCORE: 15.2
Ant. Kmax: 51.45 D
Ant. Kmax - opposite K: 9.06 D
Ant. Irreg. (3mm): 3.00
Ant. inf. - sup. Kmean: 5.18 D
Post. elev. of thinnest point: 38 µm
Thinnest point: 462 µm
Averaged pachymetry towards thinnest point [µm]
Relative pachymetry towards thinnest point [%]
Pupil diameter
Pupil center

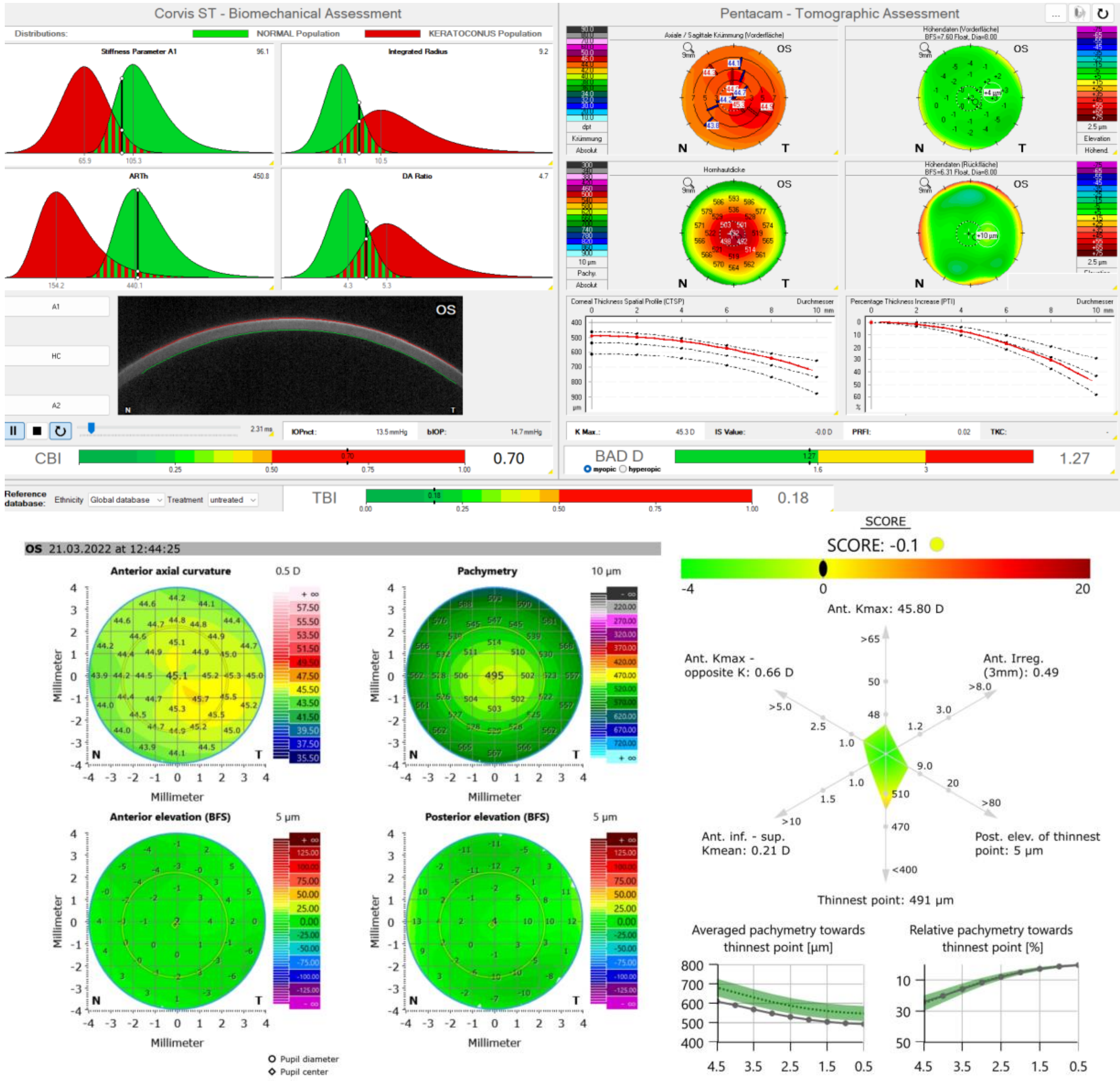

Corvis ST - Biomechanical Assessment
Pentacam - Tomographic Assessment
NORMAL Population
KERATOCONUS Population
Stiffness Parameter A1
Integrated Radius
ARTh
DA Ratio
OS
CBI
0.70
BAD D
1.27
TBI
0.18
OS 21.03.2022 at 12:44:25
Anterior axial curvature
Pachymetry
Anterior elevation (BFS)
Posterior elevation (BFS)
Millimeter
SCORE
SCORE: -0.1
Ant. Kmax: 45.80 D
Ant. Kmax - opposite K: 0.66 D
Ant. Irreg. (3mm): 0.49
Ant. inf. - sup. Kmean: 0.21 D
Post. elev. of thinnest point: 5 µm
Thinnest point: 491 µm
Averaged pachymetry towards thinnest point [µm]
Relative pachymetry towards thinnest point [%]
Pupil diameter
Pupil center

CASE #22

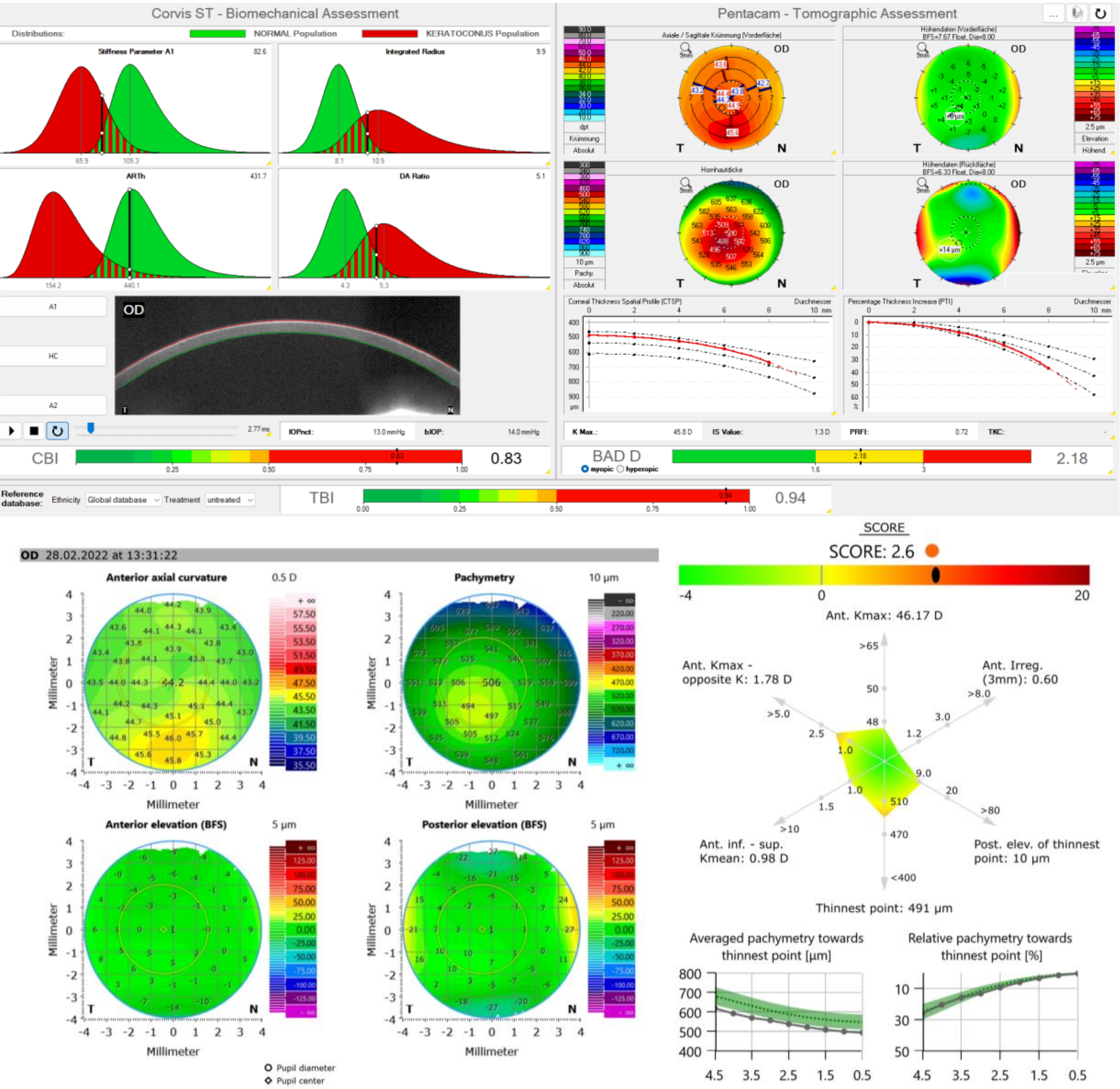
Corvis ST - Biomechanical Assessment
Pentacam - Tomographic Assessment
NORMAL Population
KERATOCONUS Population
OD
CBI
0.83
BAD D
2.18
TBI
0.94
OD 28.02.2022 at 13:31:22
Anterior axial curvature
Pachymetry
Anterior elevation (BFS)
Posterior elevation (BFS)
Millimeter
SCORE
SCORE: 2.6
Ant. Kmax: 46.17 D
Ant. Kmax - opposite K: 1.78 D
Ant. Irreg. (3mm): 0.60
Ant. inf. - sup. Kmean: 0.98 D
Post. elev. of thinnest point: 10 µm
Thinnest point: 491 µm
Averaged pachymetry towards thinnest point [µm]
Relative pachymetry towards thinnest point [%]
Pupil diameter
Pupil center

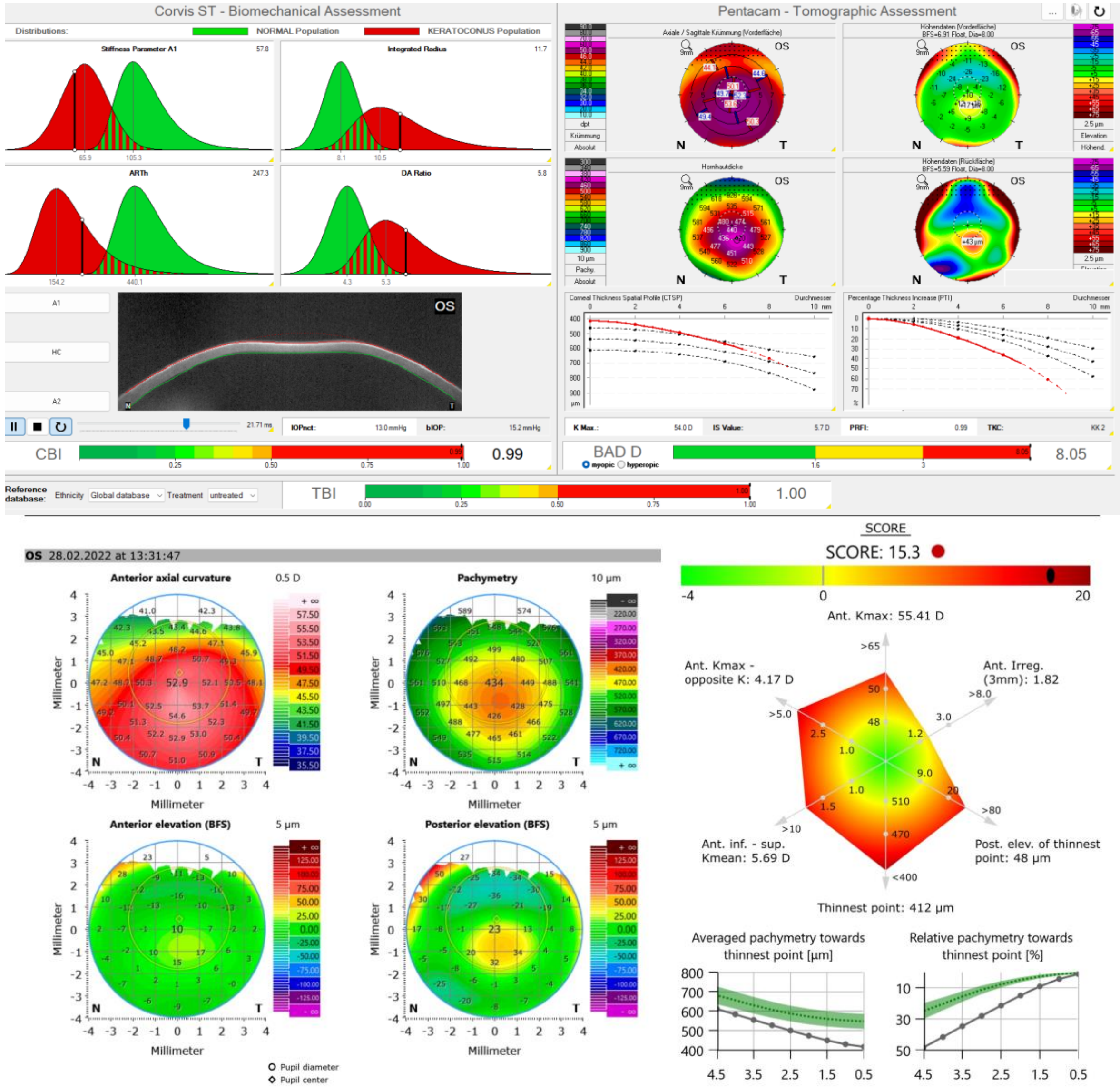
Corvis ST - Biomechanical Assessment
Pentacam - Tomographic Assessment
NORMAL Population
KERATOCONUS Population
OS
CBI
0.99
BAD D
8.05
TBI
1.00
OS 28.02.2022 at 13:31:47
Anterior axial curvature
Pachymetry
Anterior elevation (BFS)
Posterior elevation (BFS)
Millimeter
SCORE
SCORE: 15.3
Ant. Kmax: 55.41 D
Ant. Kmax - opposite K: 4.17 D
Ant. Irreg. (3mm): 1.82
Ant. inf. - sup. Kmean: 5.69 D
Post. elev. of thinnest point: 48 µm
Thinnest point: 412 µm
Averaged pachymetry towards thinnest point [µm]
Relative pachymetry towards thinnest point [%]
Pupil diameter
Pupil center

CASE #23

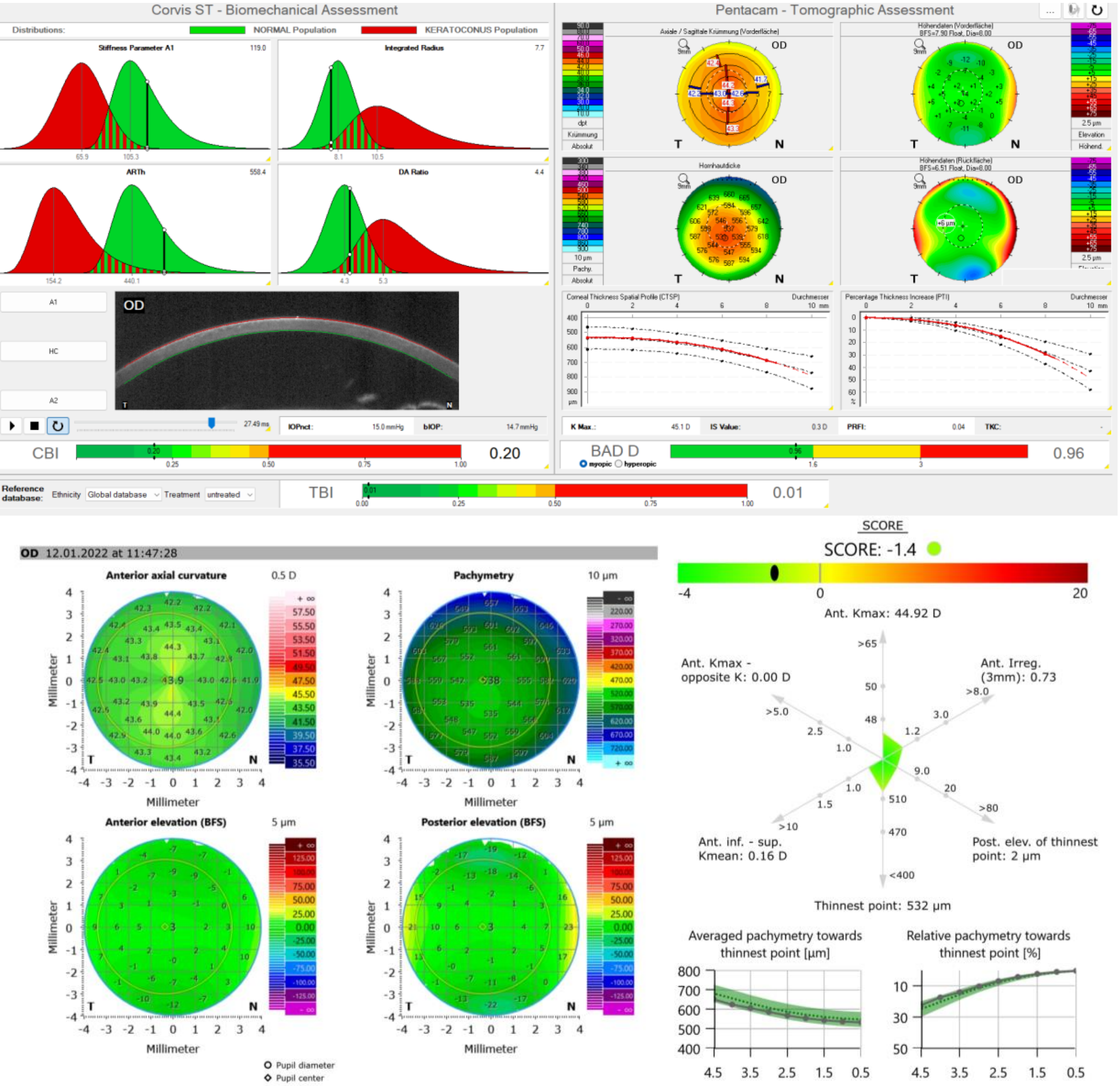

Excluded for analysis due to prior CXL

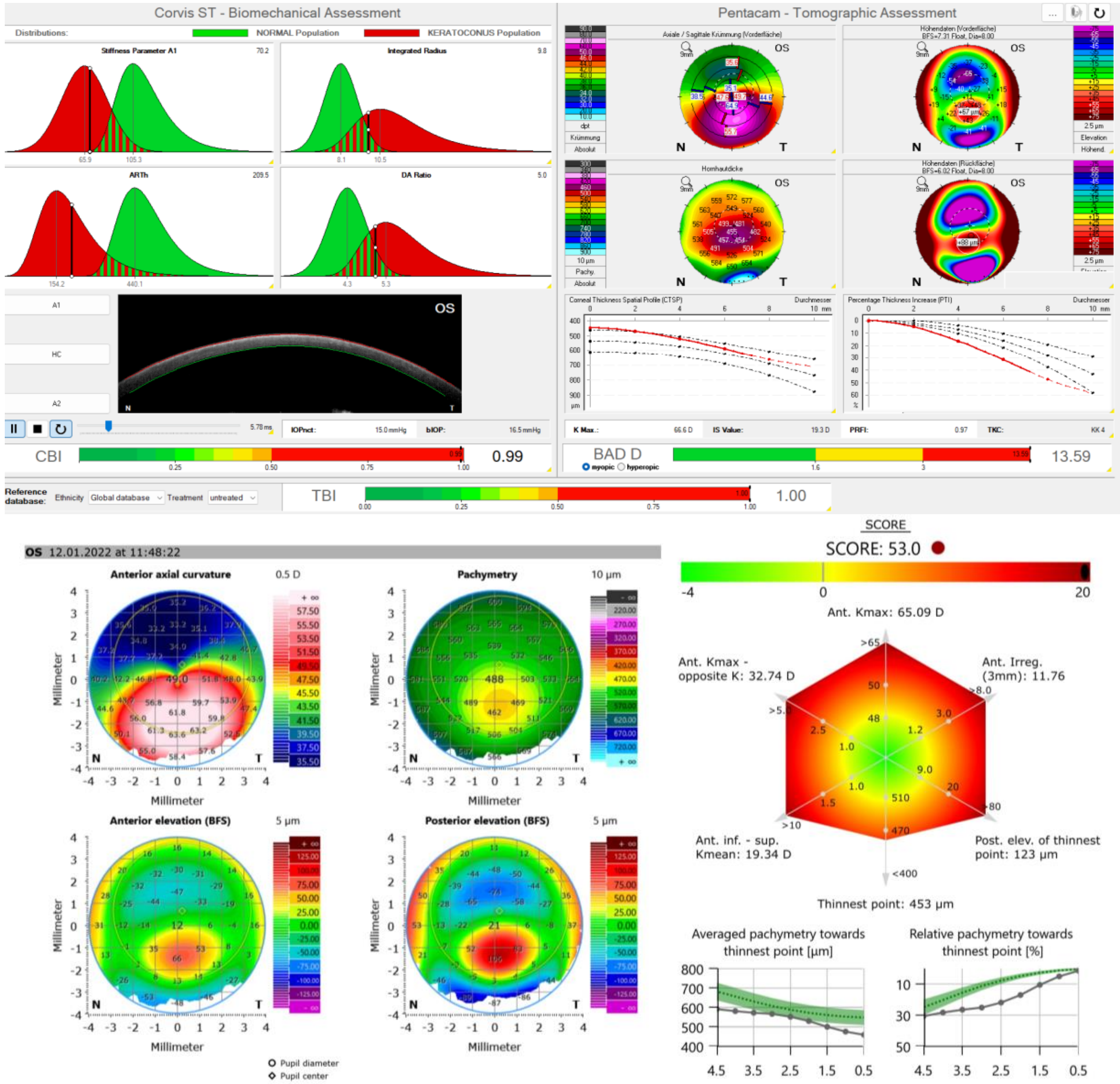

CASE #24

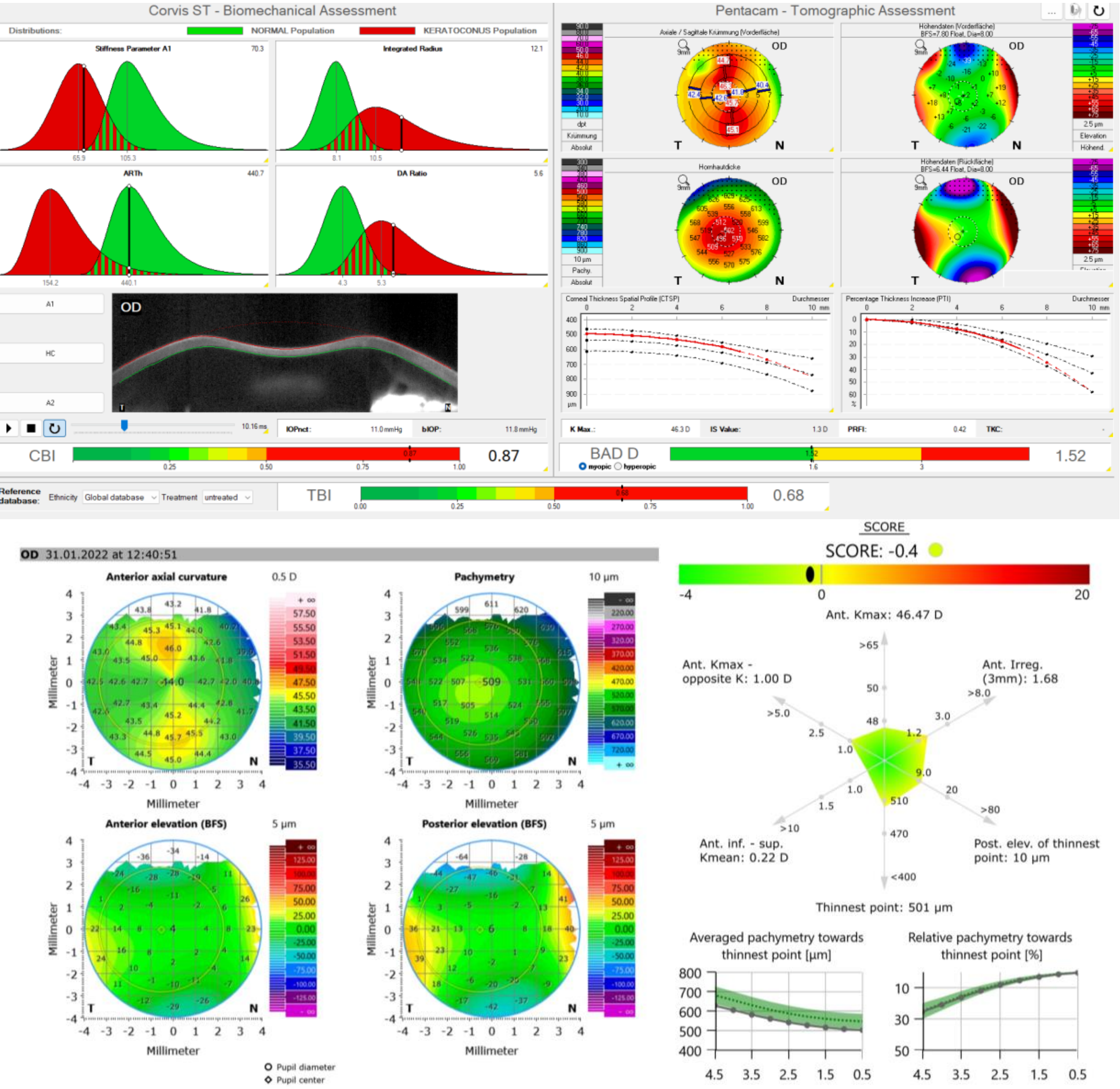

Corvis ST - Biomechanical Assessment
Pentacam - Tomographic Assessment
OD
CBI 0.87
BAD D 1.52
TBI 0.68
OD 31.01.2022 at 12:40:51
Anterior axial curvature
Pachymetry
Anterior elevation (BFS)
Posterior elevation (BFS)
SCORE
SCORE: -0.4
Ant. Kmax: 46.47 D
Ant. Kmax - opposite K: 1.00 D
Ant. Irreg. (3mm): 1.68
Ant. inf. - sup. Kmean: 0.22 D
Post. elev. of thinnest point: 10 µm
Thinnest point: 501 µm
Averaged pachymetry towards thinnest point [µm]
Relative pachymetry towards thinnest point [%]

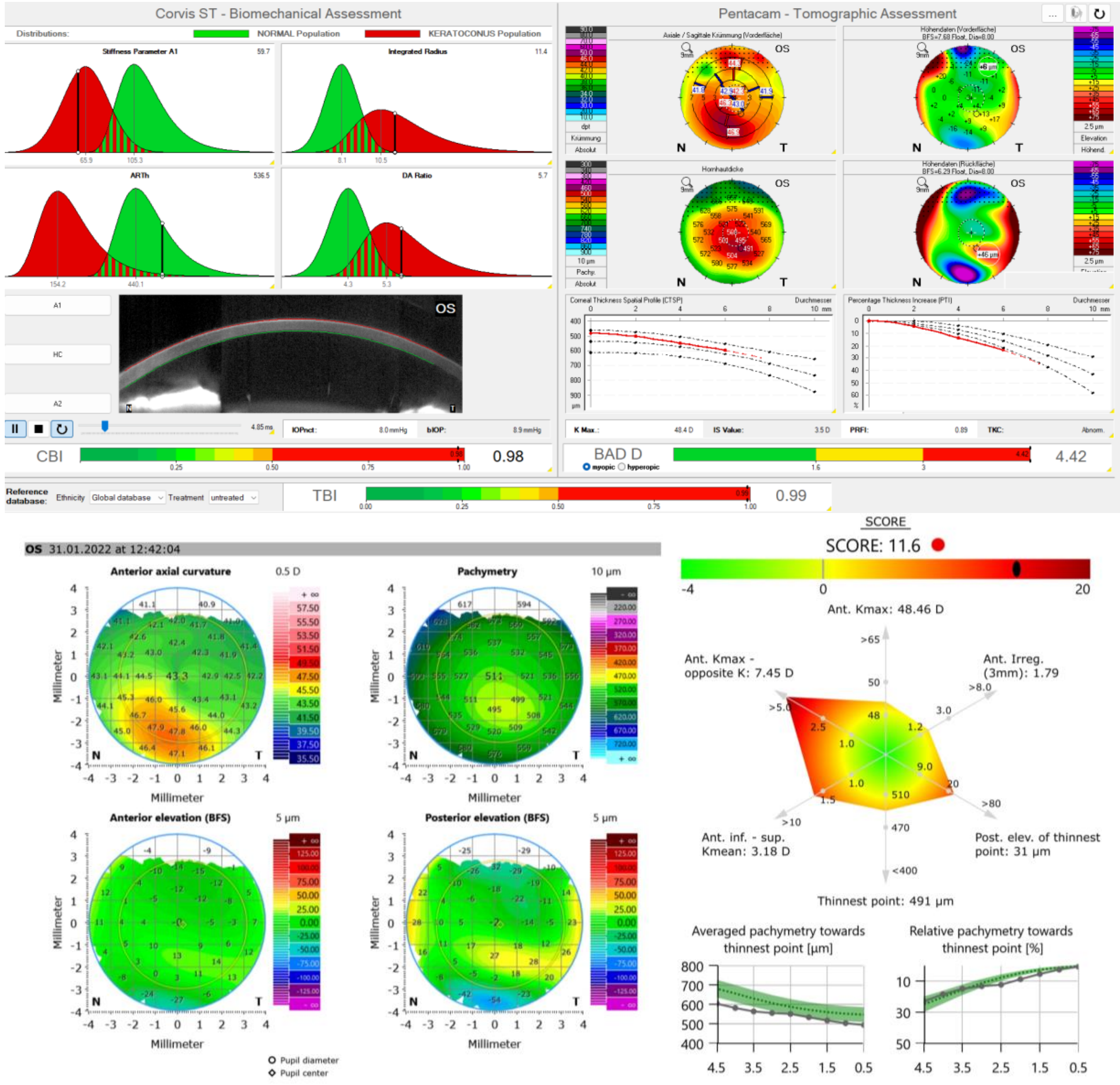

Corvis ST - Biomechanical Assessment
Pentacam - Tomographic Assessment
OS
CBI 0.98
BAD D 4.42
TBI 0.99
OS 31.01.2022 at 12:42:04
Anterior axial curvature
Pachymetry
Anterior elevation (BFS)
Posterior elevation (BFS)
SCORE
SCORE: 11.6
Ant. Kmax: 48.46 D
Ant. Kmax - opposite K: 7.45 D
Ant. Irreg. (3mm): 1.79
Ant. inf. - sup. Kmean: 3.18 D
Post. elev. of thinnest point: 31 µm
Thinnest point: 491 µm
Averaged pachymetry towards thinnest point [µm]
Relative pachymetry towards thinnest point [%]

CASE #25

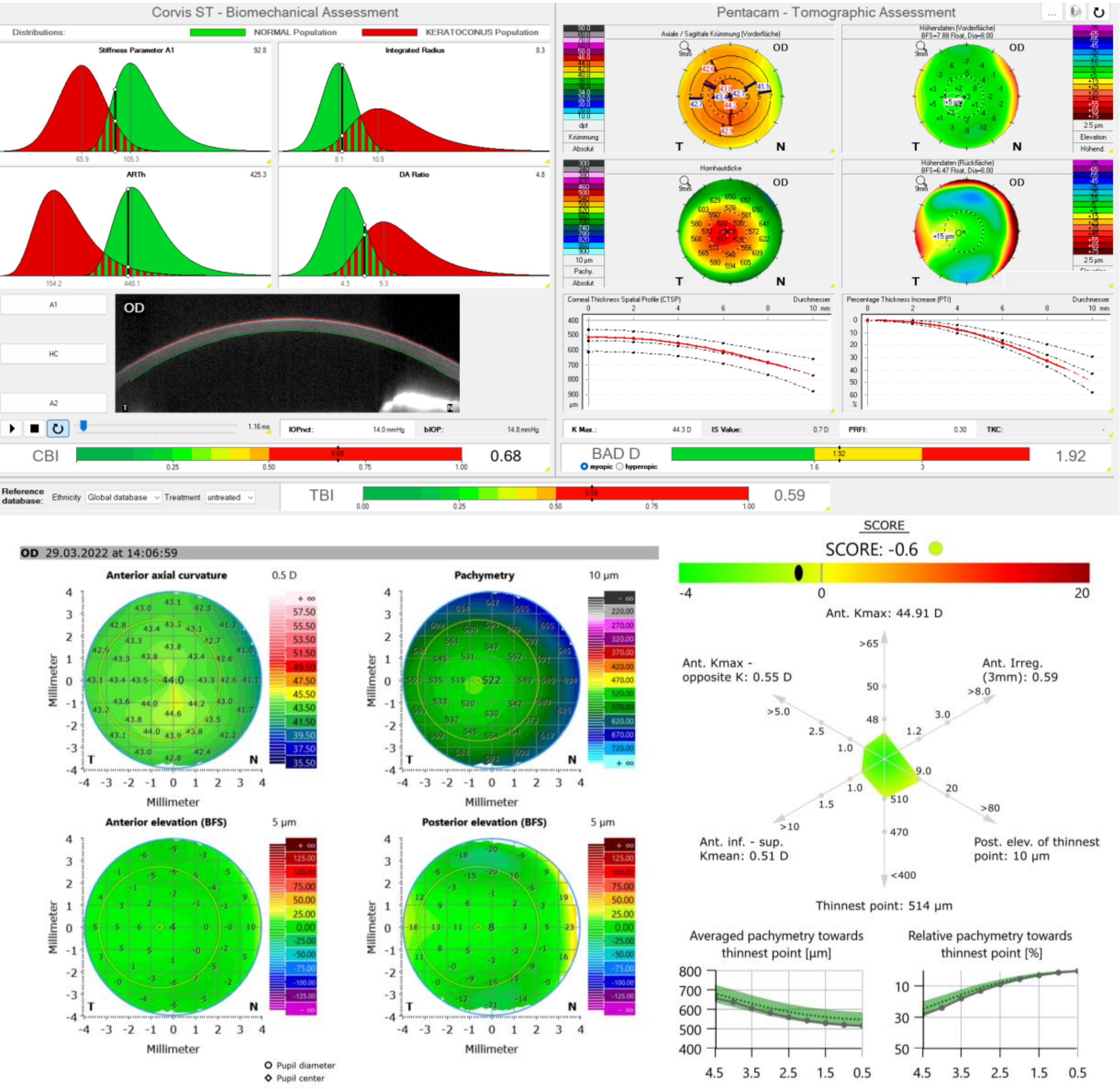
Corvis ST - Biomechanical Assessment
Pentacam - Tomographic Assessment
NORMAL Population
KERATOCONUS Population
Stiffness Parameter A1
Integrated Radius
ARTh
DA Ratio
OD
CBI
0.68
BAD D
1.92
TBI
0.59
OD 29.03.2022 at 14:06:59
Anterior axial curvature
Pachymetry
Anterior elevation (BFS)
Posterior elevation (BFS)
Millimeter
SCORE
SCORE: -0.6
Ant. Kmax: 44.91 D
Ant. Kmax - opposite K: 0.55 D
Ant. Irreg. (3mm): 0.59
Ant. inf. - sup. Kmean: 0.51 D
Post. elev. of thinnest point: 10 µm
Thinnest point: 514 µm
Averaged pachymetry towards thinnest point [µm]
Relative pachymetry towards thinnest point [%]
Pupil diameter
Pupil center

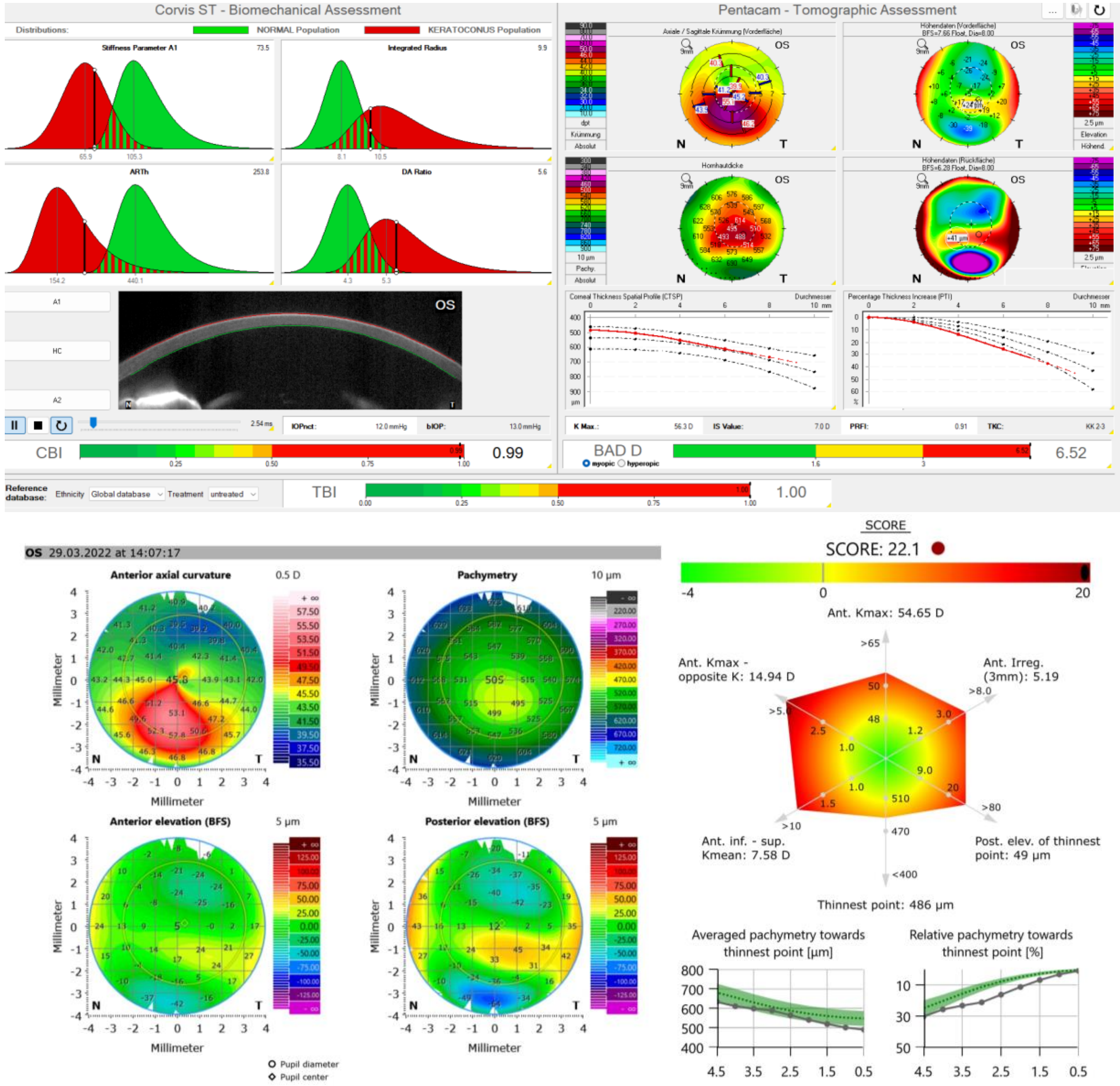
Corvis ST - Biomechanical Assessment
Pentacam - Tomographic Assessment
NORMAL Population
KERATOCONUS Population
Stiffness Parameter A1
Integrated Radius
ARTh
DA Ratio
OS
CBI
0.99
BAD D
6.52
TBI
1.00
OS 29.03.2022 at 14:07:17
Anterior axial curvature
Pachymetry
Anterior elevation (BFS)
Posterior elevation (BFS)
Millimeter
SCORE
SCORE: 22.1
Ant. Kmax: 54.65 D
Ant. Kmax - opposite K: 14.94 D
Ant. Irreg. (3mm): 5.19
Ant. inf. - sup. Kmean: 7.58 D
Post. elev. of thinnest point: 49 µm
Thinnest point: 486 µm
Averaged pachymetry towards thinnest point [µm]
Relative pachymetry towards thinnest point [%]
Pupil diameter
Pupil center

CASE #26

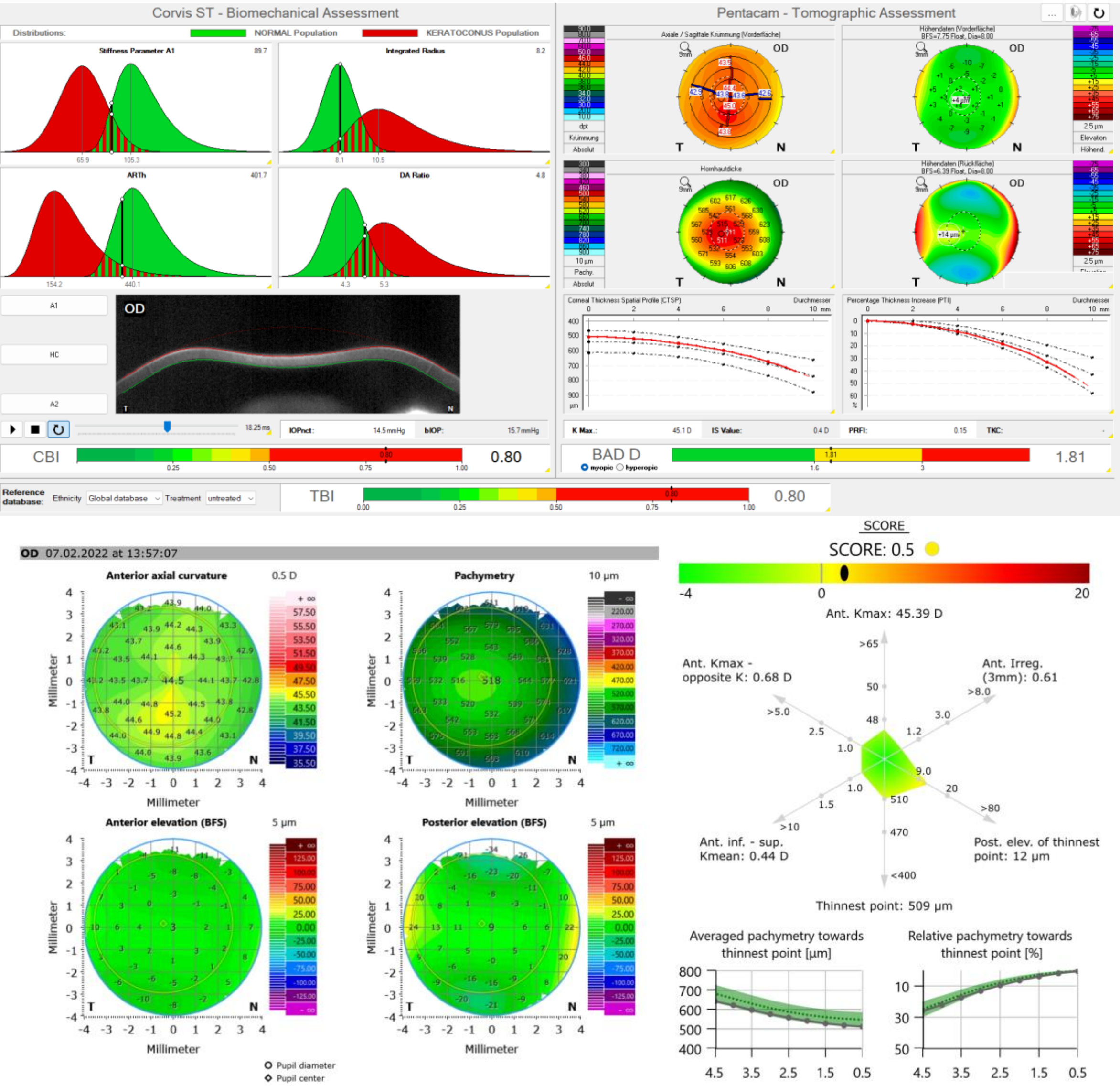

Excluded for analysis due to prior CXL

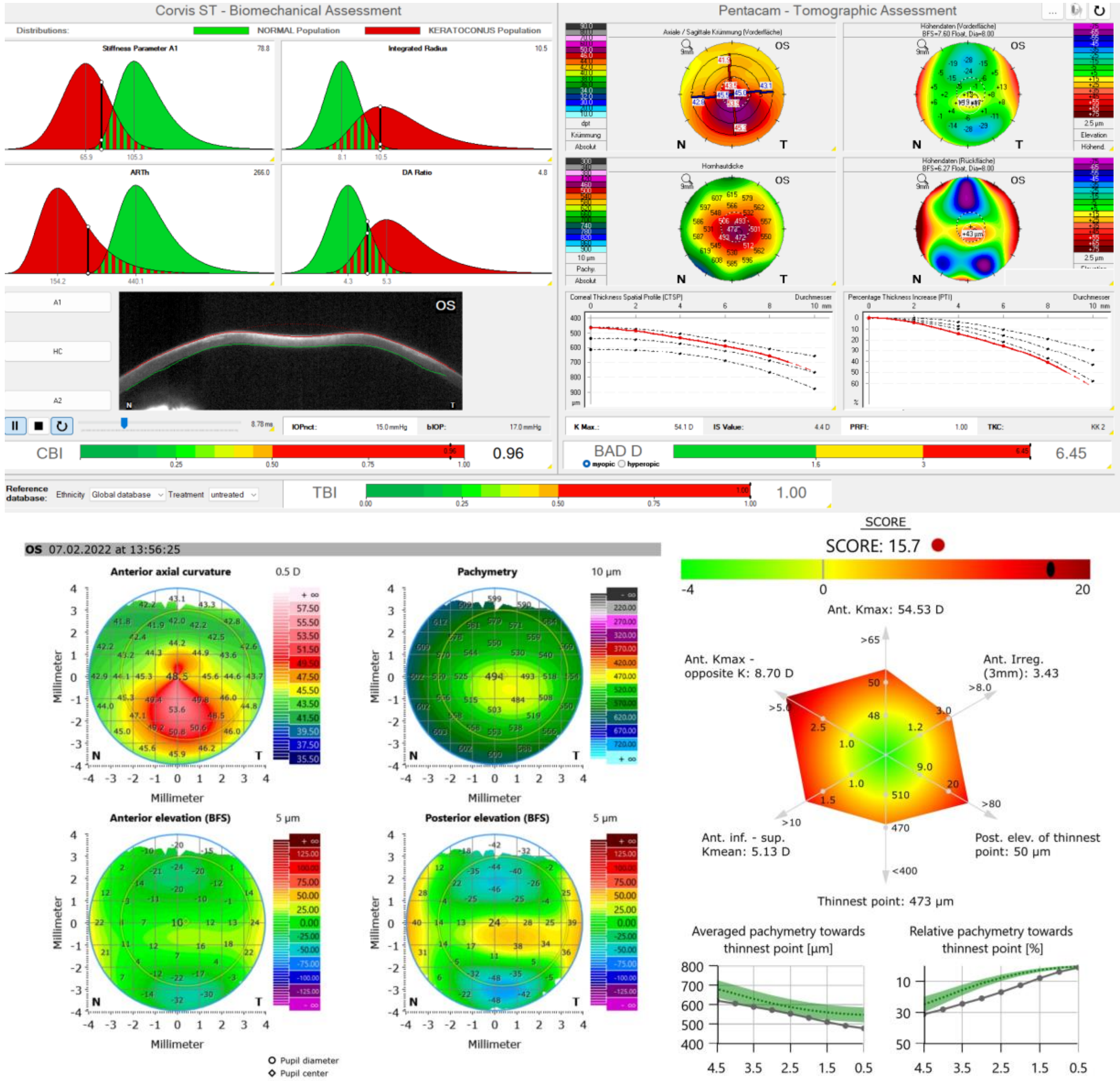

CASE #27

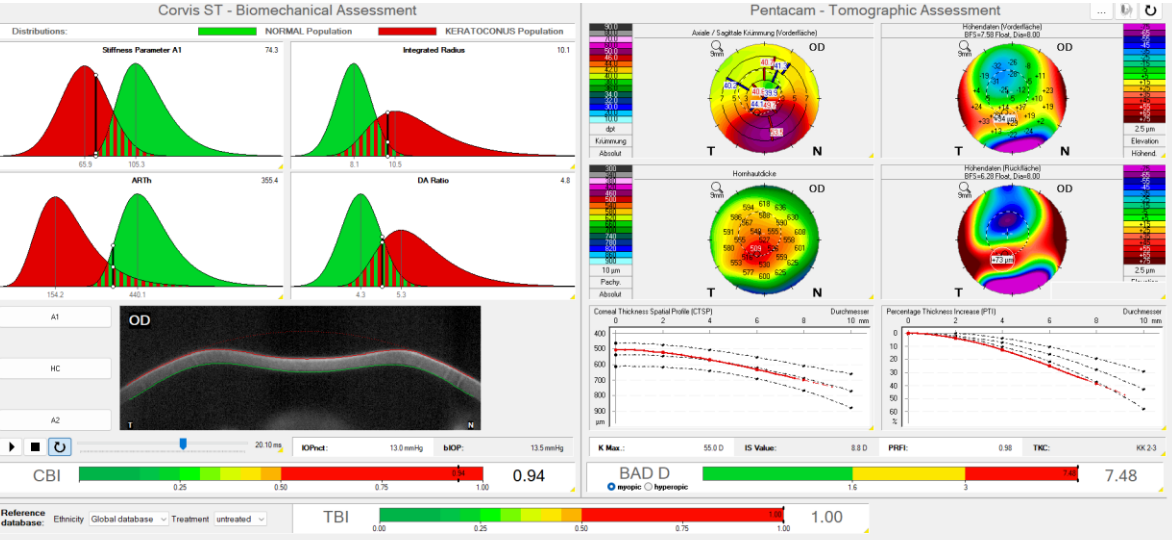

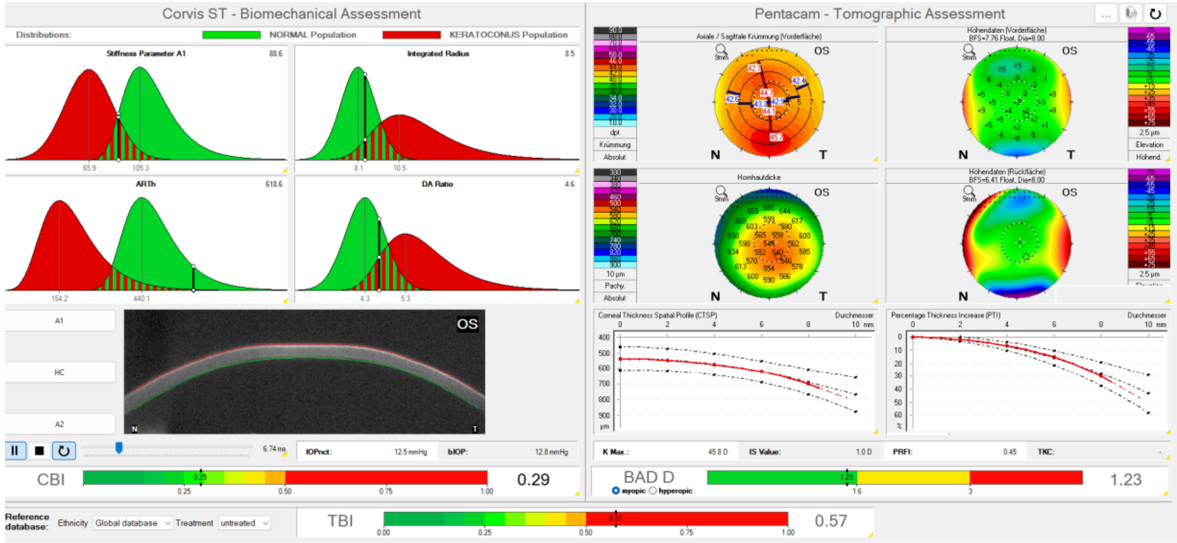

Measurements were lost in the ANTERION device

CASE #28

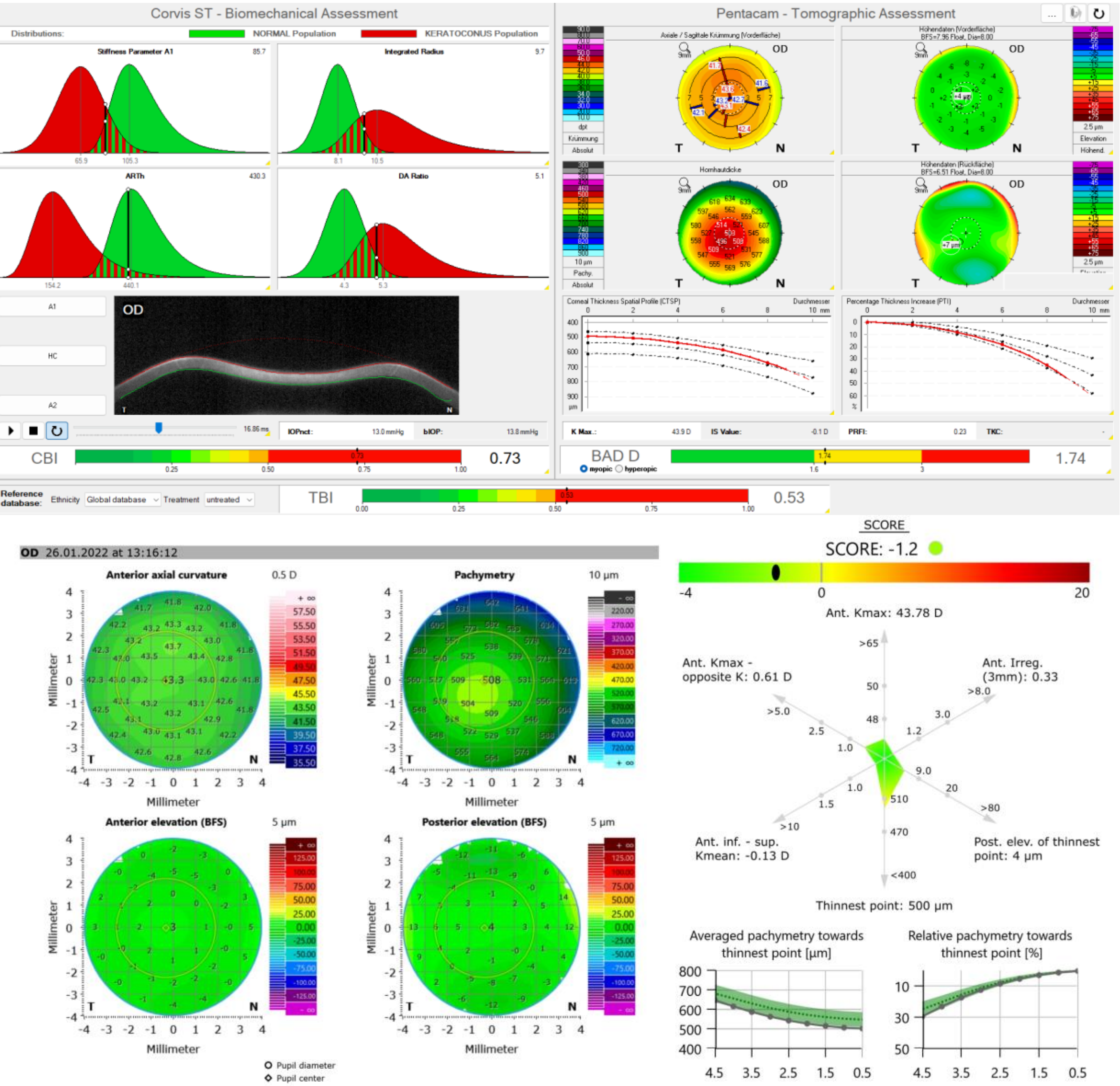

Corvis ST - Biomechanical Assessment
Pentacam - Tomographic Assessment
OD
CBI 0.73
BAD D 1.74
TBI 0.53
OD 26.01.2022 at 13:16:12
Anterior axial curvature
Pachymetry
Anterior elevation (BFS)
Posterior elevation (BFS)
SCORE
SCORE: -1.2
Ant. Kmax: 43.78 D
Ant. Kmax - opposite K: 0.61 D
Ant. Irreg. (3mm): 0.33
Ant. inf. - sup. Kmean: -0.13 D
Post. elev. of thinnest point: 4 µm
Thinnest point: 500 µm
Averaged pachymetry towards thinnest point [µm]
Relative pachymetry towards thinnest point [%]

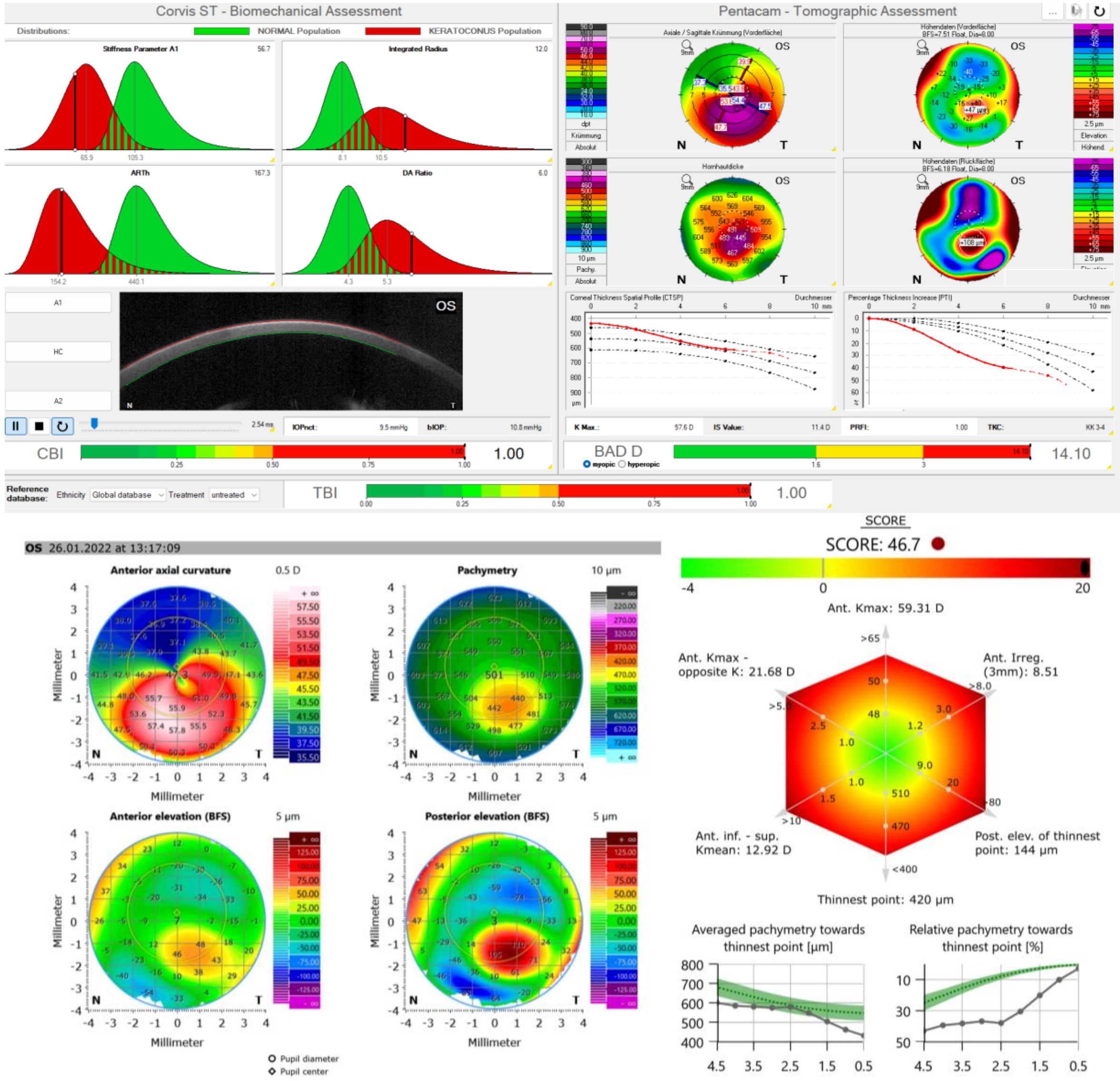

Corvis ST - Biomechanical Assessment
Pentacam - Tomographic Assessment
OS
CBI 1.00
BAD D 14.10
TBI 1.00
OS 26.01.2022 at 13:17:09
Anterior axial curvature
Pachymetry
Anterior elevation (BFS)
Posterior elevation (BFS)
SCORE
SCORE: 46.7
Ant. Kmax: 59.31 D
Ant. Kmax - opposite K: 21.68 D
Ant. Irreg. (3mm): 8.51
Ant. inf. - sup. Kmean: 12.92 D
Post. elev. of thinnest point: 144 µm
Thinnest point: 420 µm
Averaged pachymetry towards thinnest point [µm]
Relative pachymetry towards thinnest point [%]

CASE #29

Excluded for analysis due to prior CXL

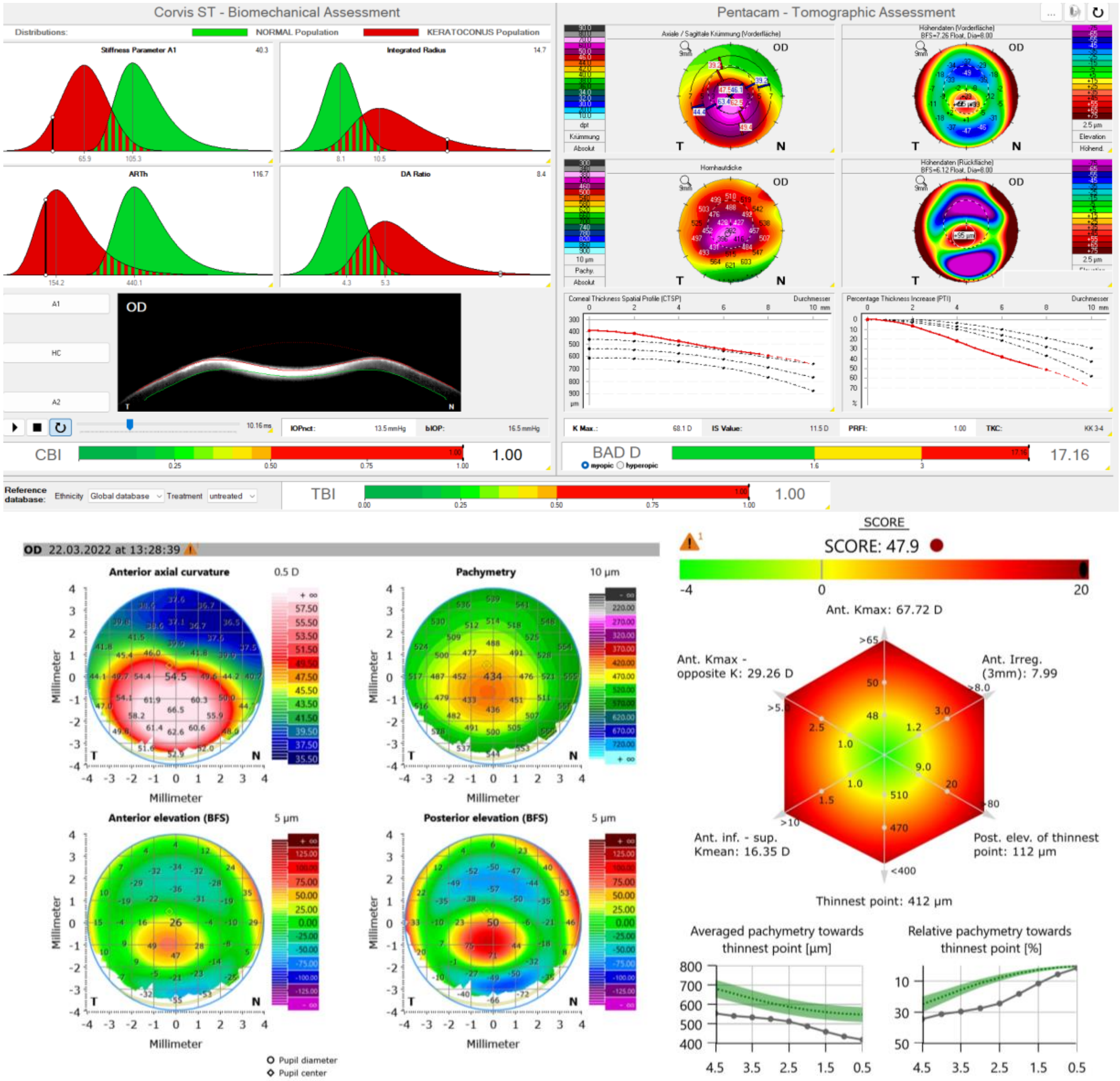

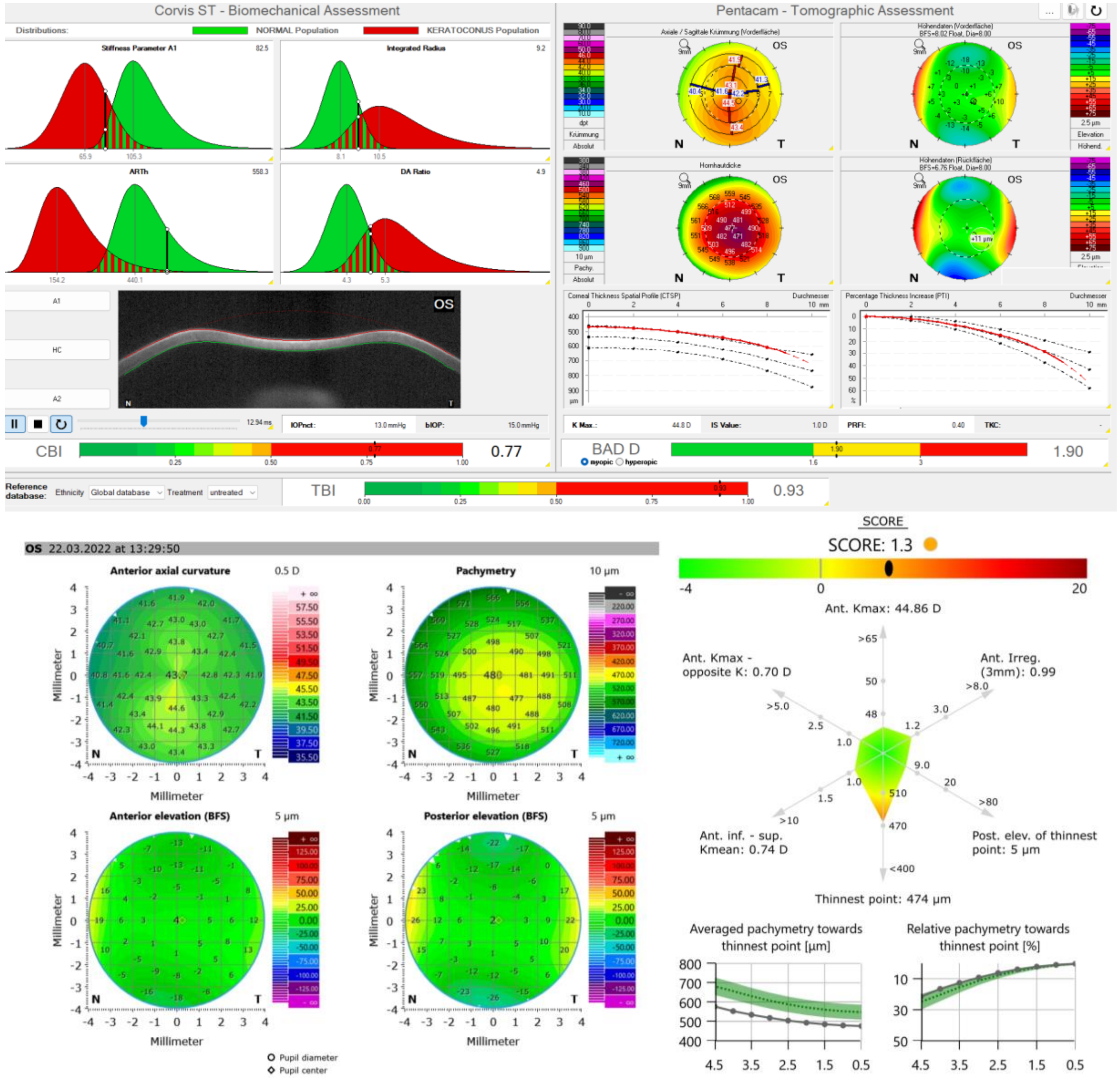

CASE #30

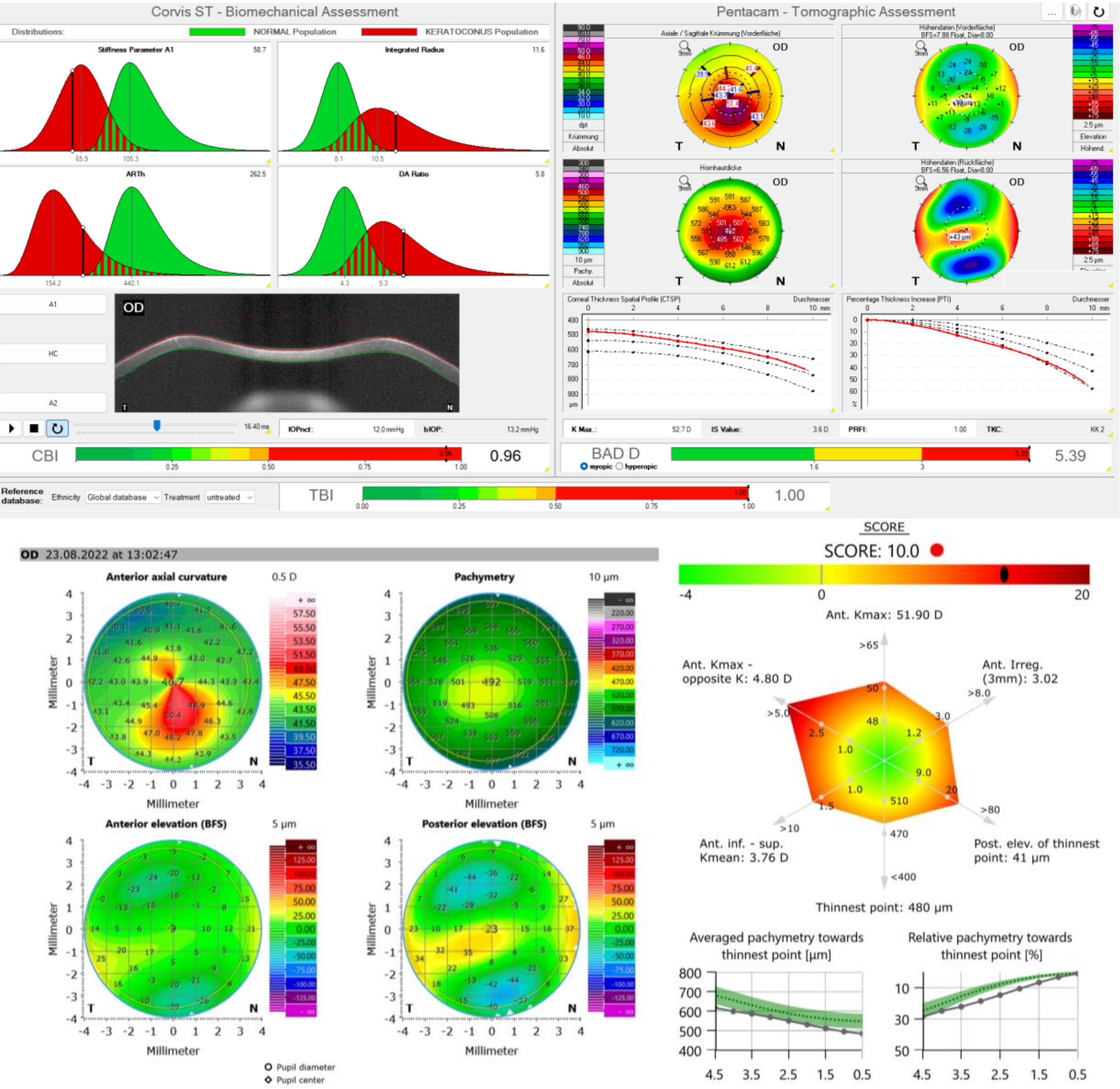
Corvis ST - Biomechanical Assessment
Pentacam - Tomographic Assessment
OD
CBI 0.96
BAD D 5.39
TBI 1.00
OD 23.08.2022 at 13:02:47
Anterior axial curvature
Pachymetry
Anterior elevation (BFS)
Posterior elevation (BFS)
SCORE
SCORE: 10.0
Ant. Kmax: 51.90 D
Ant. Kmax - opposite K: 4.80 D
Ant. Irreg. (3mm): 3.02
Ant. inf. - sup. Kmean: 3.76 D
Post. elev. of thinnest point: 41 µm
Thinnest point: 480 µm
Averaged pachymetry towards thinnest point [µm]
Relative pachymetry towards thinnest point [%]
Millimeter
Pupil diameter
Pupil center

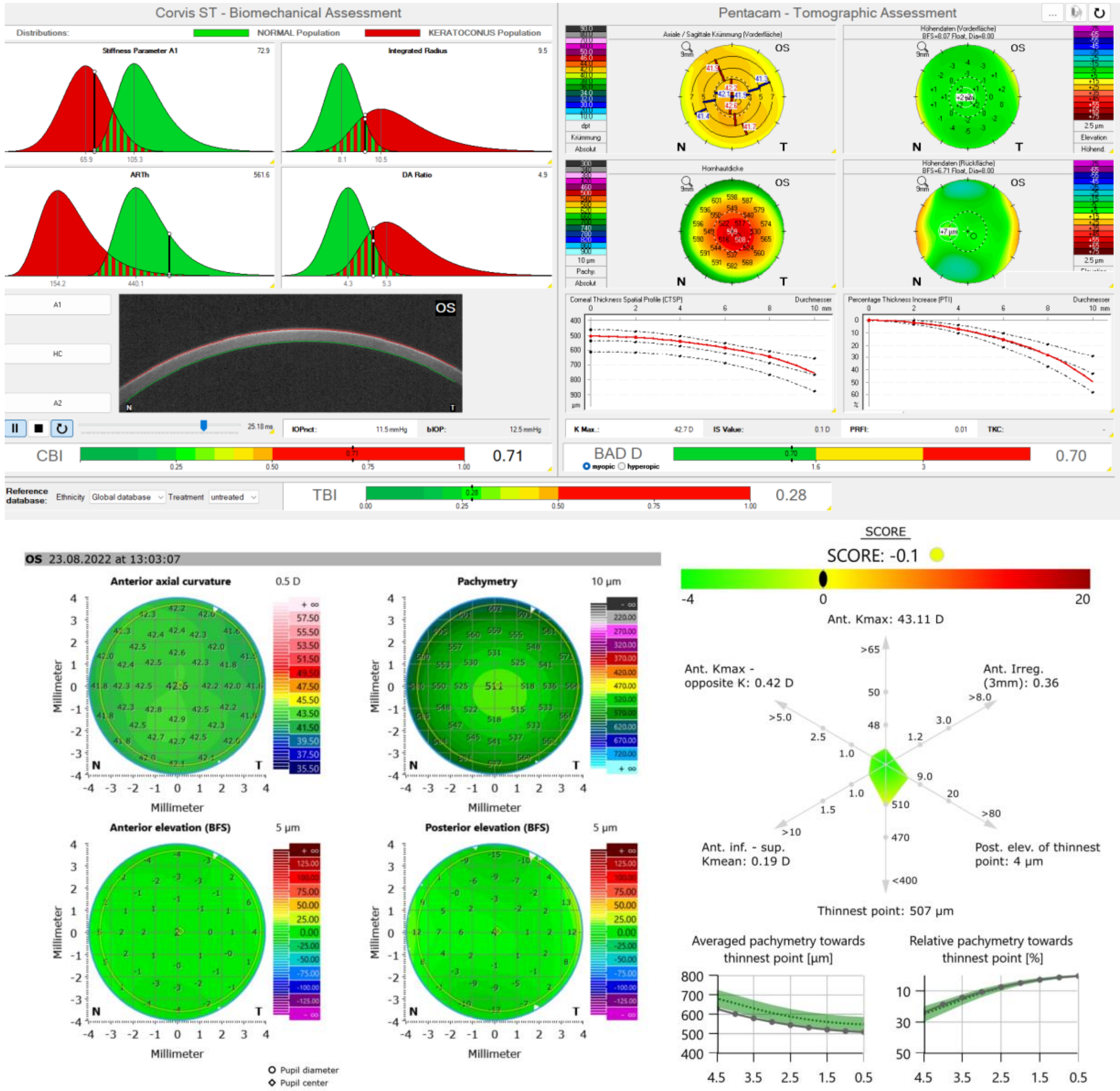
Corvis ST - Biomechanical Assessment
Pentacam - Tomographic Assessment
OS
CBI 0.71
BAD D 0.70
TBI 0.28
OS 23.08.2022 at 13:03:07
Anterior axial curvature
Pachymetry
Anterior elevation (BFS)
Posterior elevation (BFS)
SCORE
SCORE: -0.1
Ant. Kmax: 43.11 D
Ant. Kmax - opposite K: 0.42 D
Ant. Irreg. (3mm): 0.36
Ant. inf. - sup. Kmean: 0.19 D
Post. elev. of thinnest point: 4 µm
Thinnest point: 507 µm
Averaged pachymetry towards thinnest point [µm]
Relative pachymetry towards thinnest point [%]
Millimeter
Pupil diameter
Pupil center

CASE #31

Excluded for analysis due to prior CXL, Corvis not available

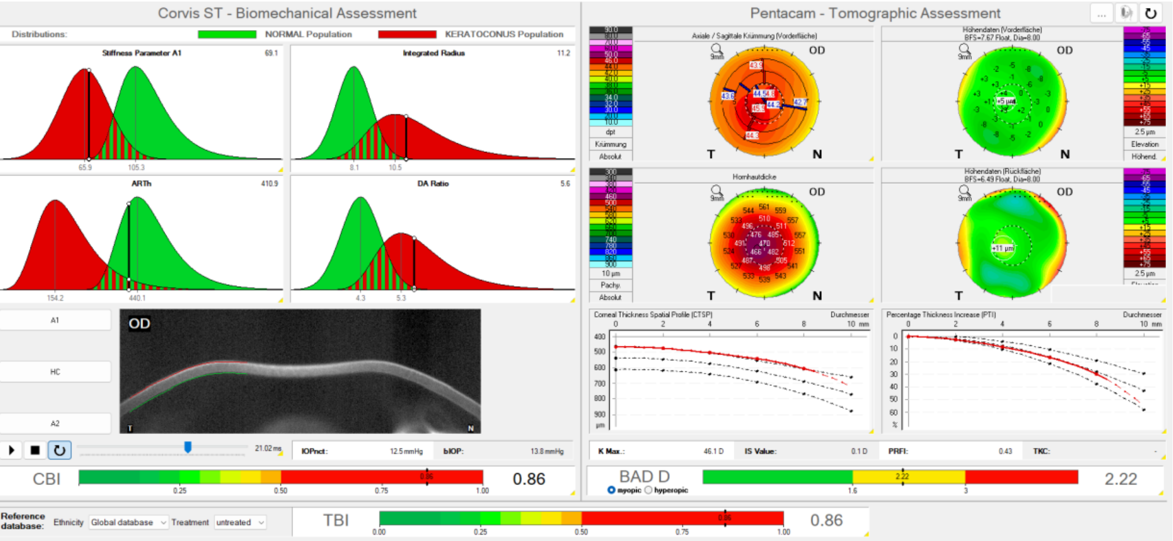

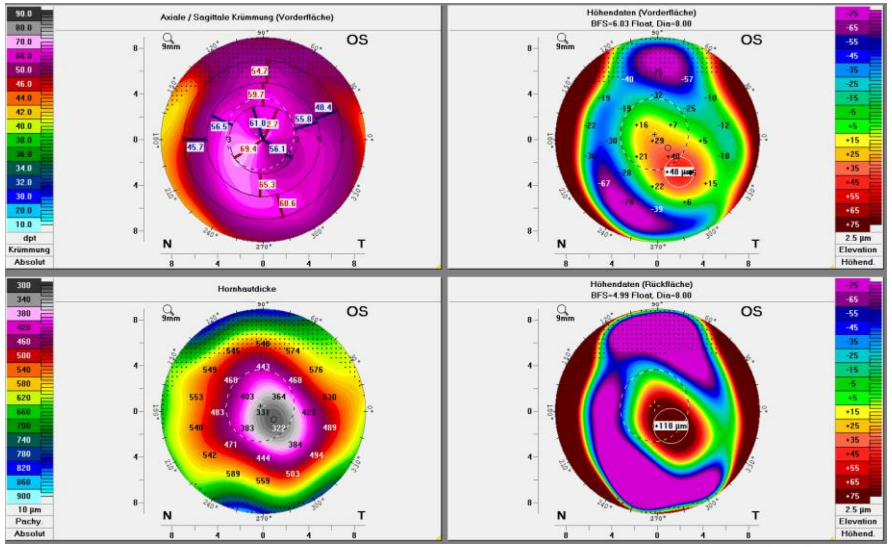

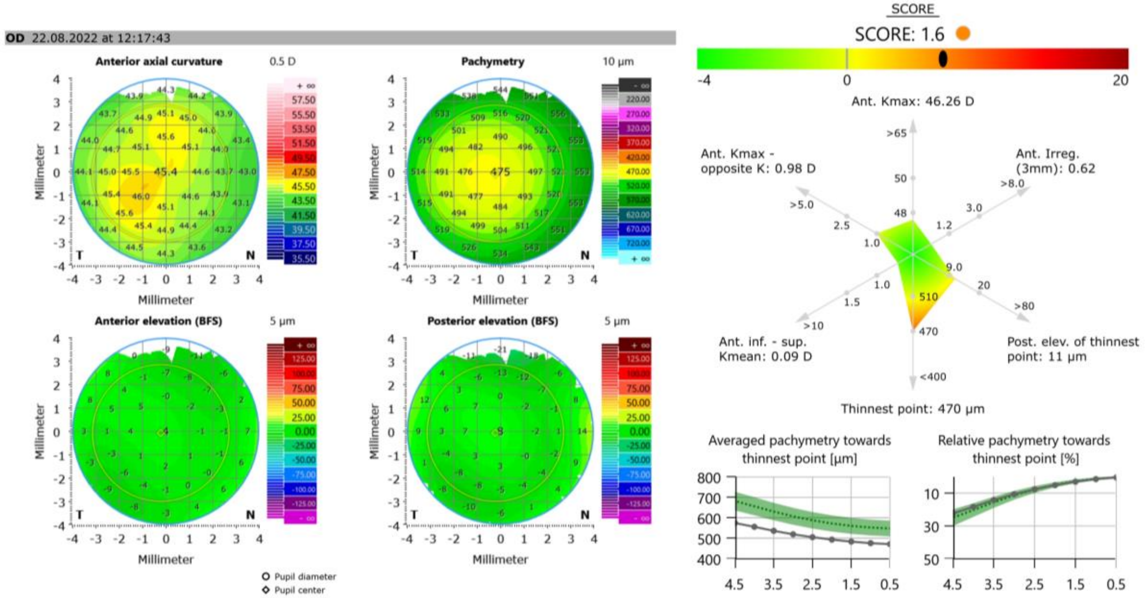

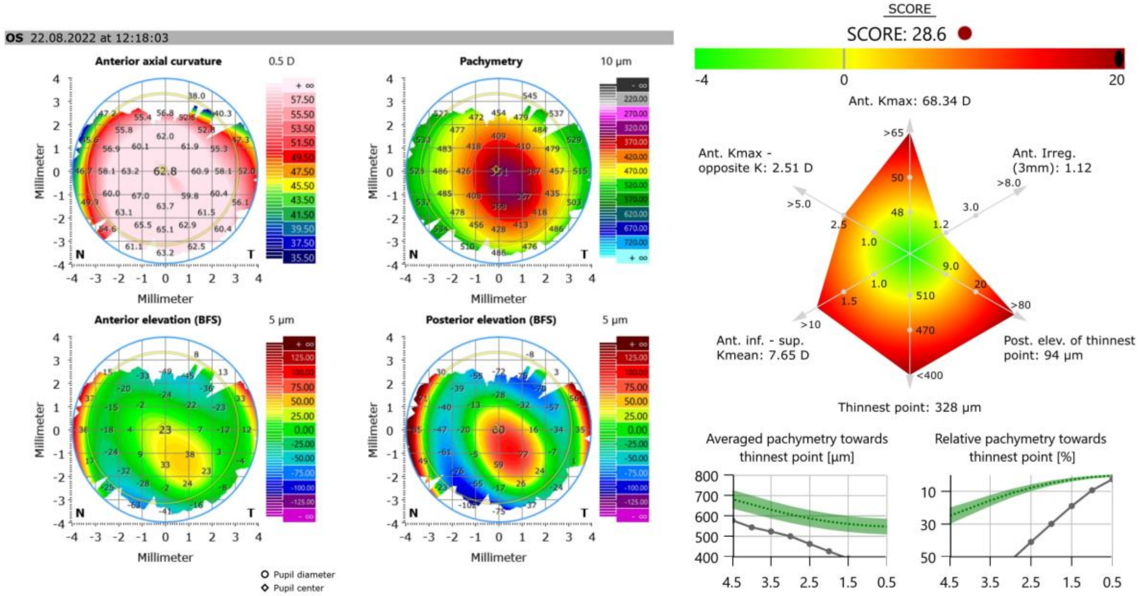

CASE #32

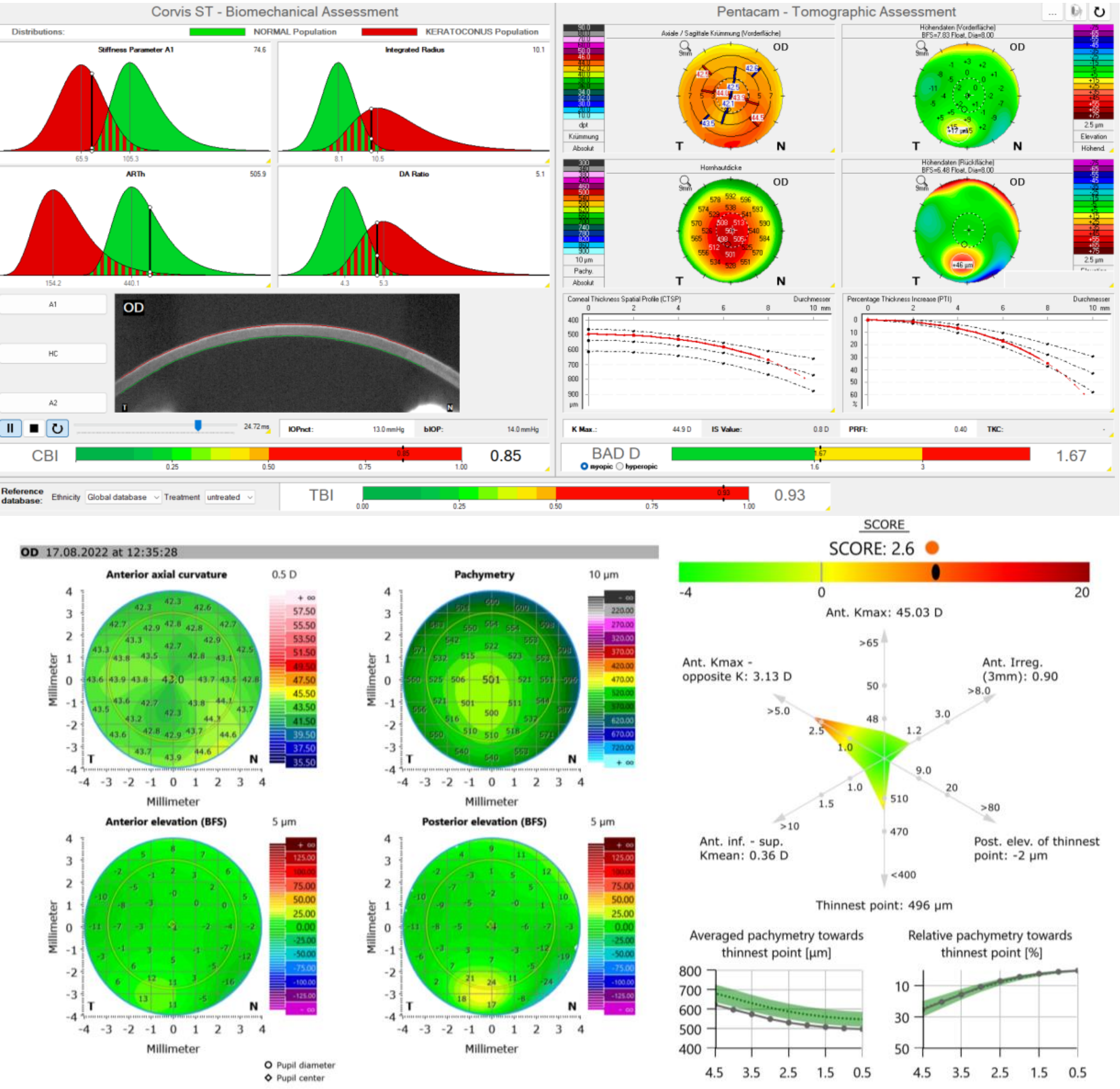

Corvis ST - Biomechanical Assessment
Pentacam - Tomographic Assessment
CBI 0.85
BAD D 1.67
TBI 0.93
OD 17.08.2022 at 12:35:28
SCORE: 2.6
Ant. Kmax: 45.03 D
Ant. Kmax - opposite K: 3.13 D
Ant. Irreg. (3mm): 0.90
Ant. inf. - sup. Kmean: 0.36 D
Post. elev. of thinnest point: -2 µm
Thinnest point: 496 µm
Averaged pachymetry towards thinnest point [µm]
Relative pachymetry towards thinnest point [%]

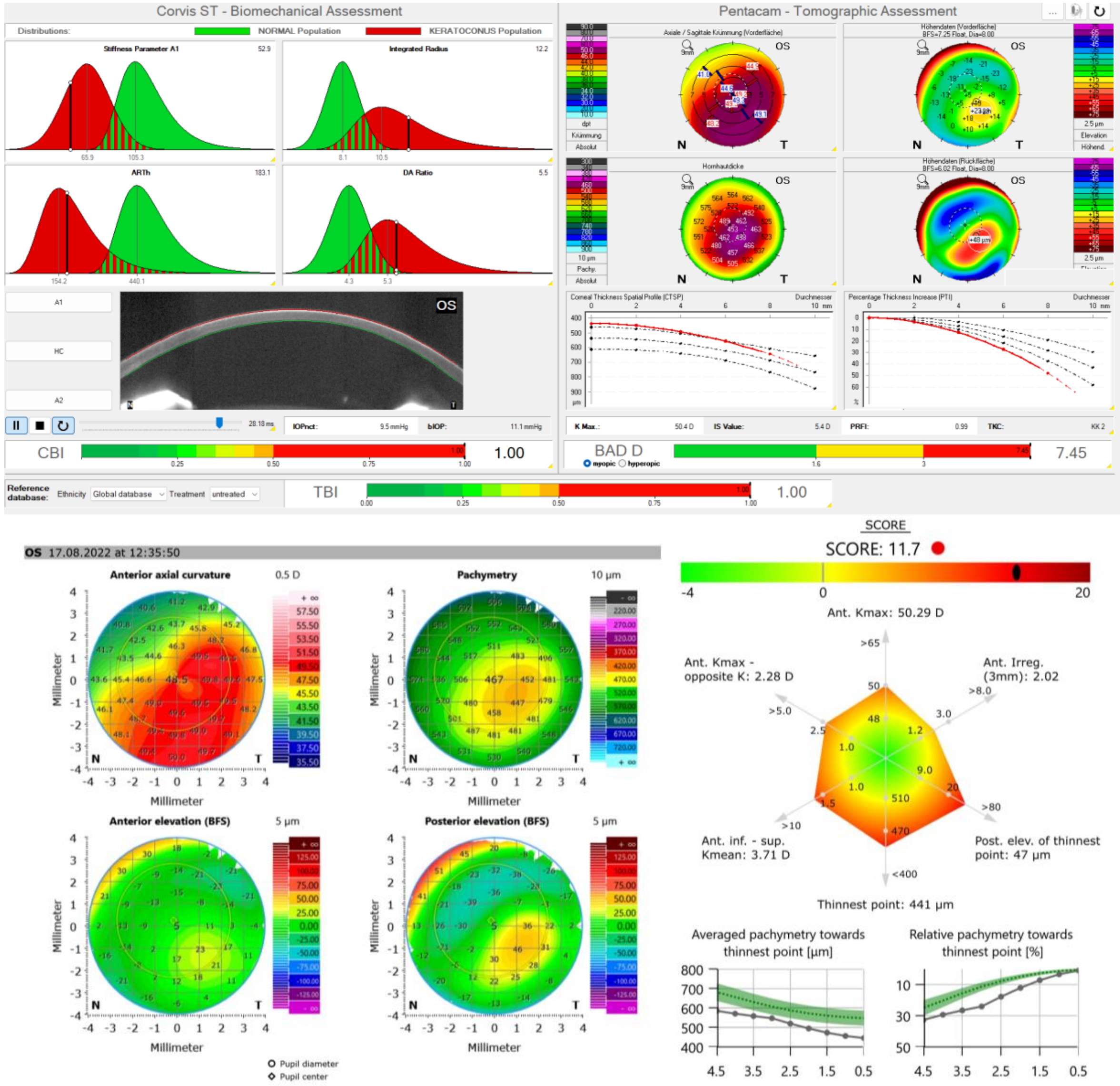

Corvis ST - Biomechanical Assessment
Pentacam - Tomographic Assessment
CBI 1.00
BAD D 7.45
TBI 1.00
OS 17.08.2022 at 12:35:50
SCORE: 11.7
Ant. Kmax: 50.29 D
Ant. Kmax - opposite K: 2.28 D
Ant. Irreg. (3mm): 2.02
Ant. inf. - sup. Kmean: 3.71 D
Post. elev. of thinnest point: 47 µm
Thinnest point: 441 µm
Averaged pachymetry towards thinnest point [µm]
Relative pachymetry towards thinnest point [%]

CASE #33

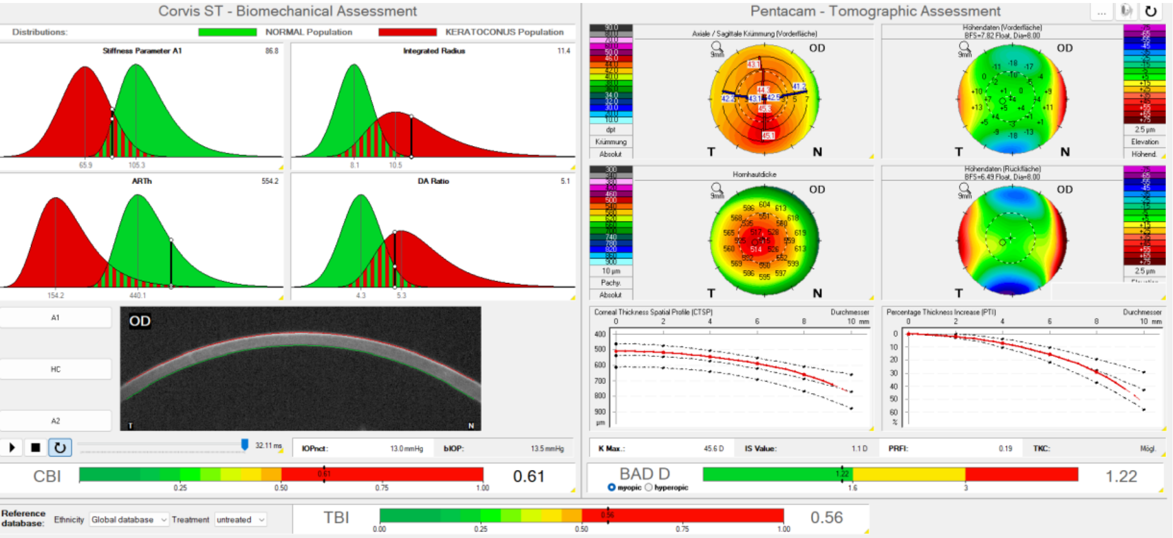
Corvis ST - Biomechanical Assessment
Pentacam - Tomographic Assessment
NORMAL Population
KERATOCONUS Population
Stiffness Parameter A1
Integrated Radius
ARTh
DA Ratio
OD
CBI 0.61
BAD D 1.22
TBI 0.56
Reference database: Ethnicity Global database Treatment untreated

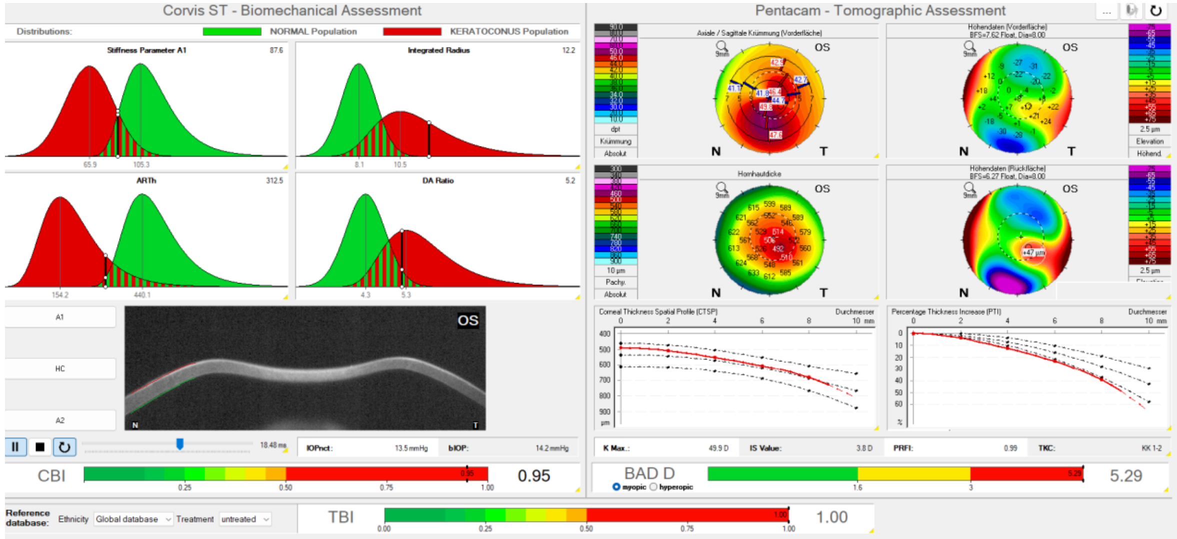
Corvis ST - Biomechanical Assessment
Pentacam - Tomographic Assessment
NORMAL Population
KERATOCONUS Population
Stiffness Parameter A1
Integrated Radius
ARTh
DA Ratio
OS
CBI 0.95
BAD D 5.29
TBI 1.00
Reference database: Ethnicity Global database Treatment untreated

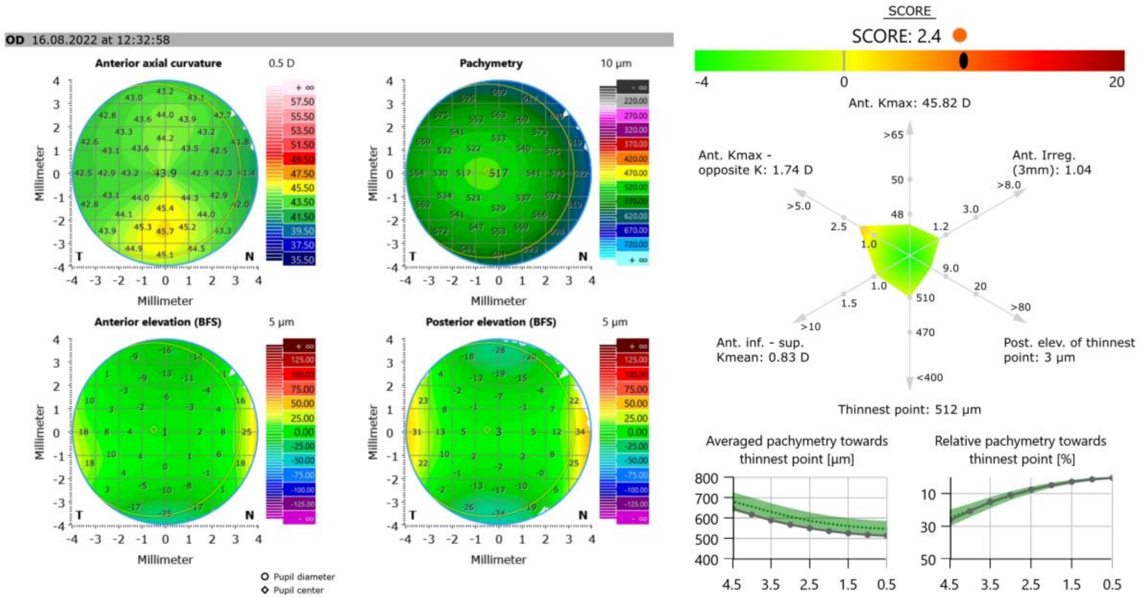
OD 16.08.2022 at 12:32:58
Anterior axial curvature
Pachymetry
Anterior elevation (BFS)
Posterior elevation (BFS)
Millimeter
Pupil diameter
Pupil center
SCORE
SCORE: 2.4
Ant. Kmax: 45.82 D
Ant. Kmax - opposite K: 1.74 D
Ant. Irreg. (3mm): 1.04
Ant. inf. - sup. Kmean: 0.83 D
Post. elev. of thinnest point: 3 µm
Thinnest point: 512 µm
Averaged pachymetry towards thinnest point [µm]
Relative pachymetry towards thinnest point [%]

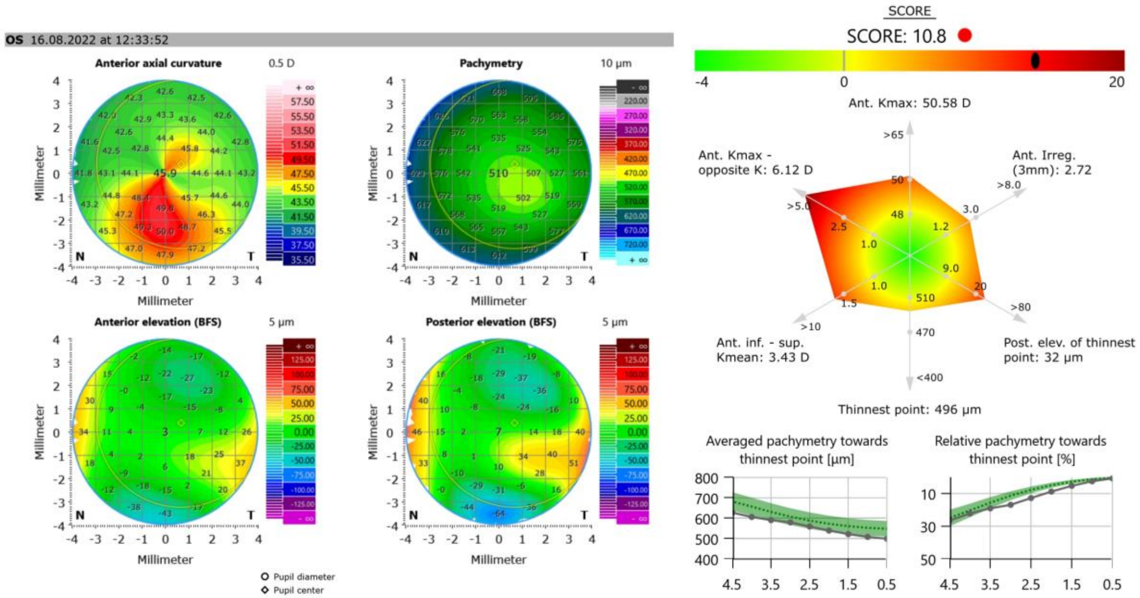
OS 16.08.2022 at 12:33:52
Anterior axial curvature
Pachymetry
Anterior elevation (BFS)
Posterior elevation (BFS)
Millimeter
Pupil diameter
Pupil center
SCORE
SCORE: 10.8
Ant. Kmax: 50.58 D
Ant. Kmax - opposite K: 6.12 D
Ant. Irreg. (3mm): 2.72
Ant. inf. - sup. Kmean: 3.43 D
Post. elev. of thinnest point: 32 µm
Thinnest point: 496 µm
Averaged pachymetry towards thinnest point [µm]
Relative pachymetry towards thinnest point [%]

CASE #34

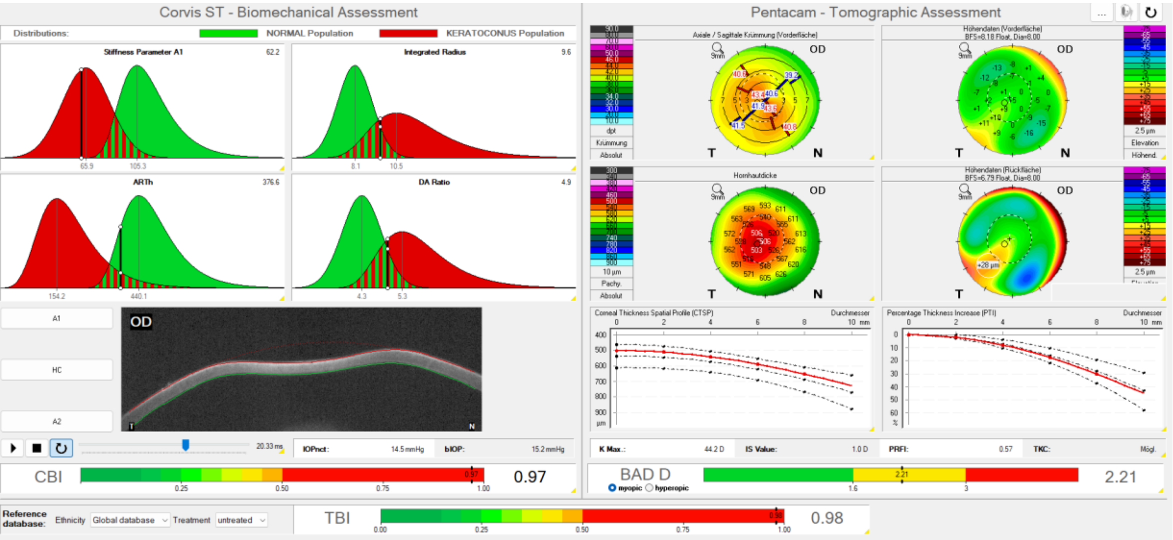
Corvis ST - Biomechanical Assessment
Pentacam - Tomographic Assessment
OD
CBI 0.97
BAD D 2.21
TBI 0.98

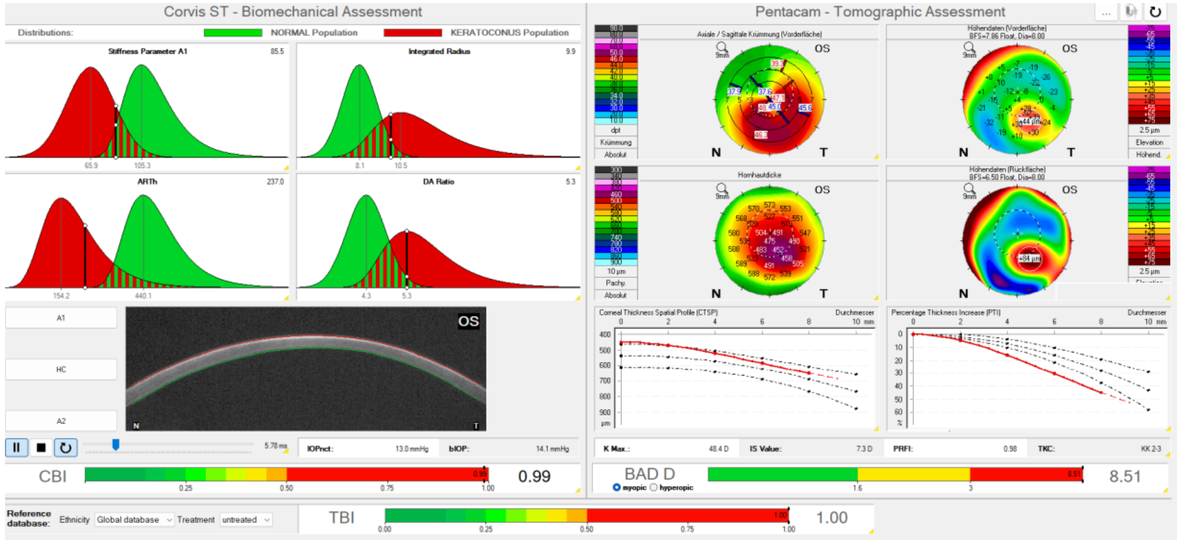
Corvis ST - Biomechanical Assessment
Pentacam - Tomographic Assessment
OS
CBI 0.99
BAD D 8.51
TBI 1.00

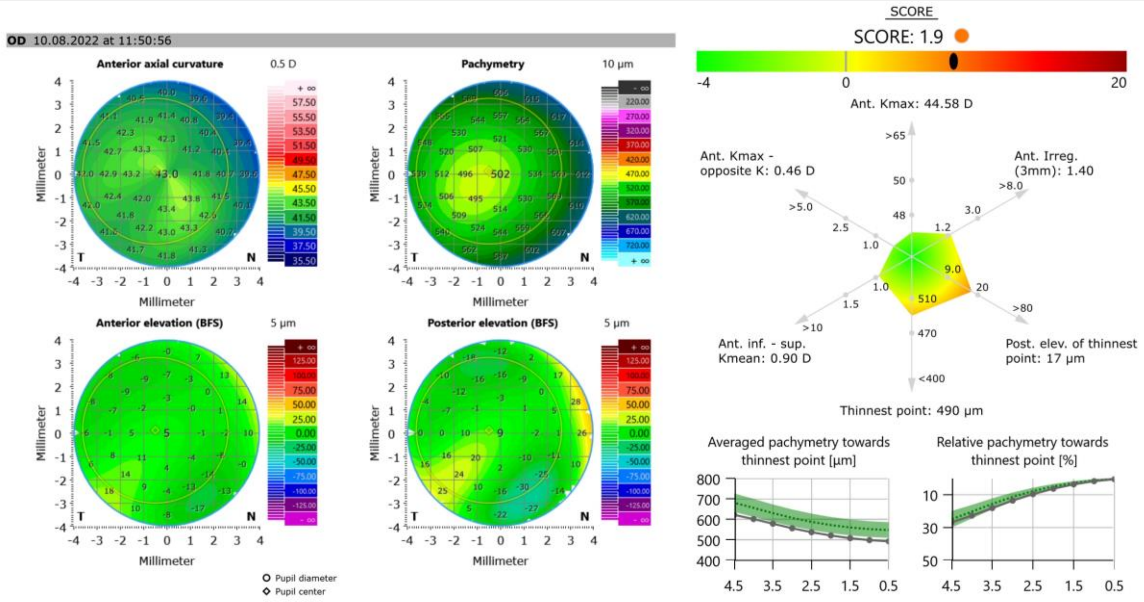
OD 10.08.2022 at 11:50:56
Anterior axial curvature
Pachymetry
Anterior elevation (BFS)
Posterior elevation (BFS)
Millimeter
SCORE
SCORE: 1.9
Ant. Kmax: 44.58 D
Ant. Kmax - opposite K: 0.46 D
Ant. Irreg. (3mm): 1.40
Ant. inf. - sup. Kmean: 0.90 D
Post. elev. of thinnest point: 17 µm
Thinnest point: 490 µm
Averaged pachymetry towards thinnest point [µm]
Relative pachymetry towards thinnest point [%]
Pupil diameter
Pupil center

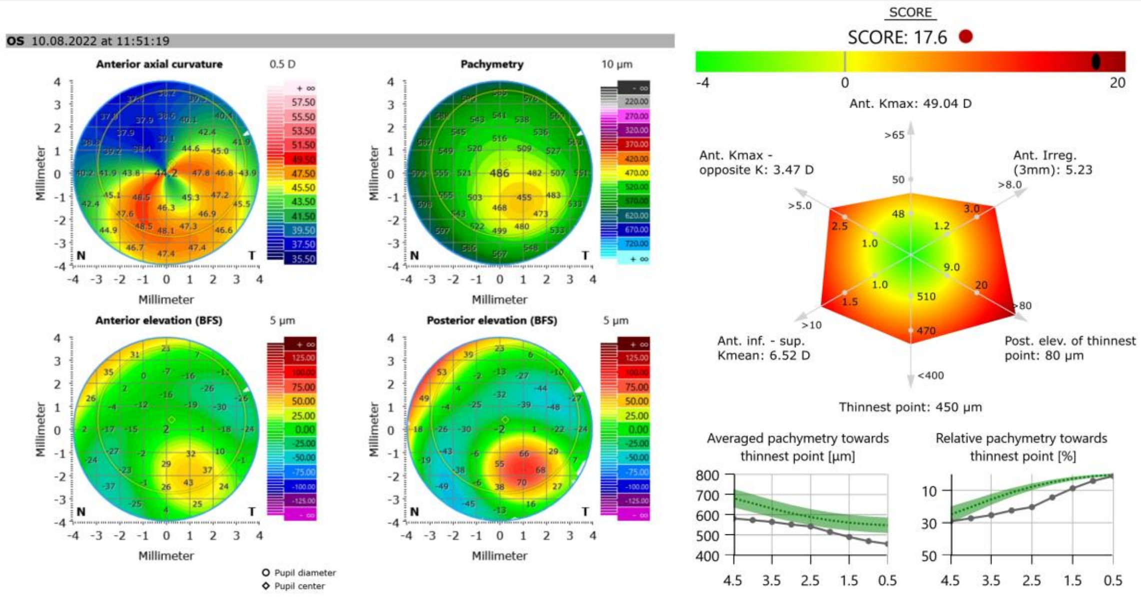
OS 10.08.2022 at 11:51:19
Anterior axial curvature
Pachymetry
Anterior elevation (BFS)
Posterior elevation (BFS)
Millimeter
SCORE
SCORE: 17.6
Ant. Kmax: 49.04 D
Ant. Kmax - opposite K: 3.47 D
Ant. Irreg. (3mm): 5.23
Ant. inf. - sup. Kmean: 6.52 D
Post. elev. of thinnest point: 80 µm
Thinnest point: 450 µm
Averaged pachymetry towards thinnest point [µm]
Relative pachymetry towards thinnest point [%]
Pupil diameter
Pupil center

CASE #35

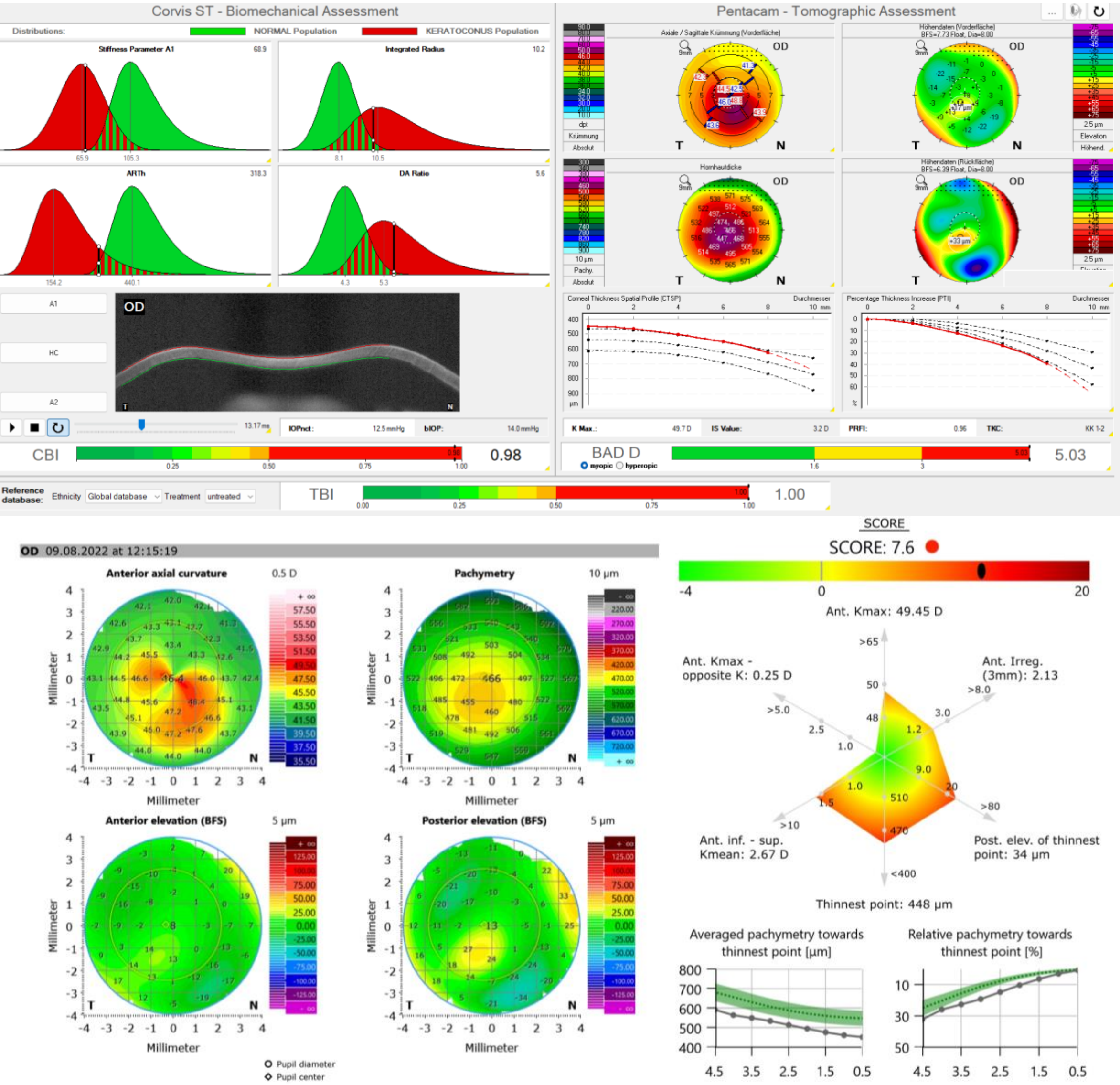

Corvis ST - Biomechanical Assessment
Pentacam - Tomographic Assessment
NORMAL Population
KERATOCONUS Population
OD
CBI 0.98
BAD D 5.03
TBI 1.00
OD 09.08.2022 at 12:15:19
Anterior axial curvature
Pachymetry
Anterior elevation (BFS)
Posterior elevation (BFS)
Millimeter
SCORE
SCORE: 7.6
Ant. Kmax: 49.45 D
Ant. Kmax - opposite K: 0.25 D
Ant. Irreg. (3mm): 2.13
Ant. inf. - sup. Kmean: 2.67 D
Post. elev. of thinnest point: 34 µm
Thinnest point: 448 µm
Averaged pachymetry towards thinnest point [µm]
Relative pachymetry towards thinnest point [%]
Pupil diameter
Pupil center

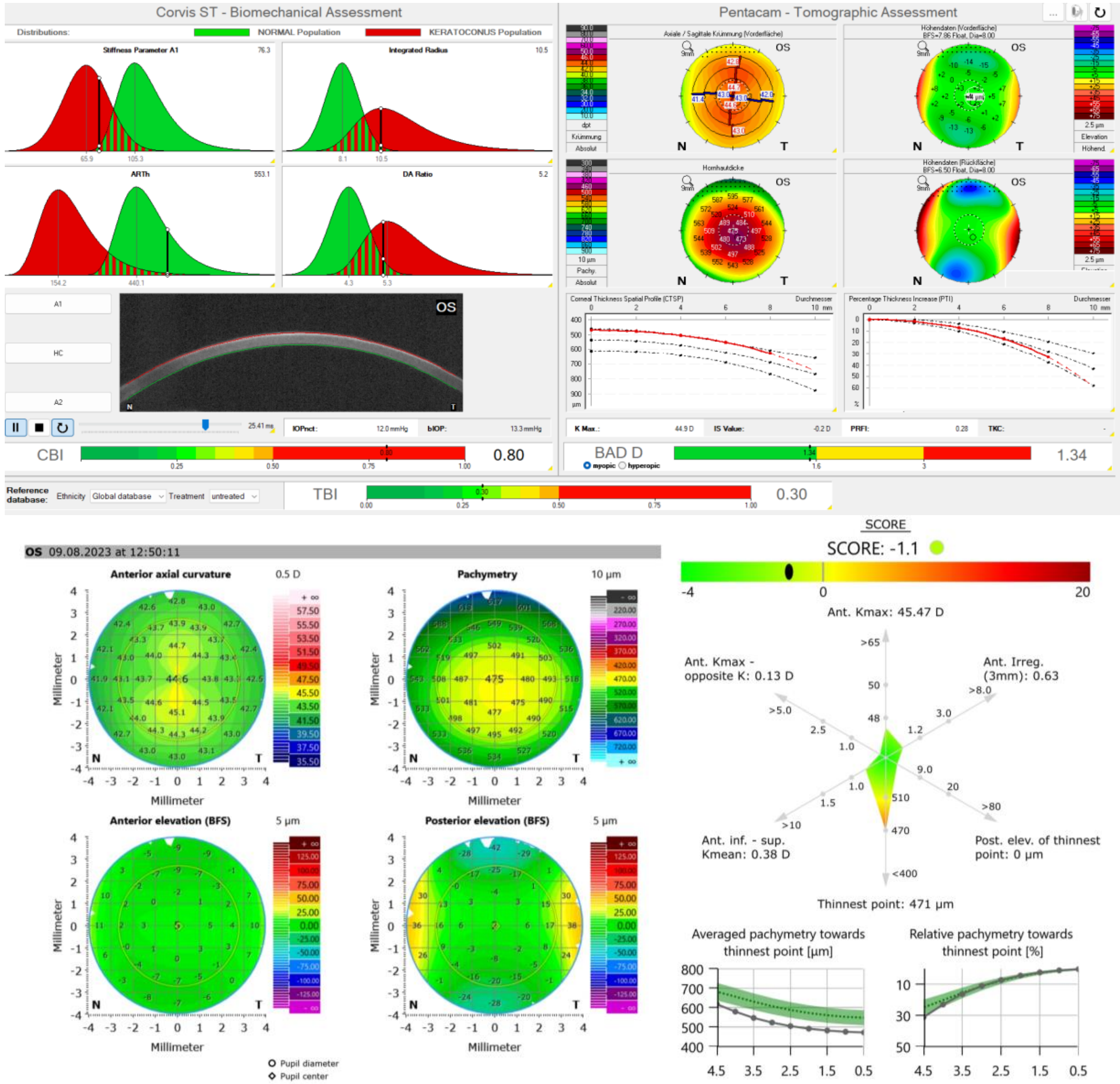

Corvis ST - Biomechanical Assessment
Pentacam - Tomographic Assessment
NORMAL Population
KERATOCONUS Population
OS
CBI 0.80
BAD D 1.34
TBI 0.30
OS 09.08.2023 at 12:50:11
Anterior axial curvature
Pachymetry
Anterior elevation (BFS)
Posterior elevation (BFS)
Millimeter
SCORE
SCORE: -1.1
Ant. Kmax: 45.47 D
Ant. Kmax - opposite K: 0.13 D
Ant. Irreg. (3mm): 0.63
Ant. inf. - sup. Kmean: 0.38 D
Post. elev. of thinnest point: 0 µm
Thinnest point: 471 µm
Averaged pachymetry towards thinnest point [µm]
Relative pachymetry towards thinnest point [%]
Pupil diameter
Pupil center

CASE #36

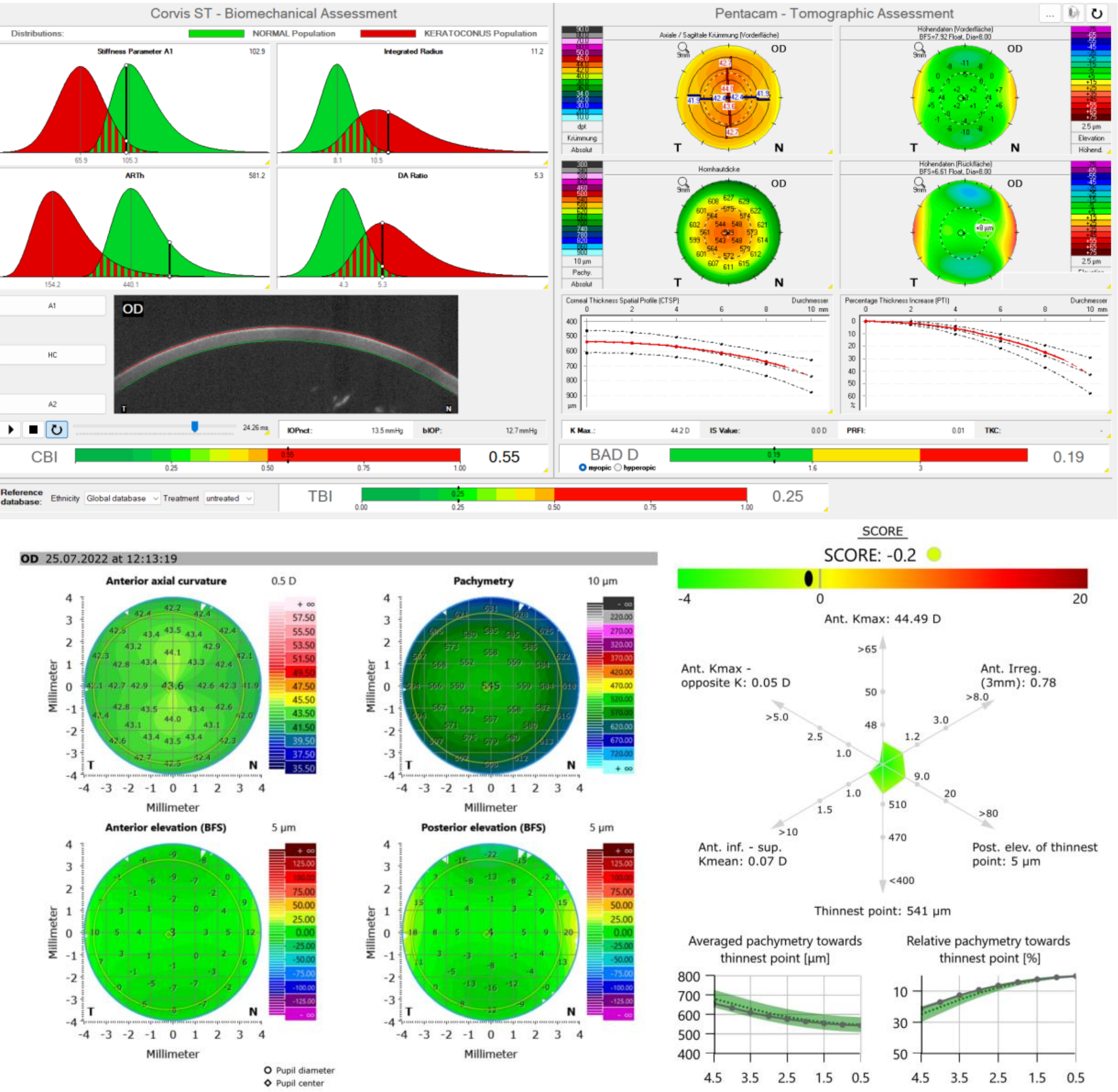

Excluded for analysis due to prior CXL

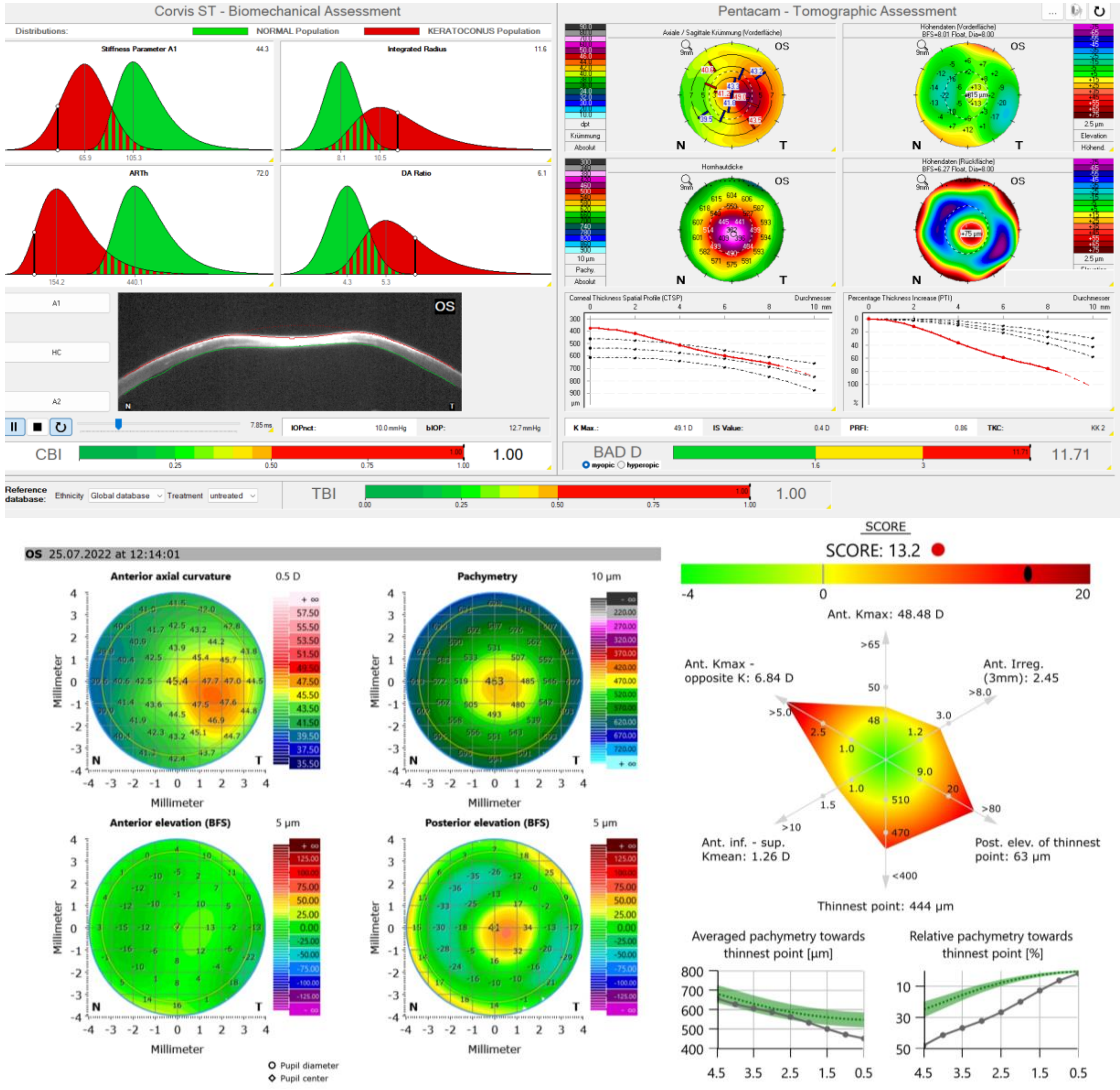

CASE #37

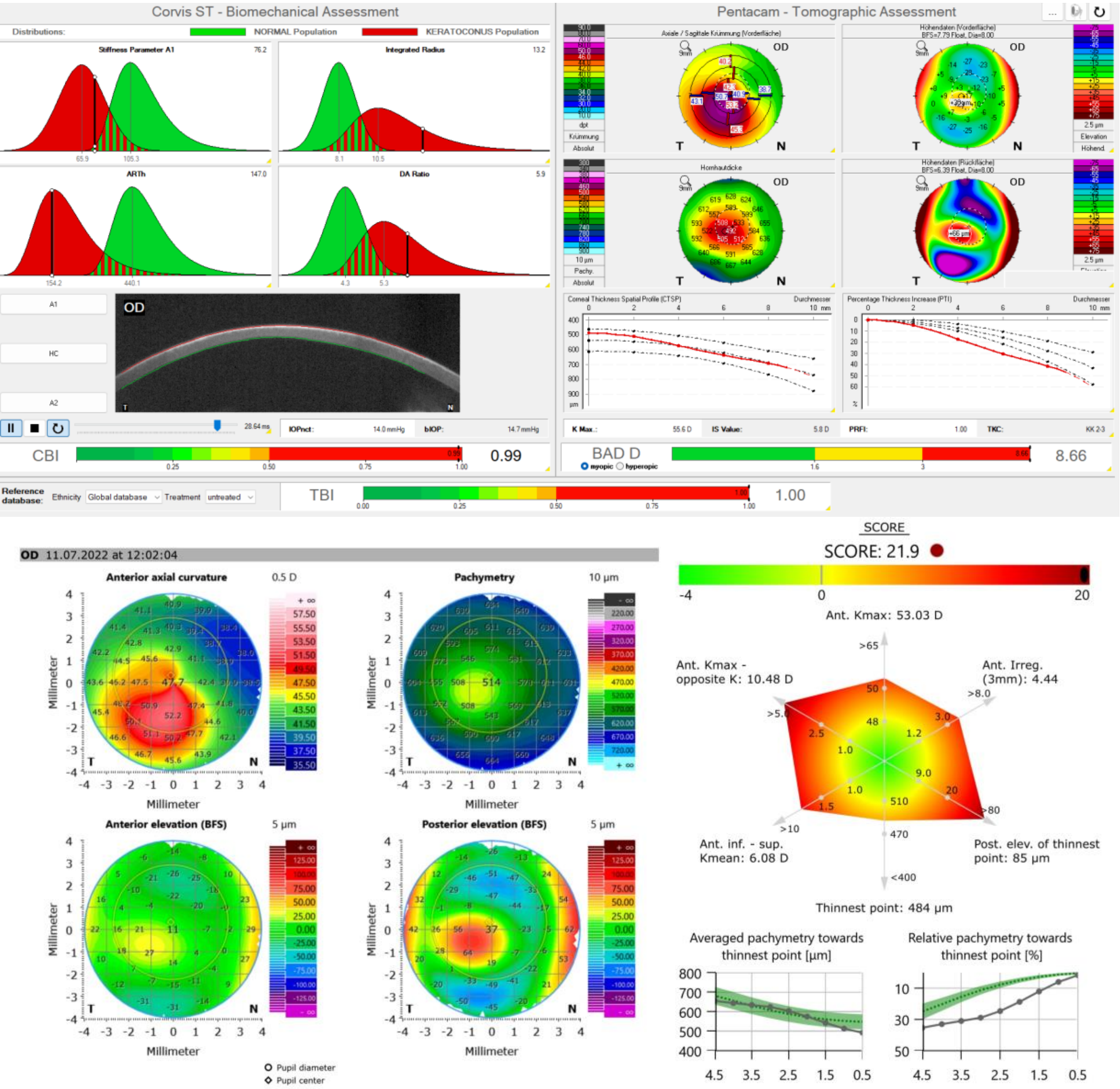

Corvis ST - Biomechanical Assessment
Pentacam - Tomographic Assessment
NORMAL Population
KERATOCONUS Population
Stiffness Parameter A1
Integrated Radius
ARTh
DA Ratio
OD
CBI
0.99
BAD D
8.66
TBI
1.00
SCORE
SCORE: 21.9
OD 11.07.2022 at 12:02:04
Anterior axial curvature
Pachymetry
Anterior elevation (BFS)
Posterior elevation (BFS)
Millimeter
Ant. Kmax: 53.03 D
Ant. Kmax - opposite K: 10.48 D
Ant. Irreg. (3mm): 4.44
Ant. inf. - sup. Kmean: 6.08 D
Post. elev. of thinnest point: 85 µm
Thinnest point: 484 µm
Averaged pachymetry towards thinnest point [µm]
Relative pachymetry towards thinnest point [%]
Pupil diameter
Pupil center

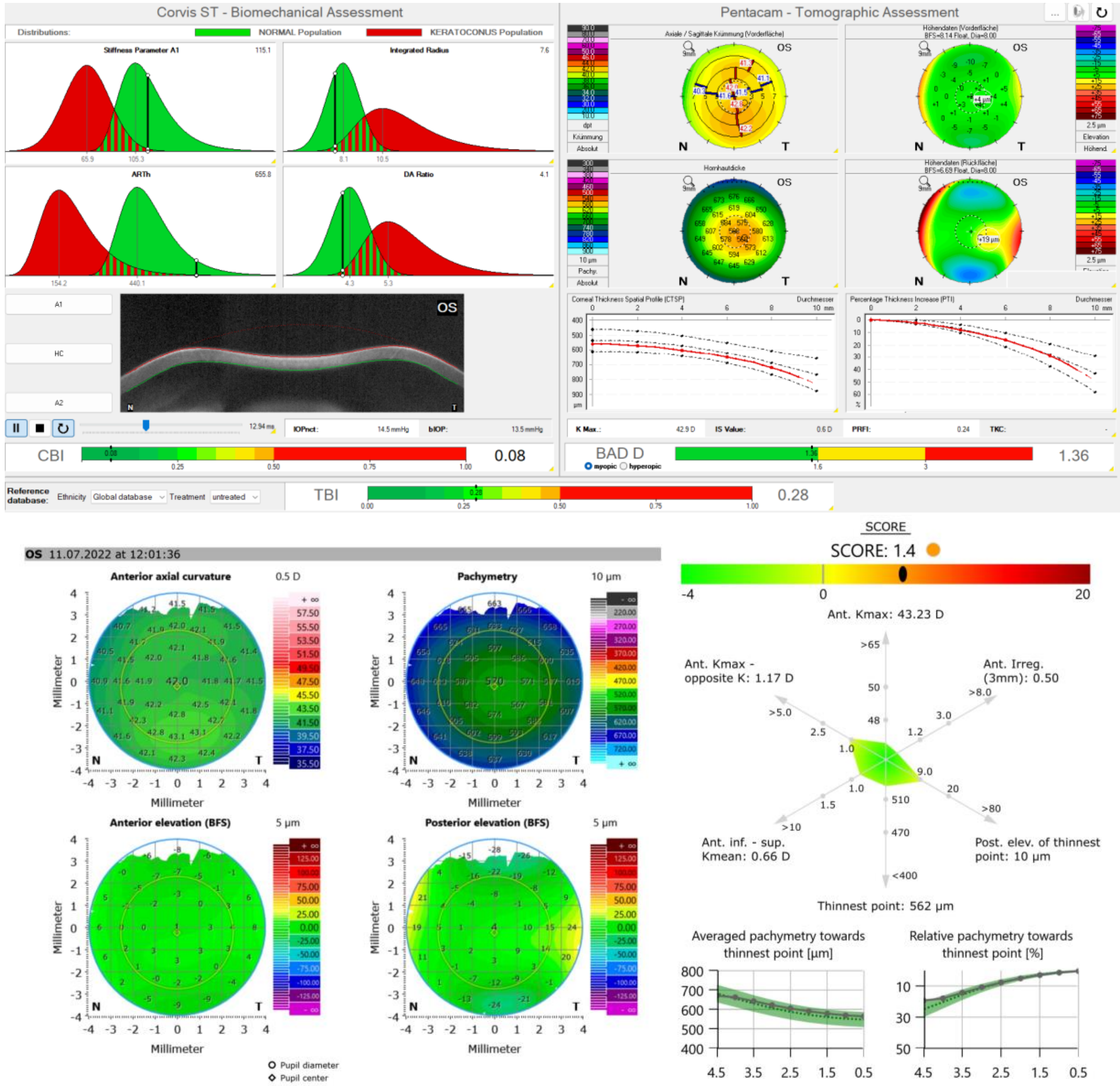

Corvis ST - Biomechanical Assessment
Pentacam - Tomographic Assessment
NORMAL Population
KERATOCONUS Population
Stiffness Parameter A1
Integrated Radius
ARTh
DA Ratio
OS
CBI
0.08
BAD D
1.36
TBI
0.28
SCORE
SCORE: 1.4
OS 11.07.2022 at 12:01:36
Anterior axial curvature
Pachymetry
Anterior elevation (BFS)
Posterior elevation (BFS)
Millimeter
Ant. Kmax: 43.23 D
Ant. Kmax - opposite K: 1.17 D
Ant. Irreg. (3mm): 0.50
Ant. inf. - sup. Kmean: 0.66 D
Post. elev. of thinnest point: 10 µm
Thinnest point: 562 µm
Averaged pachymetry towards thinnest point [µm]
Relative pachymetry towards thinnest point [%]
Pupil diameter
Pupil center

CASE #38

Excluded for analysis due to prior CXL

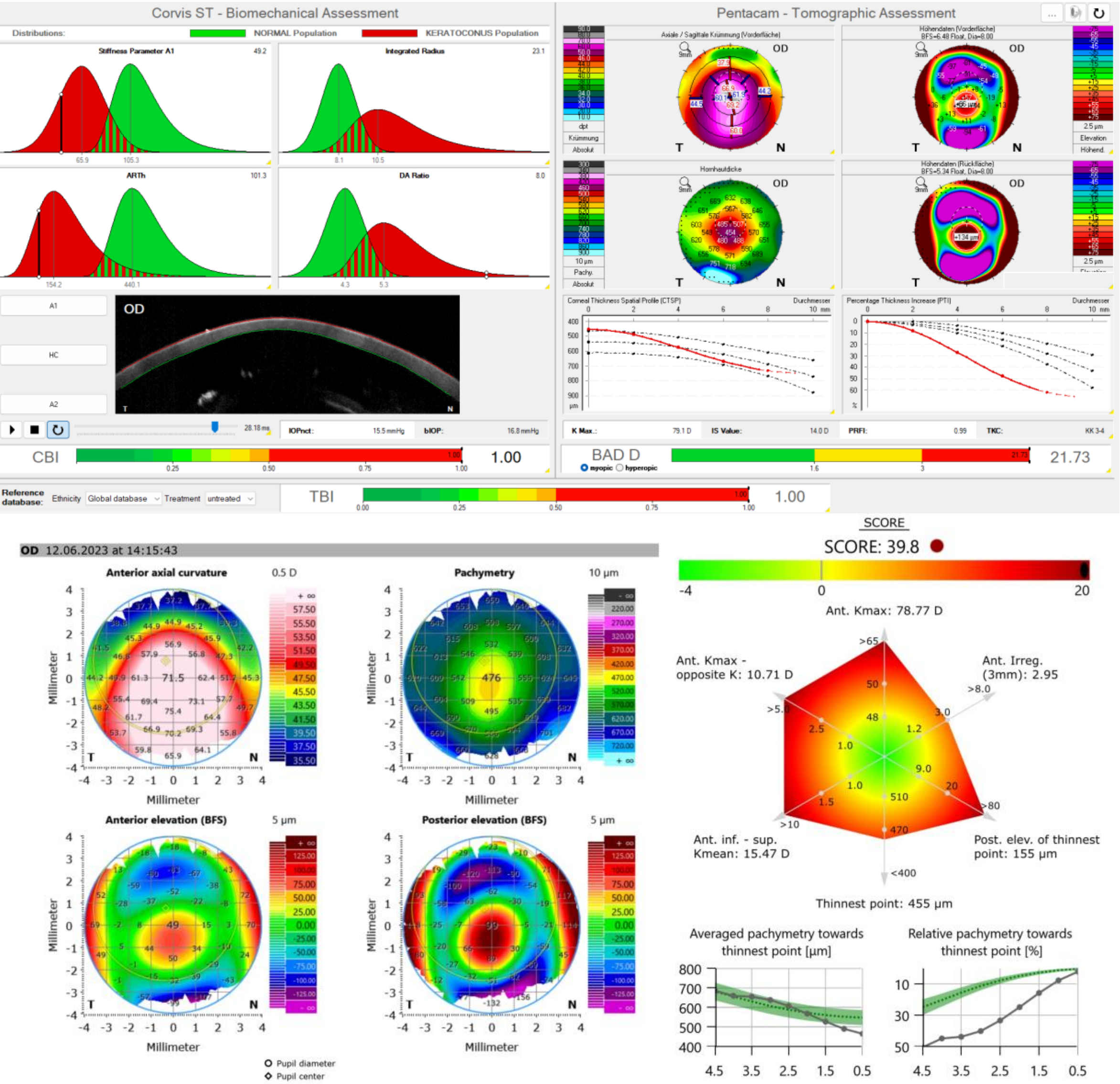

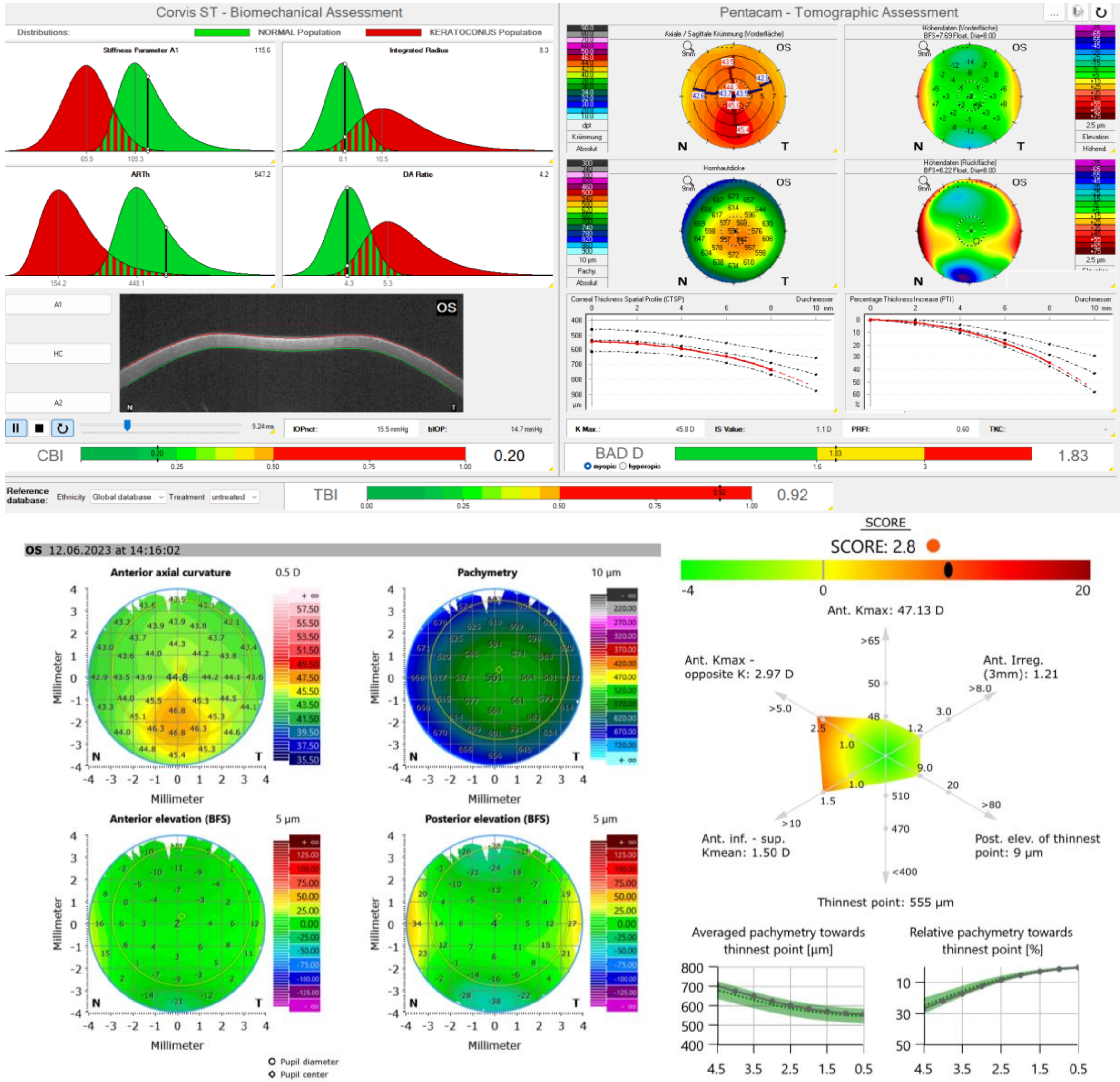

CASE #39

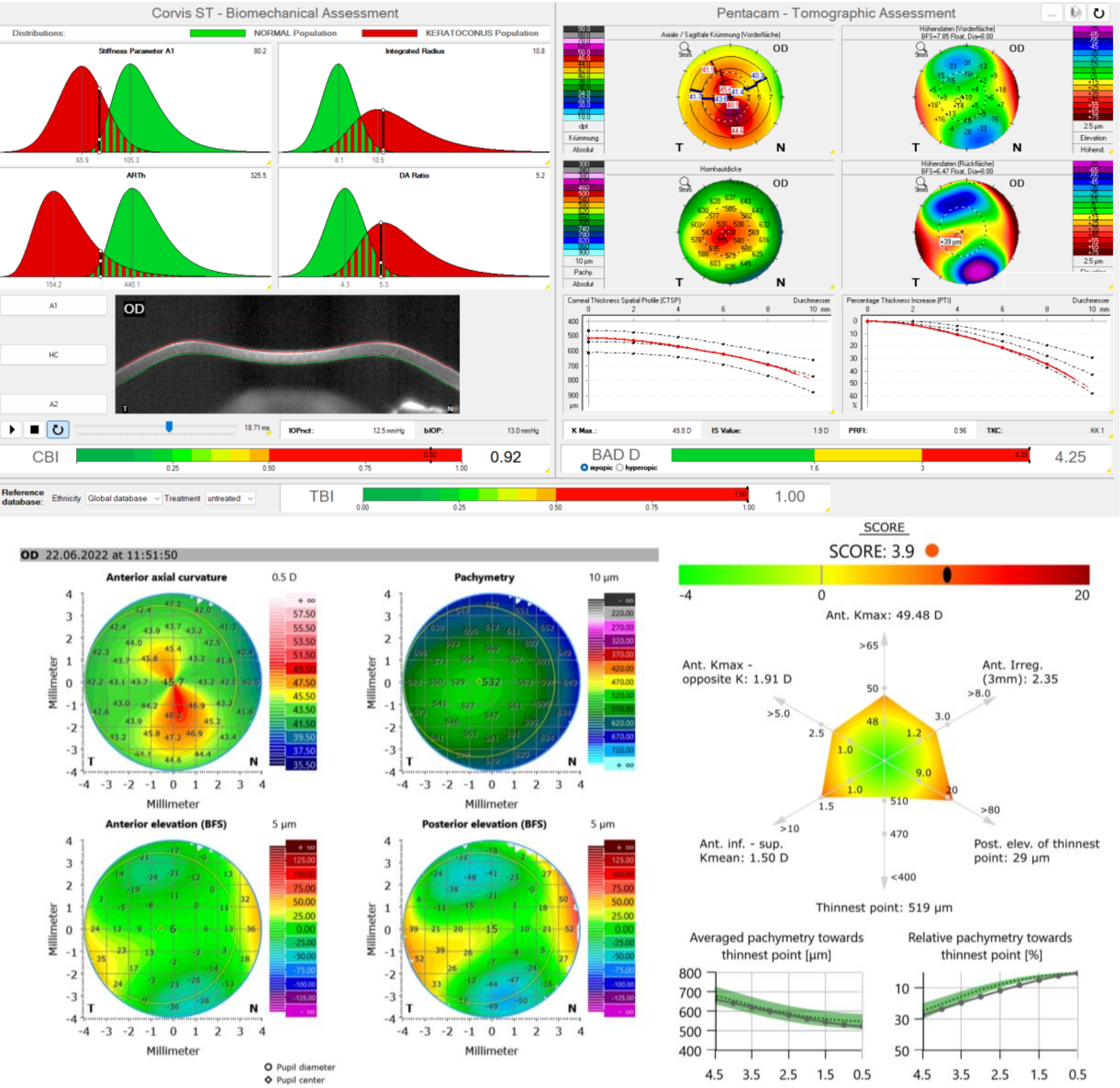

Corvis ST - Biomechanical Assessment
NORMAL Population
KERATOCONUS Population
Stiffness Parameter A1
Integrated Radius
ARTh
DA Ratio
OD
CBI
0.92
Pentacam - Tomographic Assessment
BAD D
4.25
TBI
1.00
OD 22.06.2022 at 11:51:50
Anterior axial curvature
Pachymetry
Anterior elevation (BFS)
Posterior elevation (BFS)
Millimeter
SCORE
SCORE: 3.9
Ant. Kmax: 49.48 D
Ant. Kmax - opposite K: 1.91 D
Ant. Irreg. (3mm): 2.35
Ant. inf. - sup. Kmean: 1.50 D
Post. elev. of thinnest point: 29 µm
Thinnest point: 519 µm
Averaged pachymetry towards thinnest point [µm]
Relative pachymetry towards thinnest point [%]
Pupil diameter
Pupil center

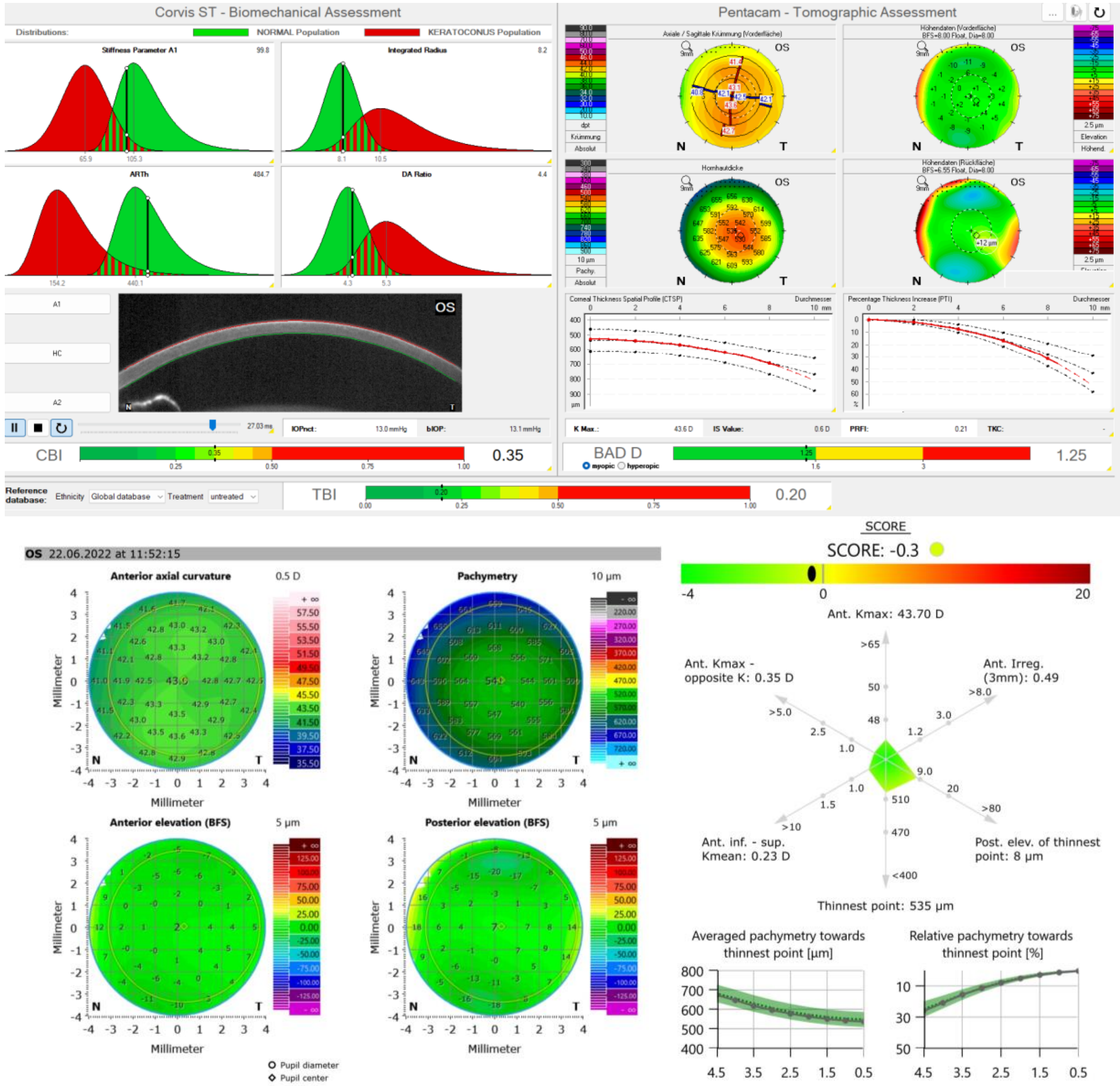

Corvis ST - Biomechanical Assessment
NORMAL Population
KERATOCONUS Population
Stiffness Parameter A1
Integrated Radius
ARTh
DA Ratio
OS
CBI
0.35
Pentacam - Tomographic Assessment
BAD D
1.25
TBI
0.20
OS 22.06.2022 at 11:52:15
Anterior axial curvature
Pachymetry
Anterior elevation (BFS)
Posterior elevation (BFS)
Millimeter
SCORE
SCORE: -0.3
Ant. Kmax: 43.70 D
Ant. Kmax - opposite K: 0.35 D
Ant. Irreg. (3mm): 0.49
Ant. inf. - sup. Kmean: 0.23 D
Post. elev. of thinnest point: 8 µm
Thinnest point: 535 µm
Averaged pachymetry towards thinnest point [µm]
Relative pachymetry towards thinnest point [%]
Pupil diameter
Pupil center

CASE #40

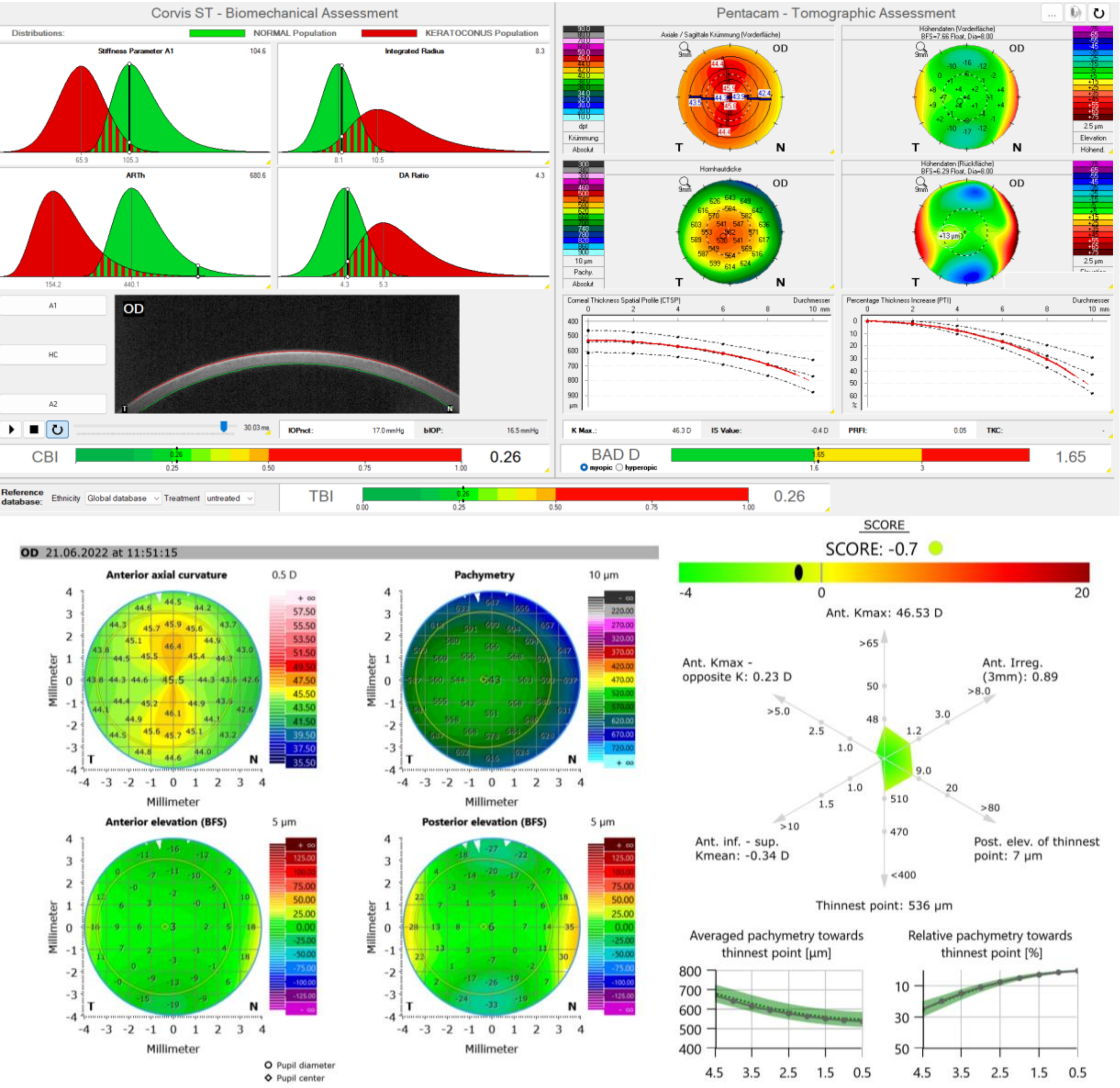
Corvis ST - Biomechanical Assessment
Pentacam - Tomographic Assessment
CBI 0.26
BAD D 1.65
TBI 0.26
OD 21.06.2022 at 11:51:15
Anterior axial curvature
Pachymetry
Anterior elevation (BFS)
Posterior elevation (BFS)
SCORE
SCORE: -0.7
Ant. Kmax: 46.53 D
Ant. Kmax - opposite K: 0.23 D
Ant. Irreg. (3mm): 0.89
Ant. inf. - sup. Kmean: -0.34 D
Post. elev. of thinnest point: 7 μm
Thinnest point: 536 μm
Averaged pachymetry towards thinnest point [μm]
Relative pachymetry towards thinnest point [%]

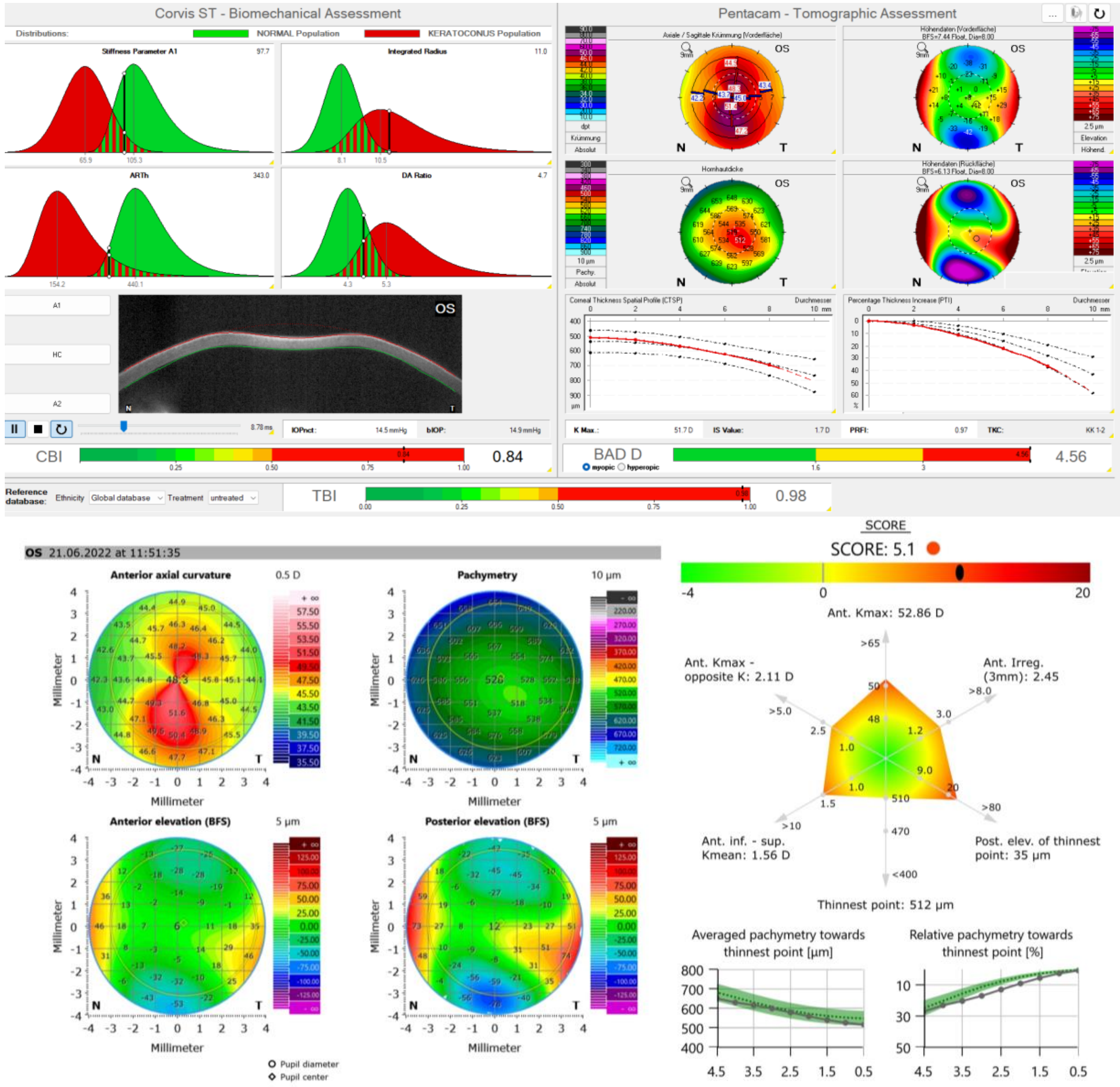
Corvis ST - Biomechanical Assessment
Pentacam - Tomographic Assessment
CBI 0.84
BAD D 4.56
TBI 0.98
OS 21.06.2022 at 11:51:35
Anterior axial curvature
Pachymetry
Anterior elevation (BFS)
Posterior elevation (BFS)
SCORE
SCORE: 5.1
Ant. Kmax: 52.86 D
Ant. Kmax - opposite K: 2.11 D
Ant. Irreg. (3mm): 2.45
Ant. inf. - sup. Kmean: 1.56 D
Post. elev. of thinnest point: 35 μm
Thinnest point: 512 μm
Averaged pachymetry towards thinnest point [μm]
Relative pachymetry towards thinnest point [%]

CASE #41

Excluded for analysis due to prior CXL

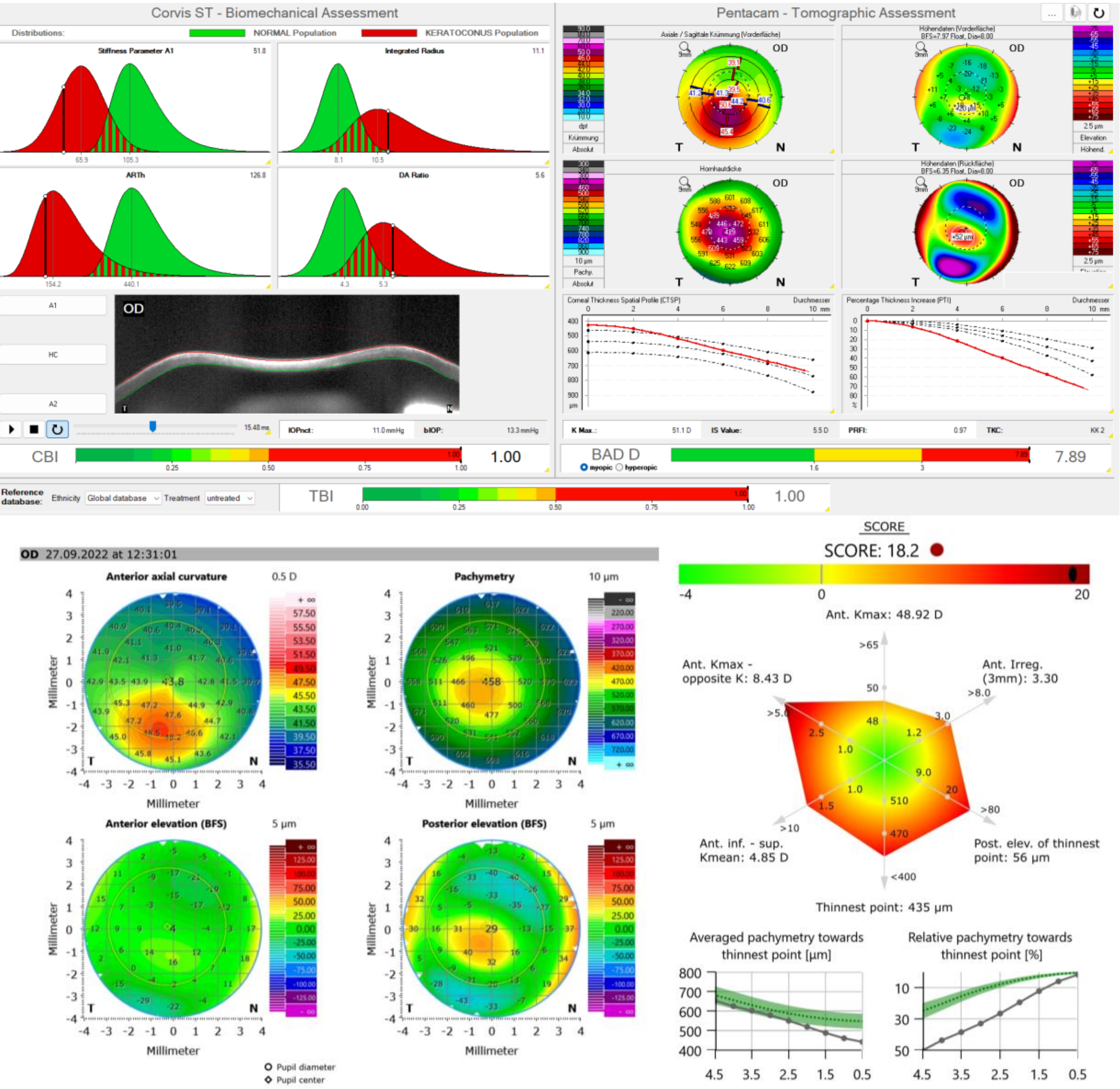

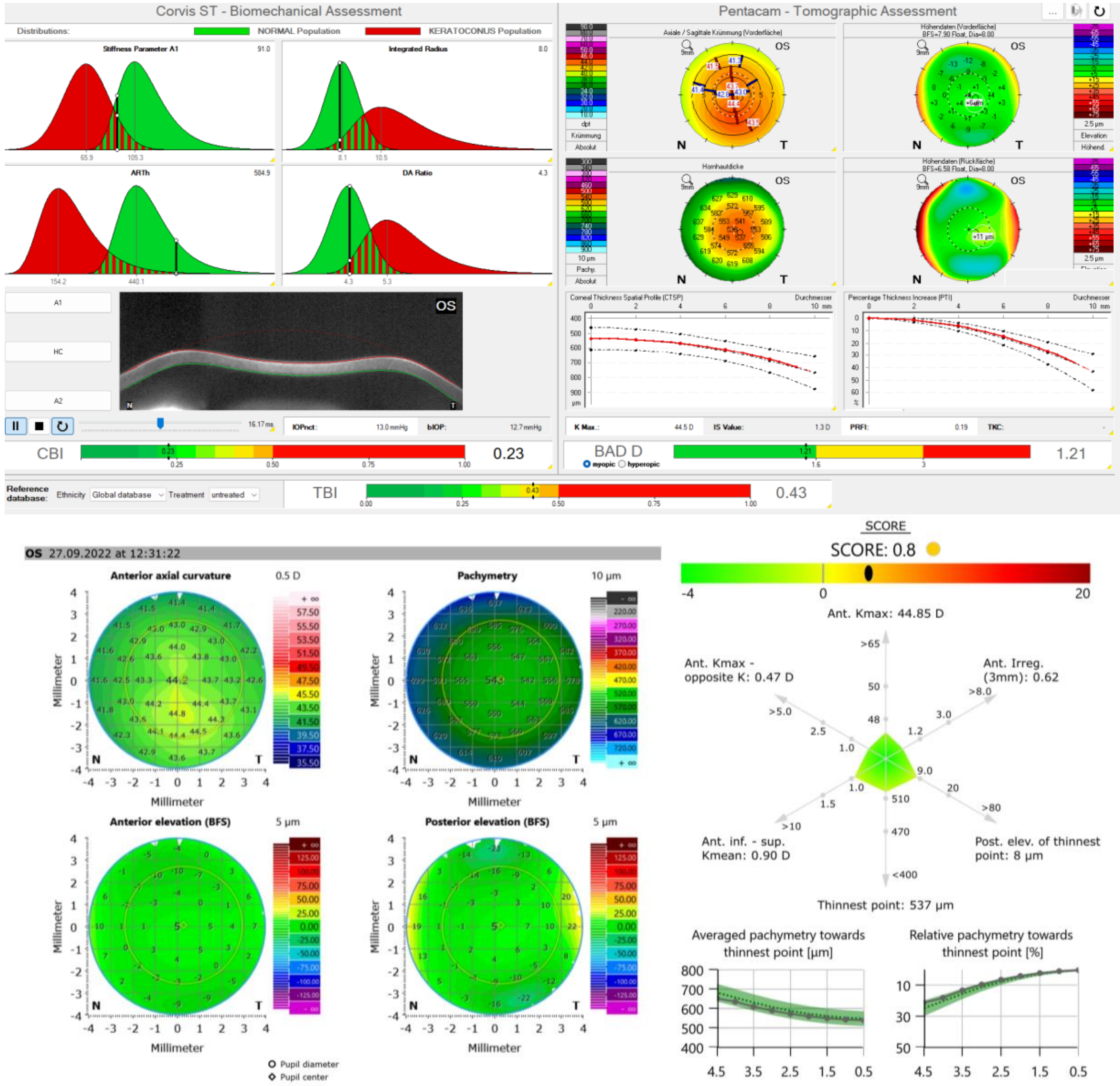

CASE #42

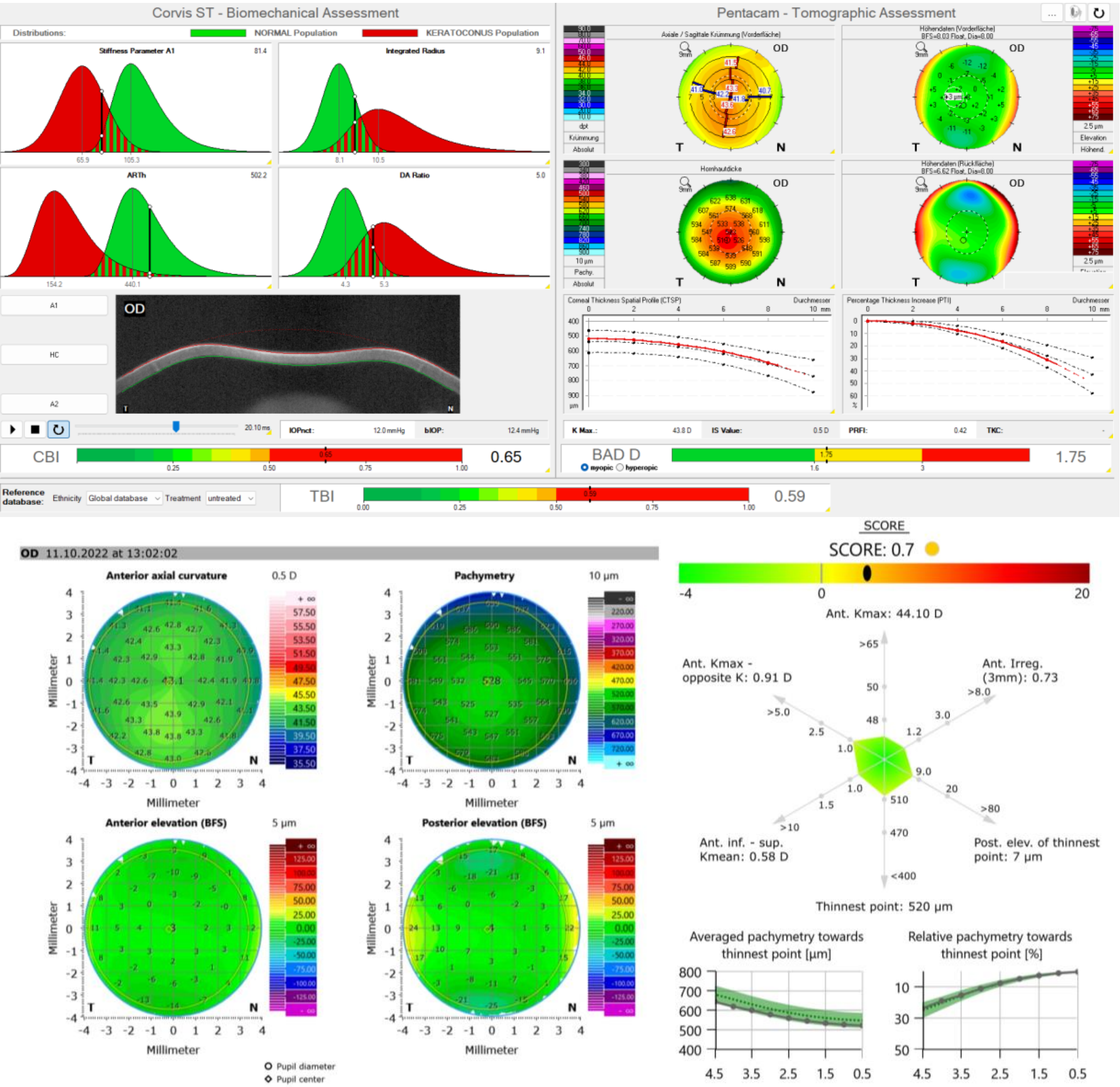

Excluded for analysis due to prior CXL

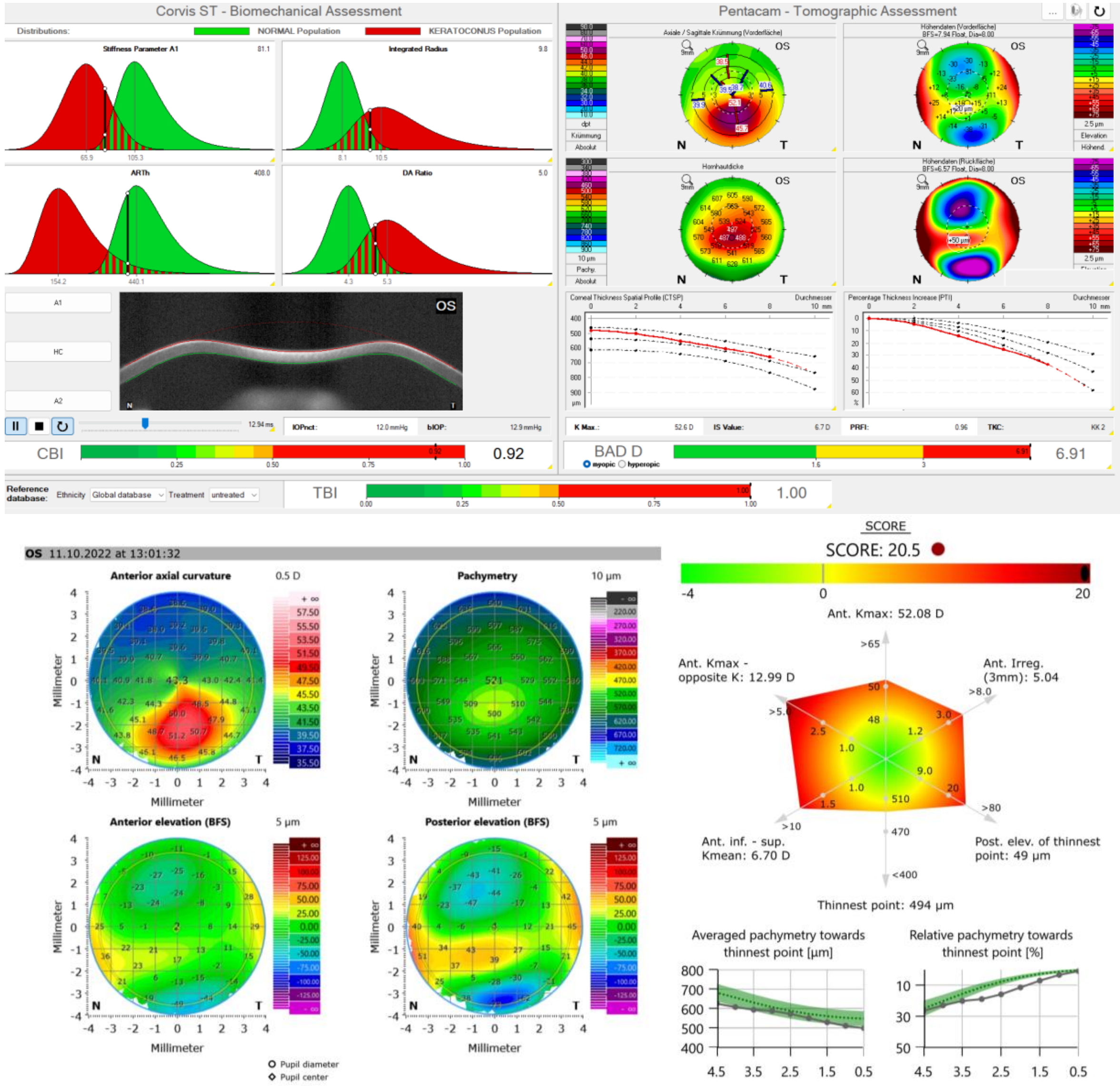

CASE #43

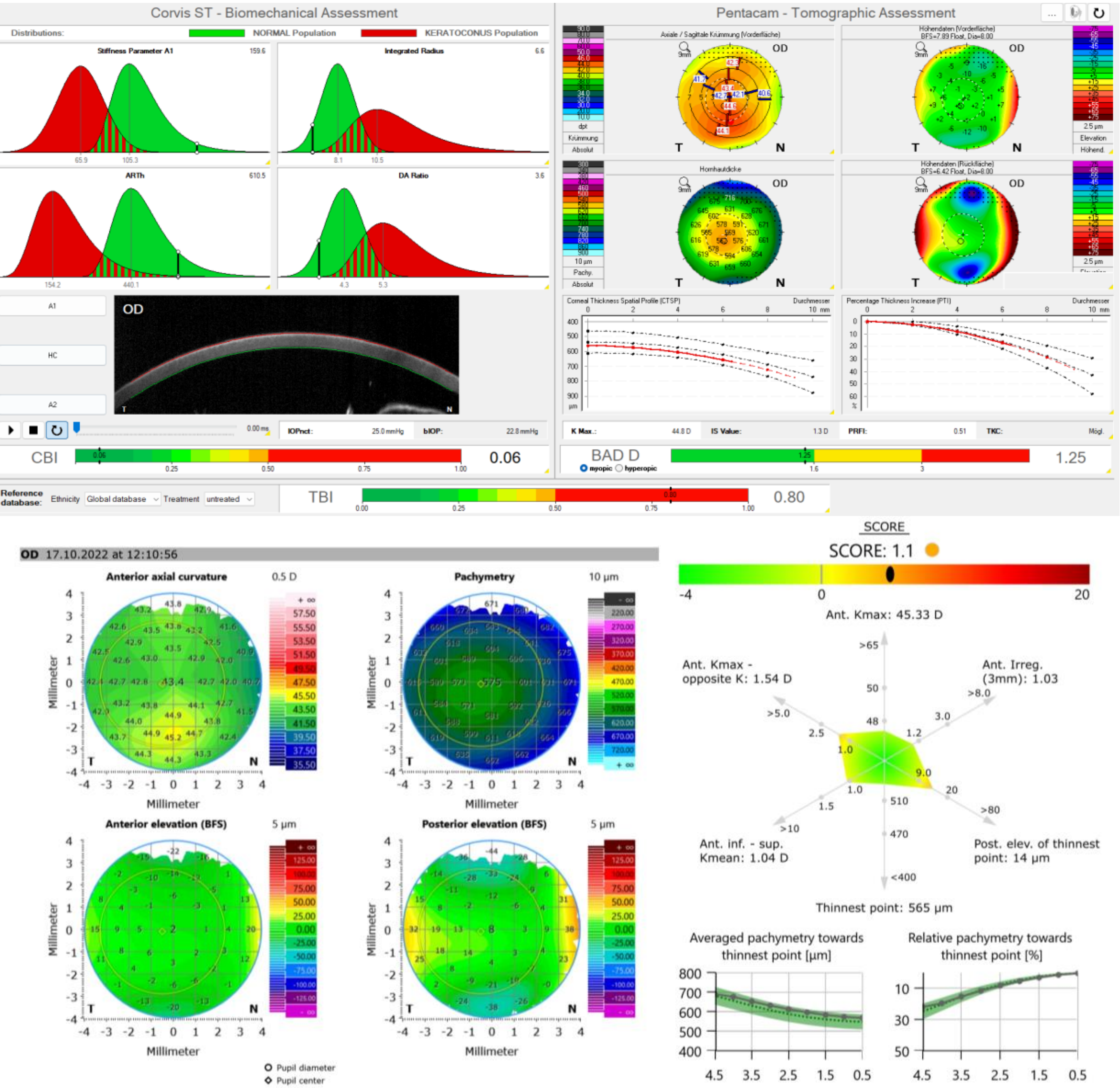
Corvis ST - Biomechanical Assessment
Pentacam - Tomographic Assessment
NORMAL Population
KERATOCONUS Population
Stiffness Parameter A1
Integrated Radius
ARTh
DA Ratio
OD
CBI
0.06
BAD D
1.25
TBI
0.80
OD 17.10.2022 at 12:10:56
Anterior axial curvature
Pachymetry
Anterior elevation (BFS)
Posterior elevation (BFS)
Millimeter
Pupil diameter
Pupil center
SCORE
SCORE: 1.1
Ant. Kmax: 45.33 D
Ant. Kmax - opposite K: 1.54 D
Ant. Irreg. (3mm): 1.03
Ant. inf. - sup. Kmean: 1.04 D
Post. elev. of thinnest point: 14 µm
Thinnest point: 565 µm
Averaged pachymetry towards thinnest point [µm]
Relative pachymetry towards thinnest point [%]

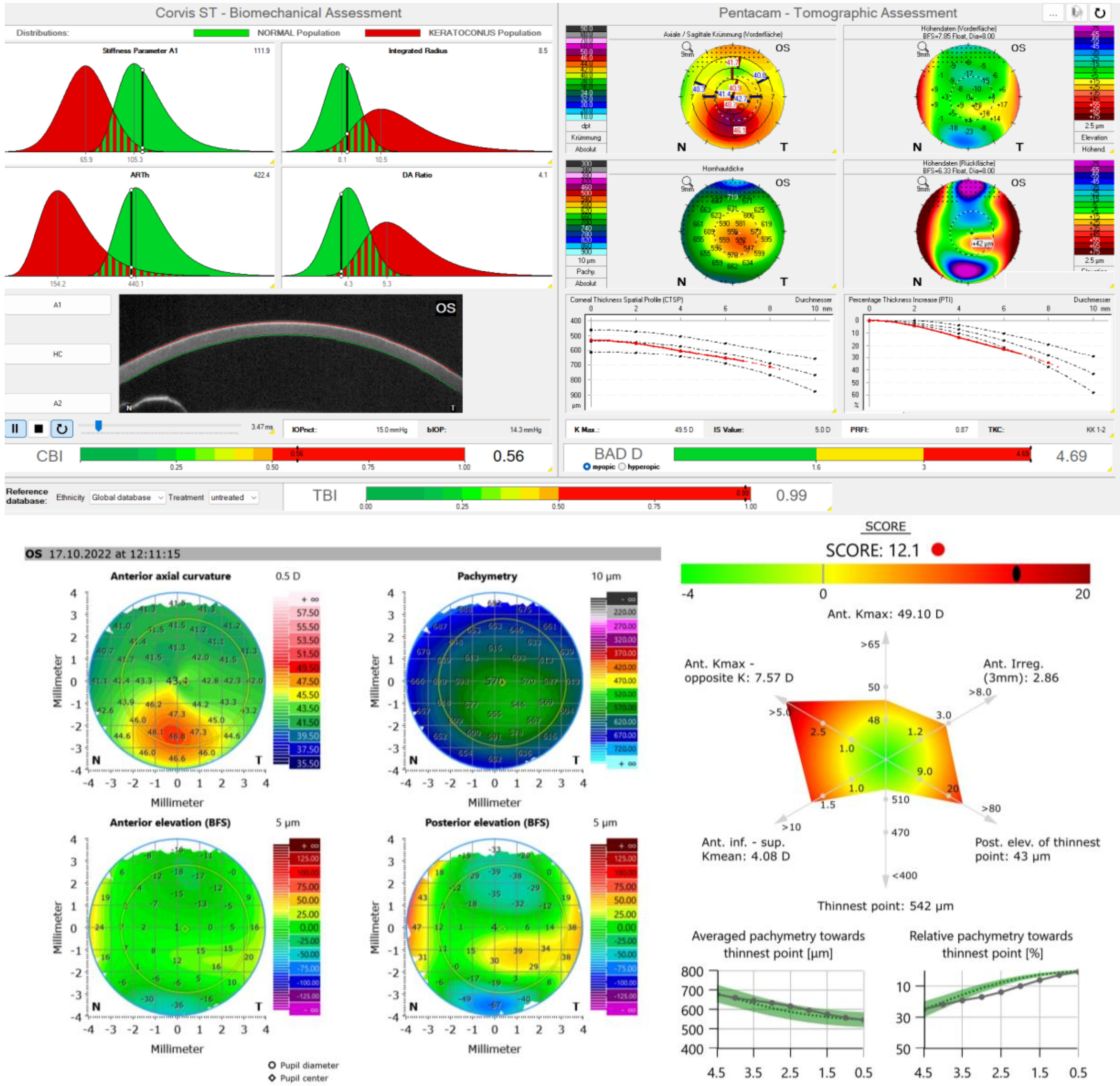
Corvis ST - Biomechanical Assessment
Pentacam - Tomographic Assessment
NORMAL Population
KERATOCONUS Population
Stiffness Parameter A1
Integrated Radius
ARTh
DA Ratio
OS
CBI
0.56
BAD D
4.69
TBI
0.99
OS 17.10.2022 at 12:11:15
Anterior axial curvature
Pachymetry
Anterior elevation (BFS)
Posterior elevation (BFS)
Millimeter
Pupil diameter
Pupil center
SCORE
SCORE: 12.1
Ant. Kmax: 49.10 D
Ant. Kmax - opposite K: 7.57 D
Ant. Irreg. (3mm): 2.86
Ant. inf. - sup. Kmean: 4.08 D
Post. elev. of thinnest point: 43 µm
Thinnest point: 542 µm
Averaged pachymetry towards thinnest point [µm]
Relative pachymetry towards thinnest point [%]

CASE #44

Excluded for analysis due to prior CXL

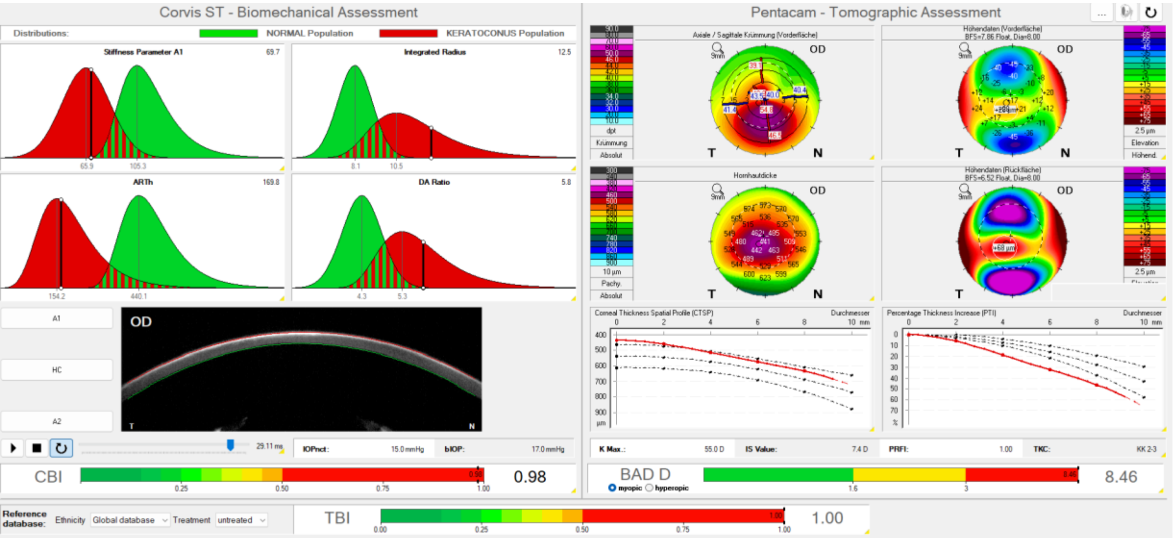

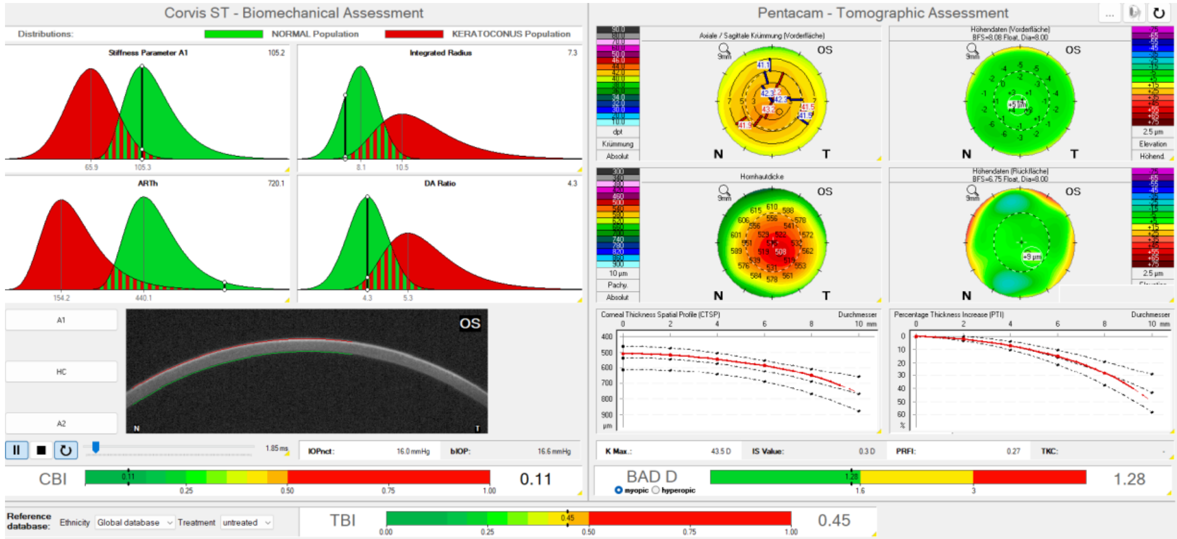

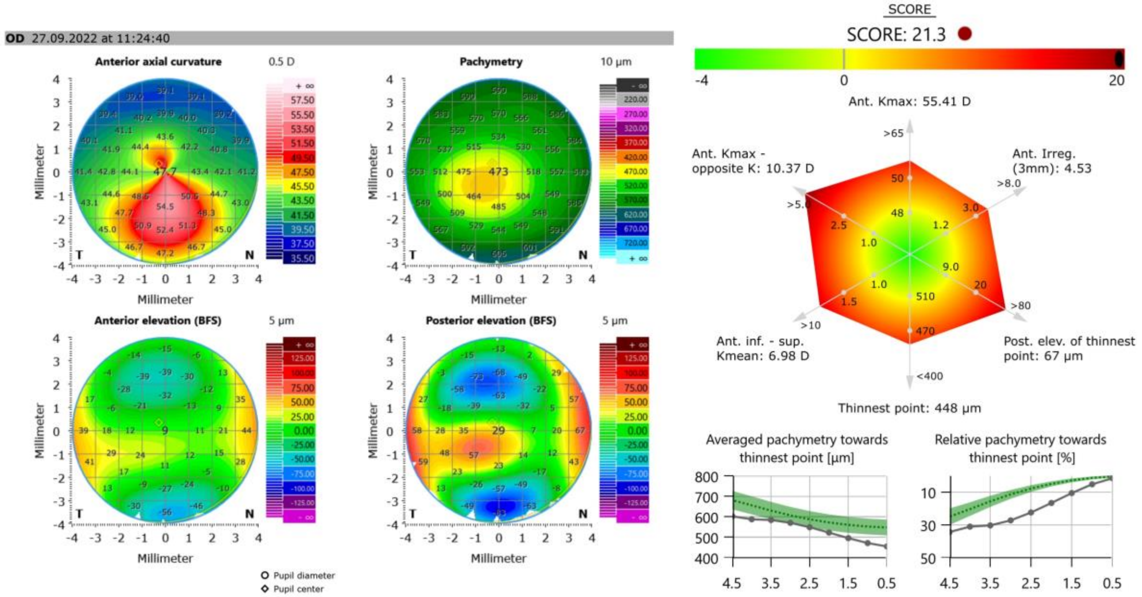

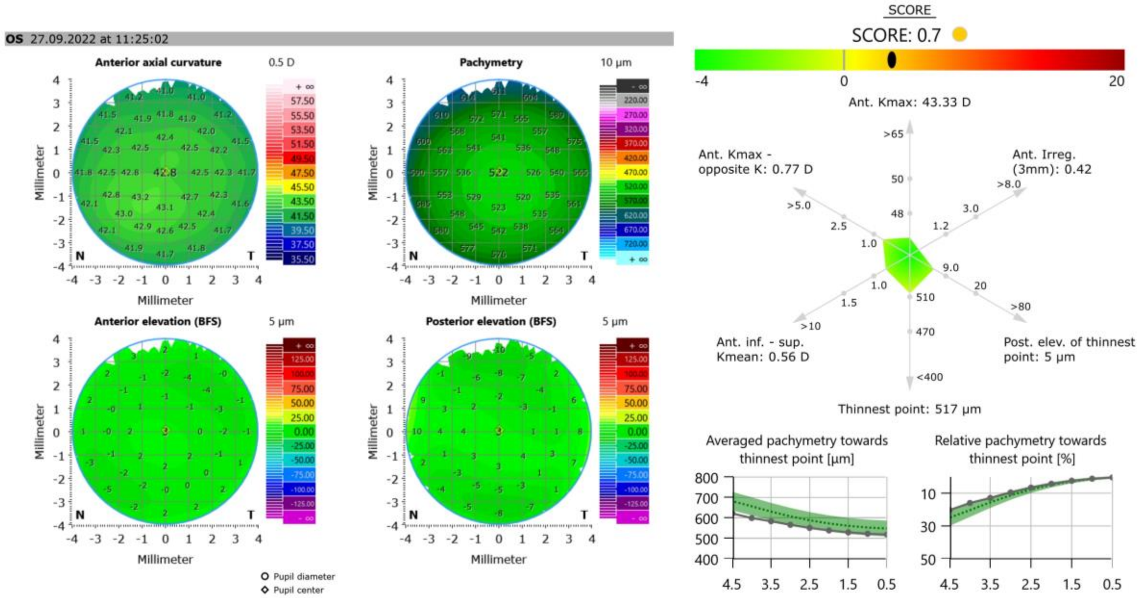

CASE #45

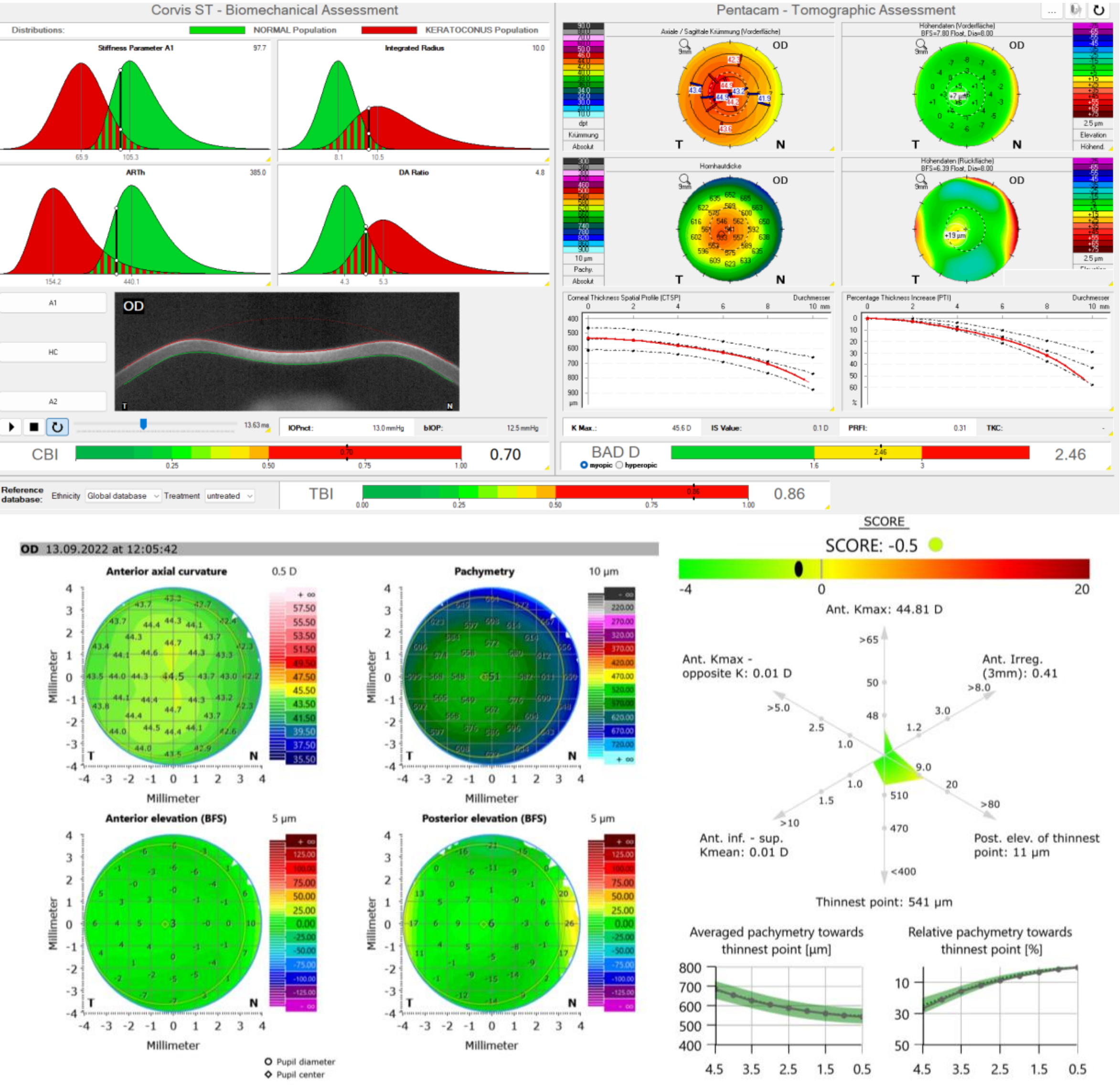

Corvis ST - Biomechanical Assessment
Pentacam - Tomographic Assessment
CBI 0.70
BAD D 2.46
TBI 0.86
SCORE
SCORE: -0.5
Ant. Kmax: 44.81 D
Ant. Kmax - opposite K: 0.01 D
Ant. Irreg. (3mm): 0.41
Ant. inf. - sup. Kmean: 0.01 D
Post. elev. of thinnest point: 11 µm
Thinnest point: 541 µm
OD 13.09.2022 at 12:05:42
Anterior axial curvature
Pachymetry
Anterior elevation (BFS)
Posterior elevation (BFS)
Averaged pachymetry towards thinnest point [µm]
Relative pachymetry towards thinnest point [%]

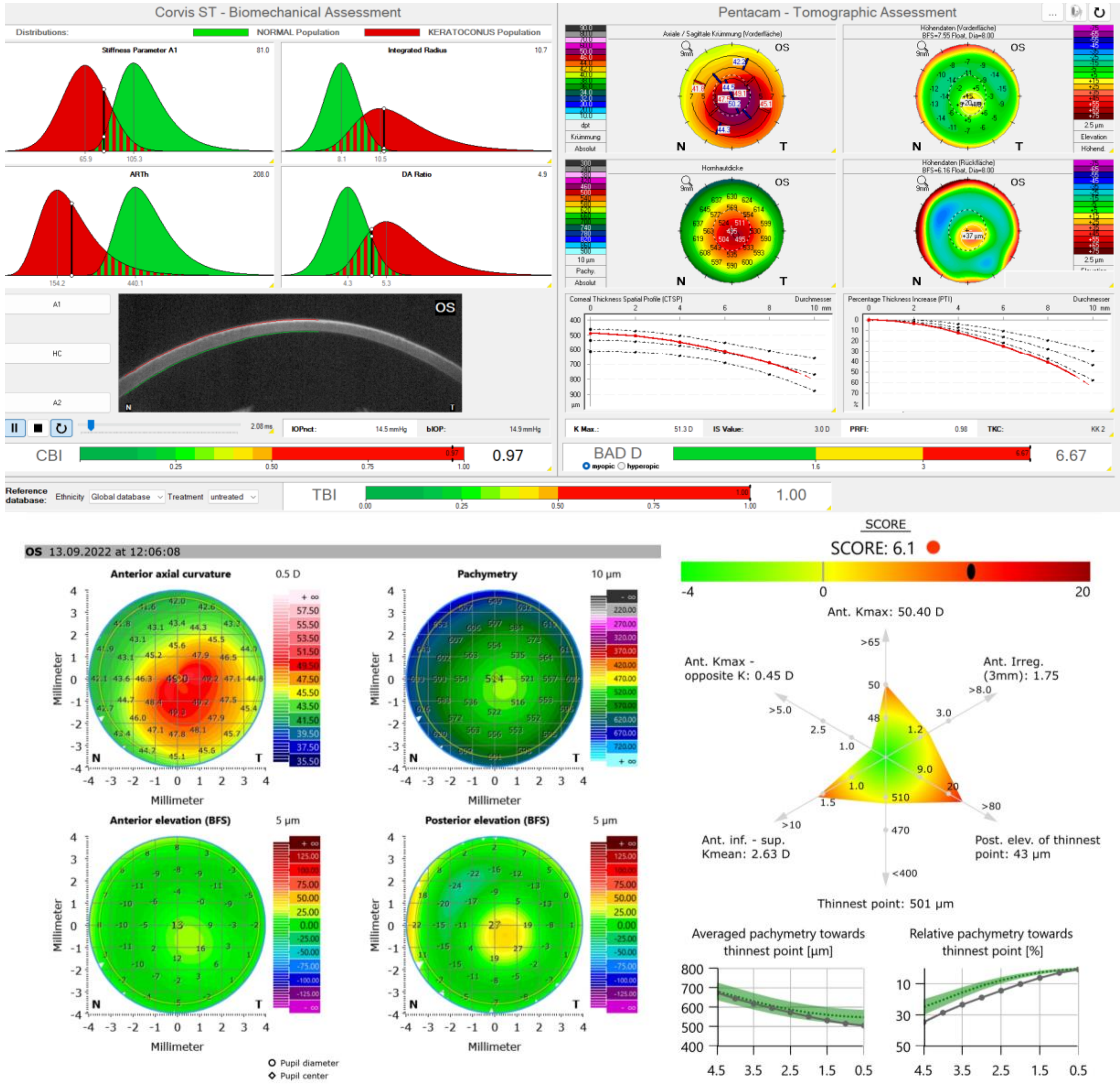

Corvis ST - Biomechanical Assessment
Pentacam - Tomographic Assessment
CBI 0.97
BAD D 6.67
TBI 1.00
SCORE
SCORE: 6.1
Ant. Kmax: 50.40 D
Ant. Kmax - opposite K: 0.45 D
Ant. Irreg. (3mm): 1.75
Ant. inf. - sup. Kmean: 2.63 D
Post. elev. of thinnest point: 43 µm
Thinnest point: 501 µm
OS 13.09.2022 at 12:06:08
Anterior axial curvature
Pachymetry
Anterior elevation (BFS)
Posterior elevation (BFS)
Averaged pachymetry towards thinnest point [µm]
Relative pachymetry towards thinnest point [%]

CASE #46

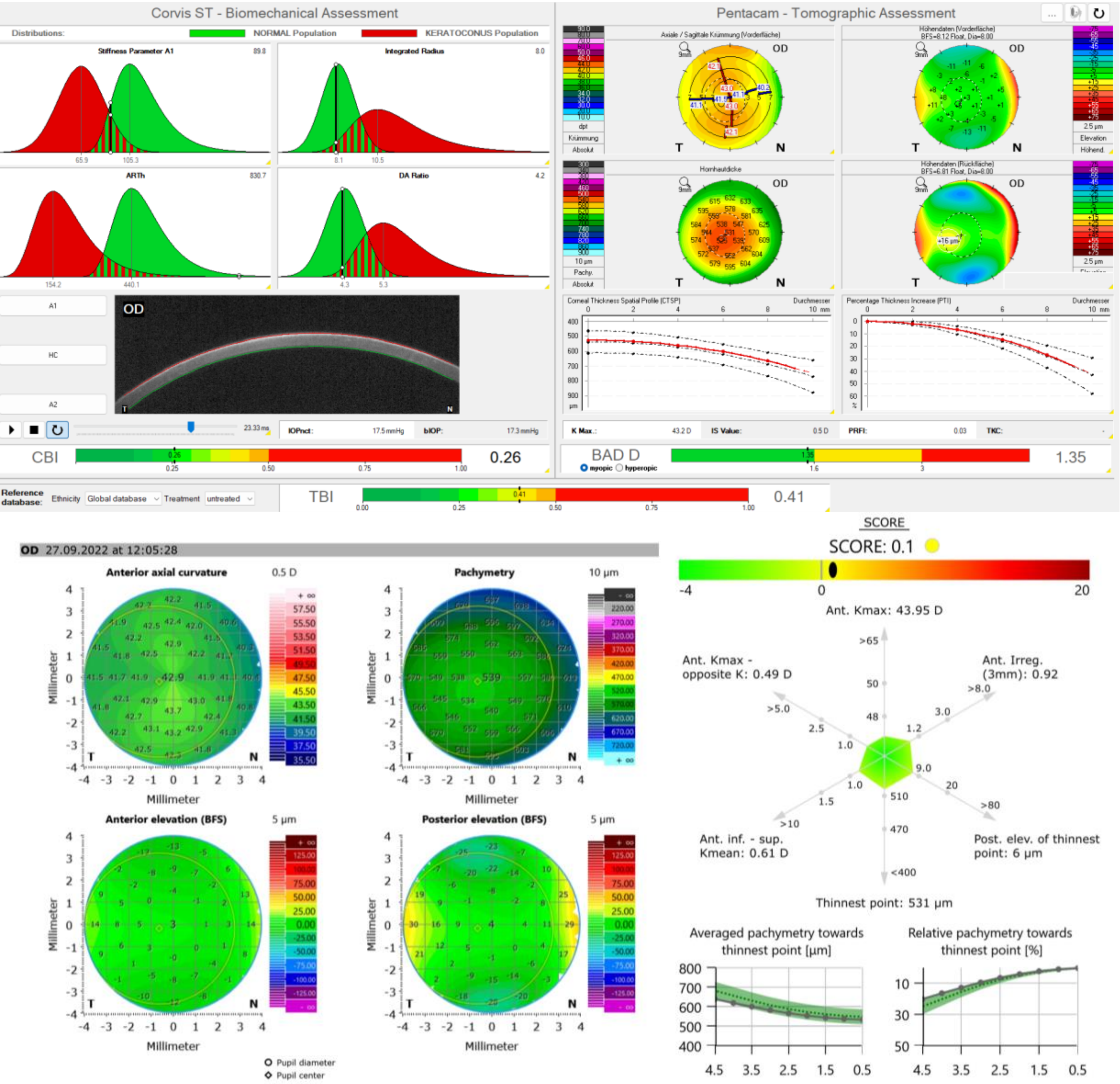
Corvis ST - Biomechanical Assessment
Pentacam - Tomographic Assessment
NORMAL Population
KERATOCONUS Population
Stiffness Parameter A1
Integrated Radius
ARTh
DA Ratio
OD
CBI
0.26
BAD D
1.35
TBI
0.41
OD 27.09.2022 at 12:05:28
Anterior axial curvature
Pachymetry
Anterior elevation (BFS)
Posterior elevation (BFS)
Millimeter
SCORE
SCORE: 0.1
Ant. Kmax: 43.95 D
Ant. Kmax - opposite K: 0.49 D
Ant. Irreg. (3mm): 0.92
Ant. inf. - sup. Kmean: 0.61 D
Post. elev. of thinnest point: 6 µm
Thinnest point: 531 µm
Averaged pachymetry towards thinnest point [µm]
Relative pachymetry towards thinnest point [%]
Pupil diameter
Pupil center

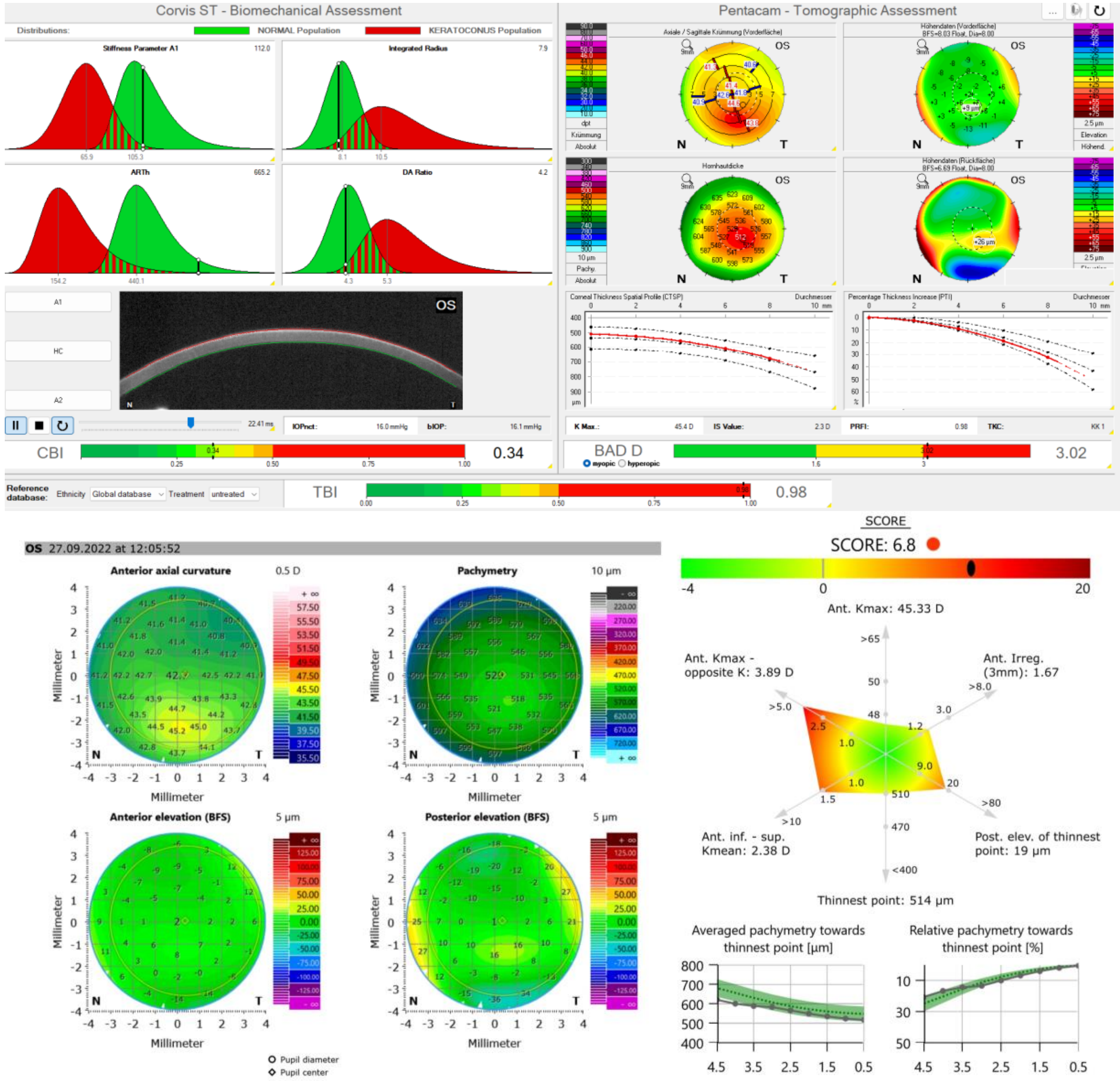
Corvis ST - Biomechanical Assessment
Pentacam - Tomographic Assessment
NORMAL Population
KERATOCONUS Population
Stiffness Parameter A1
Integrated Radius
ARTh
DA Ratio
OS
CBI
0.34
BAD D
3.02
TBI
0.98
OS 27.09.2022 at 12:05:52
Anterior axial curvature
Pachymetry
Anterior elevation (BFS)
Posterior elevation (BFS)
Millimeter
SCORE
SCORE: 6.8
Ant. Kmax: 45.33 D
Ant. Kmax - opposite K: 3.89 D
Ant. Irreg. (3mm): 1.67
Ant. inf. - sup. Kmean: 2.38 D
Post. elev. of thinnest point: 19 µm
Thinnest point: 514 µm
Averaged pachymetry towards thinnest point [µm]
Relative pachymetry towards thinnest point [%]
Pupil diameter
Pupil center

CASE #47

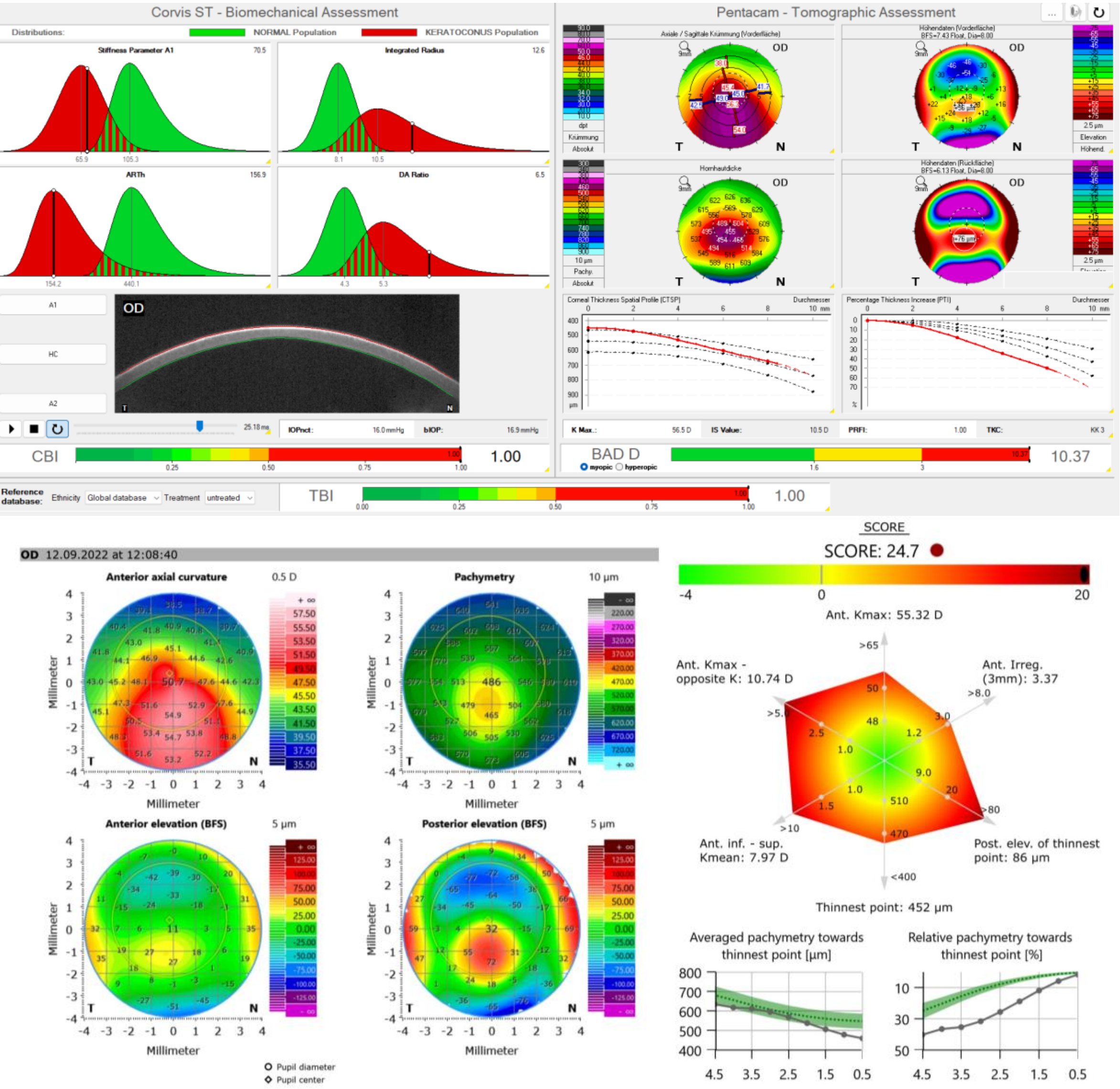
Corvis ST - Biomechanical Assessment
Pentacam - Tomographic Assessment
NORMAL Population
KERATOCONUS Population
OD
CBI
1.00
BAD D
10.37
TBI
1.00
SCORE
SCORE: 24.7
Ant. Kmax: 55.32 D
Ant. Kmax - opposite K: 10.74 D
Ant. Irreg. (3mm): 3.37
Ant. inf. - sup. Kmean: 7.97 D
Post. elev. of thinnest point: 86 µm
Thinnest point: 452 µm
OD 12.09.2022 at 12:08:40
Anterior axial curvature
Pachymetry
Anterior elevation (BFS)
Posterior elevation (BFS)
Millimeter
Averaged pachymetry towards thinnest point [µm]
Relative pachymetry towards thinnest point [%]
Pupil diameter
Pupil center

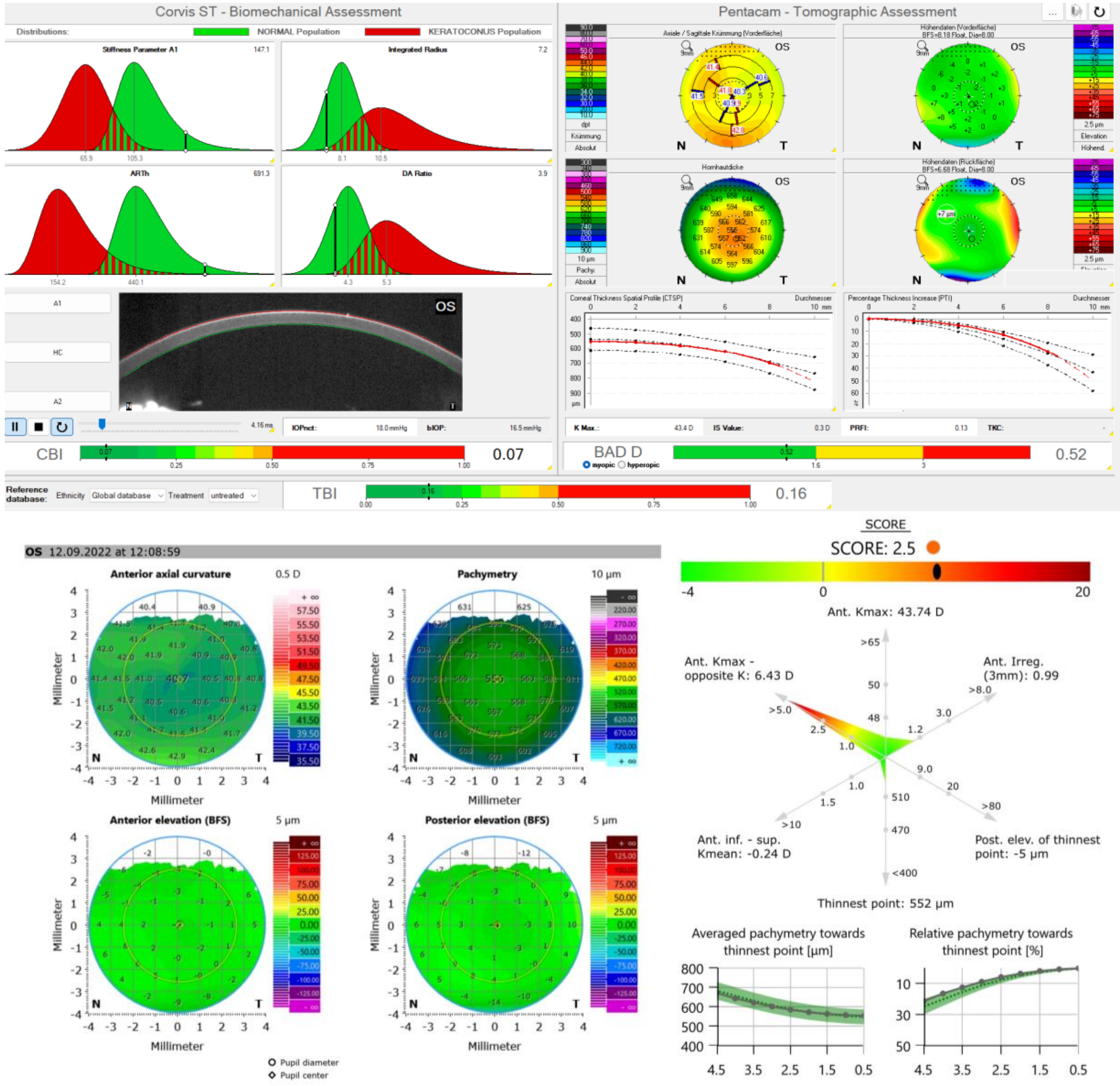
Corvis ST - Biomechanical Assessment
Pentacam - Tomographic Assessment
NORMAL Population
KERATOCONUS Population
OS
CBI
0.07
BAD D
0.52
TBI
0.16
SCORE
SCORE: 2.5
Ant. Kmax: 43.74 D
Ant. Kmax - opposite K: 6.43 D
Ant. Irreg. (3mm): 0.99
Ant. inf. - sup. Kmean: -0.24 D
Post. elev. of thinnest point: -5 µm
Thinnest point: 552 µm
OS 12.09.2022 at 12:08:59
Anterior axial curvature
Pachymetry
Anterior elevation (BFS)
Posterior elevation (BFS)
Millimeter
Averaged pachymetry towards thinnest point [µm]
Relative pachymetry towards thinnest point [%]
Pupil diameter
Pupil center

CASE #48

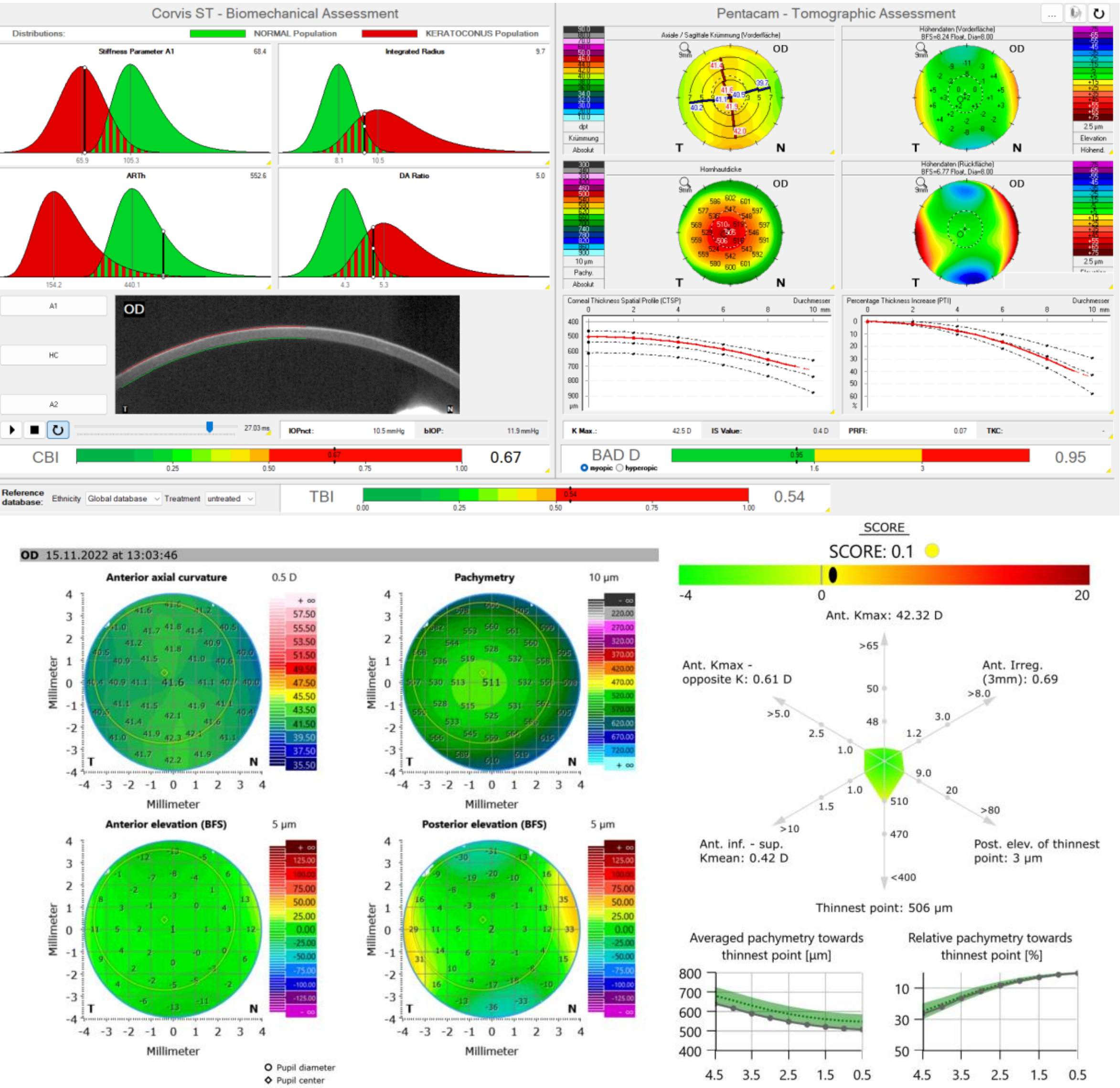
Corvis ST - Biomechanical Assessment
Pentacam - Tomographic Assessment
Distributions: NORMAL Population KERATOCONUS Population
Stiffness Parameter A1
Integrated Radius
ARTh
DA Ratio
OD
CBI 0.67
BAD D 0.95
TBI 0.54
Reference database: Ethnicity Global database Treatment untreated
OD 15.11.2022 at 13:03:46
Anterior axial curvature
Pachymetry
Anterior elevation (BFS)
Posterior elevation (BFS)
Millimeter
Pupil diameter
Pupil center
SCORE
SCORE: 0.1
Ant. Kmax: 42.32 D
Ant. Kmax - opposite K: 0.61 D
Ant. Irreg. (3mm): 0.69
Ant. inf. - sup. Kmean: 0.42 D
Post. elev. of thinnest point: 3 µm
Thinnest point: 506 µm
Averaged pachymetry towards thinnest point [µm]
Relative pachymetry towards thinnest point [%]

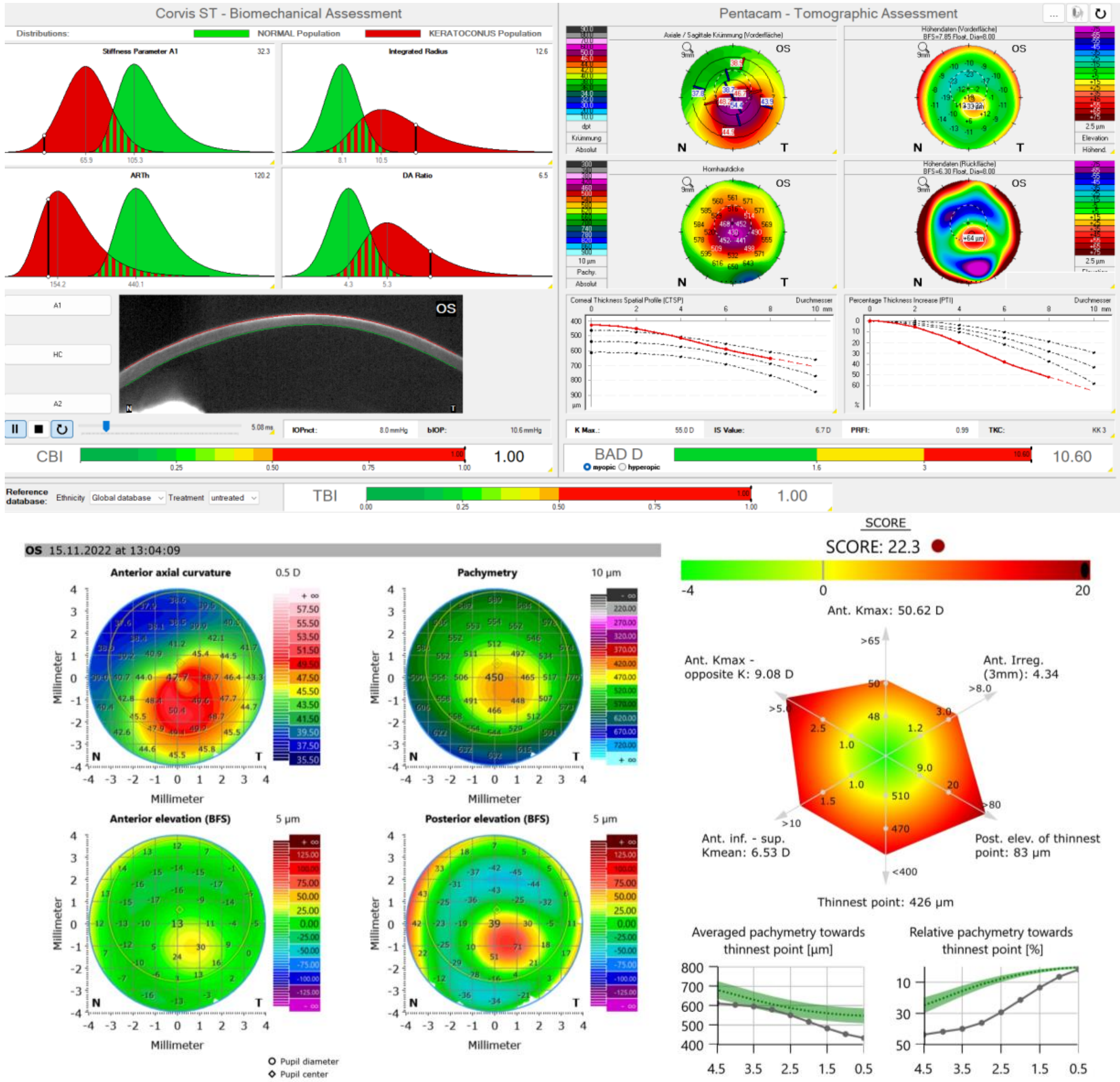
Corvis ST - Biomechanical Assessment
Pentacam - Tomographic Assessment
Distributions: NORMAL Population KERATOCONUS Population
Stiffness Parameter A1
Integrated Radius
ARTh
DA Ratio
OS
CBI 1.00
BAD D 10.60
TBI 1.00
Reference database: Ethnicity Global database Treatment untreated
OS 15.11.2022 at 13:04:09
Anterior axial curvature
Pachymetry
Anterior elevation (BFS)
Posterior elevation (BFS)
Millimeter
Pupil diameter
Pupil center
SCORE
SCORE: 22.3
Ant. Kmax: 50.62 D
Ant. Kmax - opposite K: 9.08 D
Ant. Irreg. (3mm): 4.34
Ant. inf. - sup. Kmean: 6.53 D
Post. elev. of thinnest point: 83 µm
Thinnest point: 426 µm
Averaged pachymetry towards thinnest point [µm]
Relative pachymetry towards thinnest point [%]

CASE #49

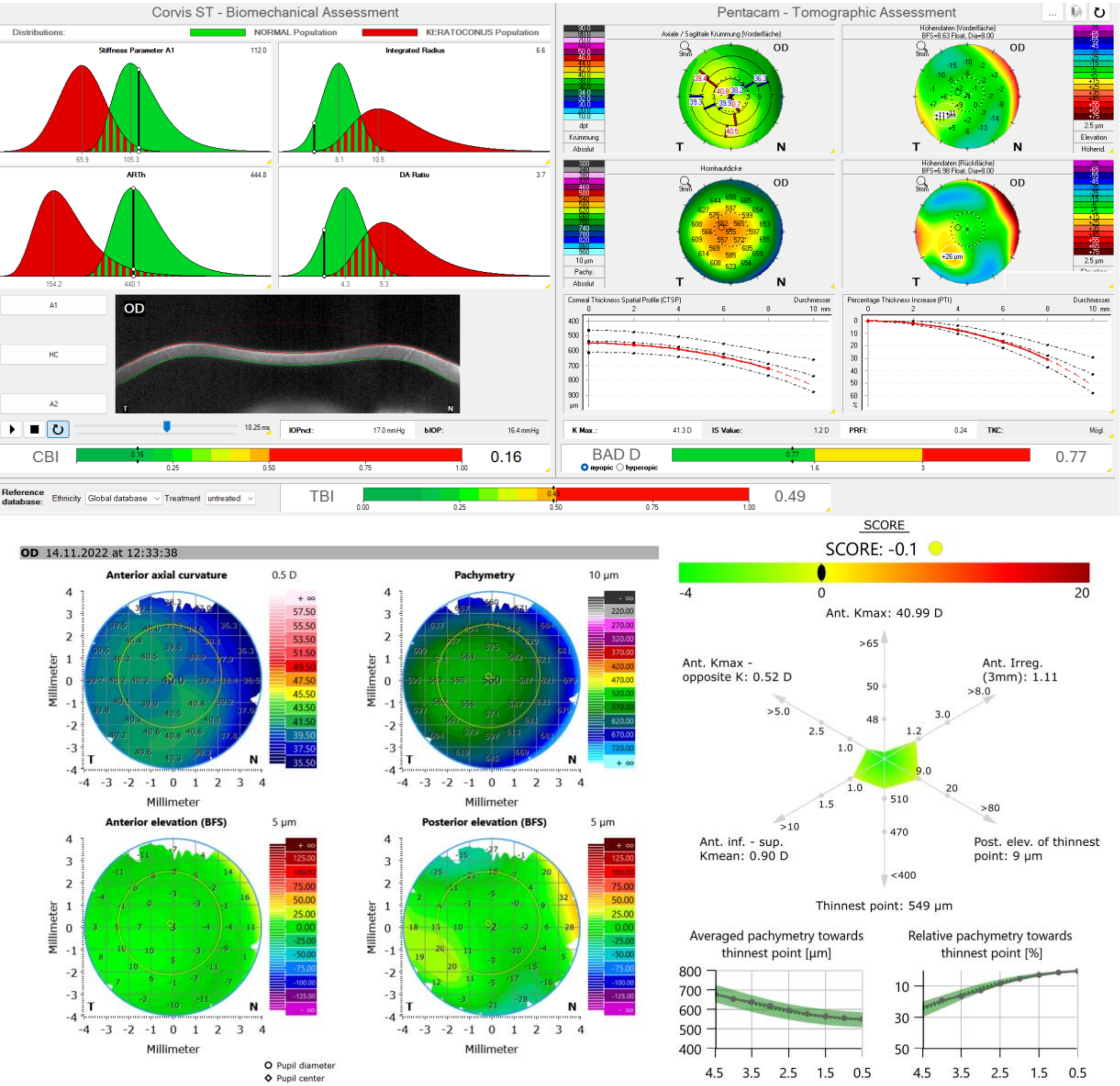

Corvis ST - Biomechanical Assessment
Pentacam - Tomographic Assessment
NORMAL Population
KERATOCONUS Population
Stiffness Parameter A1
Integrated Radius
ARTh
DA Ratio
OD
CBI
0.16
BAD D
0.77
TBI
0.49
OD 14.11.2022 at 12:33:38
Anterior axial curvature
Pachymetry
Anterior elevation (BFS)
Posterior elevation (BFS)
Millimeter
SCORE
SCORE: -0.1
Ant. Kmax: 40.99 D
Ant. Kmax - opposite K: 0.52 D
Ant. Irreg. (3mm): 1.11
Ant. inf. - sup. Kmean: 0.90 D
Post. elev. of thinnest point: 9 µm
Thinnest point: 549 µm
Averaged pachymetry towards thinnest point [µm]
Relative pachymetry towards thinnest point [%]
Pupil diameter
Pupil center

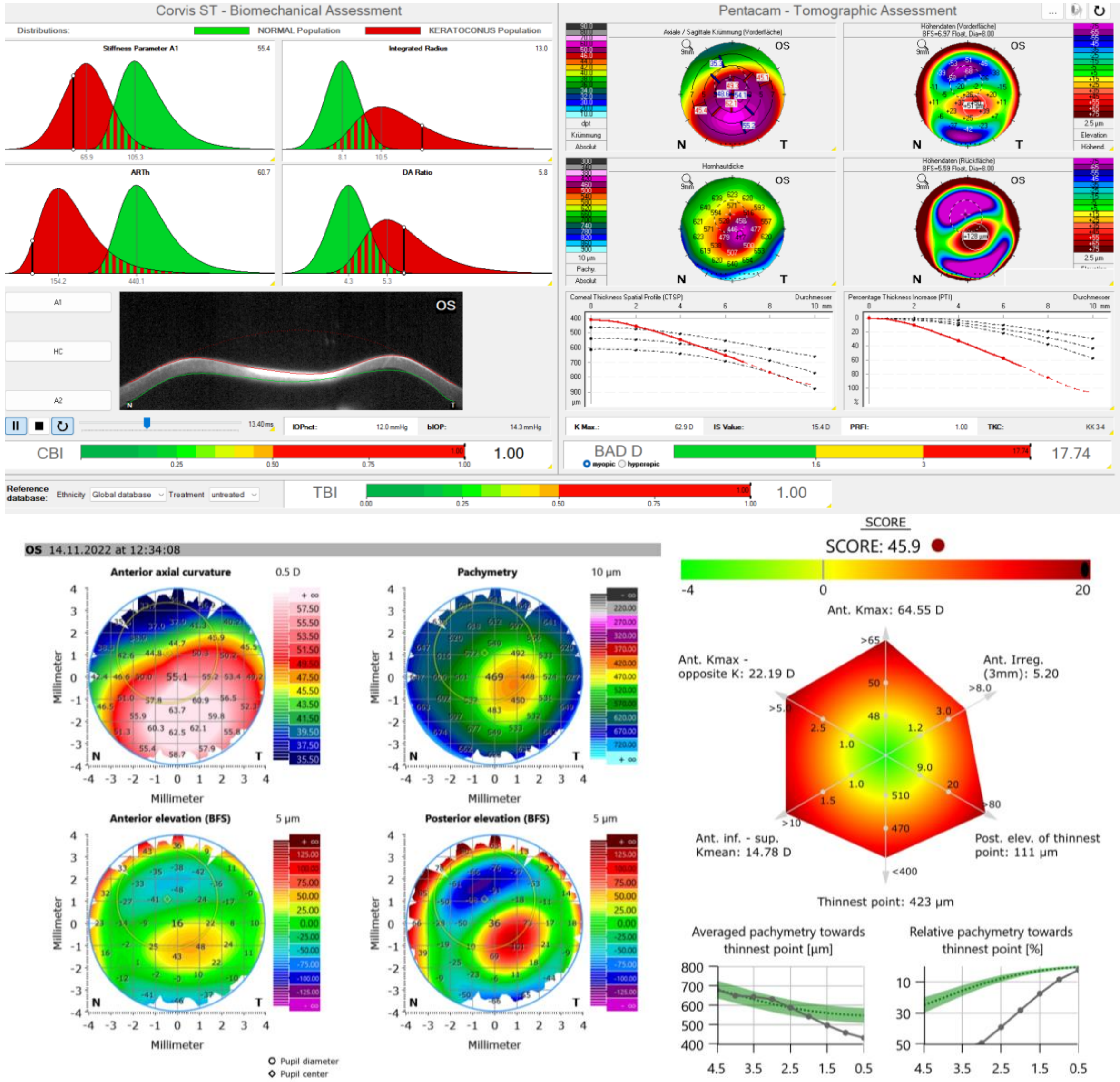

Corvis ST - Biomechanical Assessment
Pentacam - Tomographic Assessment
NORMAL Population
KERATOCONUS Population
Stiffness Parameter A1
Integrated Radius
ARTh
DA Ratio
OS
CBI
1.00
BAD D
17.74
TBI
1.00
OS 14.11.2022 at 12:34:08
Anterior axial curvature
Pachymetry
Anterior elevation (BFS)
Posterior elevation (BFS)
Millimeter
SCORE
SCORE: 45.9
Ant. Kmax: 64.55 D
Ant. Kmax - opposite K: 22.19 D
Ant. Irreg. (3mm): 5.20
Ant. inf. - sup. Kmean: 14.78 D
Post. elev. of thinnest point: 111 µm
Thinnest point: 423 µm
Averaged pachymetry towards thinnest point [µm]
Relative pachymetry towards thinnest point [%]
Pupil diameter
Pupil center

CASE #50

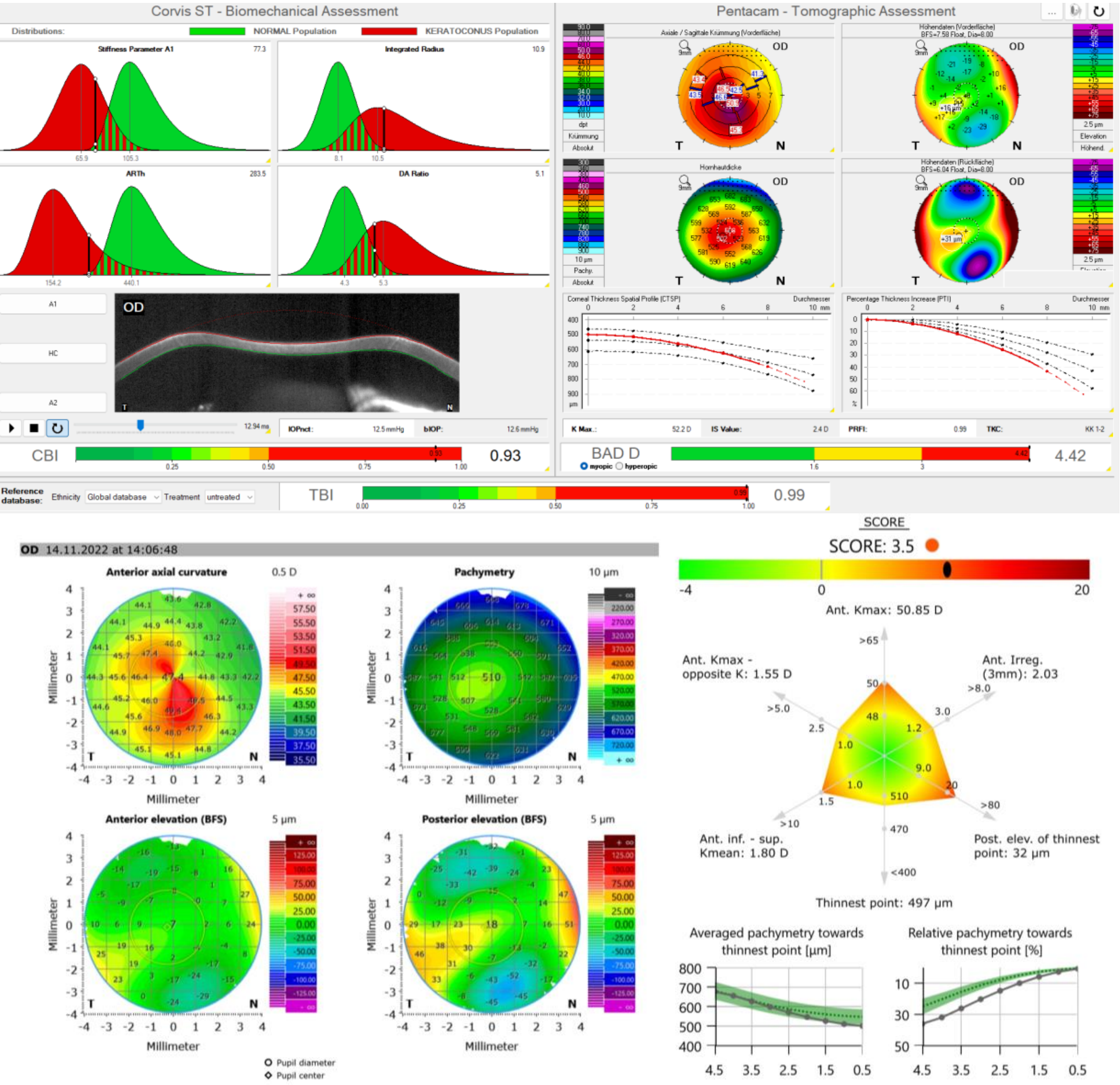
Corvis ST - Biomechanical Assessment
Pentacam - Tomographic Assessment
NORMAL Population
KERATOCONUS Population
Stiffness Parameter A1
Integrated Radius
ARTh
DA Ratio
OD
CBI
0.93
BAD D
4.42
TBI
0.99
SCORE
SCORE: 3.5
OD 14.11.2022 at 14:06:48
Anterior axial curvature
Pachymetry
Anterior elevation (BFS)
Posterior elevation (BFS)
Millimeter
Ant. Kmax: 50.85 D
Ant. Kmax - opposite K: 1.55 D
Ant. Irreg. (3mm): 2.03
Ant. inf. - sup. Kmean: 1.80 D
Post. elev. of thinnest point: 32 µm
Thinnest point: 497 µm
Averaged pachymetry towards thinnest point [µm]
Relative pachymetry towards thinnest point [%]
Pupil diameter
Pupil center

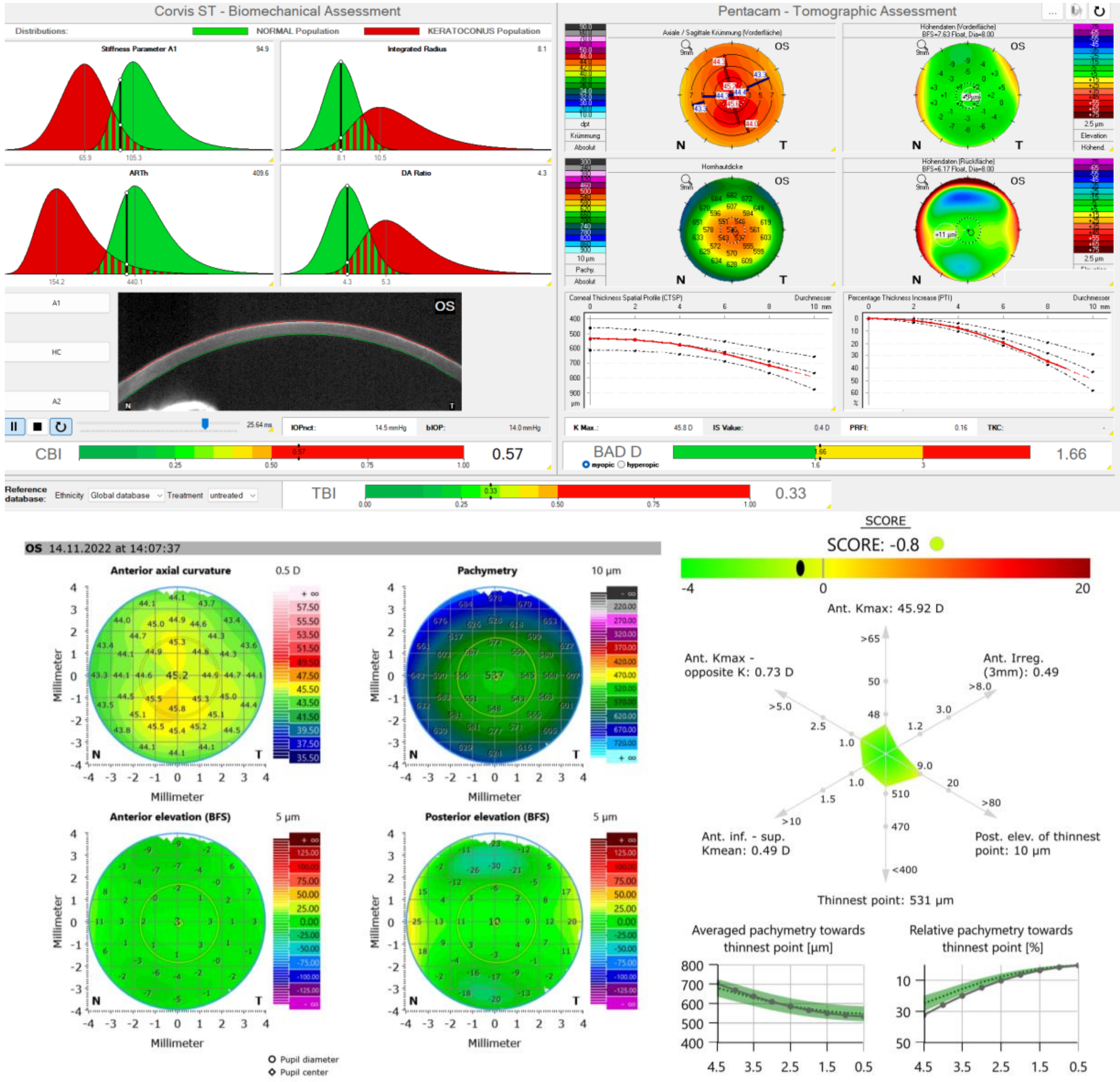
Corvis ST - Biomechanical Assessment
Pentacam - Tomographic Assessment
NORMAL Population
KERATOCONUS Population
Stiffness Parameter A1
Integrated Radius
ARTh
DA Ratio
OS
CBI
0.57
BAD D
1.66
TBI
0.33
SCORE
SCORE: -0.8
OS 14.11.2022 at 14:07:37
Anterior axial curvature
Pachymetry
Anterior elevation (BFS)
Posterior elevation (BFS)
Millimeter
Ant. Kmax: 45.92 D
Ant. Kmax - opposite K: 0.73 D
Ant. Irreg. (3mm): 0.49
Ant. inf. - sup. Kmean: 0.49 D
Post. elev. of thinnest point: 10 µm
Thinnest point: 531 µm
Averaged pachymetry towards thinnest point [µm]
Relative pachymetry towards thinnest point [%]
Pupil diameter
Pupil center

CASE #51

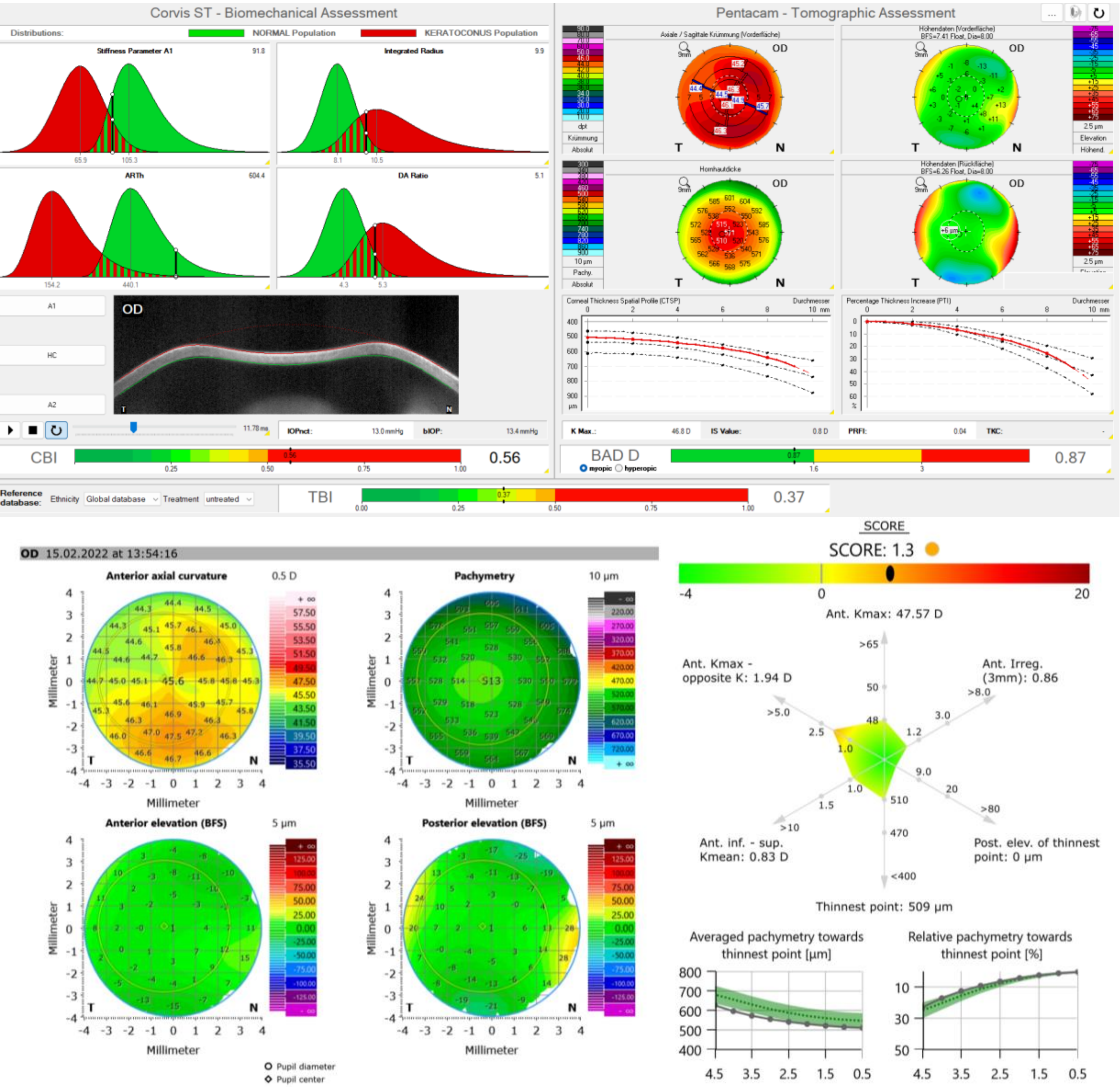

Excluded for analysis due to prior CXL

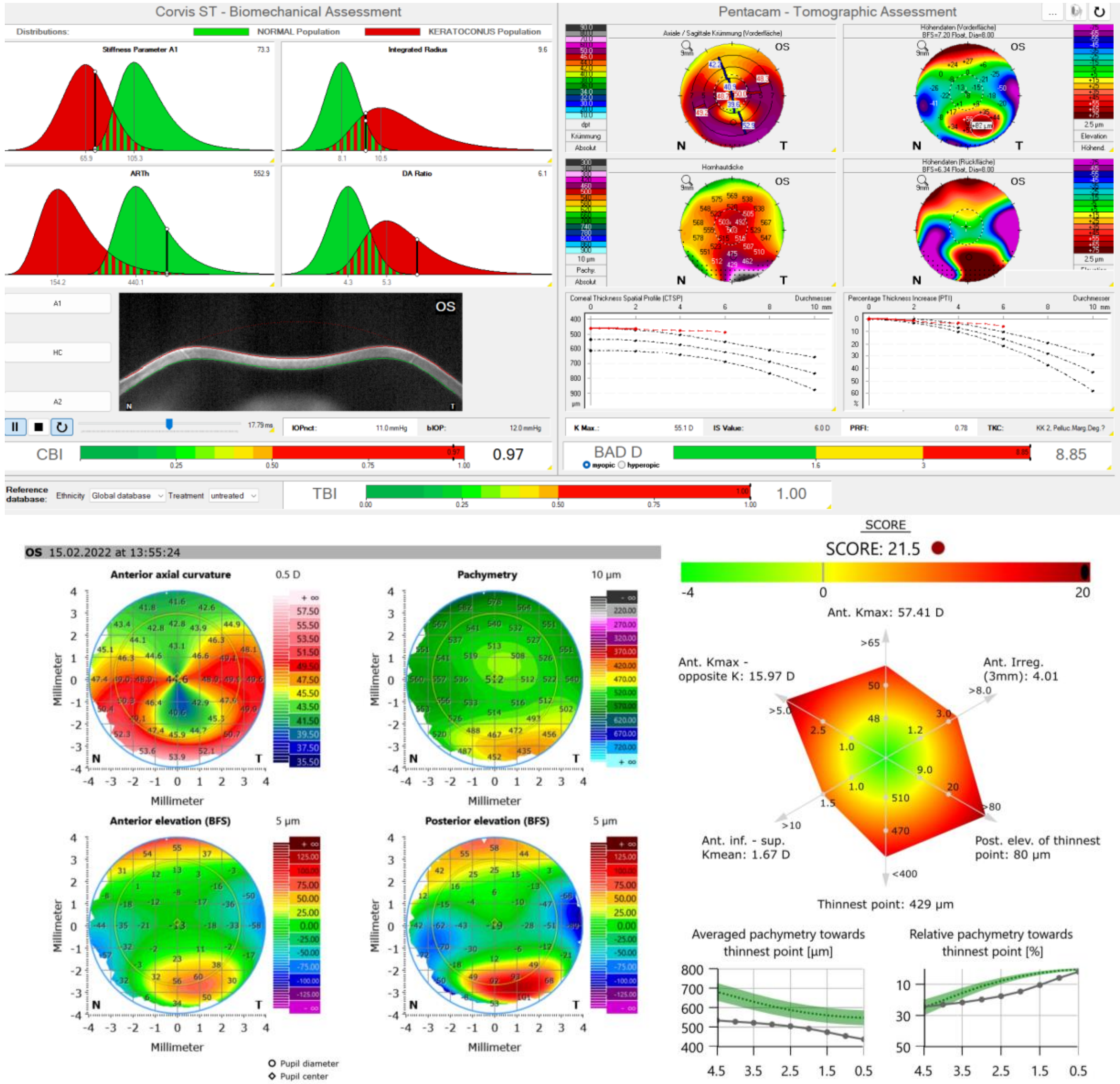

CASE #52

Excluded for analysis due to prior CXL

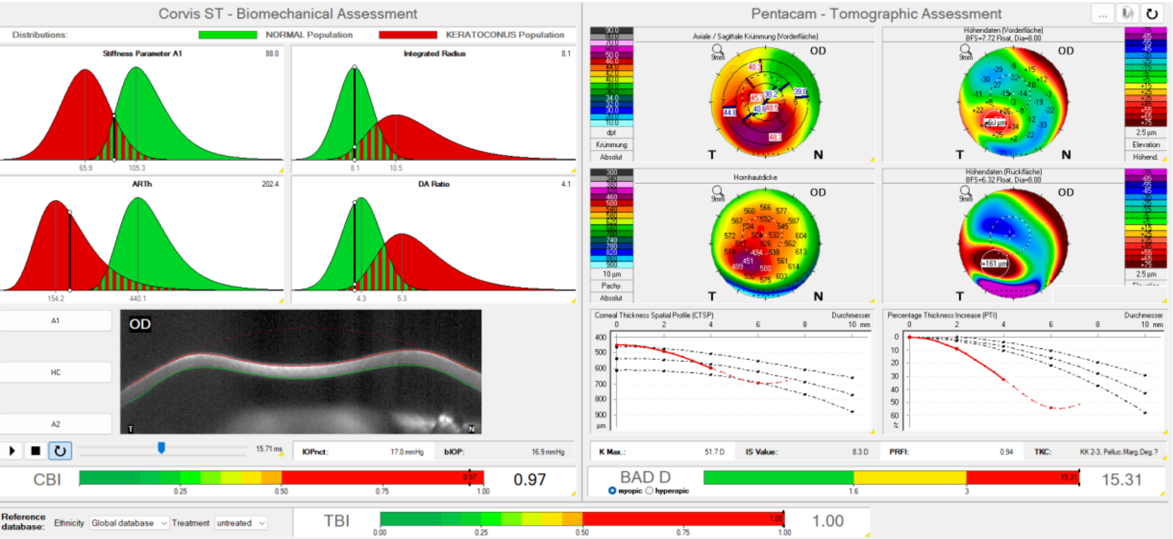

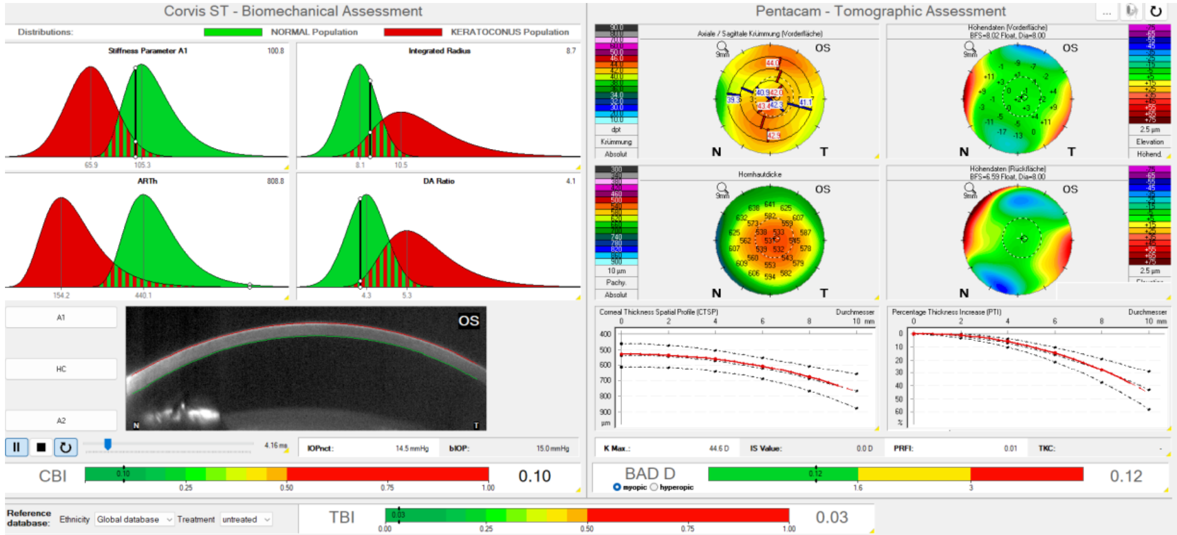

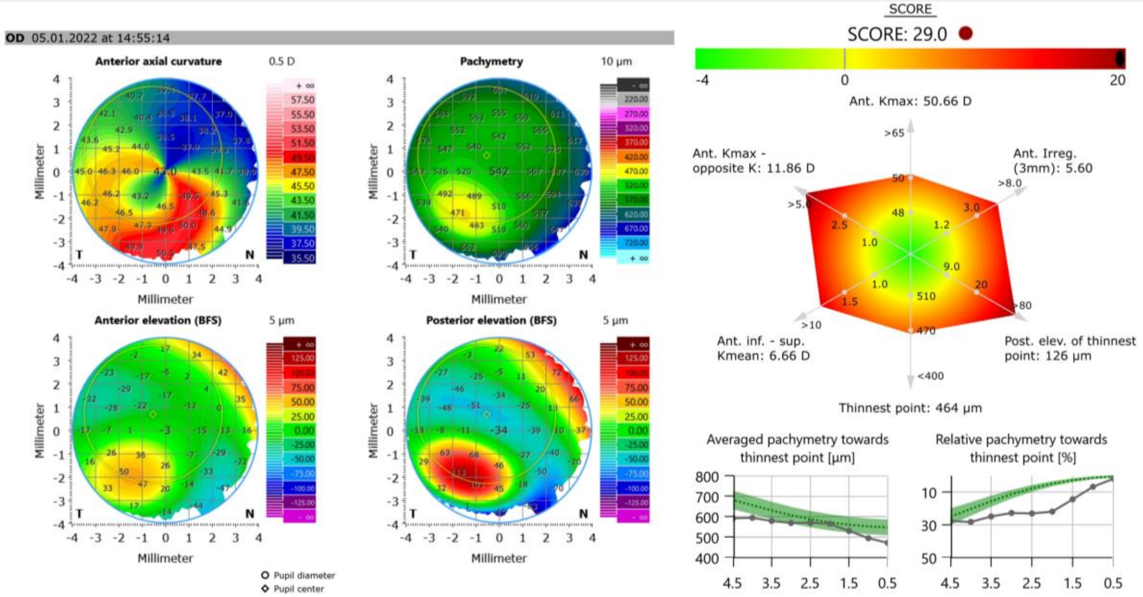

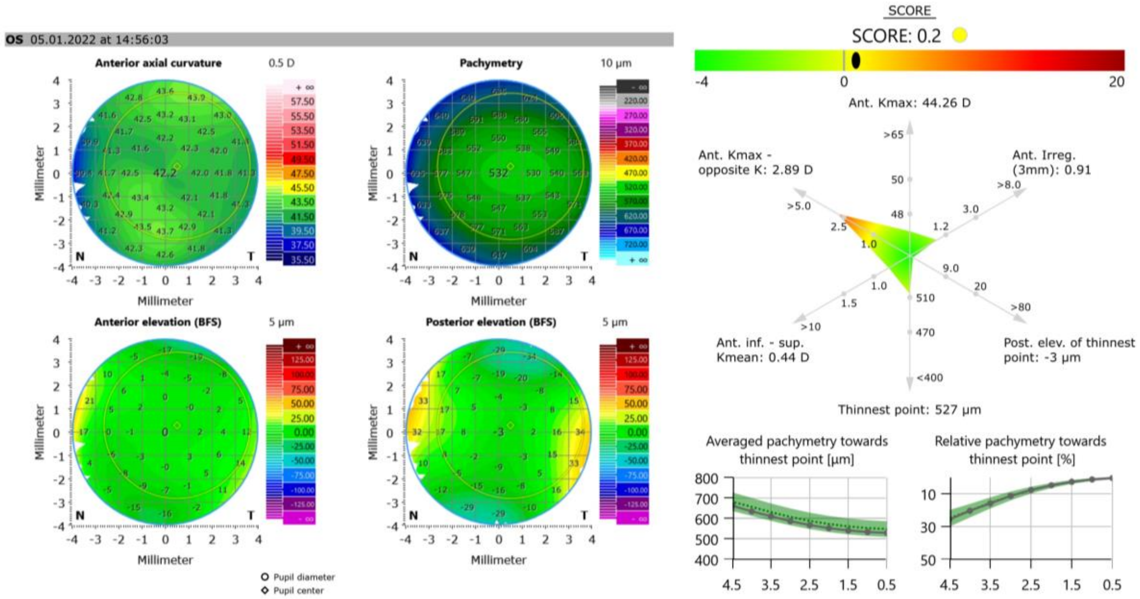

CASE #53

Excluded for analysis due to prior CXL

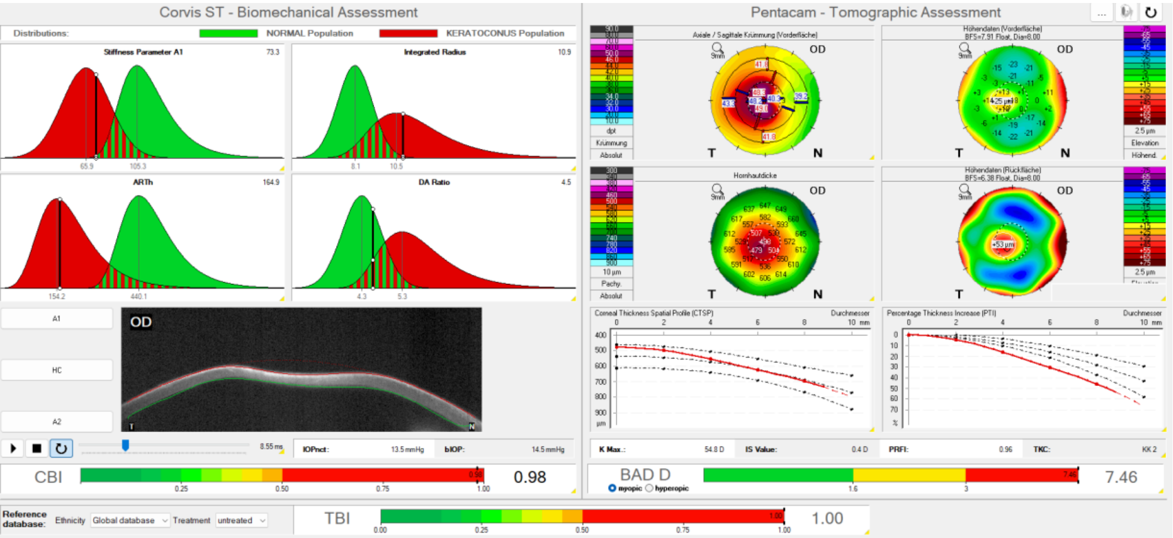

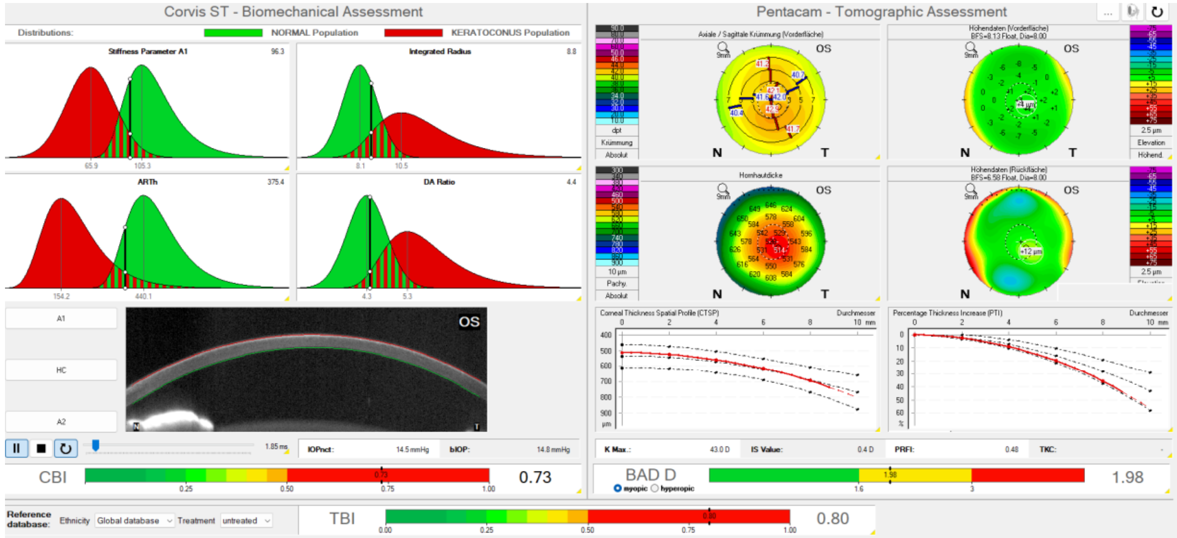

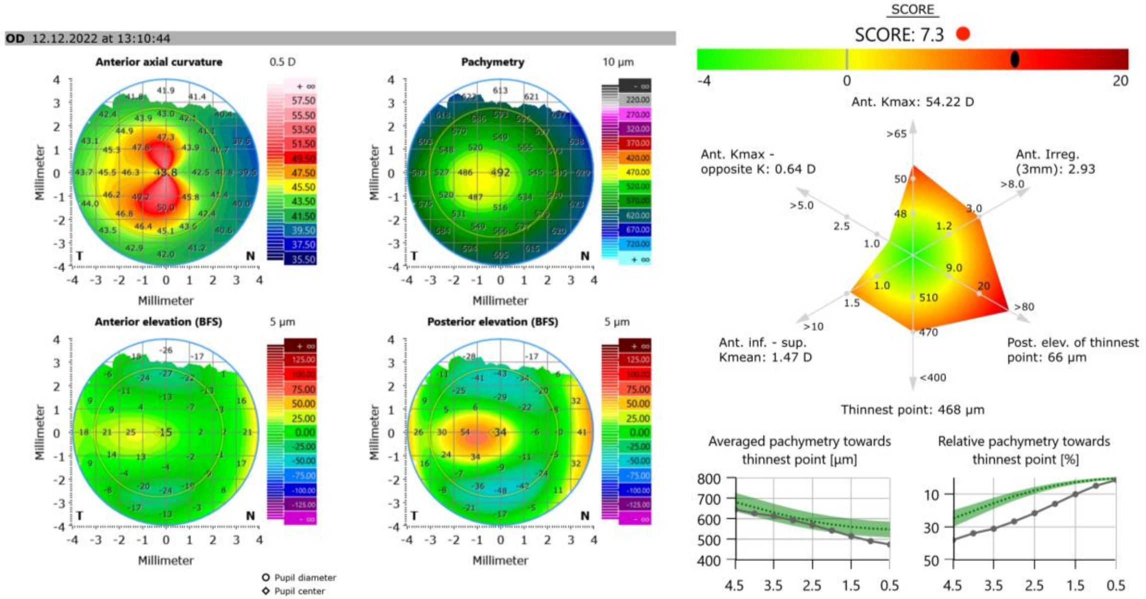

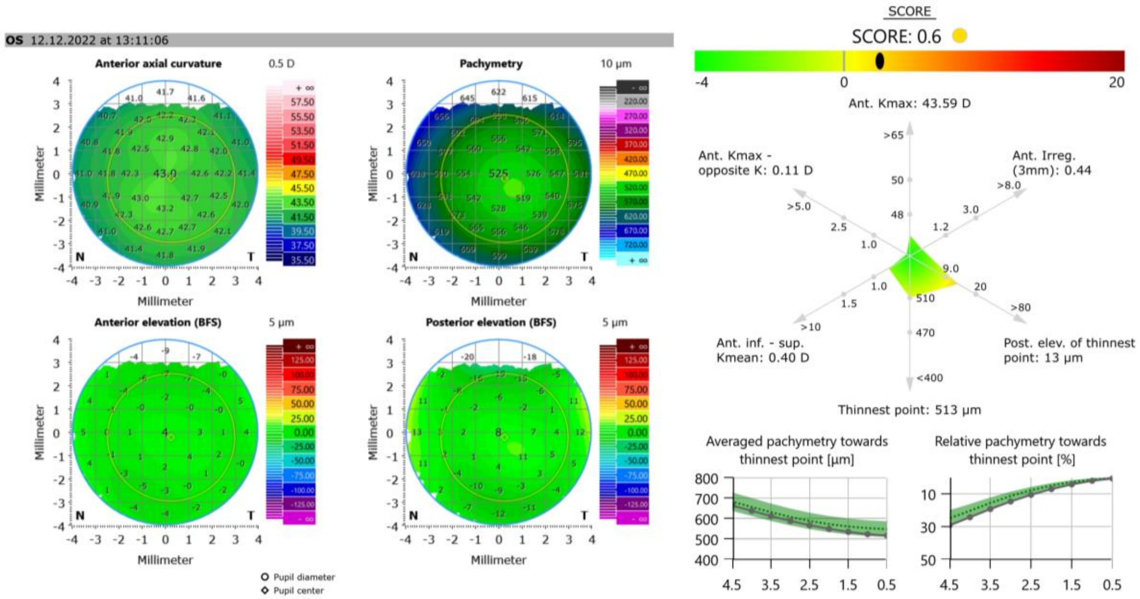

CASE #54

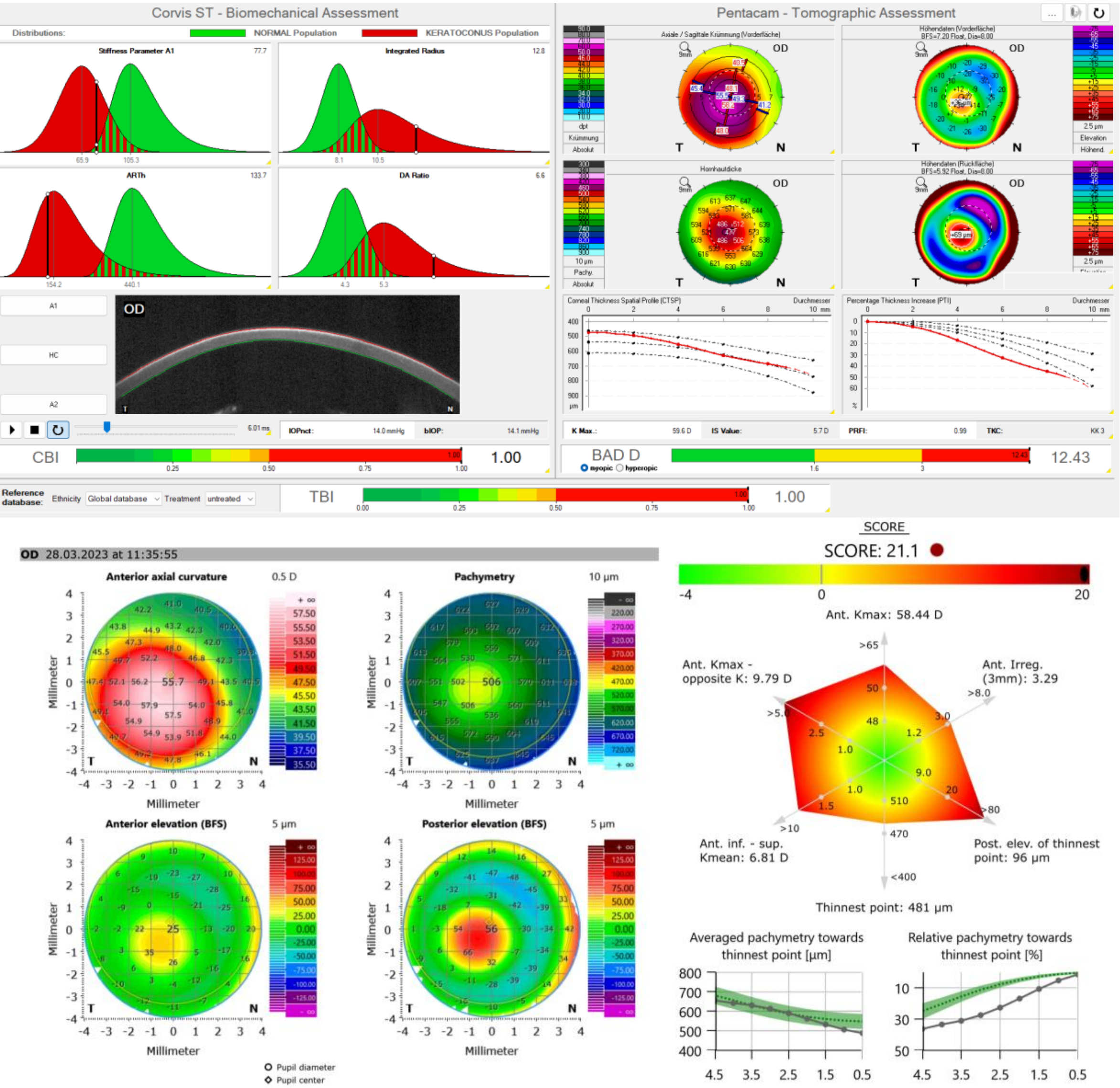
Corvis ST - Biomechanical Assessment
Pentacam - Tomographic Assessment
OD
CBI 1.00
BAD D 12.43
TBI 1.00
OD 28.03.2023 at 11:35:55
SCORE
SCORE: 21.1
Ant. Kmax: 58.44 D
Ant. Kmax - opposite K: 9.79 D
Ant. Irreg. (3mm): 3.29
Ant. inf. - sup. Kmean: 6.81 D
Post. elev. of thinnest point: 96 μm
Thinnest point: 481 μm
Averaged pachymetry towards thinnest point [μm]
Relative pachymetry towards thinnest point [%]
Anterior axial curvature
Pachymetry
Anterior elevation (BFS)
Posterior elevation (BFS)
Millimeter

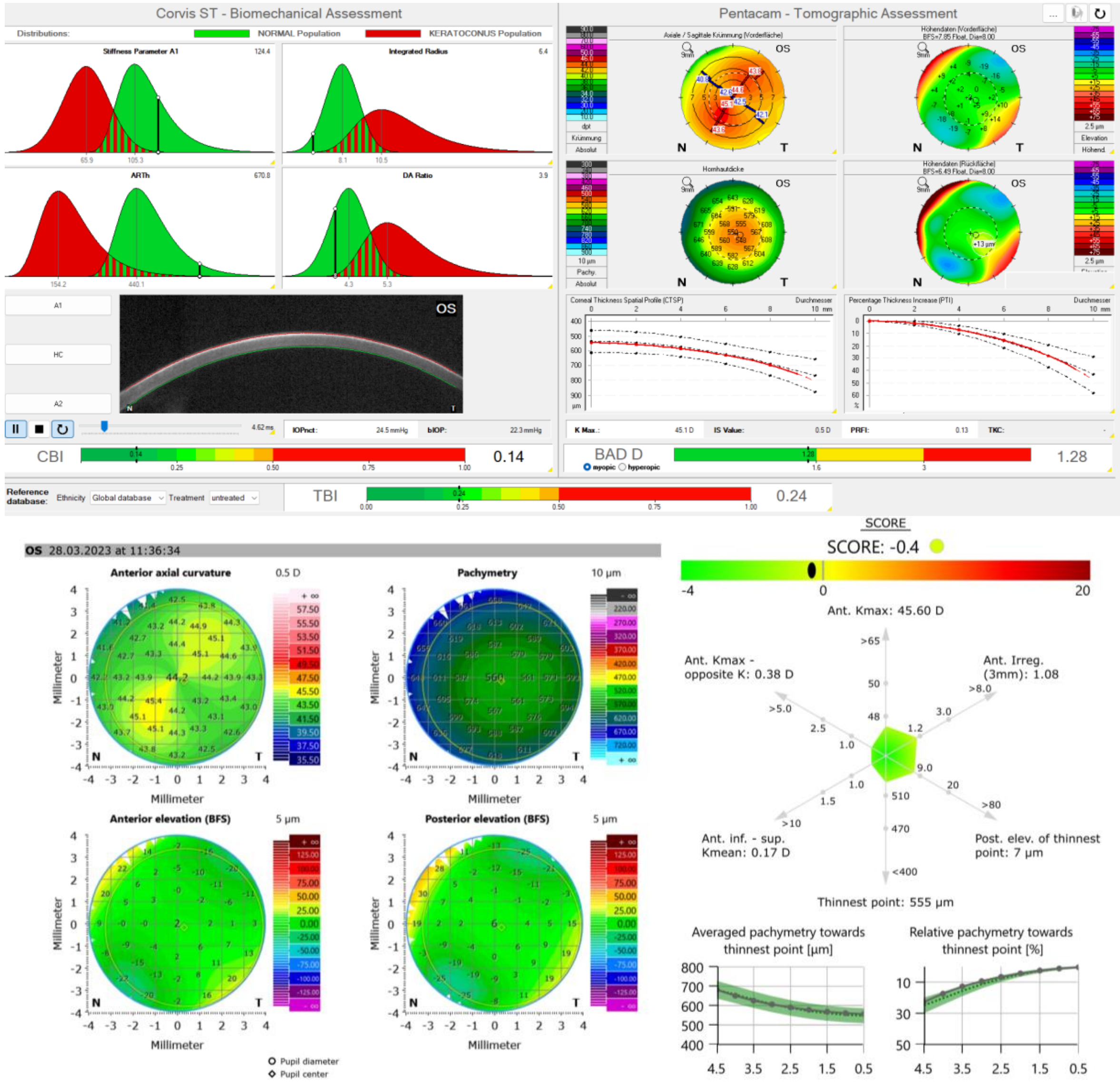
Corvis ST - Biomechanical Assessment
Pentacam - Tomographic Assessment
OS
CBI 0.14
BAD D 1.28
TBI 0.24
OS 28.03.2023 at 11:36:34
SCORE
SCORE: -0.4
Ant. Kmax: 45.60 D
Ant. Kmax - opposite K: 0.38 D
Ant. Irreg. (3mm): 1.08
Ant. inf. - sup. Kmean: 0.17 D
Post. elev. of thinnest point: 7 μm
Thinnest point: 555 μm
Averaged pachymetry towards thinnest point [μm]
Relative pachymetry towards thinnest point [%]
Anterior axial curvature
Pachymetry
Anterior elevation (BFS)
Posterior elevation (BFS)
Millimeter

CASE #55

Excluded for analysis due to prior CXL

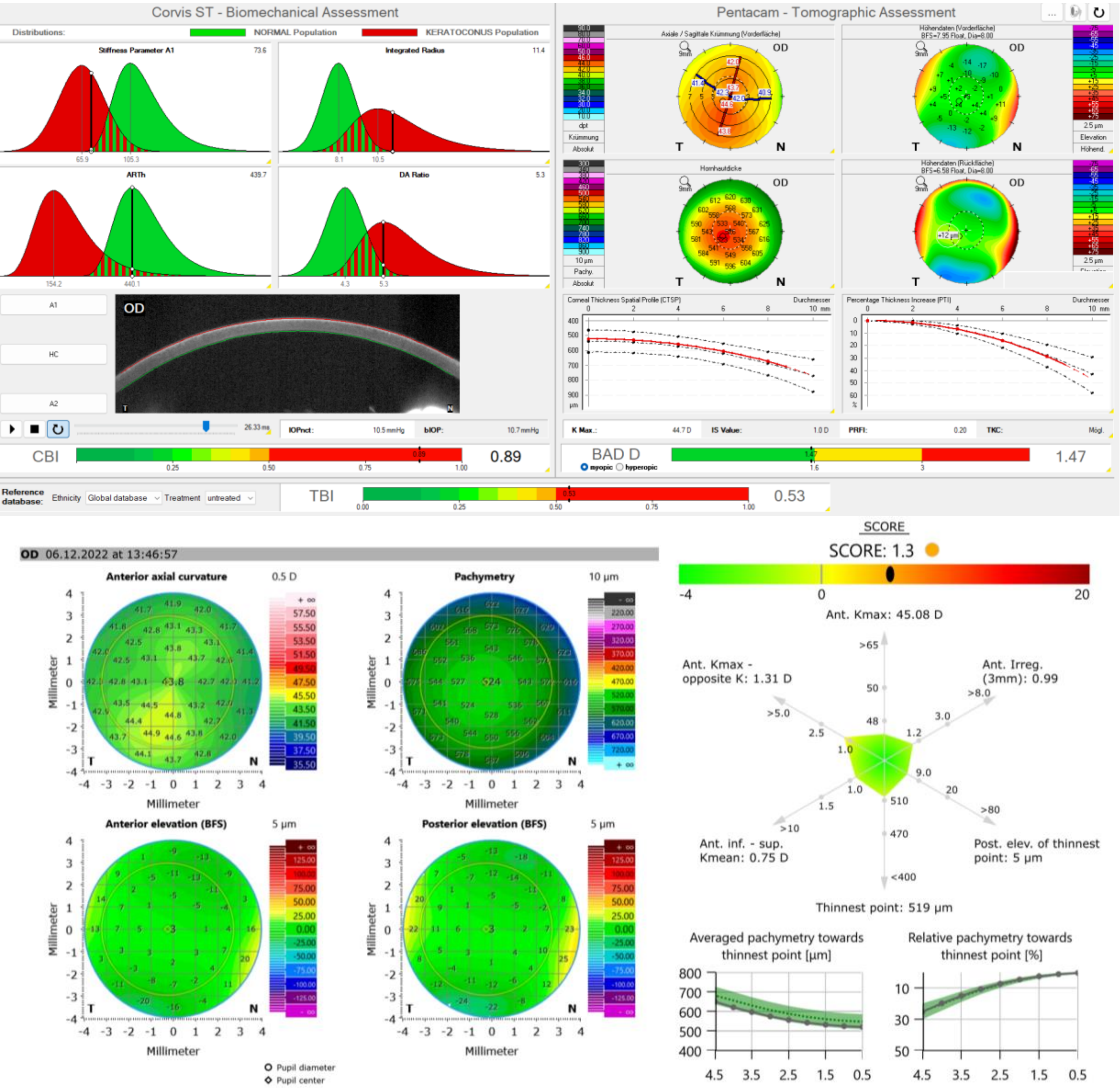

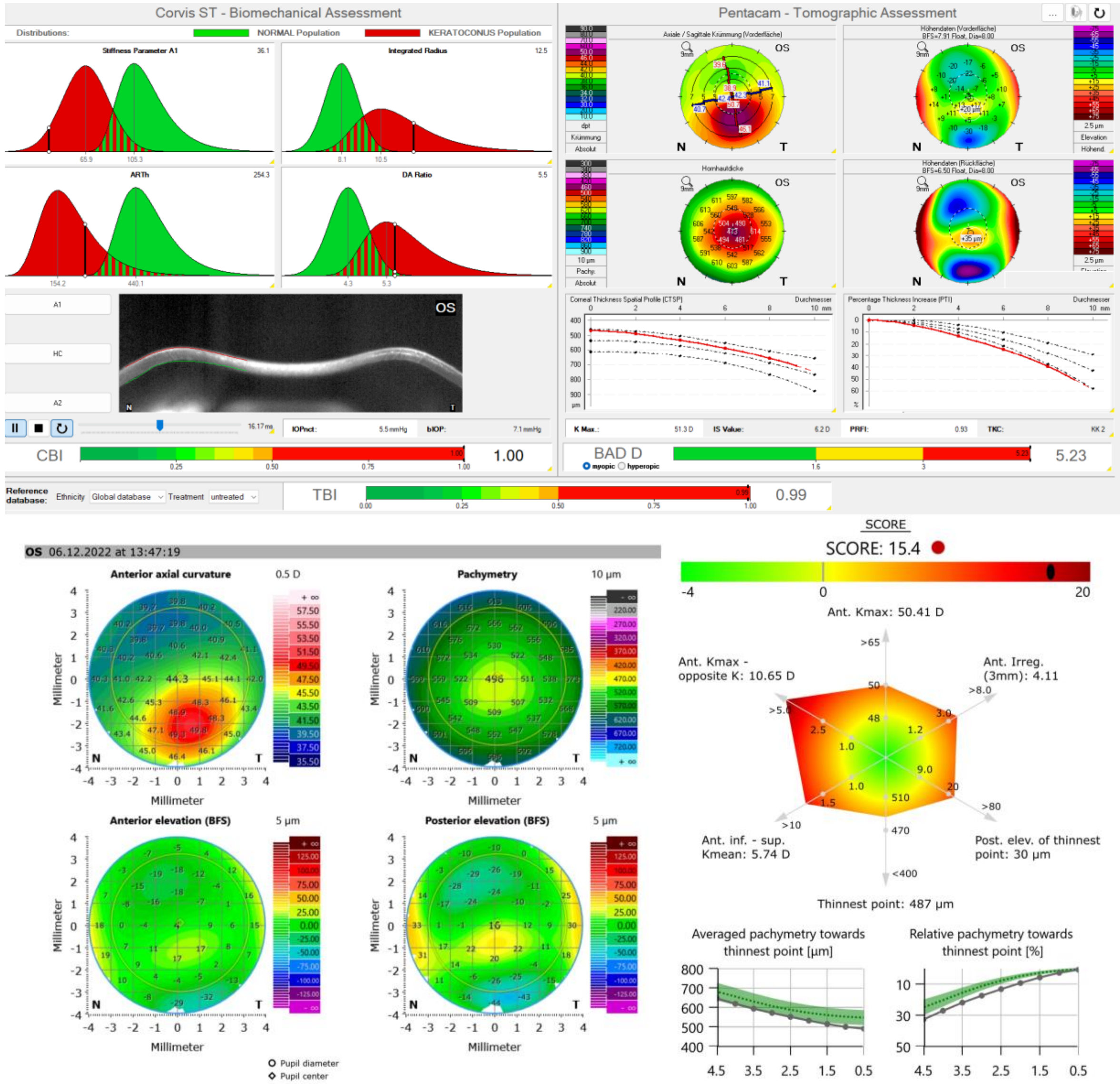

CASE #56

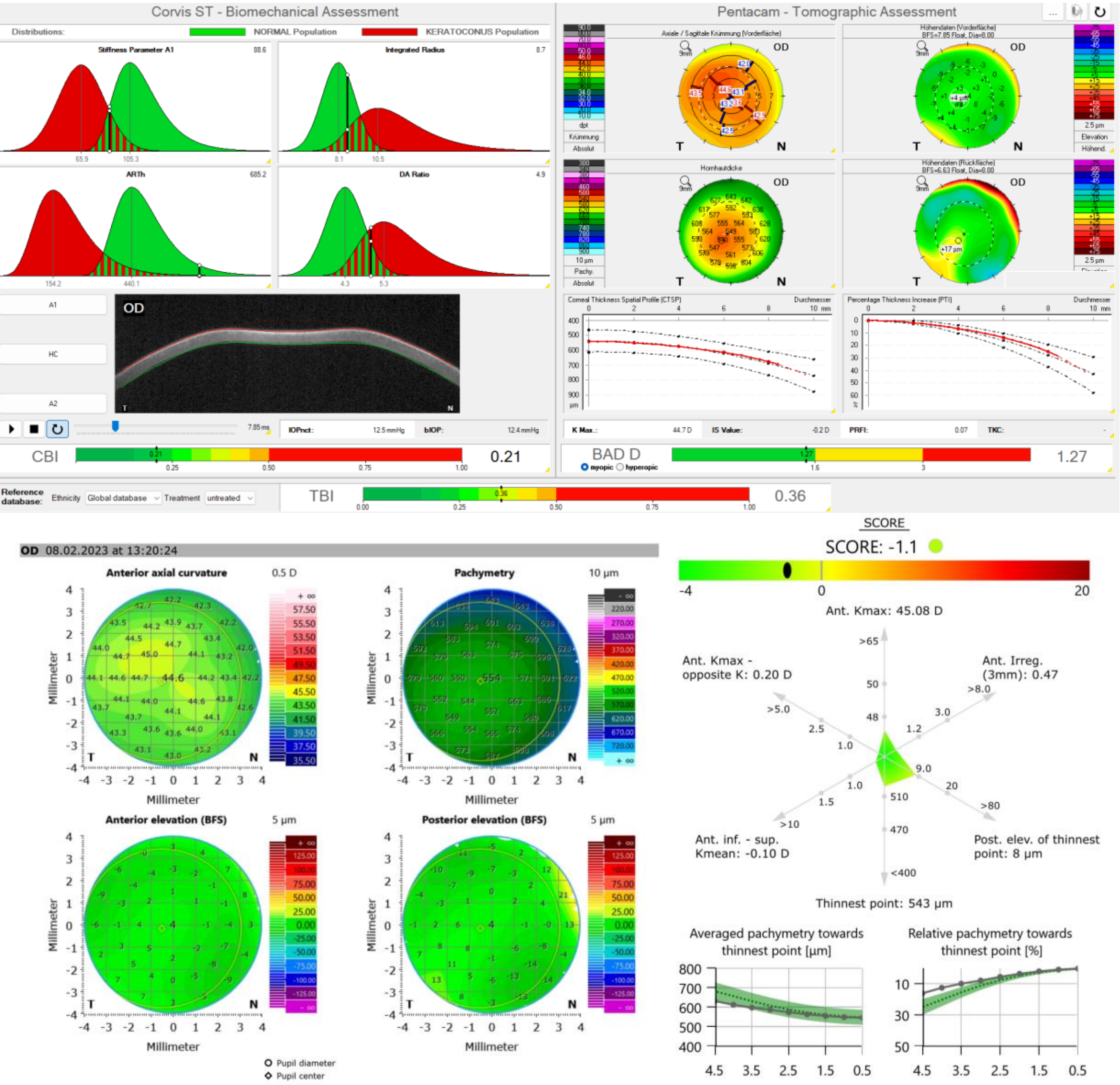
Corvis ST - Biomechanical Assessment
Pentacam - Tomographic Assessment
NORMAL Population
KERATOCONUS Population
Stiffness Parameter A1
Integrated Radius
ARTh
DA Ratio
OD
CBI
0.21
BAD D
1.27
TBI
0.36
OD 08.02.2023 at 13:20:24
Anterior axial curvature
Pachymetry
Anterior elevation (BFS)
Posterior elevation (BFS)
Millimeter
SCORE
SCORE: -1.1
Ant. Kmax: 45.08 D
Ant. Kmax - opposite K: 0.20 D
Ant. Irreg. (3mm): 0.47
Ant. inf. - sup. Kmean: -0.10 D
Post. elev. of thinnest point: 8 µm
Thinnest point: 543 µm
Averaged pachymetry towards thinnest point [µm]
Relative pachymetry towards thinnest point [%]
Pupil diameter
Pupil center

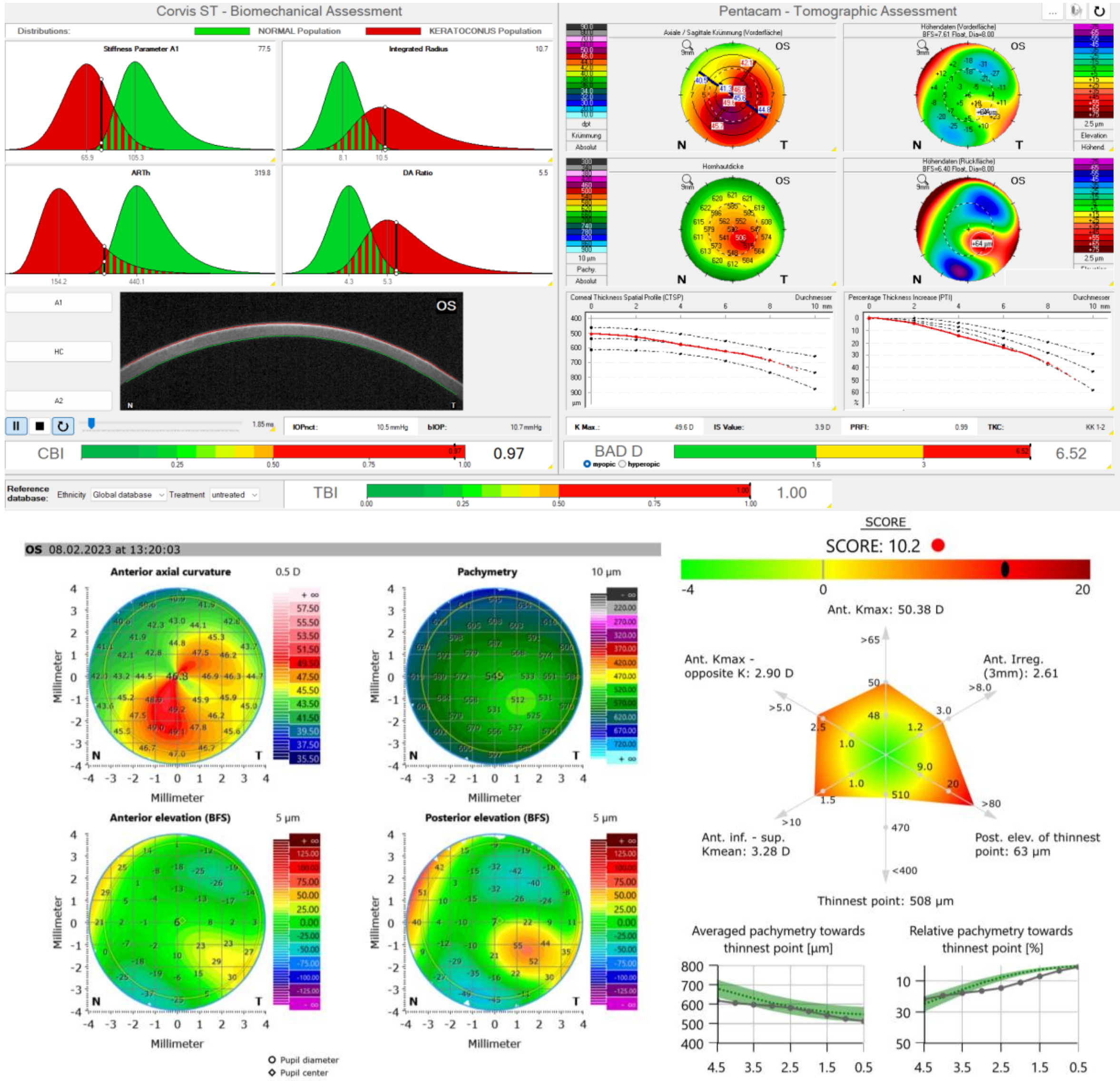
Corvis ST - Biomechanical Assessment
Pentacam - Tomographic Assessment
NORMAL Population
KERATOCONUS Population
Stiffness Parameter A1
Integrated Radius
ARTh
DA Ratio
OS
CBI
0.97
BAD D
6.52
TBI
1.00
OS 08.02.2023 at 13:20:03
Anterior axial curvature
Pachymetry
Anterior elevation (BFS)
Posterior elevation (BFS)
Millimeter
SCORE
SCORE: 10.2
Ant. Kmax: 50.38 D
Ant. Kmax - opposite K: 2.90 D
Ant. Irreg. (3mm): 2.61
Ant. inf. - sup. Kmean: 3.28 D
Post. elev. of thinnest point: 63 µm
Thinnest point: 508 µm
Averaged pachymetry towards thinnest point [µm]
Relative pachymetry towards thinnest point [%]
Pupil diameter
Pupil center

CASE #57

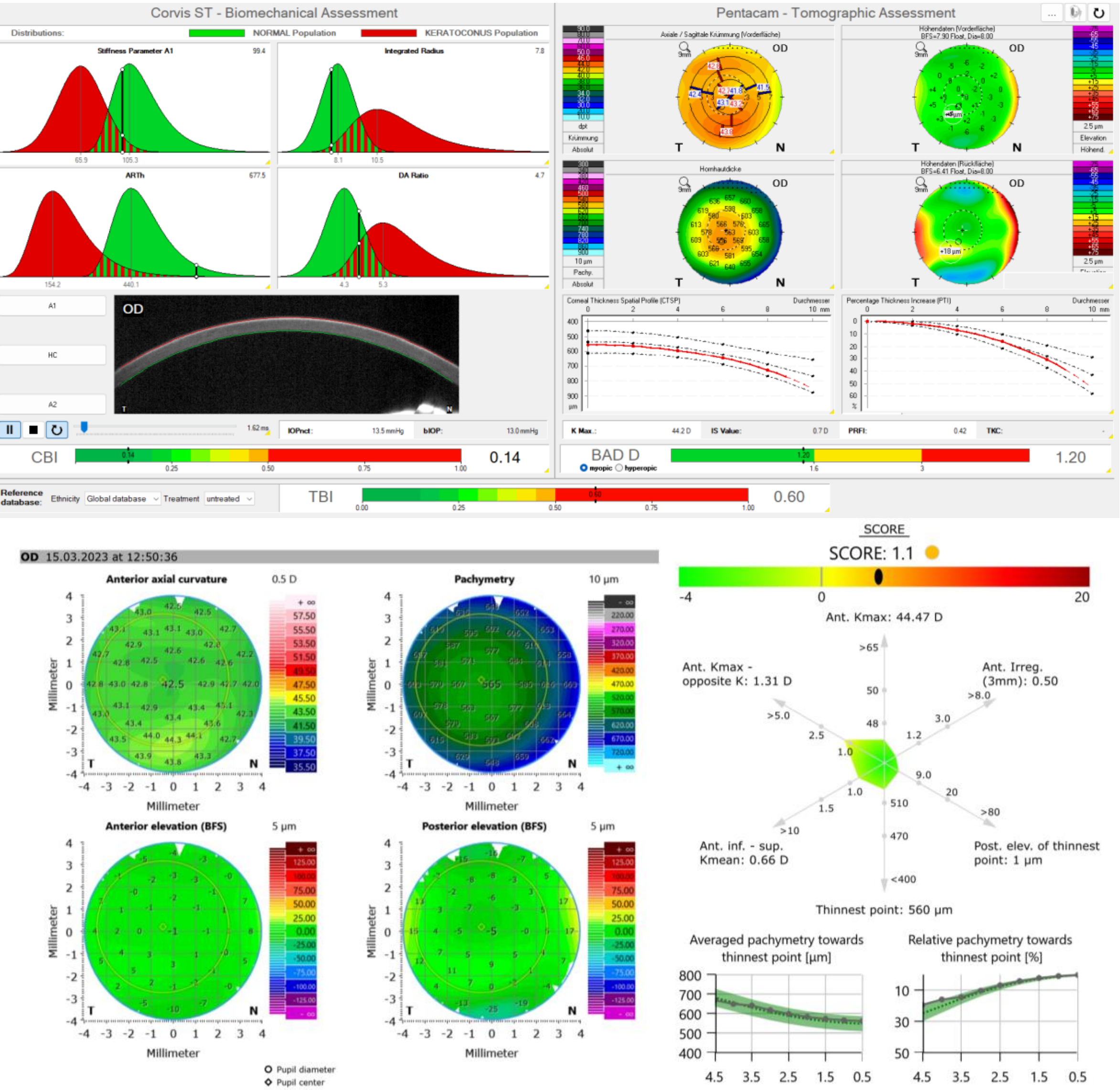

Corvis ST - Biomechanical Assessment
Pentacam - Tomographic Assessment
NORMAL Population
KERATOCONUS Population
Stiffness Parameter A1
Integrated Radius
ARTh
DA Ratio
OD
CBI 0.14
BAD D 1.20
TBI 0.60
OD 15.03.2023 at 12:50:36
Anterior axial curvature
Pachymetry
Anterior elevation (BFS)
Posterior elevation (BFS)
Millimeter
SCORE
SCORE: 1.1
Ant. Kmax: 44.47 D
Ant. Kmax - opposite K: 1.31 D
Ant. Irreg. (3mm): 0.50
Ant. inf. - sup. Kmean: 0.66 D
Post. elev. of thinnest point: 1 µm
Thinnest point: 560 µm
Averaged pachymetry towards thinnest point [µm]
Relative pachymetry towards thinnest point [%]
Pupil diameter
Pupil center

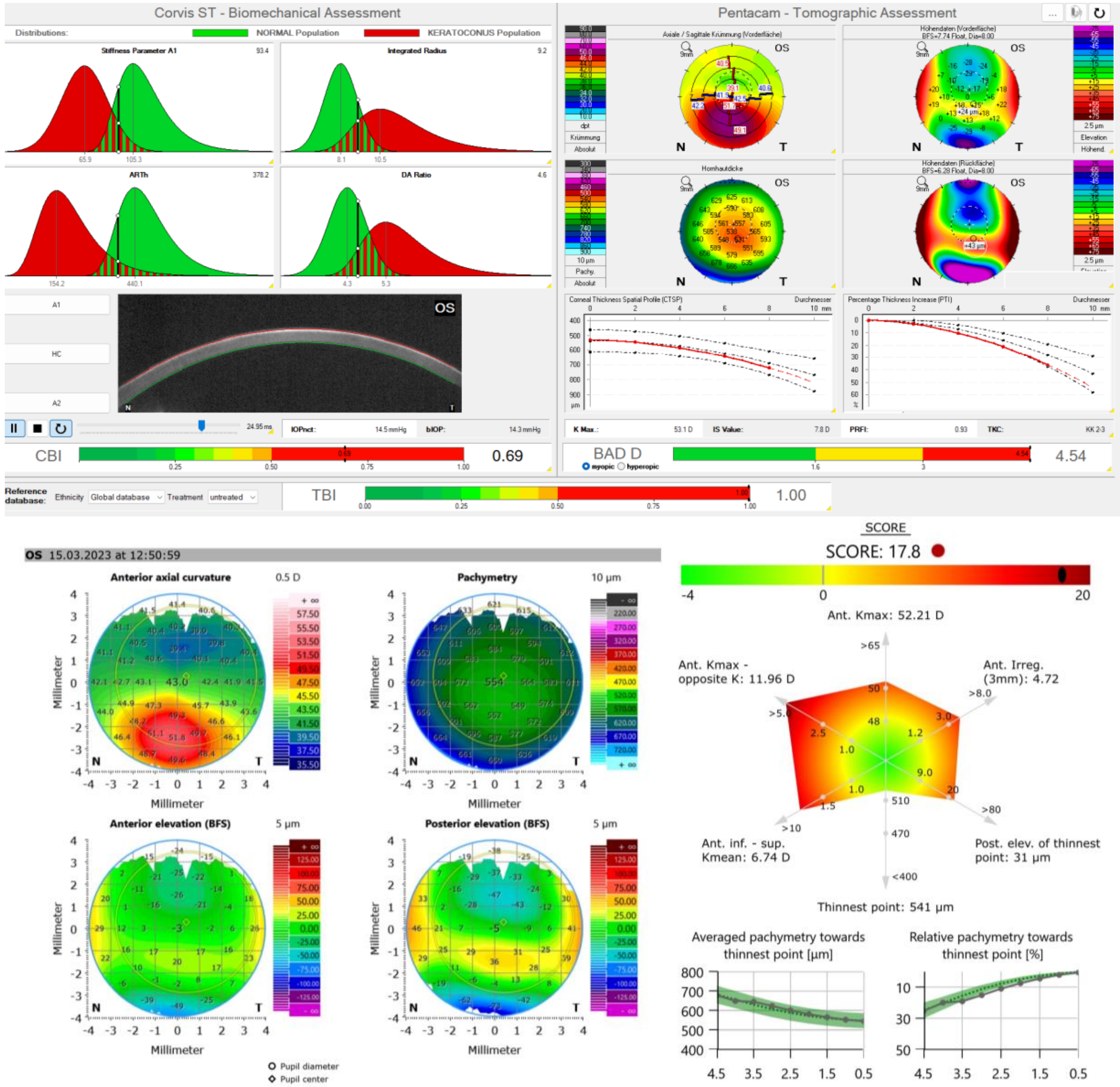

Corvis ST - Biomechanical Assessment
Pentacam - Tomographic Assessment
NORMAL Population
KERATOCONUS Population
Stiffness Parameter A1
Integrated Radius
ARTh
DA Ratio
OS
CBI 0.69
BAD D 4.54
TBI 1.00
OS 15.03.2023 at 12:50:59
Anterior axial curvature
Pachymetry
Anterior elevation (BFS)
Posterior elevation (BFS)
Millimeter
SCORE
SCORE: 17.8
Ant. Kmax: 52.21 D
Ant. Kmax - opposite K: 11.96 D
Ant. Irreg. (3mm): 4.72
Ant. inf. - sup. Kmean: 6.74 D
Post. elev. of thinnest point: 31 µm
Thinnest point: 541 µm
Averaged pachymetry towards thinnest point [µm]
Relative pachymetry towards thinnest point [%]
Pupil diameter
Pupil center

CASE #58

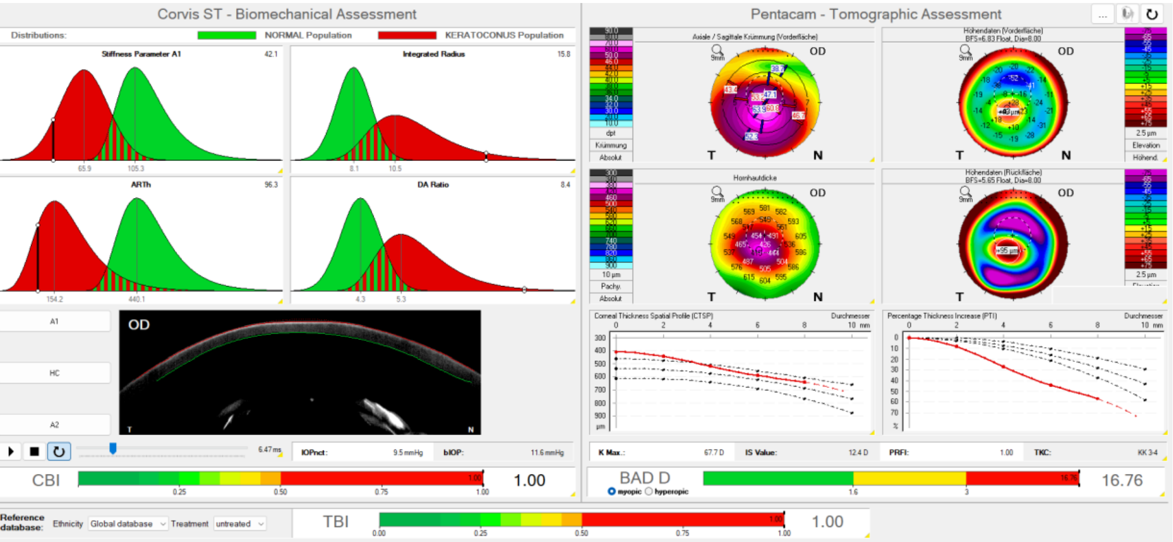
Corvis ST - Biomechanical Assessment
Pentacam - Tomographic Assessment
NORMAL Population
KERATOCONUS Population
Stiffness Parameter A1
Integrated Radius
ARTh
DA Ratio
OD
CBI
1.00
BAD D
16.76
TBI
1.00

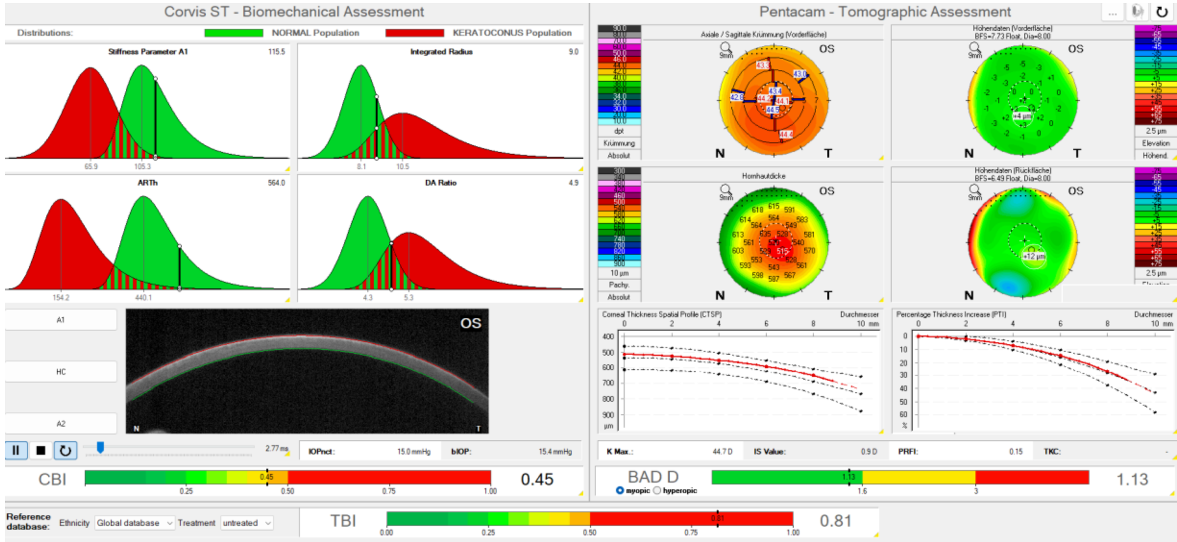
Corvis ST - Biomechanical Assessment
Pentacam - Tomographic Assessment
NORMAL Population
KERATOCONUS Population
Stiffness Parameter A1
Integrated Radius
ARTh
DA Ratio
OS
CBI
0.45
BAD D
1.13
TBI
0.81

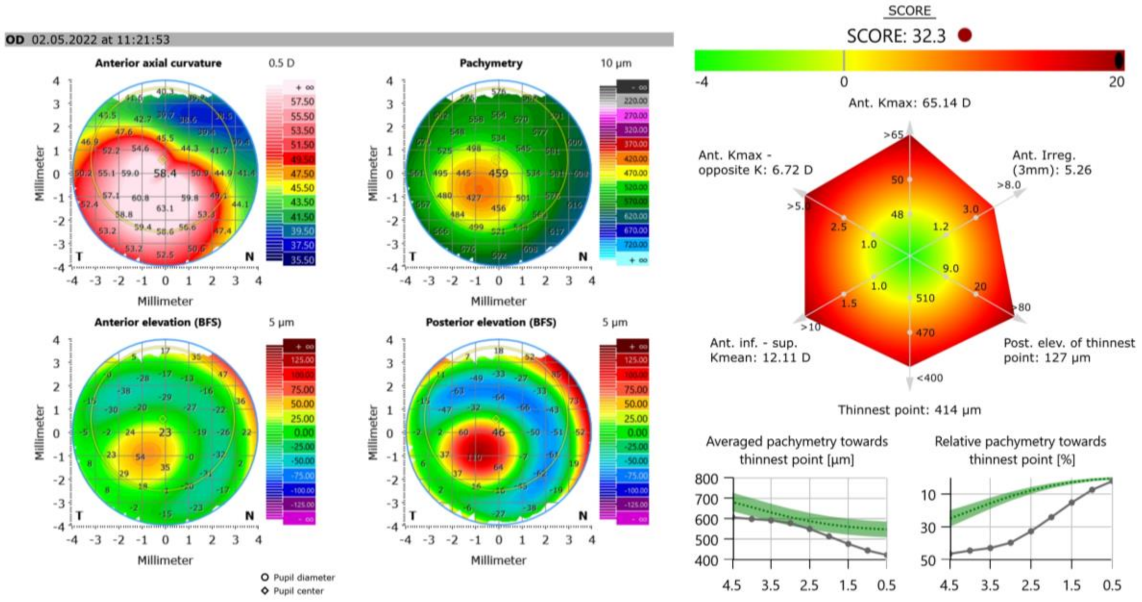
OD 02.05.2022 at 11:21:53
Anterior axial curvature
Pachymetry
Anterior elevation (BFS)
Posterior elevation (BFS)
Millimeter
SCORE
SCORE: 32.3
Ant. Kmax: 65.14 D
Ant. Kmax - opposite K: 6.72 D
Ant. Irreg. (3mm): 5.26
Ant. inf. - sup. Kmean: 12.11 D
Post. elev. of thinnest point: 127 µm
Thinnest point: 414 µm
Averaged pachymetry towards thinnest point [µm]
Relative pachymetry towards thinnest point [%]
Pupil diameter
Pupil center

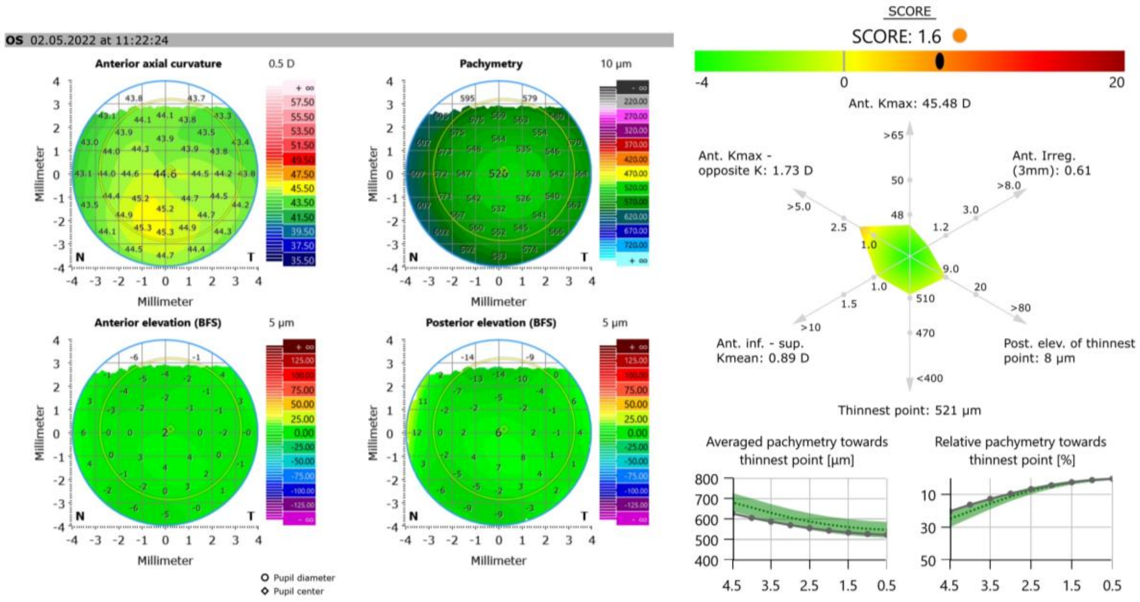
OS 02.05.2022 at 11:22:24
Anterior axial curvature
Pachymetry
Anterior elevation (BFS)
Posterior elevation (BFS)
Millimeter
SCORE
SCORE: 1.6
Ant. Kmax: 45.48 D
Ant. Kmax - opposite K: 1.73 D
Ant. Irreg. (3mm): 0.61
Ant. inf. - sup. Kmean: 0.89 D
Post. elev. of thinnest point: 8 µm
Thinnest point: 521 µm
Averaged pachymetry towards thinnest point [µm]
Relative pachymetry towards thinnest point [%]
Pupil diameter
Pupil center

CASE #59

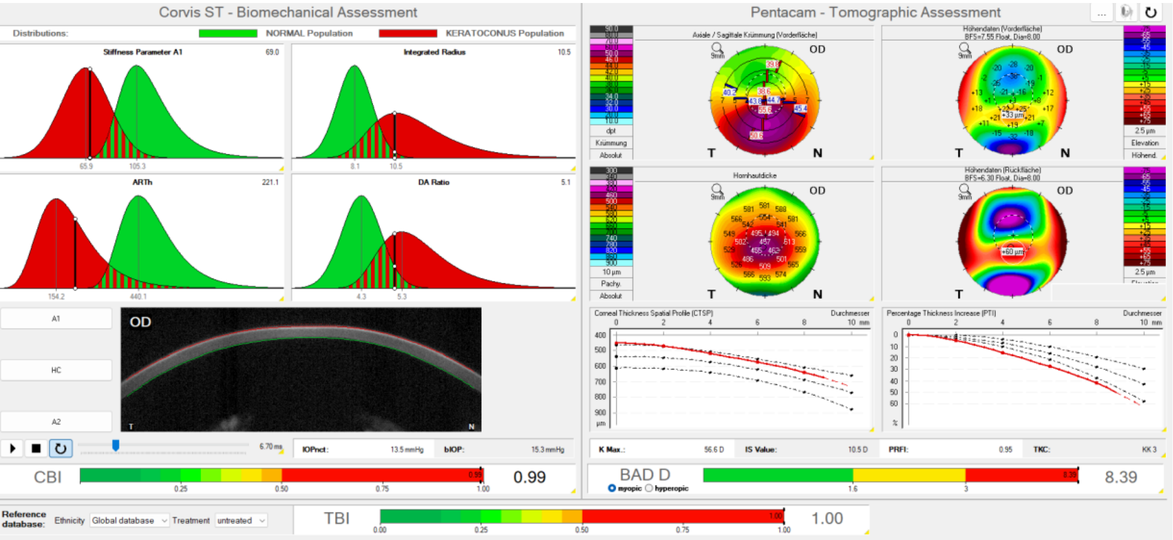
Corvis ST - Biomechanical Assessment
Pentacam - Tomographic Assessment
NORMAL Population
KERATOCONUS Population
OD
CBI
0.99
BAD D
8.39
TBI
1.00

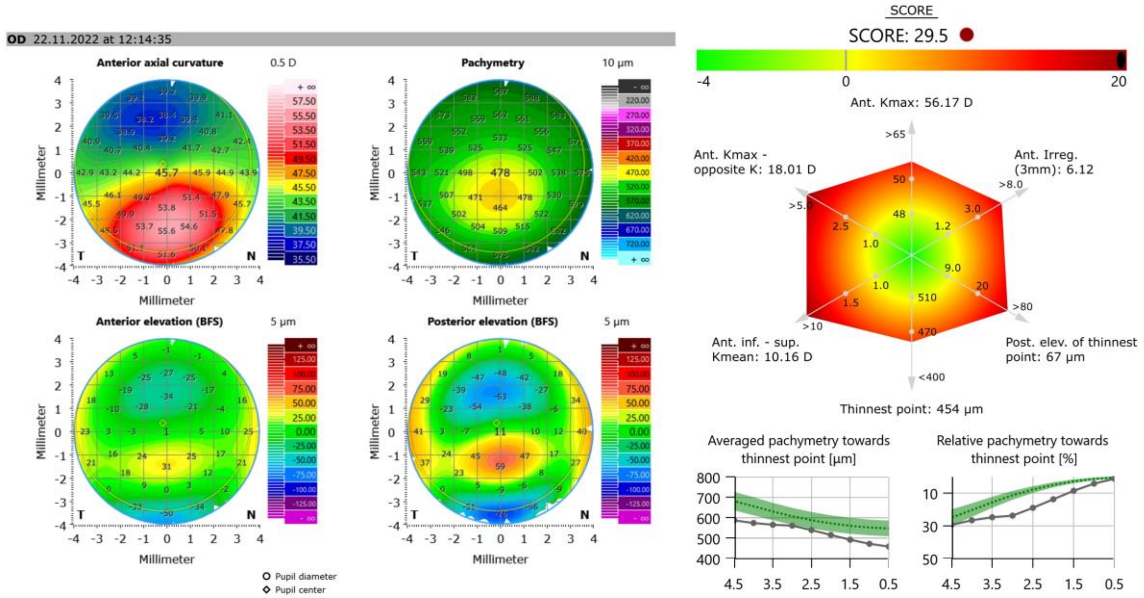
OD 22.11.2022 at 12:14:35
Anterior axial curvature
Pachymetry
Anterior elevation (BFS)
Posterior elevation (BFS)
Millimeter
SCORE
SCORE: 29.5
Ant. Kmax: 56.17 D
Ant. Kmax - opposite K: 18.01 D
Ant. Irreg. (3mm): 6.12
Ant. inf. - sup. Kmean: 10.16 D
Post. elev. of thinnest point: 67 µm
Thinnest point: 454 µm
Averaged pachymetry towards thinnest point [µm]
Relative pachymetry towards thinnest point [%]
Pupil diameter
Pupil center

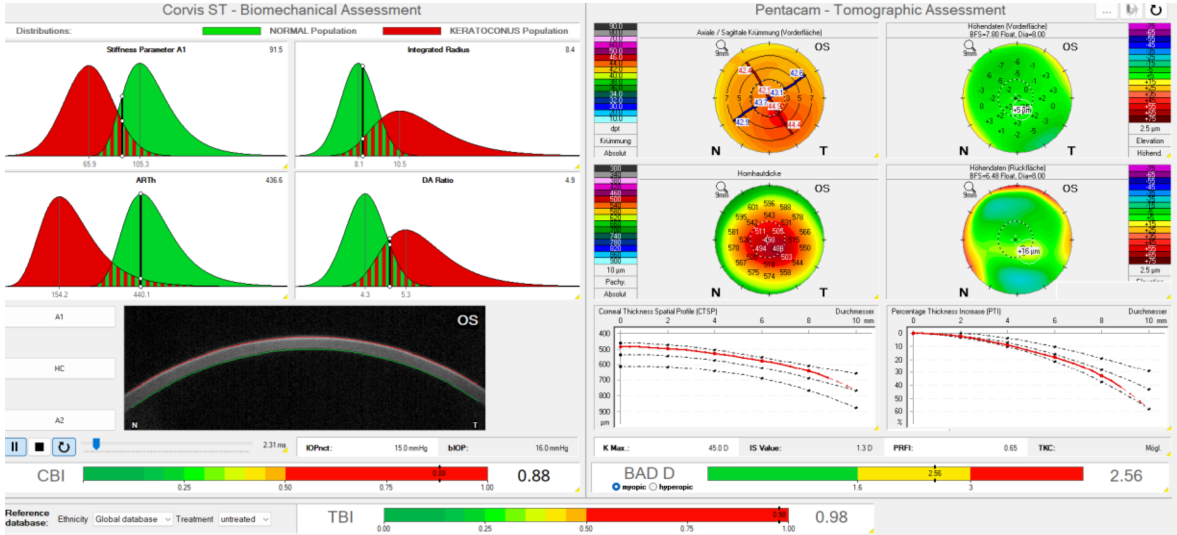
Corvis ST - Biomechanical Assessment
Pentacam - Tomographic Assessment
NORMAL Population
KERATOCONUS Population
OS
CBI
0.88
BAD D
2.56
TBI
0.98

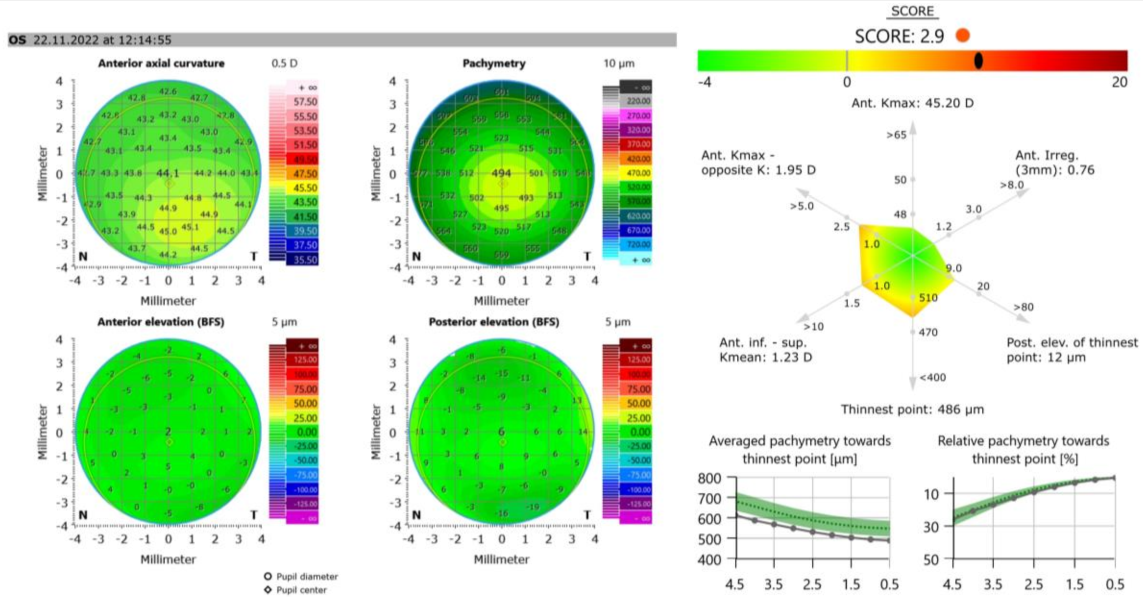
OS 22.11.2022 at 12:14:55
Anterior axial curvature
Pachymetry
Anterior elevation (BFS)
Posterior elevation (BFS)
Millimeter
SCORE
SCORE: 2.9
Ant. Kmax: 45.20 D
Ant. Kmax - opposite K: 1.95 D
Ant. Irreg. (3mm): 0.76
Ant. inf. - sup. Kmean: 1.23 D
Post. elev. of thinnest point: 12 µm
Thinnest point: 486 µm
Averaged pachymetry towards thinnest point [µm]
Relative pachymetry towards thinnest point [%]
Pupil diameter
Pupil center

CASE #60

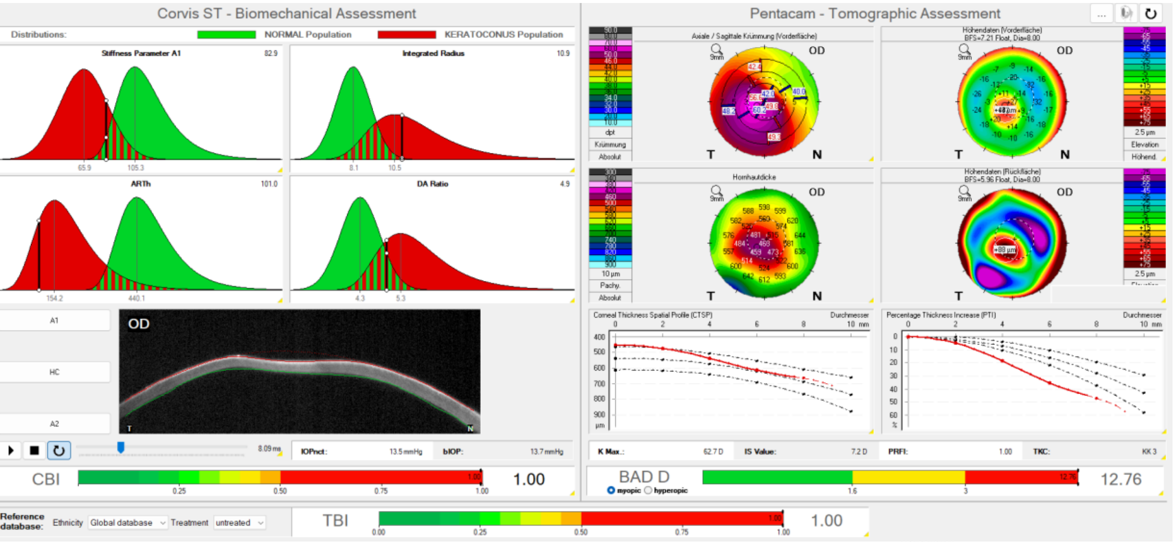

Corvis ST - Biomechanical Assessment
Pentacam - Tomographic Assessment
NORMAL Population
KERATOCONUS Population
Stiffness Parameter A1
Integrated Radius
ARTh
DA Ratio
OD
CBI 1.00
BAD D 12.76
TBI 1.00

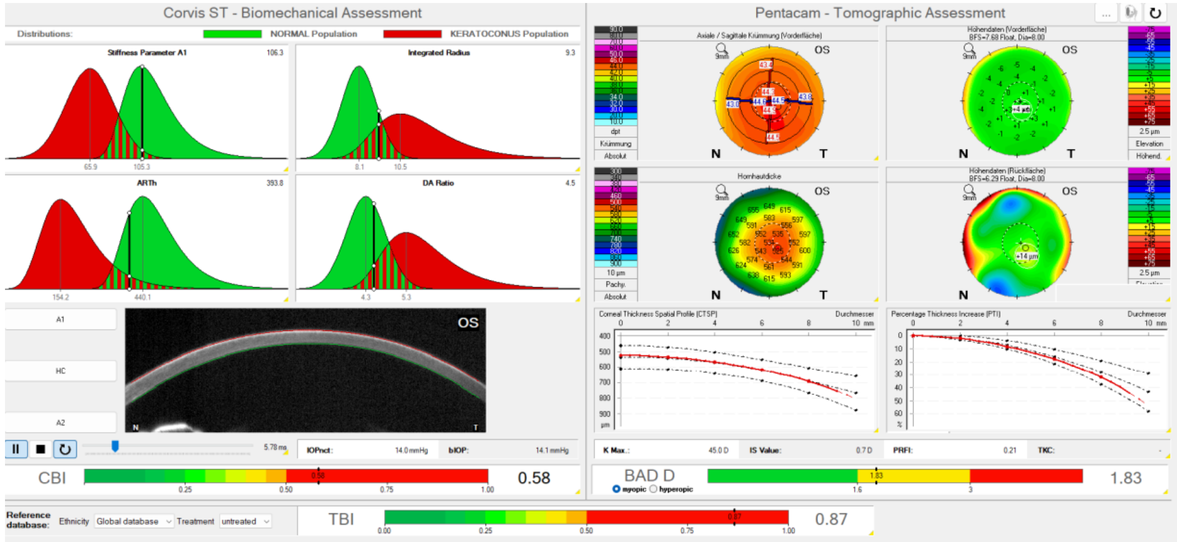

Corvis ST - Biomechanical Assessment
Pentacam - Tomographic Assessment
NORMAL Population
KERATOCONUS Population
Stiffness Parameter A1
Integrated Radius
ARTh
DA Ratio
OS
CBI 0.58
BAD D 1.83
TBI 0.87

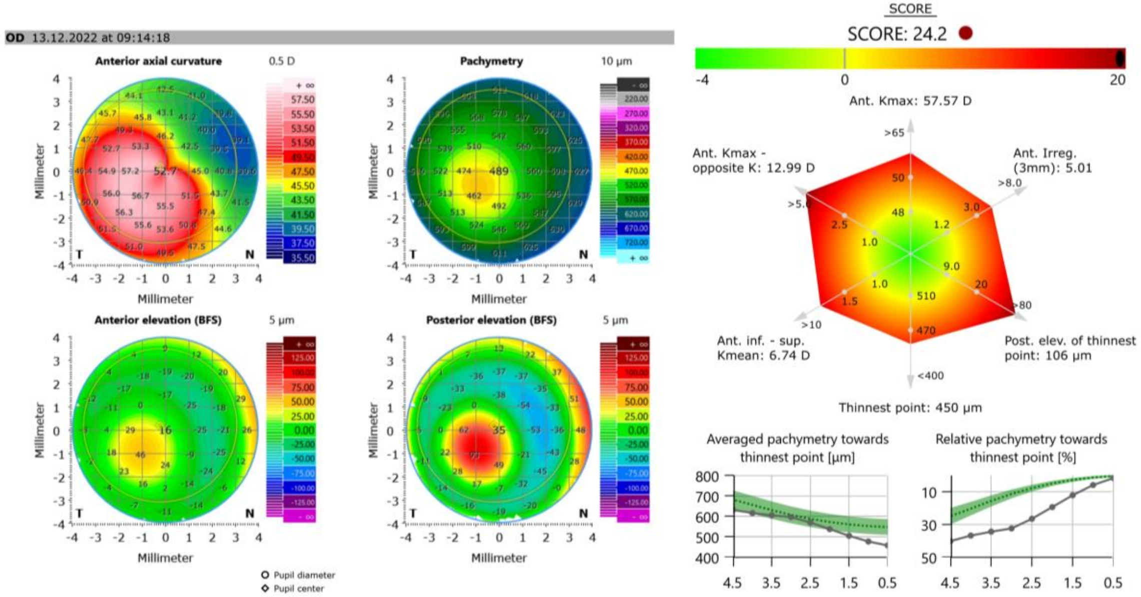

OD 13.12.2022 at 09:14:18
Anterior axial curvature
Pachymetry
Anterior elevation (BFS)
Posterior elevation (BFS)
Millimeter
SCORE
SCORE: 24.2
Ant. Kmax: 57.57 D
Ant. Kmax - opposite K: 12.99 D
Ant. Irreg. (3mm): 5.01
Ant. inf. - sup. Kmean: 6.74 D
Post. elev. of thinnest point: 106 µm
Thinnest point: 450 µm
Averaged pachymetry towards thinnest point [µm]
Relative pachymetry towards thinnest point [%]
Pupil diameter
Pupil center

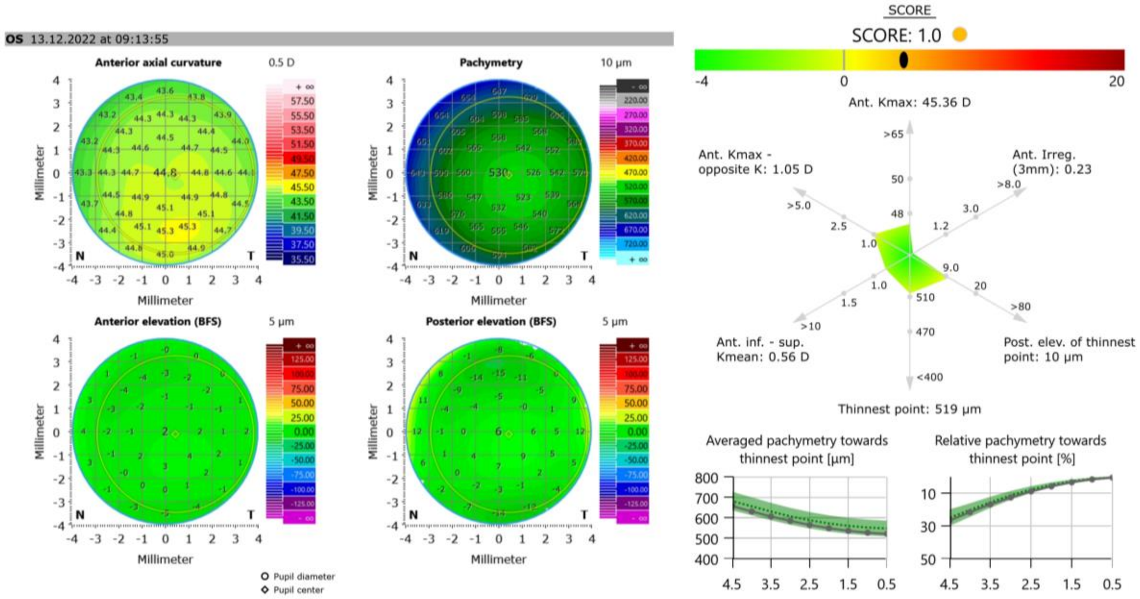

OS 13.12.2022 at 09:13:55
Anterior axial curvature
Pachymetry
Anterior elevation (BFS)
Posterior elevation (BFS)
Millimeter
SCORE
SCORE: 1.0
Ant. Kmax: 45.36 D
Ant. Kmax - opposite K: 1.05 D
Ant. Irreg. (3mm): 0.23
Ant. inf. - sup. Kmean: 0.56 D
Post. elev. of thinnest point: 10 µm
Thinnest point: 519 µm
Averaged pachymetry towards thinnest point [µm]
Relative pachymetry towards thinnest point [%]
Pupil diameter
Pupil center

CASE #61

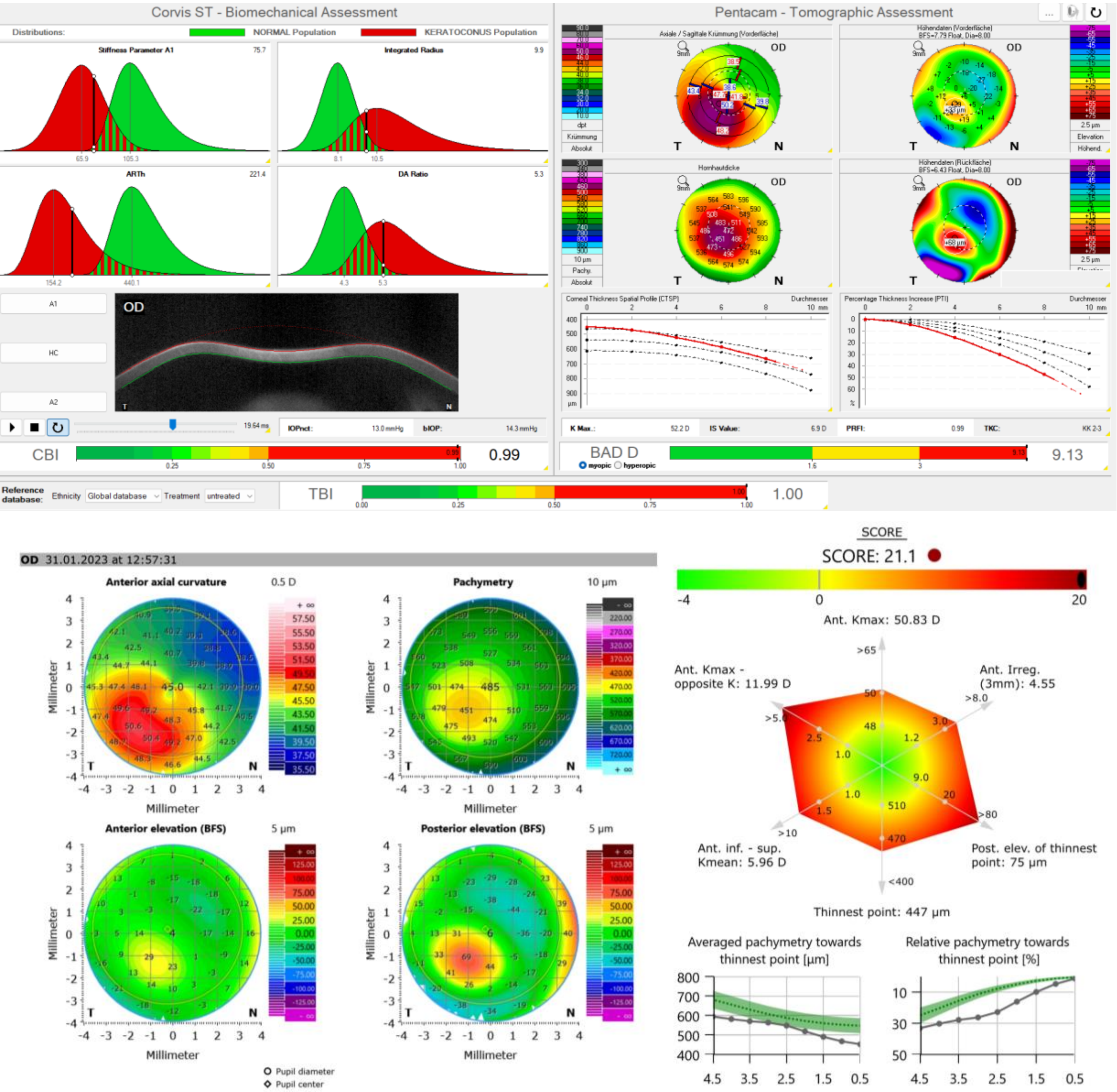
Corvis ST - Biomechanical Assessment
Pentacam - Tomographic Assessment
Distributions: NORMAL Population KERATOCONUS Population
Stiffness Parameter A1
Integrated Radius
ARTh
DA Ratio
OD
CBI 0.99
BAD D 9.13
TBI 1.00
Reference database: Ethnicity Global database Treatment untreated
OD 31.01.2023 at 12:57:31
Anterior axial curvature
Pachymetry
Anterior elevation (BFS)
Posterior elevation (BFS)
Millimeter
SCORE
SCORE: 21.1
Ant. Kmax: 50.83 D
Ant. Kmax - opposite K: 11.99 D
Ant. Irreg. (3mm): 4.55
Ant. inf. - sup. Kmean: 5.96 D
Post. elev. of thinnest point: 75 µm
Thinnest point: 447 µm
Averaged pachymetry towards thinnest point [µm]
Relative pachymetry towards thinnest point [%]
Pupil diameter
Pupil center

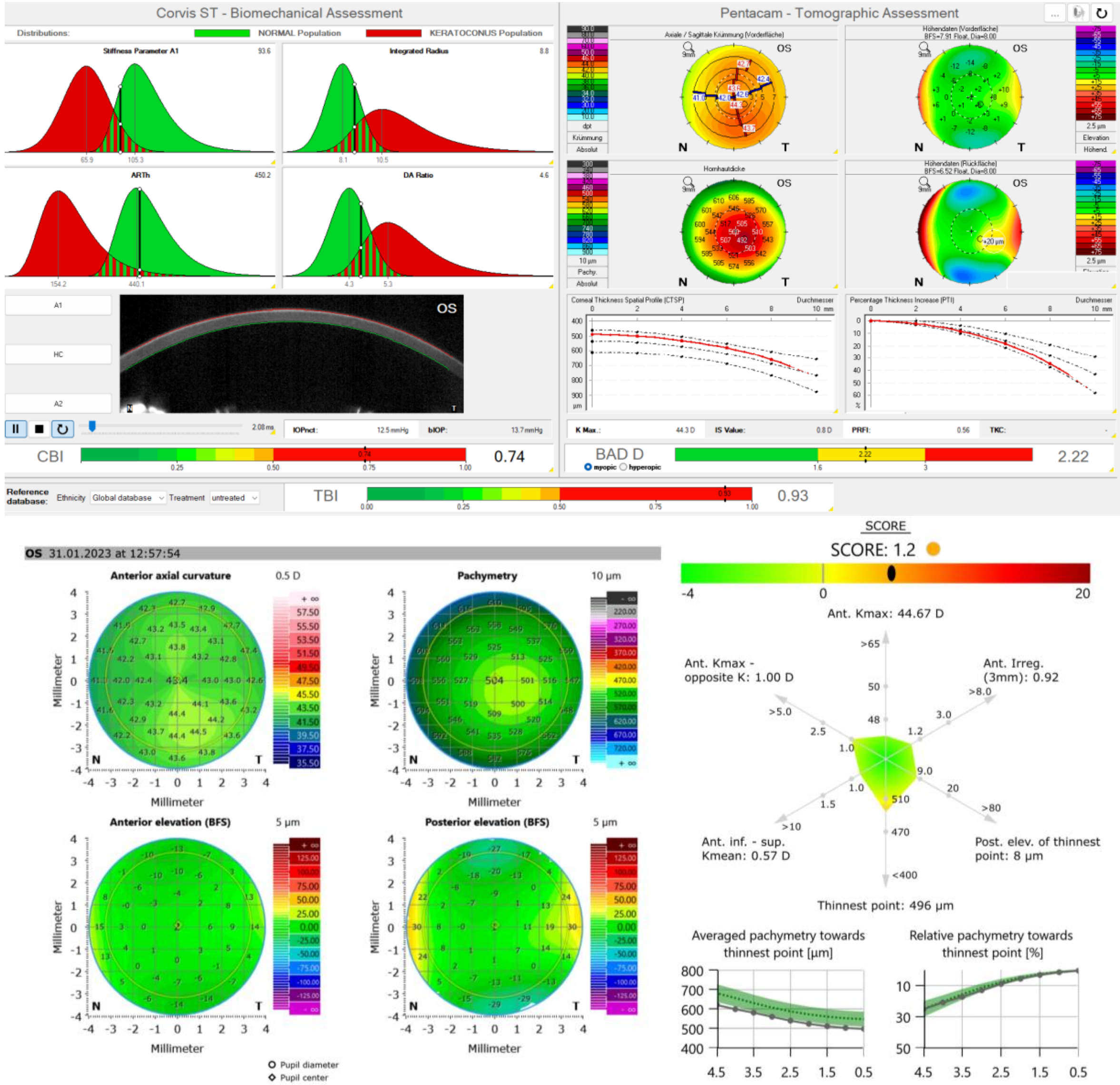
Corvis ST - Biomechanical Assessment
Pentacam - Tomographic Assessment
Distributions: NORMAL Population KERATOCONUS Population
Stiffness Parameter A1
Integrated Radius
ARTh
DA Ratio
OS
CBI 0.74
BAD D 2.22
TBI 0.93
Reference database: Ethnicity Global database Treatment untreated
OS 31.01.2023 at 12:57:54
Anterior axial curvature
Pachymetry
Anterior elevation (BFS)
Posterior elevation (BFS)
Millimeter
SCORE
SCORE: 1.2
Ant. Kmax: 44.67 D
Ant. Kmax - opposite K: 1.00 D
Ant. Irreg. (3mm): 0.92
Ant. inf. - sup. Kmean: 0.57 D
Post. elev. of thinnest point: 8 µm
Thinnest point: 496 µm
Averaged pachymetry towards thinnest point [µm]
Relative pachymetry towards thinnest point [%]
Pupil diameter
Pupil center

CASE #62

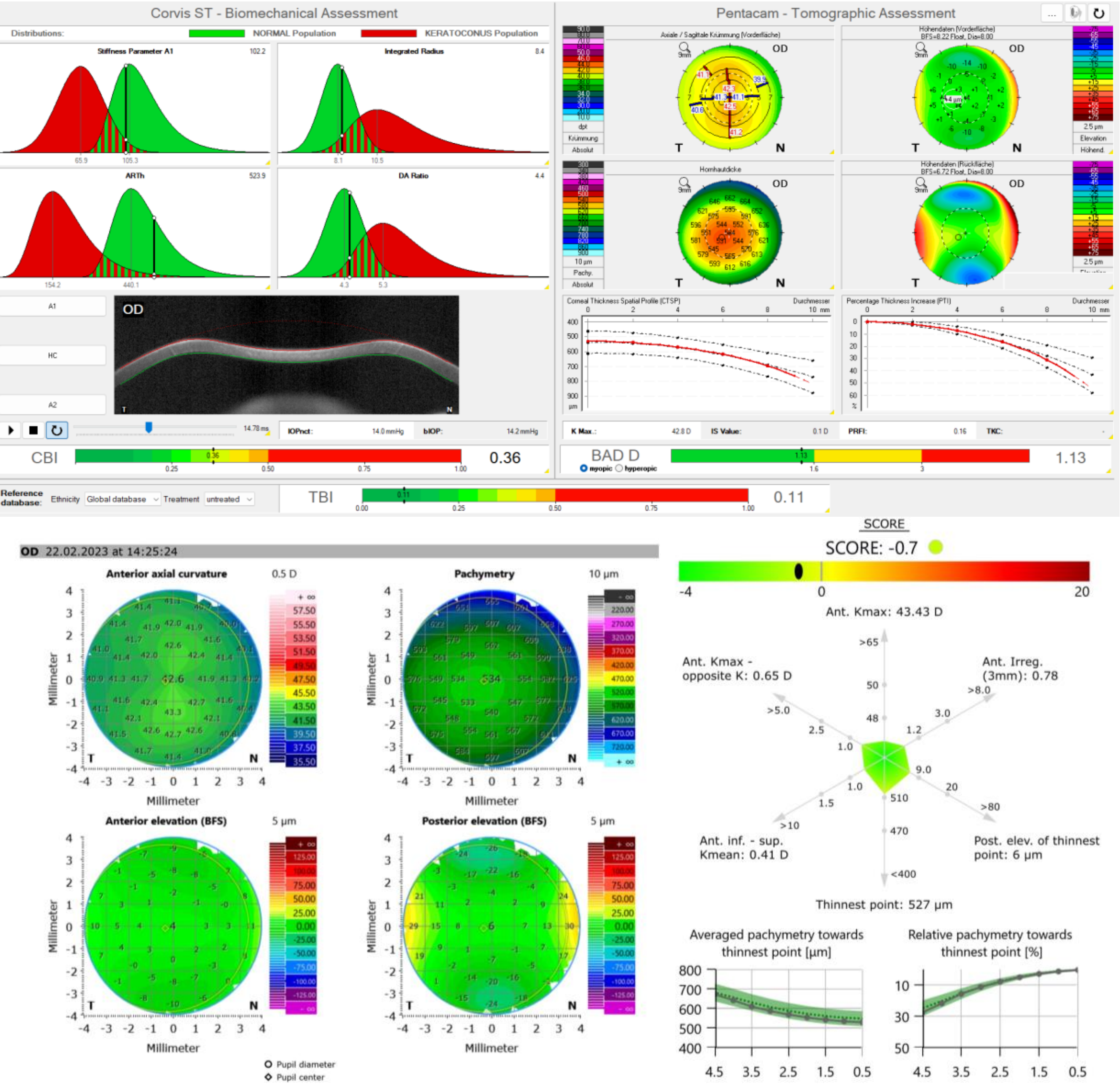
Corvis ST - Biomechanical Assessment
Pentacam - Tomographic Assessment
NORMAL Population
KERATOCONUS Population
Stiffness Parameter A1
Integrated Radius
ARTh
DA Ratio
OD
CBI 0.36
BAD D 1.13
TBI 0.11
OD 22.02.2023 at 14:25:24
Anterior axial curvature
Pachymetry
Anterior elevation (BFS)
Posterior elevation (BFS)
Millimeter
SCORE
SCORE: -0.7
Ant. Kmax: 43.43 D
Ant. Kmax - opposite K: 0.65 D
Ant. Irreg. (3mm): 0.78
Ant. inf. - sup. Kmean: 0.41 D
Post. elev. of thinnest point: 6 µm
Thinnest point: 527 µm
Averaged pachymetry towards thinnest point [µm]
Relative pachymetry towards thinnest point [%]
Pupil diameter
Pupil center

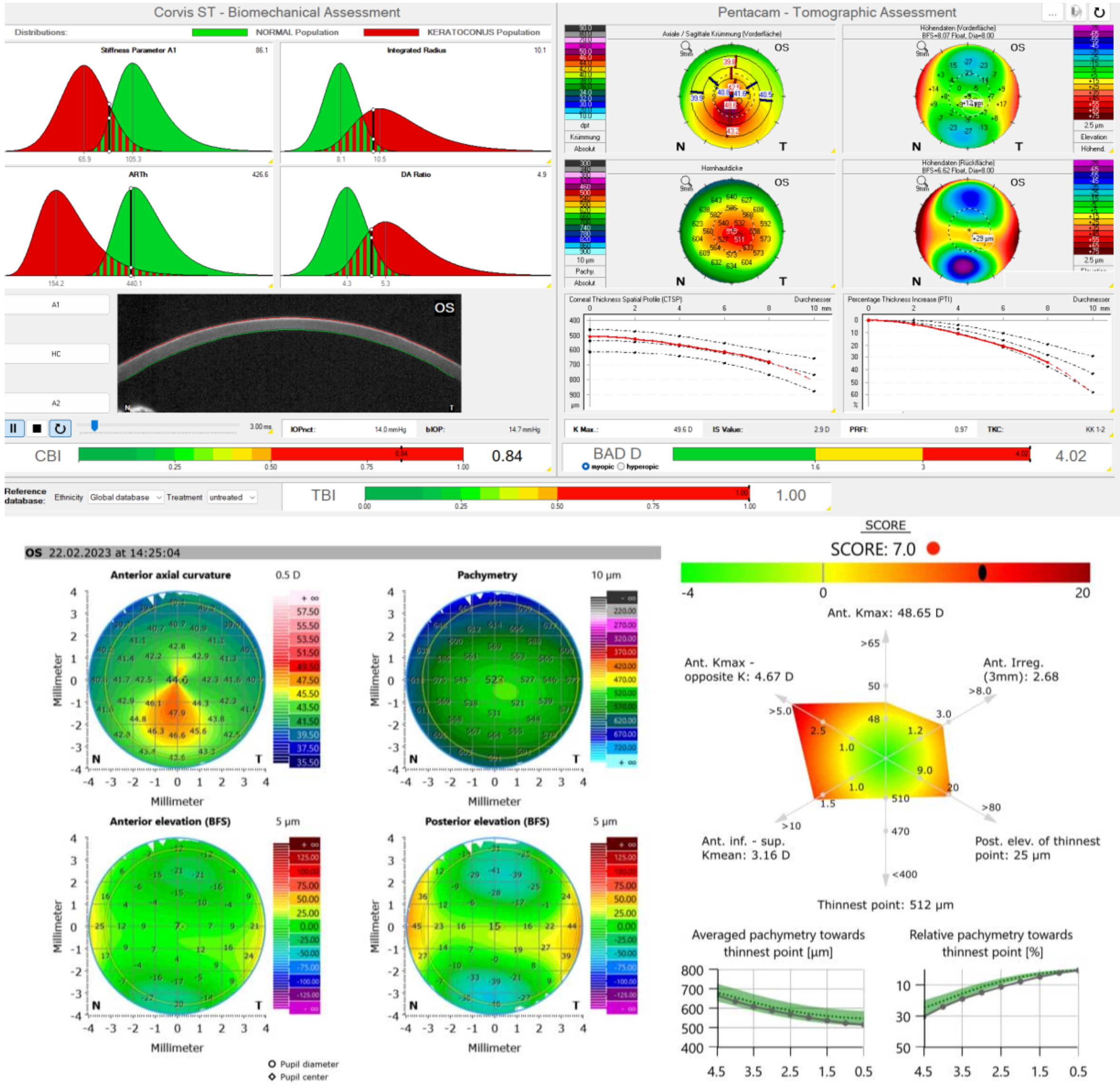
Corvis ST - Biomechanical Assessment
Pentacam - Tomographic Assessment
NORMAL Population
KERATOCONUS Population
Stiffness Parameter A1
Integrated Radius
ARTh
DA Ratio
OS
CBI 0.84
BAD D 4.02
TBI 1.00
OS 22.02.2023 at 14:25:04
Anterior axial curvature
Pachymetry
Anterior elevation (BFS)
Posterior elevation (BFS)
Millimeter
SCORE
SCORE: 7.0
Ant. Kmax: 48.65 D
Ant. Kmax - opposite K: 4.67 D
Ant. Irreg. (3mm): 2.68
Ant. inf. - sup. Kmean: 3.16 D
Post. elev. of thinnest point: 25 µm
Thinnest point: 512 µm
Averaged pachymetry towards thinnest point [µm]
Relative pachymetry towards thinnest point [%]
Pupil diameter
Pupil center

CASE #63

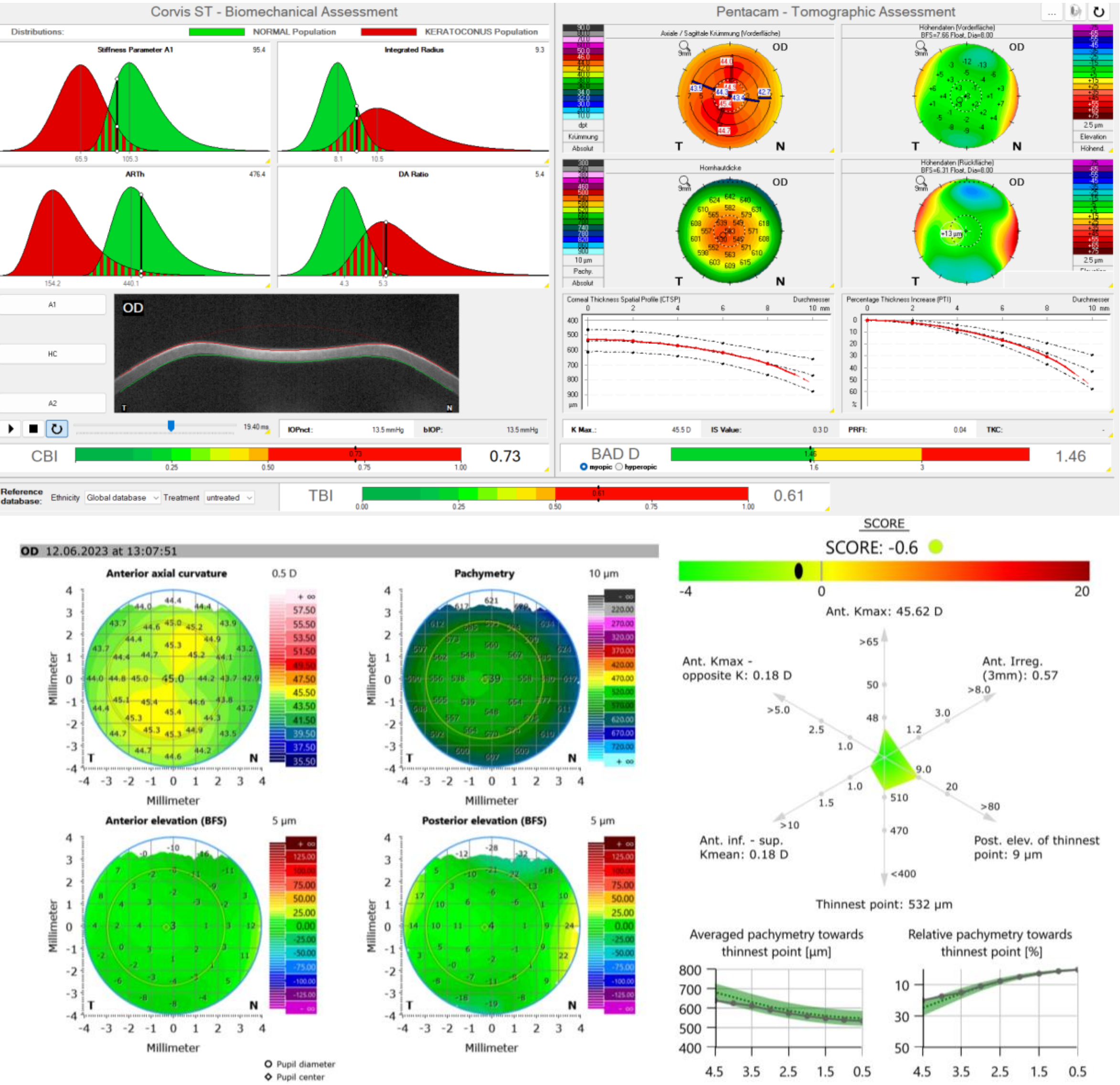

Corvis ST - Biomechanical Assessment
Pentacam - Tomographic Assessment
NORMAL Population
KERATOCONUS Population
OD
CBI 0.73
BAD D 1.46
TBI 0.61
SCORE
SCORE: -0.6
OD 12.06.2023 at 13:07:51
Anterior axial curvature
Pachymetry
Anterior elevation (BFS)
Posterior elevation (BFS)
Ant. Kmax: 45.62 D
Ant. Kmax - opposite K: 0.18 D
Ant. Irreg. (3mm): 0.57
Ant. inf. - sup. Kmean: 0.18 D
Post. elev. of thinnest point: 9 µm
Thinnest point: 532 µm
Averaged pachymetry towards thinnest point [µm]
Relative pachymetry towards thinnest point [%]
Pupil diameter
Pupil center

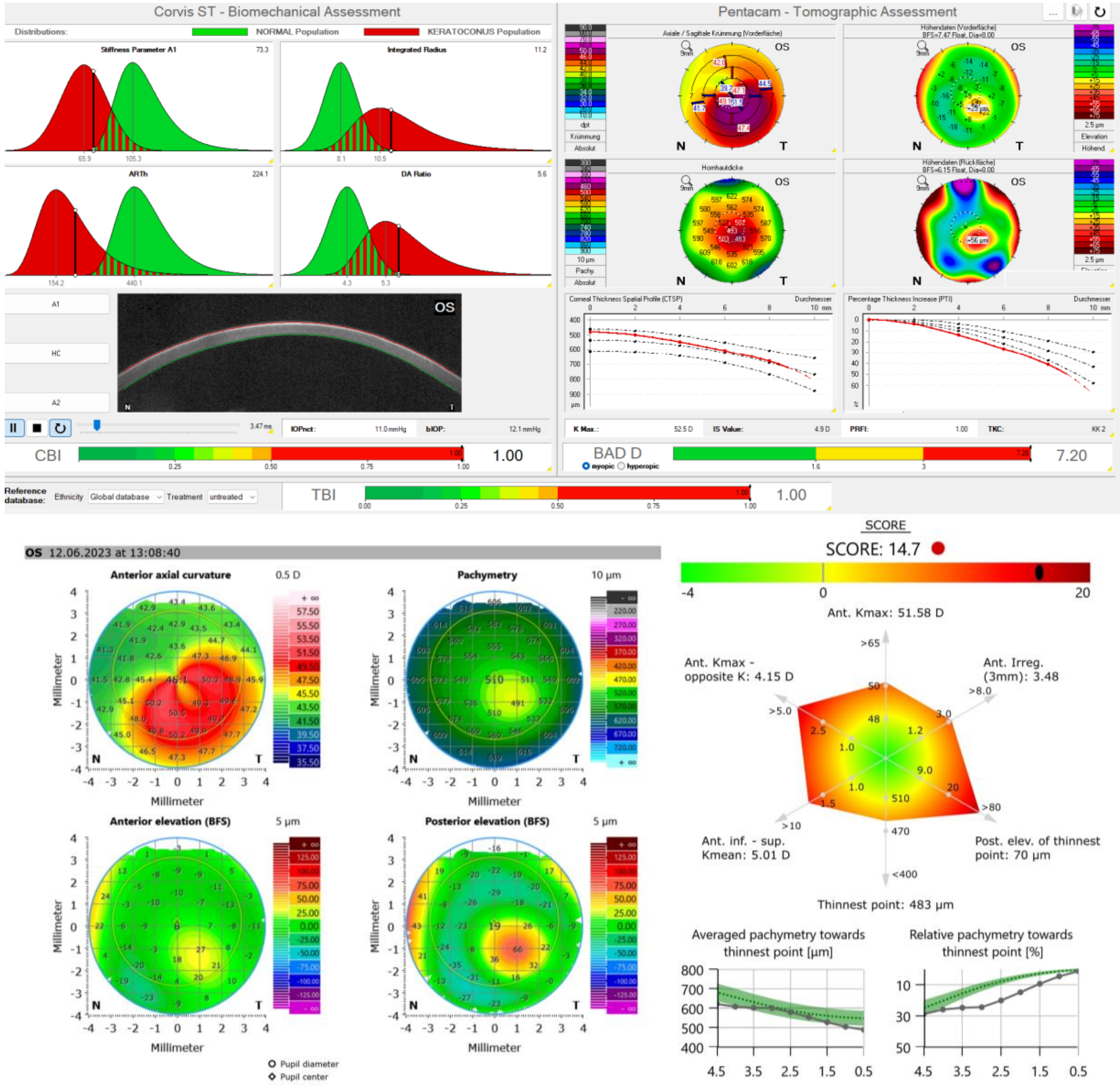

Corvis ST - Biomechanical Assessment
Pentacam - Tomographic Assessment
NORMAL Population
KERATOCONUS Population
OS
CBI 1.00
BAD D 7.20
TBI 1.00
SCORE
SCORE: 14.7
OS 12.06.2023 at 13:08:40
Anterior axial curvature
Pachymetry
Anterior elevation (BFS)
Posterior elevation (BFS)
Ant. Kmax: 51.58 D
Ant. Kmax - opposite K: 4.15 D
Ant. Irreg. (3mm): 3.48
Ant. inf. - sup. Kmean: 5.01 D
Post. elev. of thinnest point: 70 µm
Thinnest point: 483 µm
Averaged pachymetry towards thinnest point [µm]
Relative pachymetry towards thinnest point [%]
Pupil diameter
Pupil center

CASE #64

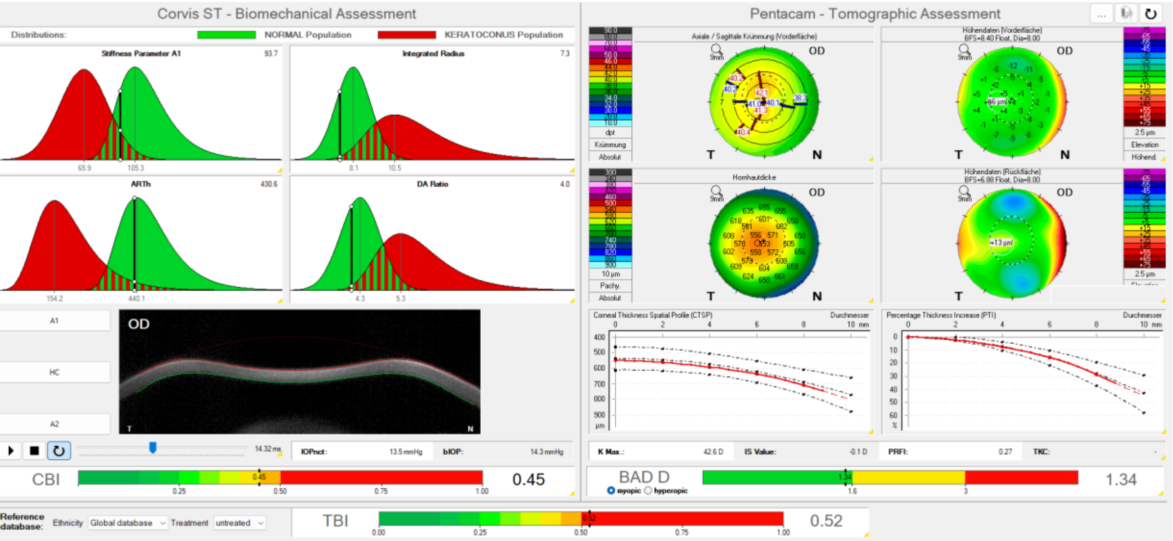
Corvis ST - Biomechanical Assessment
Pentacam - Tomographic Assessment
OD
CBI 0.45
BAD D 1.34
TBI 0.52

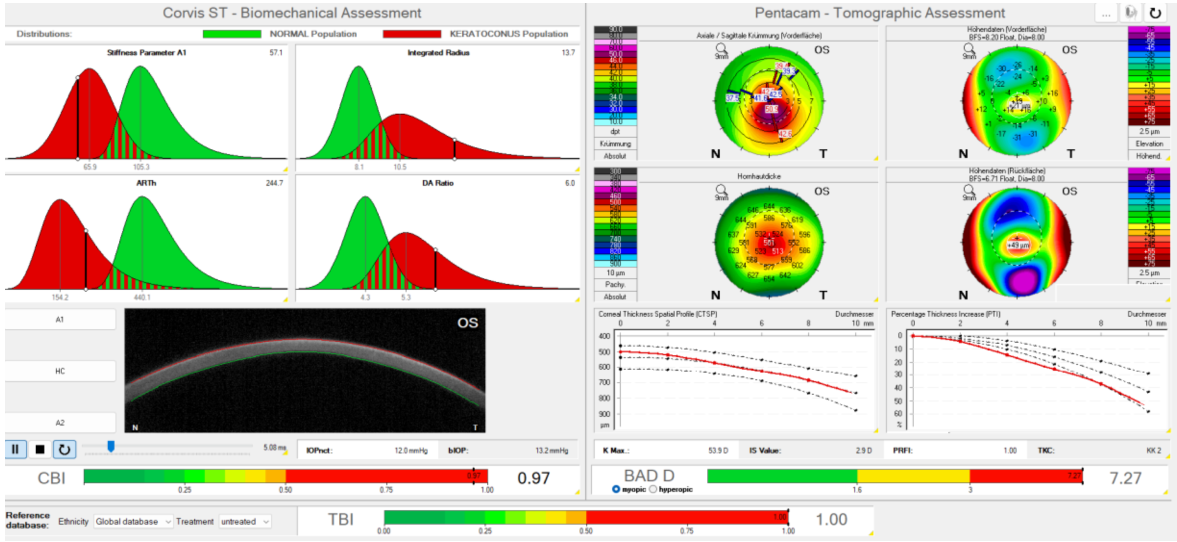
Corvis ST - Biomechanical Assessment
Pentacam - Tomographic Assessment
OS
CBI 0.97
BAD D 7.27
TBI 1.00

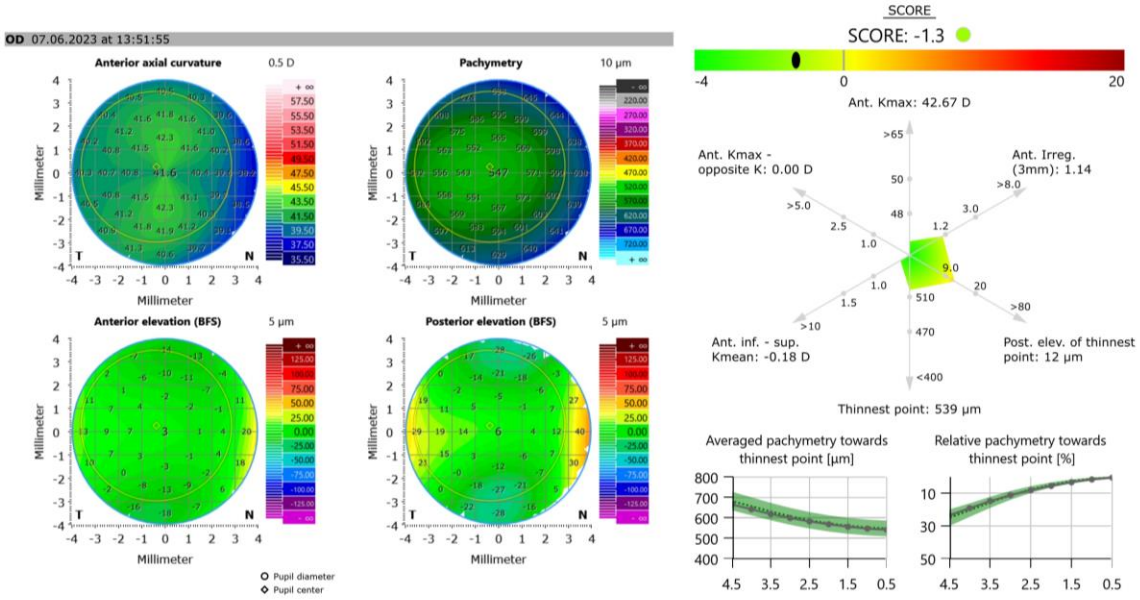
OD 07.06.2023 at 13:51:55
Anterior axial curvature
Pachymetry
Anterior elevation (BFS)
Posterior elevation (BFS)
Millimeter
SCORE
SCORE: -1.3
Ant. Kmax: 42.67 D
Ant. Kmax - opposite K: 0.00 D
Ant. Irreg. (3mm): 1.14
Ant. inf. - sup. Kmean: -0.18 D
Post. elev. of thinnest point: 12 µm
Thinnest point: 539 µm
Averaged pachymetry towards thinnest point [µm]
Relative pachymetry towards thinnest point [%]
Pupil diameter
Pupil center

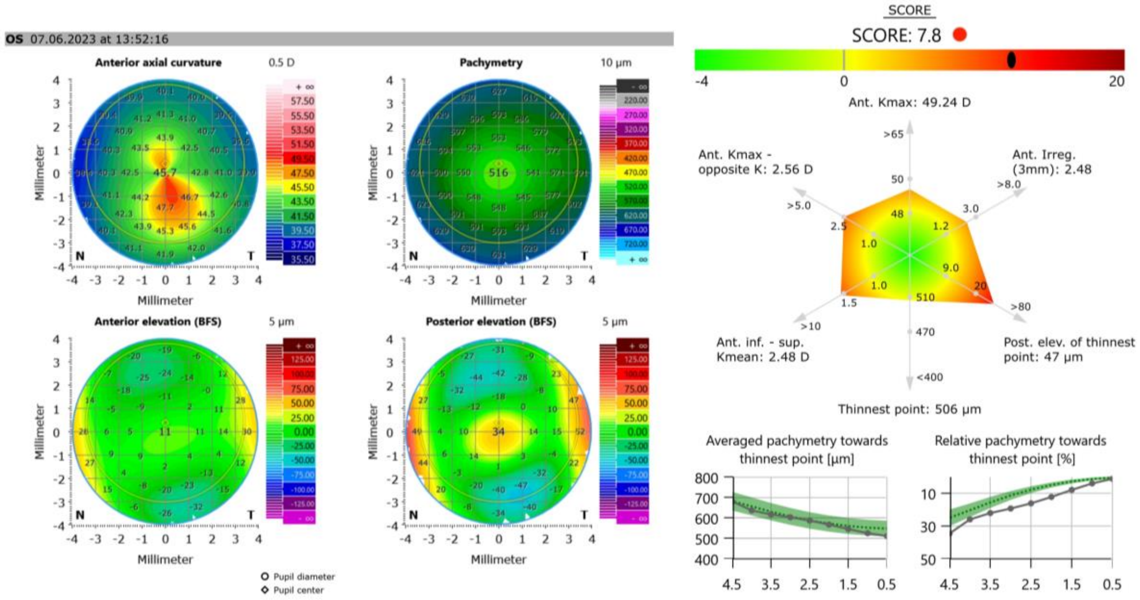
OS 07.06.2023 at 13:52:16
Anterior axial curvature
Pachymetry
Anterior elevation (BFS)
Posterior elevation (BFS)
Millimeter
SCORE
SCORE: 7.8
Ant. Kmax: 49.24 D
Ant. Kmax - opposite K: 2.56 D
Ant. Irreg. (3mm): 2.48
Ant. inf. - sup. Kmean: 2.48 D
Post. elev. of thinnest point: 47 µm
Thinnest point: 506 µm
Averaged pachymetry towards thinnest point [µm]
Relative pachymetry towards thinnest point [%]
Pupil diameter
Pupil center

CASE #65

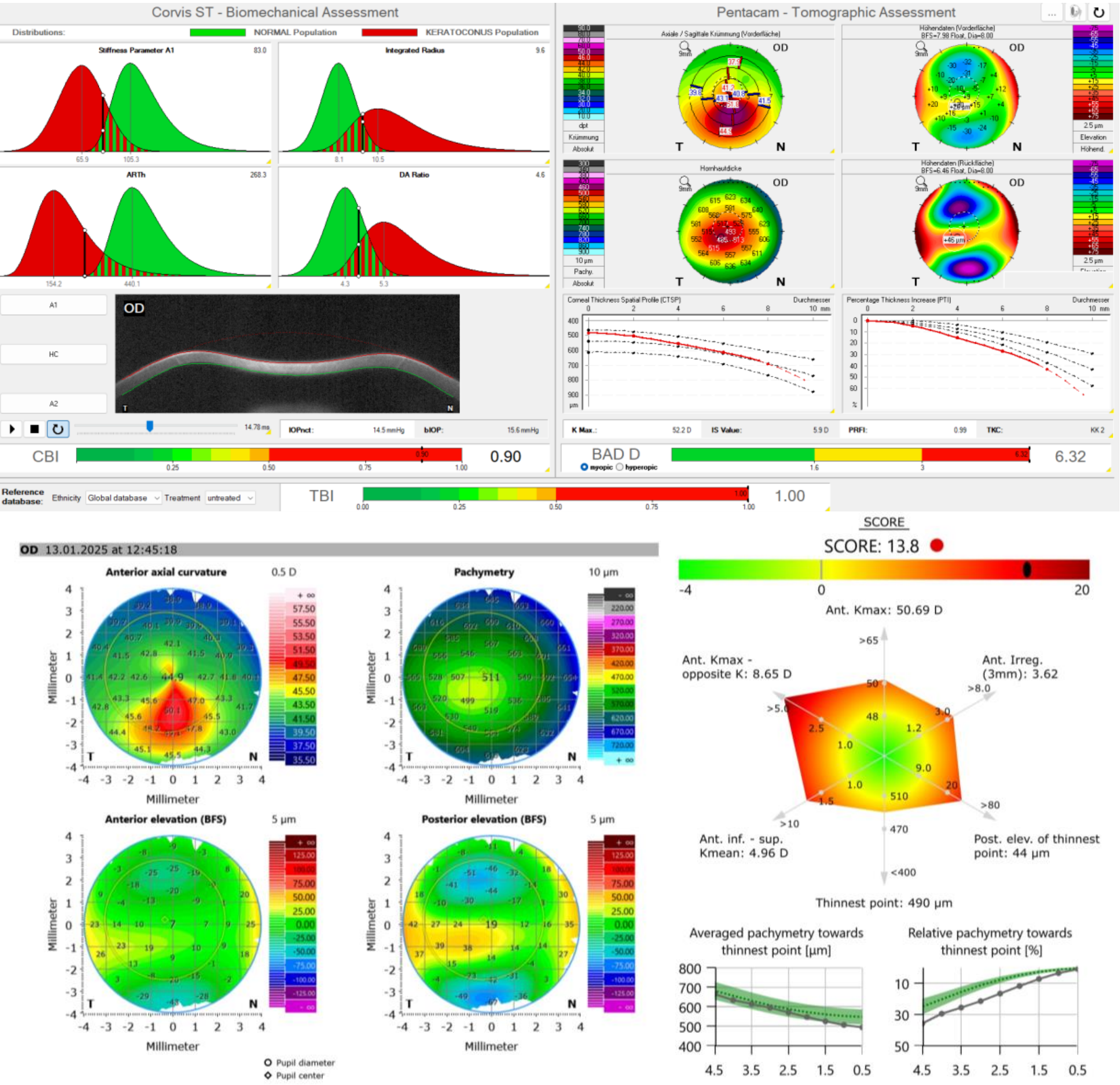

Corvis ST - Biomechanical Assessment
Pentacam - Tomographic Assessment
NORMAL Population
KERATOCONUS Population
Stiffness Parameter A1
Integrated Radius
ARTh
DA Ratio
OD
IOPnct: 14.5 mmHg
bIOP: 15.6 mmHg
K Max: 52.2 D
IS Value: 5.9 D
PRFI: 0.99
TKC: KK 2
CBI
0.90
BAD D
6.32
TBI
1.00
OD 13.01.2025 at 12:45:18
Anterior axial curvature
Pachymetry
Anterior elevation (BFS)
Posterior elevation (BFS)
Millimeter
SCORE
SCORE: 13.8
Ant. Kmax: 50.69 D
Ant. Kmax - opposite K: 8.65 D
Ant. Irreg. (3mm): 3.62
Ant. inf. - sup. Kmean: 4.96 D
Post. elev. of thinnest point: 44 µm
Thinnest point: 490 µm
Averaged pachymetry towards thinnest point [µm]
Relative pachymetry towards thinnest point [%]
Pupil diameter
Pupil center

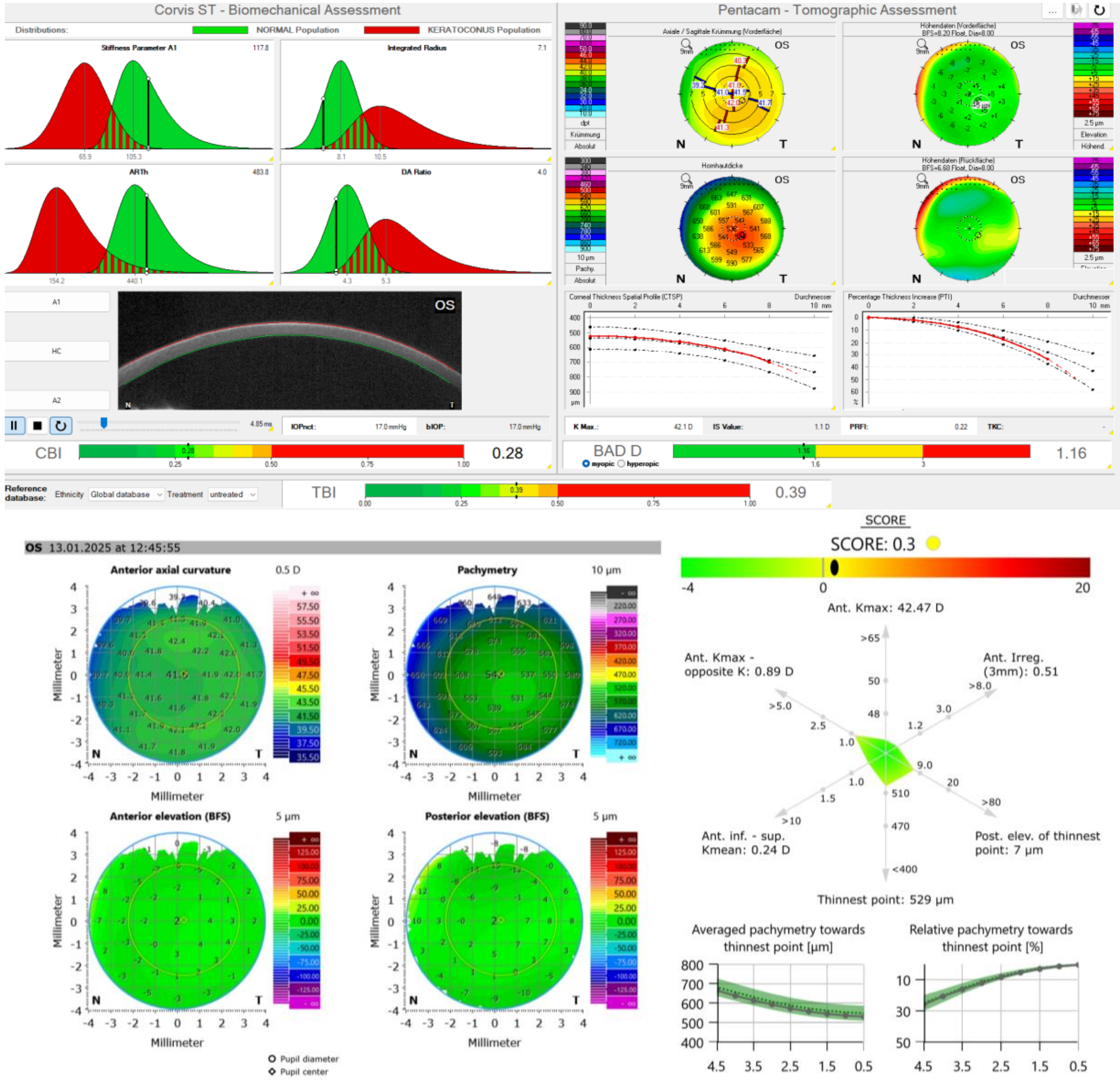

Corvis ST - Biomechanical Assessment
Pentacam - Tomographic Assessment
NORMAL Population
KERATOCONUS Population
Stiffness Parameter A1
Integrated Radius
ARTh
DA Ratio
OS
IOPnct: 17.0 mmHg
bIOP: 17.0 mmHg
K Max: 42.1 D
IS Value: 1.1 D
PRFI: 0.22
CBI
0.28
BAD D
1.16
TBI
0.39
OS 13.01.2025 at 12:45:55
Anterior axial curvature
Pachymetry
Anterior elevation (BFS)
Posterior elevation (BFS)
Millimeter
SCORE
SCORE: 0.3
Ant. Kmax: 42.47 D
Ant. Kmax - opposite K: 0.89 D
Ant. Irreg. (3mm): 0.51
Ant. inf. - sup. Kmean: 0.24 D
Post. elev. of thinnest point: 7 µm
Thinnest point: 529 µm
Averaged pachymetry towards thinnest point [µm]
Relative pachymetry towards thinnest point [%]
Pupil diameter
Pupil center

CASE #66

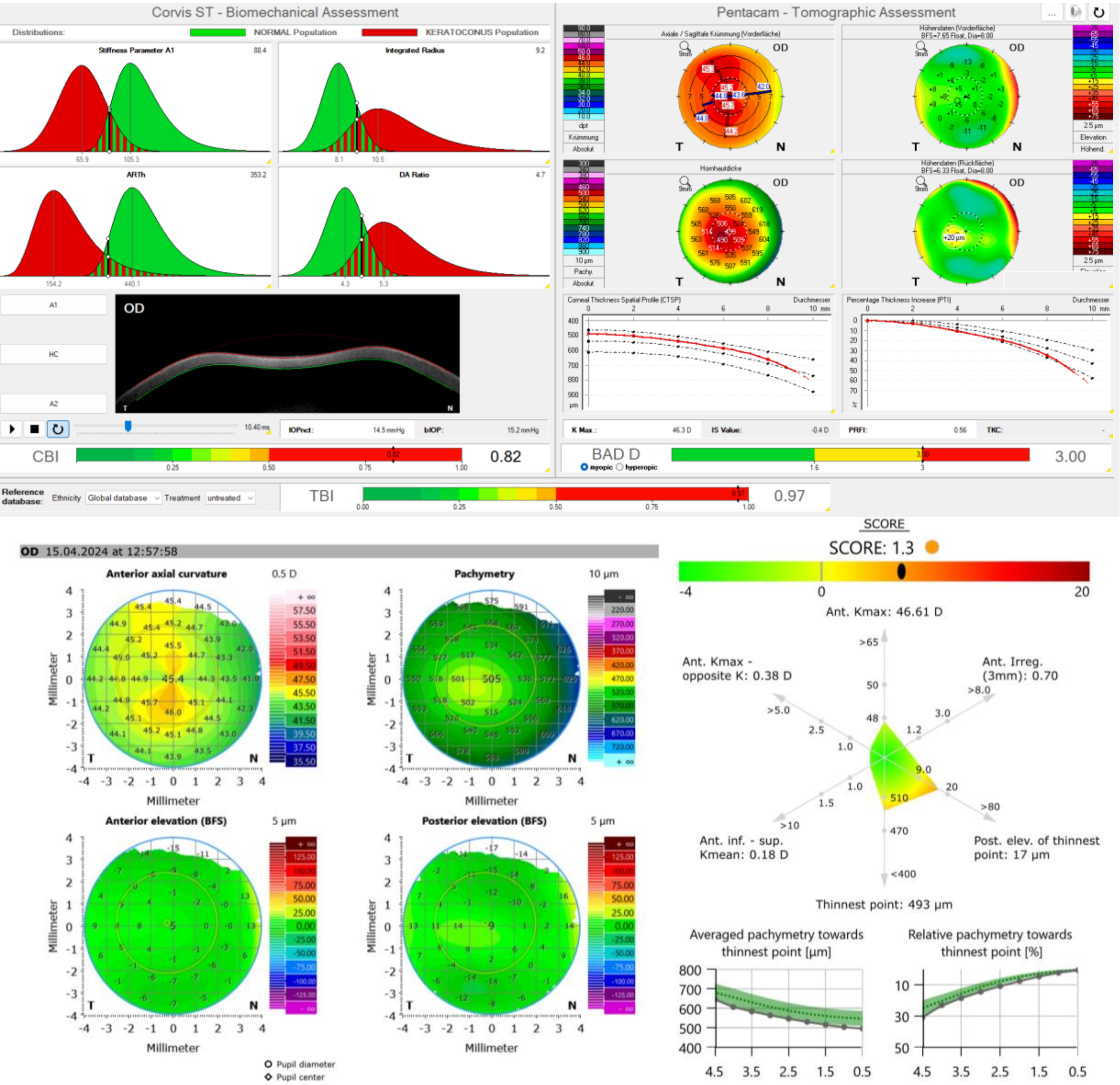

Corvis ST - Biomechanical Assessment
Pentacam - Tomographic Assessment
NORMAL Population
KERATOCONUS Population
Stiffness Parameter A1
Integrated Radius
ARTh
DA Ratio
OD
CBI
0.82
BAD D
3.00
TBI
0.97
OD 15.04.2024 at 12:57:58
Anterior axial curvature
Pachymetry
Anterior elevation (BFS)
Posterior elevation (BFS)
Millimeter
SCORE
SCORE: 1.3
Ant. Kmax: 46.61 D
Ant. Kmax - opposite K: 0.38 D
Ant. Irreg. (3mm): 0.70
Ant. inf. - sup. Kmean: 0.18 D
Post. elev. of thinnest point: 17 µm
Thinnest point: 493 µm
Averaged pachymetry towards thinnest point [µm]
Relative pachymetry towards thinnest point [%]
Pupil diameter
Pupil center

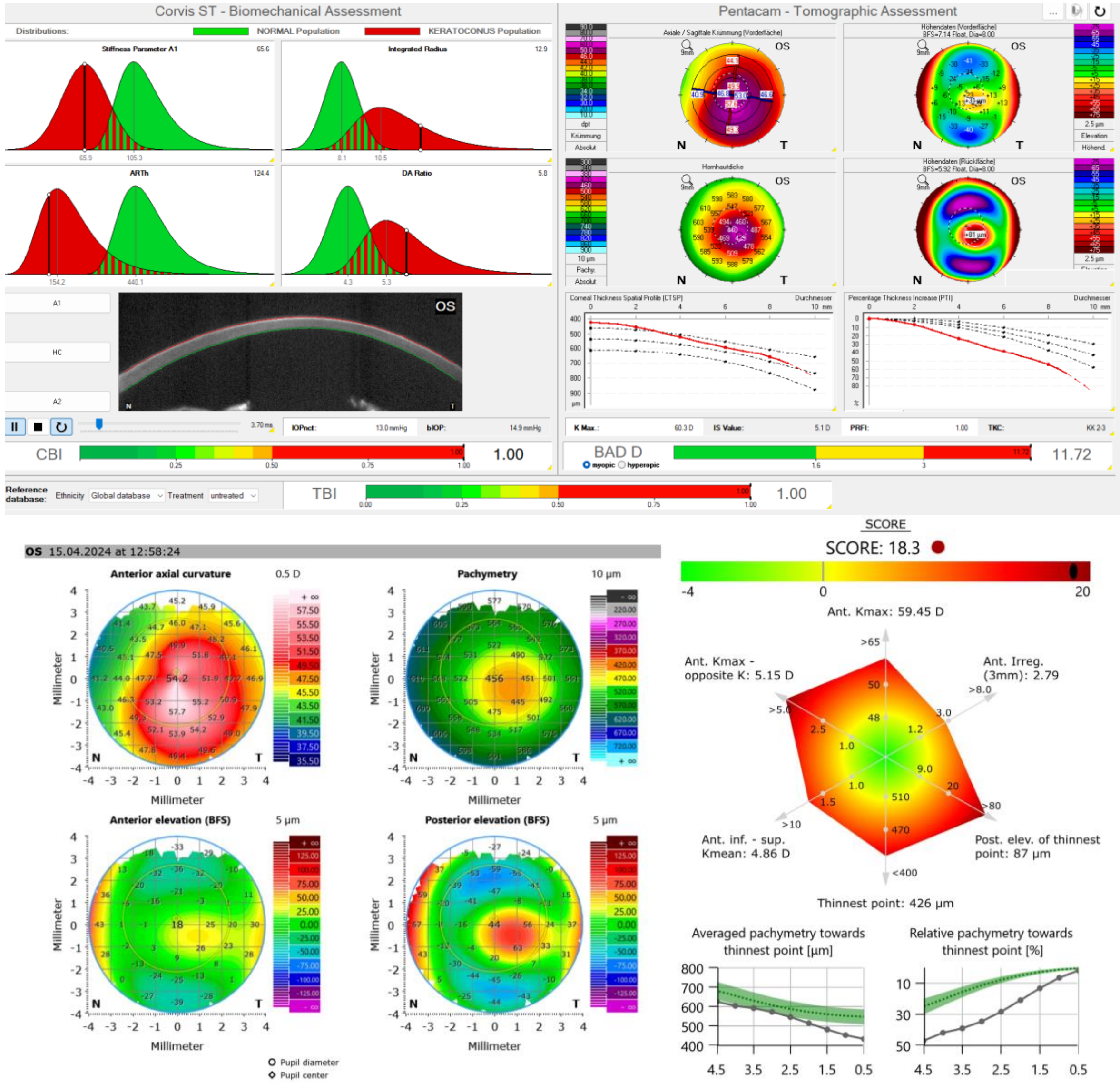

Corvis ST - Biomechanical Assessment
Pentacam - Tomographic Assessment
NORMAL Population
KERATOCONUS Population
Stiffness Parameter A1
Integrated Radius
ARTh
DA Ratio
OS
CBI
1.00
BAD D
11.72
TBI
1.00
OS 15.04.2024 at 12:58:24
Anterior axial curvature
Pachymetry
Anterior elevation (BFS)
Posterior elevation (BFS)
Millimeter
SCORE
SCORE: 18.3
Ant. Kmax: 59.45 D
Ant. Kmax - opposite K: 5.15 D
Ant. Irreg. (3mm): 2.79
Ant. inf. - sup. Kmean: 4.86 D
Post. elev. of thinnest point: 87 µm
Thinnest point: 426 µm
Averaged pachymetry towards thinnest point [µm]
Relative pachymetry towards thinnest point [%]
Pupil diameter
Pupil center

CASE #67

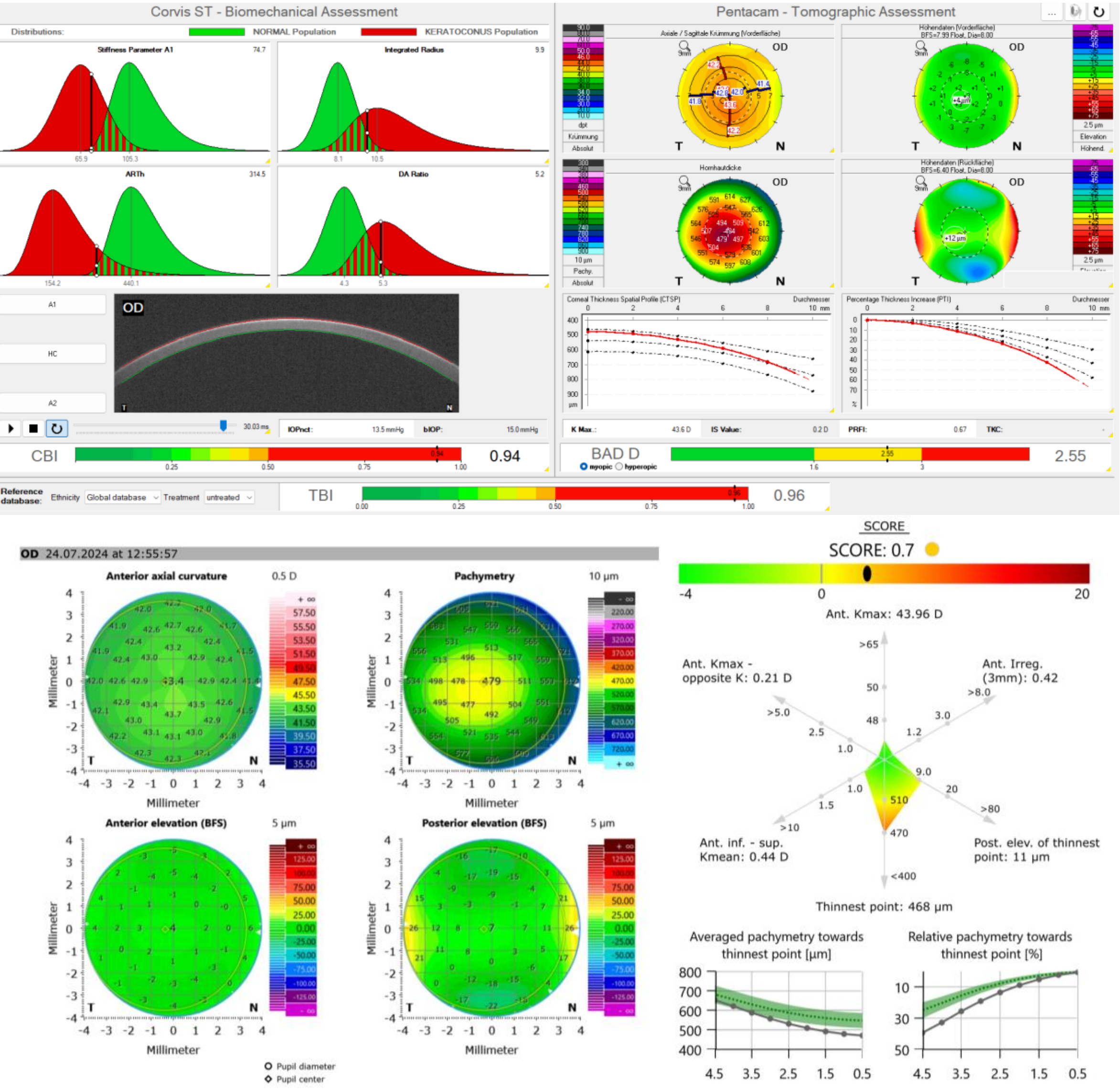

Corvis ST - Biomechanical Assessment
Pentacam - Tomographic Assessment
NORMAL Population
KERATOCONUS Population
Stiffness Parameter A1
Integrated Radius
ARTh
DA Ratio
OD
CBI 0.94
BAD D 2.55
TBI 0.96
OD 24.07.2024 at 12:55:57
Anterior axial curvature
Pachymetry
Anterior elevation (BFS)
Posterior elevation (BFS)
Millimeter
Pupil diameter
Pupil center
SCORE
SCORE: 0.7
Ant. Kmax: 43.96 D
Ant. Kmax - opposite K: 0.21 D
Ant. Irreg. (3mm): 0.42
Ant. inf. - sup. Kmean: 0.44 D
Post. elev. of thinnest point: 11 µm
Thinnest point: 468 µm
Averaged pachymetry towards thinnest point [µm]
Relative pachymetry towards thinnest point [%]

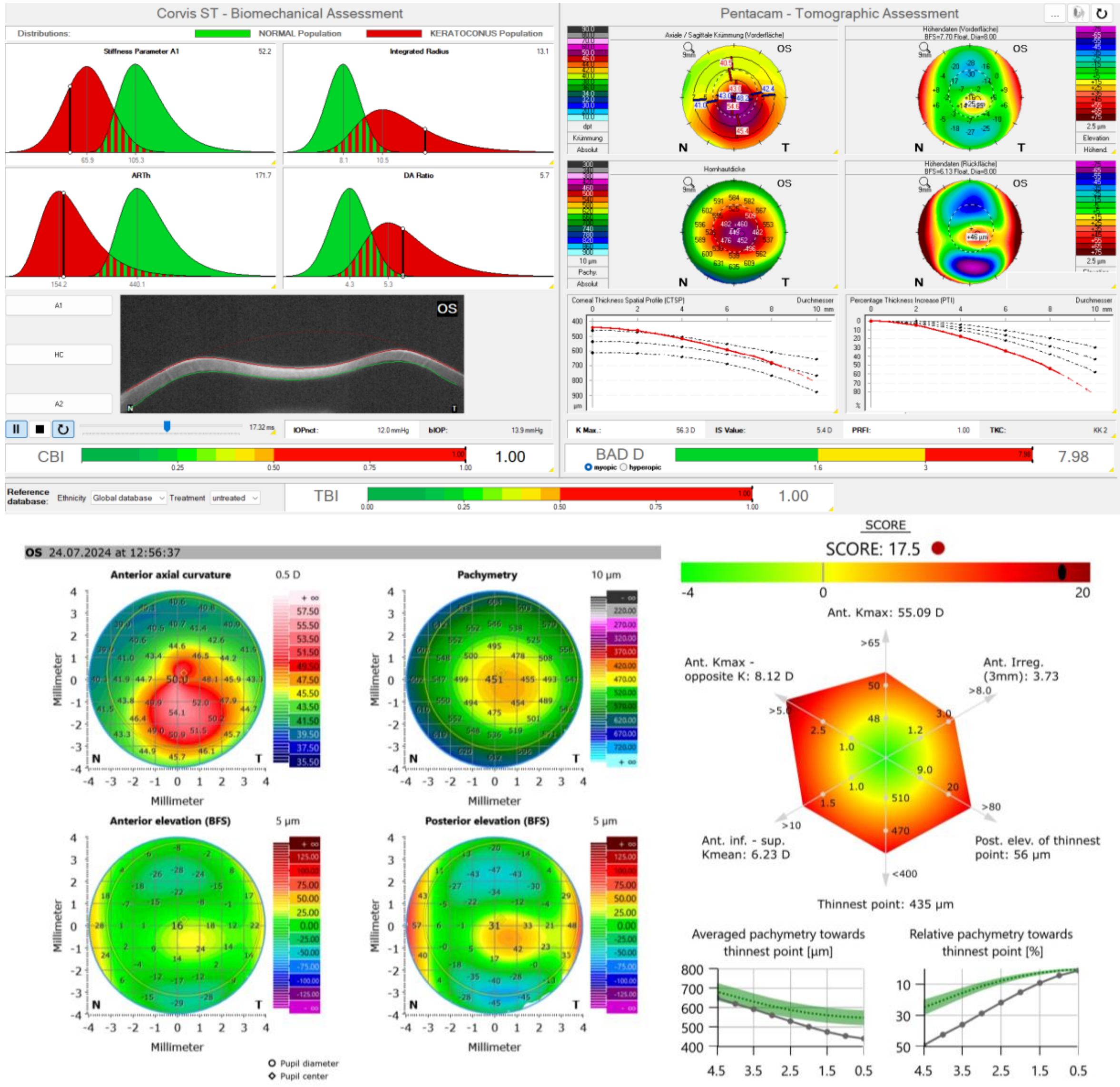

Corvis ST - Biomechanical Assessment
Pentacam - Tomographic Assessment
NORMAL Population
KERATOCONUS Population
Stiffness Parameter A1
Integrated Radius
ARTh
DA Ratio
OS
CBI 1.00
BAD D 7.98
TBI 1.00
OS 24.07.2024 at 12:56:37
Anterior axial curvature
Pachymetry
Anterior elevation (BFS)
Posterior elevation (BFS)
Millimeter
Pupil diameter
Pupil center
SCORE
SCORE: 17.5
Ant. Kmax: 55.09 D
Ant. Kmax - opposite K: 8.12 D
Ant. Irreg. (3mm): 3.73
Ant. inf. - sup. Kmean: 6.23 D
Post. elev. of thinnest point: 56 µm
Thinnest point: 435 µm
Averaged pachymetry towards thinnest point [µm]
Relative pachymetry towards thinnest point [%]

CASE #68

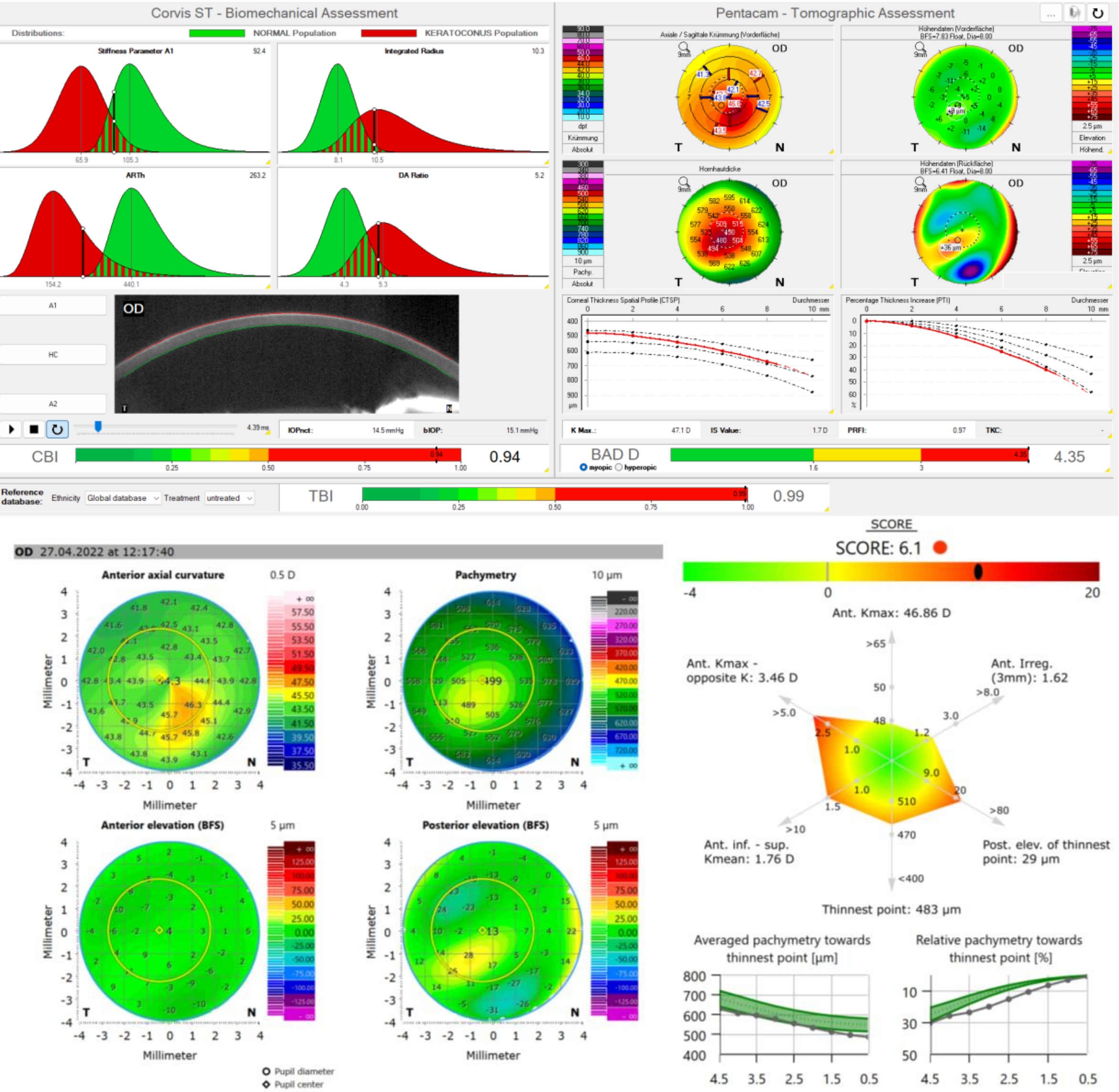
Corvis ST - Biomechanical Assessment
Pentacam - Tomographic Assessment
Distributions:
NORMAL Population
KERATOCONUS Population
Stiffness Parameter A1
Integrated Radius
ARTh
DA Ratio
OD
CBI
0.94
BAD D
4.35
TBI
0.99
Reference database:
SCORE
OD 27.04.2022 at 12:17:40
Anterior axial curvature
Pachymetry
Anterior elevation (BFS)
Posterior elevation (BFS)
Millimeter
SCORE: 6.1
Ant. Kmax: 46.86 D
Ant. Kmax - opposite K: 3.46 D
Ant. Irreg. (3mm): 1.62
Ant. inf. - sup. Kmean: 1.76 D
Post. elev. of thinnest point: 29 µm
Thinnest point: 483 µm
Averaged pachymetry towards thinnest point [µm]
Relative pachymetry towards thinnest point [%]
Pupil diameter
Pupil center

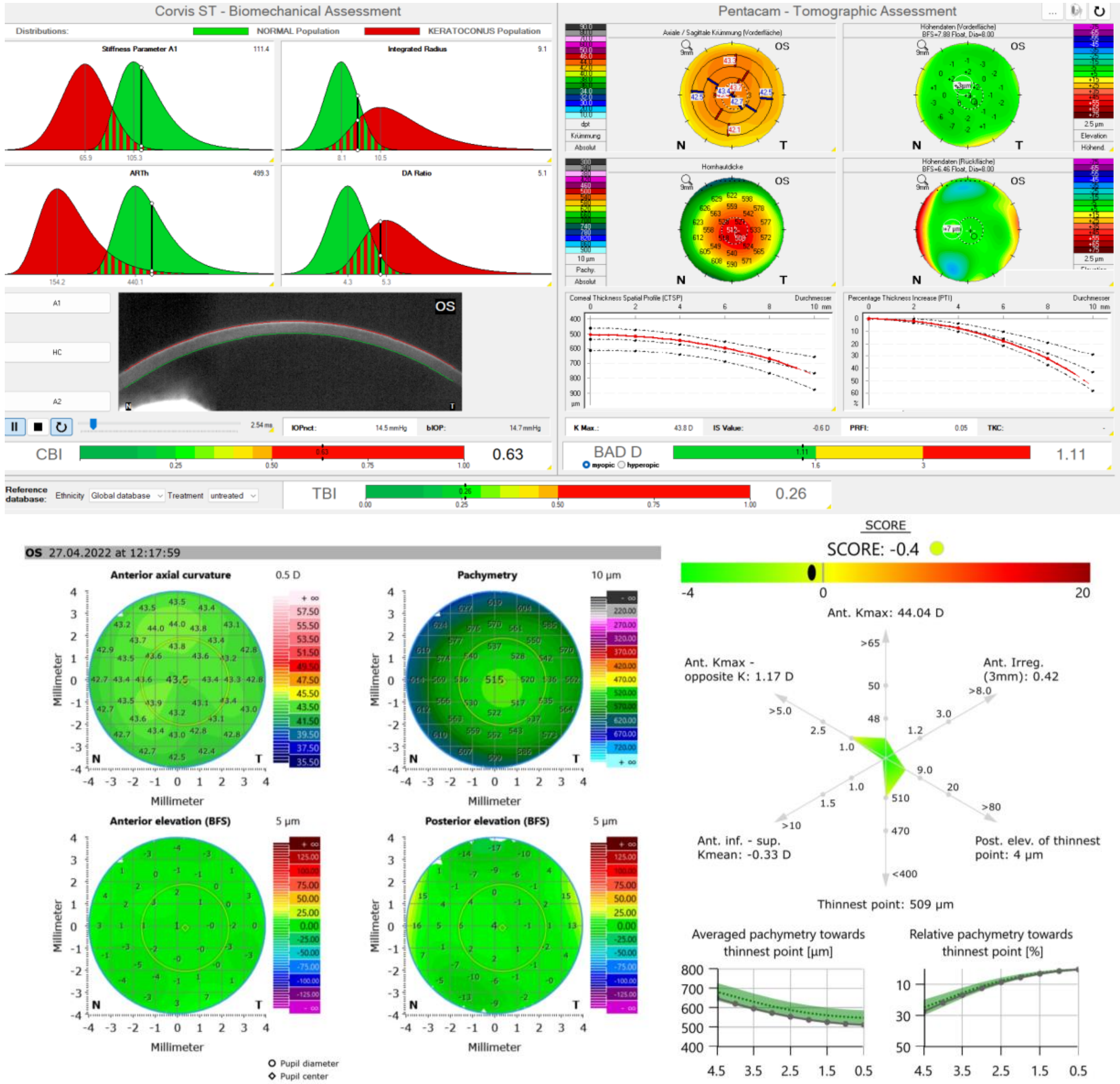
Corvis ST - Biomechanical Assessment
Pentacam - Tomographic Assessment
Distributions:
NORMAL Population
KERATOCONUS Population
Stiffness Parameter A1
Integrated Radius
ARTh
DA Ratio
OS
CBI
0.63
BAD D
1.11
TBI
0.26
Reference database:
SCORE
OS 27.04.2022 at 12:17:59
Anterior axial curvature
Pachymetry
Anterior elevation (BFS)
Posterior elevation (BFS)
Millimeter
SCORE: -0.4
Ant. Kmax: 44.04 D
Ant. Kmax - opposite K: 1.17 D
Ant. Irreg. (3mm): 0.42
Ant. inf. - sup. Kmean: -0.33 D
Post. elev. of thinnest point: 4 µm
Thinnest point: 509 µm
Averaged pachymetry towards thinnest point [µm]
Relative pachymetry towards thinnest point [%]
Pupil diameter
Pupil center

CASE #69

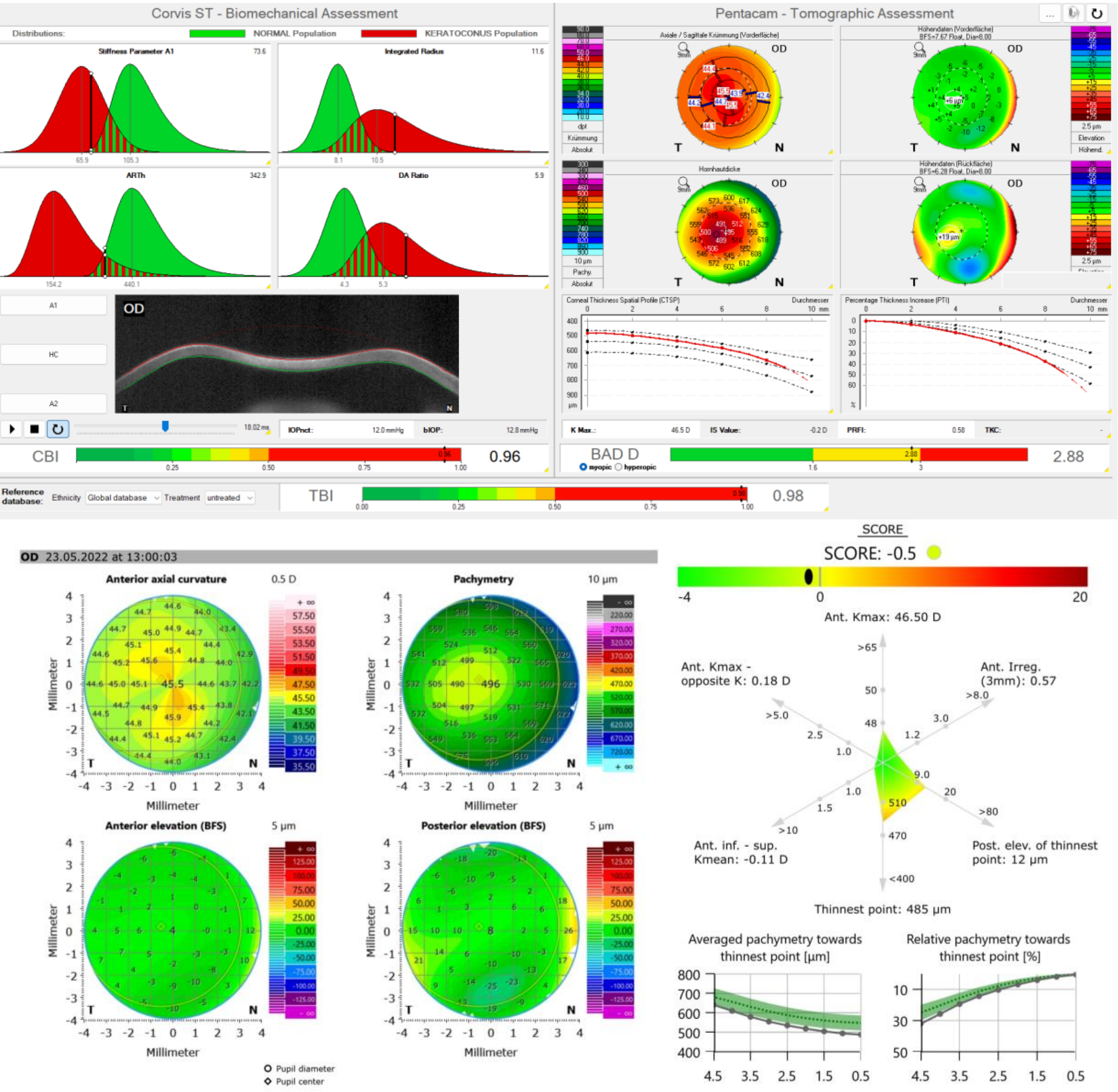

Excluded for analysis due to prior CXL

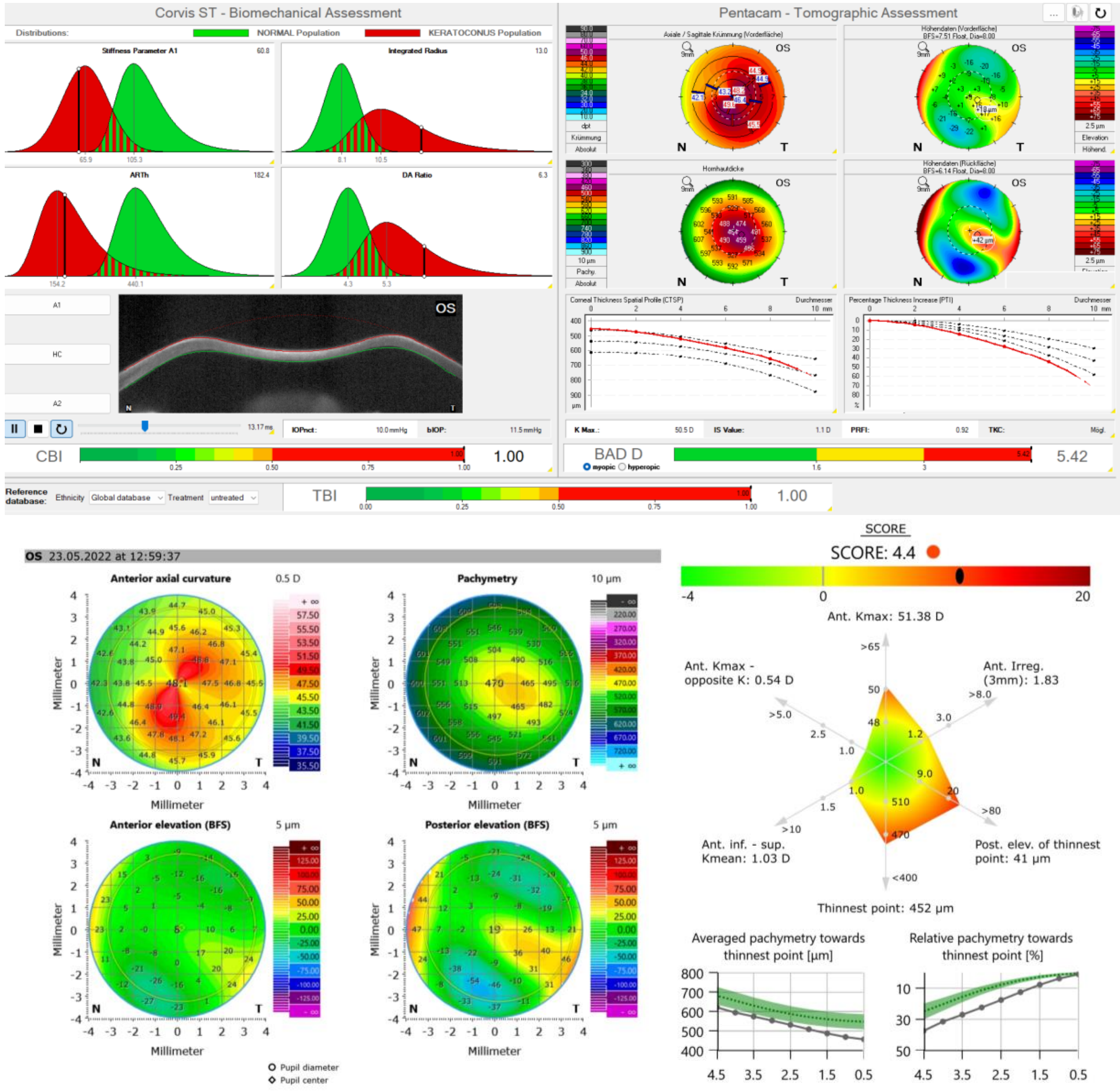

CASE #70

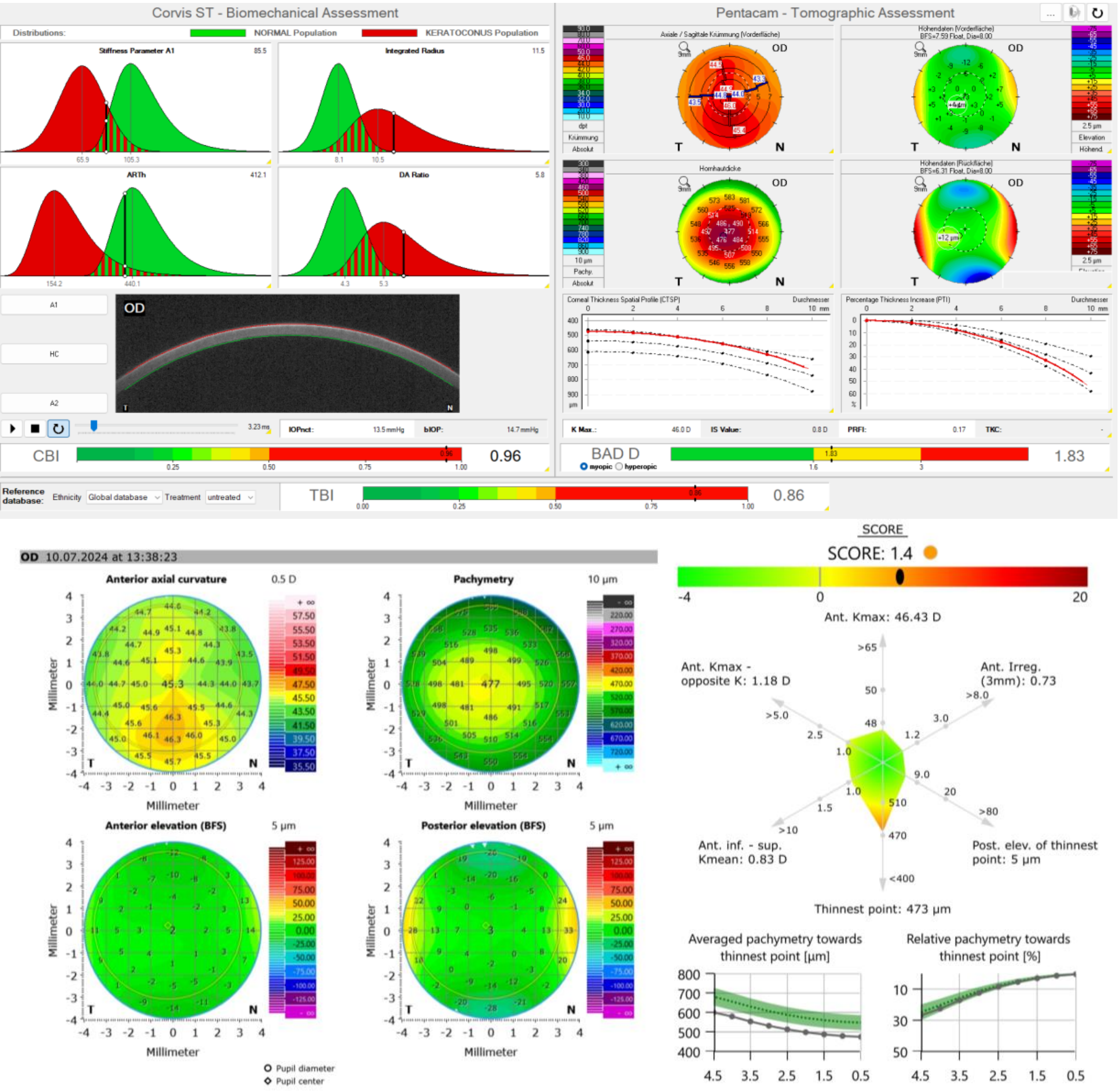

Corvis ST - Biomechanical Assessment
Pentacam - Tomographic Assessment
OD
CBI 0.96
BAD D 1.83
TBI 0.86
OD 10.07.2024 at 13:38:23
Anterior axial curvature
Pachymetry
Anterior elevation (BFS)
Posterior elevation (BFS)
SCORE
SCORE: 1.4
Ant. Kmax: 46.43 D
Ant. Kmax - opposite K: 1.18 D
Ant. Irreg. (3mm): 0.73
Ant. inf. - sup. Kmean: 0.83 D
Post. elev. of thinnest point: 5 µm
Thinnest point: 473 µm
Averaged pachymetry towards thinnest point [µm]
Relative pachymetry towards thinnest point [%]

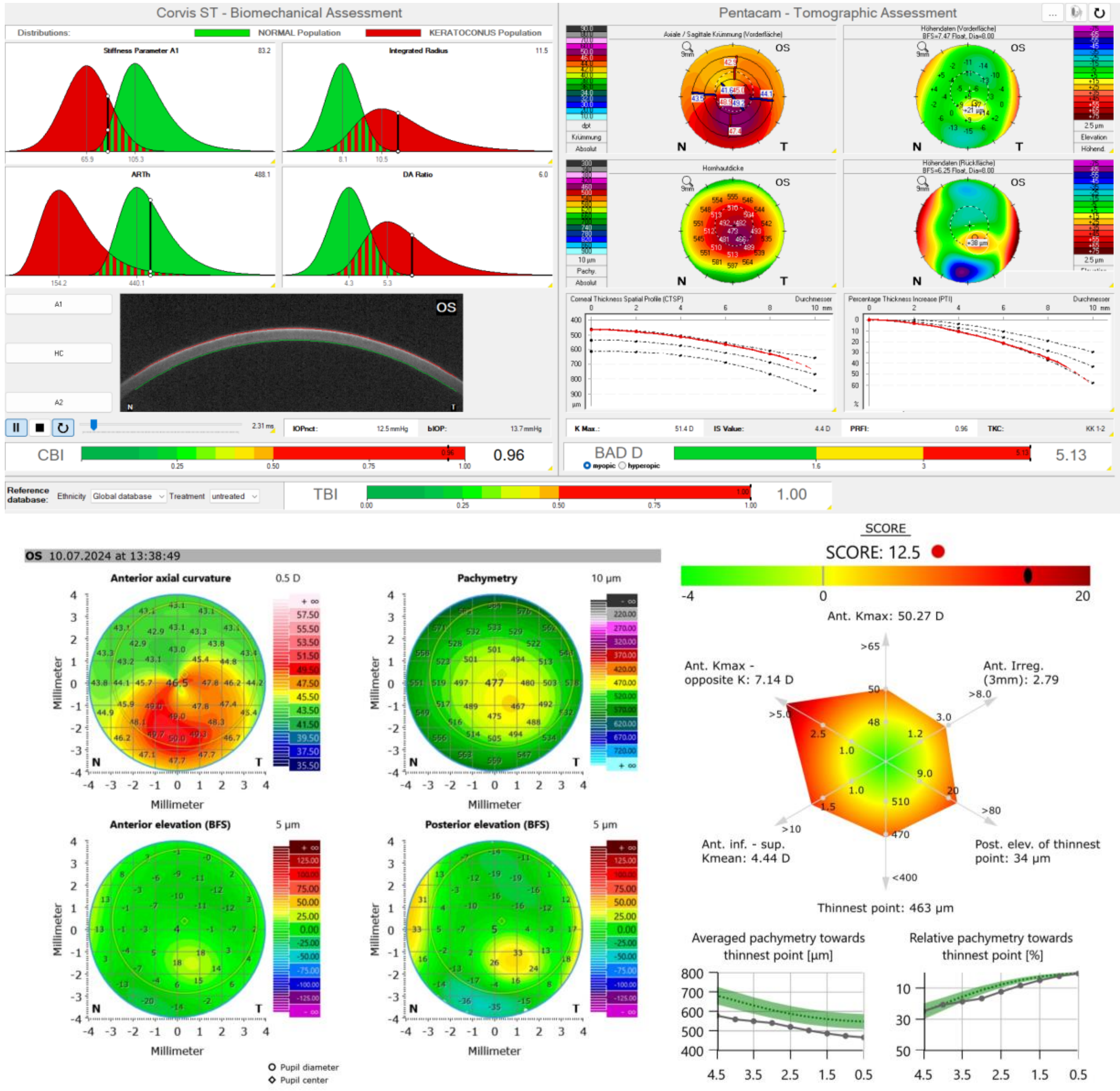

Corvis ST - Biomechanical Assessment
Pentacam - Tomographic Assessment
OS
CBI 0.96
BAD D 5.13
TBI 1.00
OS 10.07.2024 at 13:38:49
Anterior axial curvature
Pachymetry
Anterior elevation (BFS)
Posterior elevation (BFS)
SCORE
SCORE: 12.5
Ant. Kmax: 50.27 D
Ant. Kmax - opposite K: 7.14 D
Ant. Irreg. (3mm): 2.79
Ant. inf. - sup. Kmean: 4.44 D
Post. elev. of thinnest point: 34 µm
Thinnest point: 463 µm
Averaged pachymetry towards thinnest point [µm]
Relative pachymetry towards thinnest point [%]

CASE #71

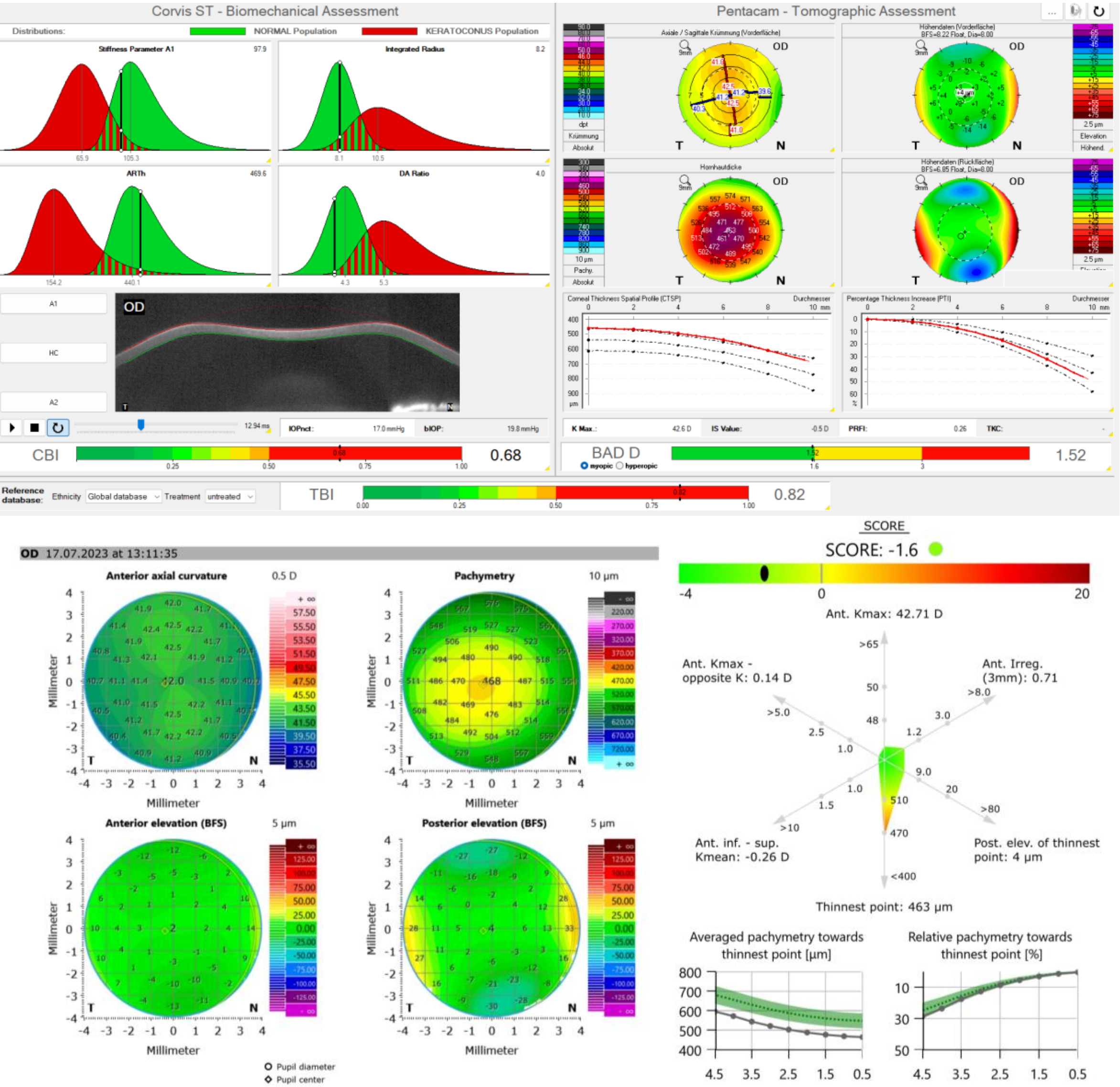

Corvis ST - Biomechanical Assessment
Pentacam - Tomographic Assessment
NORMAL Population
KERATOCONUS Population
Stiffness Parameter A1
Integrated Radius
ARTh
DA Ratio
OD
CBI
0.68
BAD D
1.52
TBI
0.82
Reference database:
OD 17.07.2023 at 13:11:35
Anterior axial curvature
Pachymetry
Anterior elevation (BFS)
Posterior elevation (BFS)
Millimeter
SCORE
SCORE: -1.6
Ant. Kmax: 42.71 D
Ant. Kmax - opposite K: 0.14 D
Ant. Irreg. (3mm): 0.71
Ant. inf. - sup. Kmean: -0.26 D
Post. elev. of thinnest point: 4 µm
Thinnest point: 463 µm
Averaged pachymetry towards thinnest point [µm]
Relative pachymetry towards thinnest point [%]
Pupil diameter
Pupil center

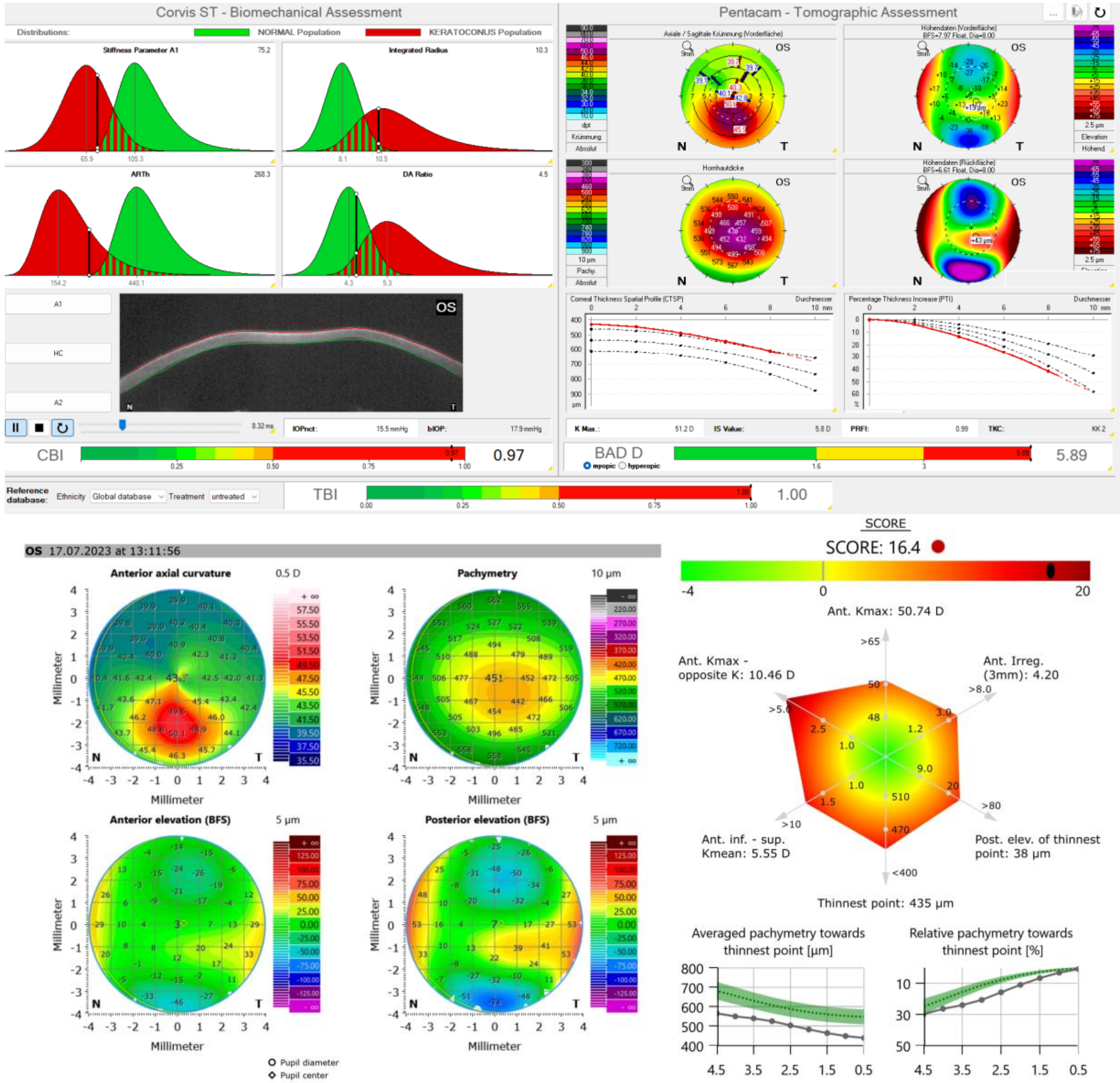

Corvis ST - Biomechanical Assessment
Pentacam - Tomographic Assessment
NORMAL Population
KERATOCONUS Population
Stiffness Parameter A1
Integrated Radius
ARTh
DA Ratio
OS
CBI
0.97
BAD D
5.89
TBI
1.00
Reference database:
OS 17.07.2023 at 13:11:56
Anterior axial curvature
Pachymetry
Anterior elevation (BFS)
Posterior elevation (BFS)
Millimeter
SCORE
SCORE: 16.4
Ant. Kmax: 50.74 D
Ant. Kmax - opposite K: 10.46 D
Ant. Irreg. (3mm): 4.20
Ant. inf. - sup. Kmean: 5.55 D
Post. elev. of thinnest point: 38 µm
Thinnest point: 435 µm
Averaged pachymetry towards thinnest point [µm]
Relative pachymetry towards thinnest point [%]
Pupil diameter
Pupil center

CASE #72

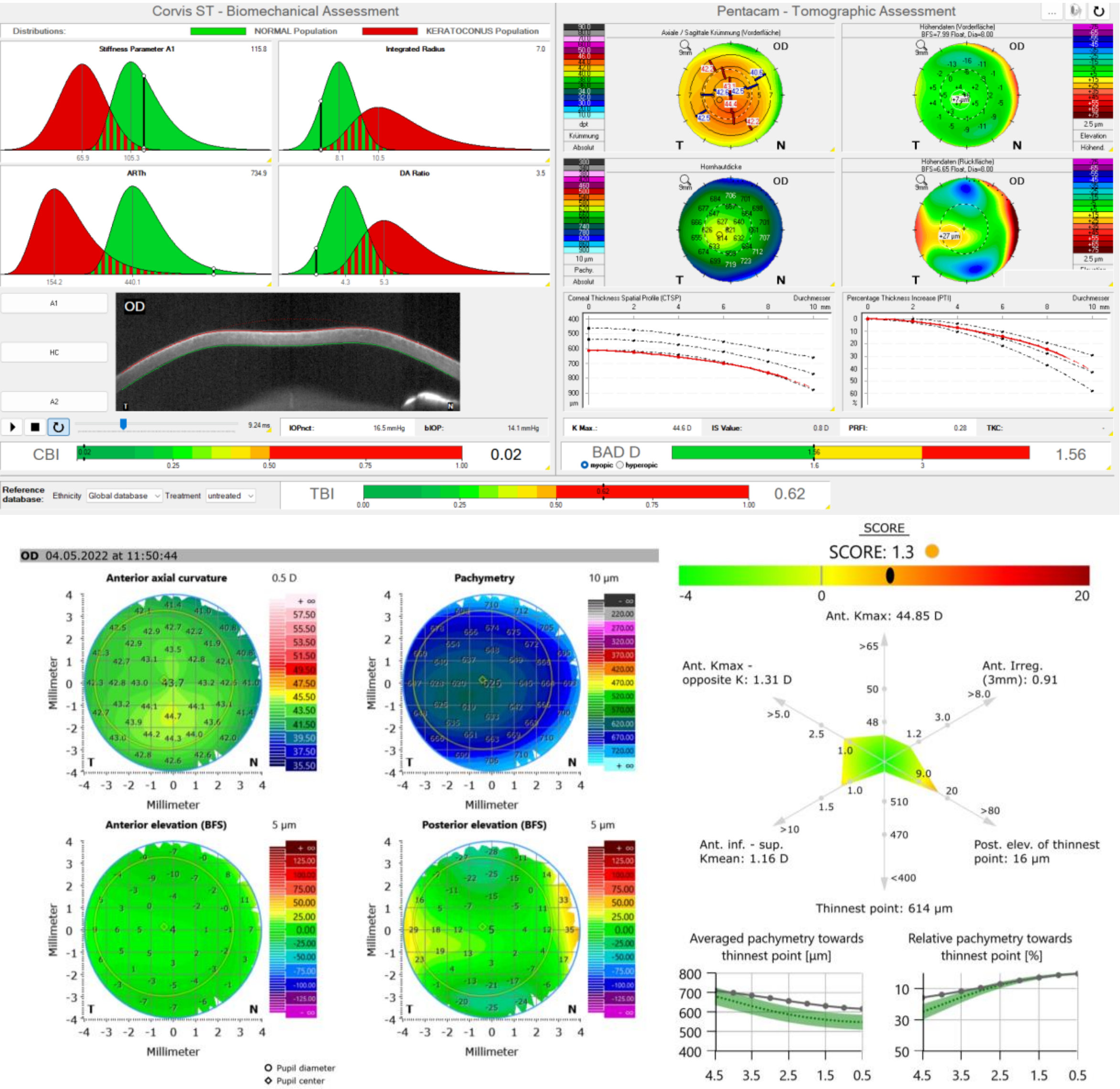

Corvis ST - Biomechanical Assessment
Pentacam - Tomographic Assessment
NORMAL Population
KERATOCONUS Population
Stiffness Parameter A1
Integrated Radius
ARTh
DA Ratio
OD
CBI 0.02
BAD D 1.56
TBI 0.62
OD 04.05.2022 at 11:50:44
Anterior axial curvature
Pachymetry
Anterior elevation (BFS)
Posterior elevation (BFS)
Millimeter
SCORE
SCORE: 1.3
Ant. Kmax: 44.85 D
Ant. Kmax - opposite K: 1.31 D
Ant. Irreg. (3mm): 0.91
Ant. inf. - sup. Kmean: 1.16 D
Post. elev. of thinnest point: 16 µm
Thinnest point: 614 µm
Averaged pachymetry towards thinnest point [µm]
Relative pachymetry towards thinnest point [%]
Pupil diameter
Pupil center

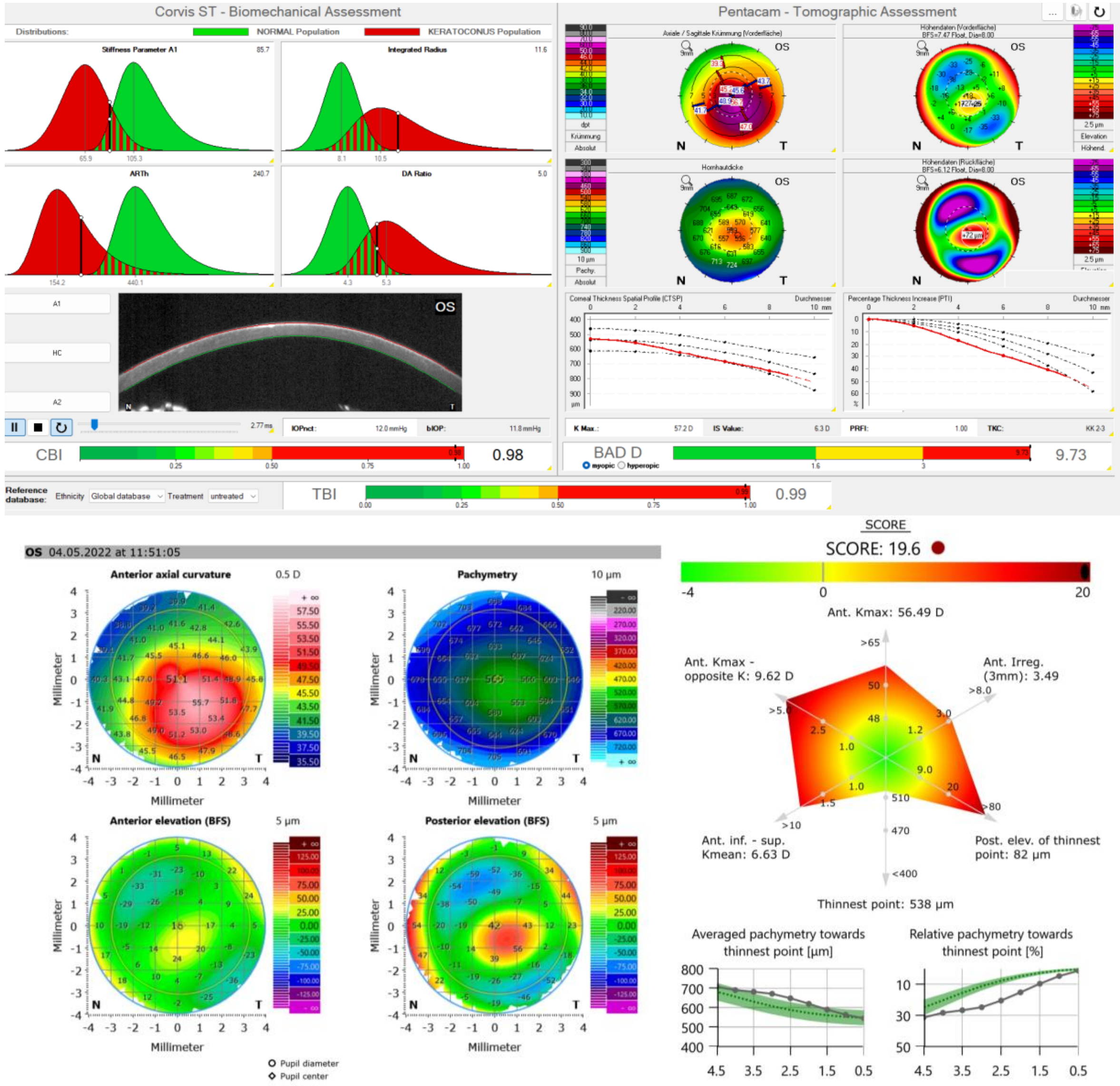

Corvis ST - Biomechanical Assessment
Pentacam - Tomographic Assessment
NORMAL Population
KERATOCONUS Population
Stiffness Parameter A1
Integrated Radius
ARTh
DA Ratio
OS
CBI 0.98
BAD D 9.73
TBI 0.99
OS 04.05.2022 at 11:51:05
Anterior axial curvature
Pachymetry
Anterior elevation (BFS)
Posterior elevation (BFS)
Millimeter
SCORE
SCORE: 19.6
Ant. Kmax: 56.49 D
Ant. Kmax - opposite K: 9.62 D
Ant. Irreg. (3mm): 3.49
Ant. inf. - sup. Kmean: 6.63 D
Post. elev. of thinnest point: 82 µm
Thinnest point: 538 µm
Averaged pachymetry towards thinnest point [µm]
Relative pachymetry towards thinnest point [%]
Pupil diameter
Pupil center

CASE #73

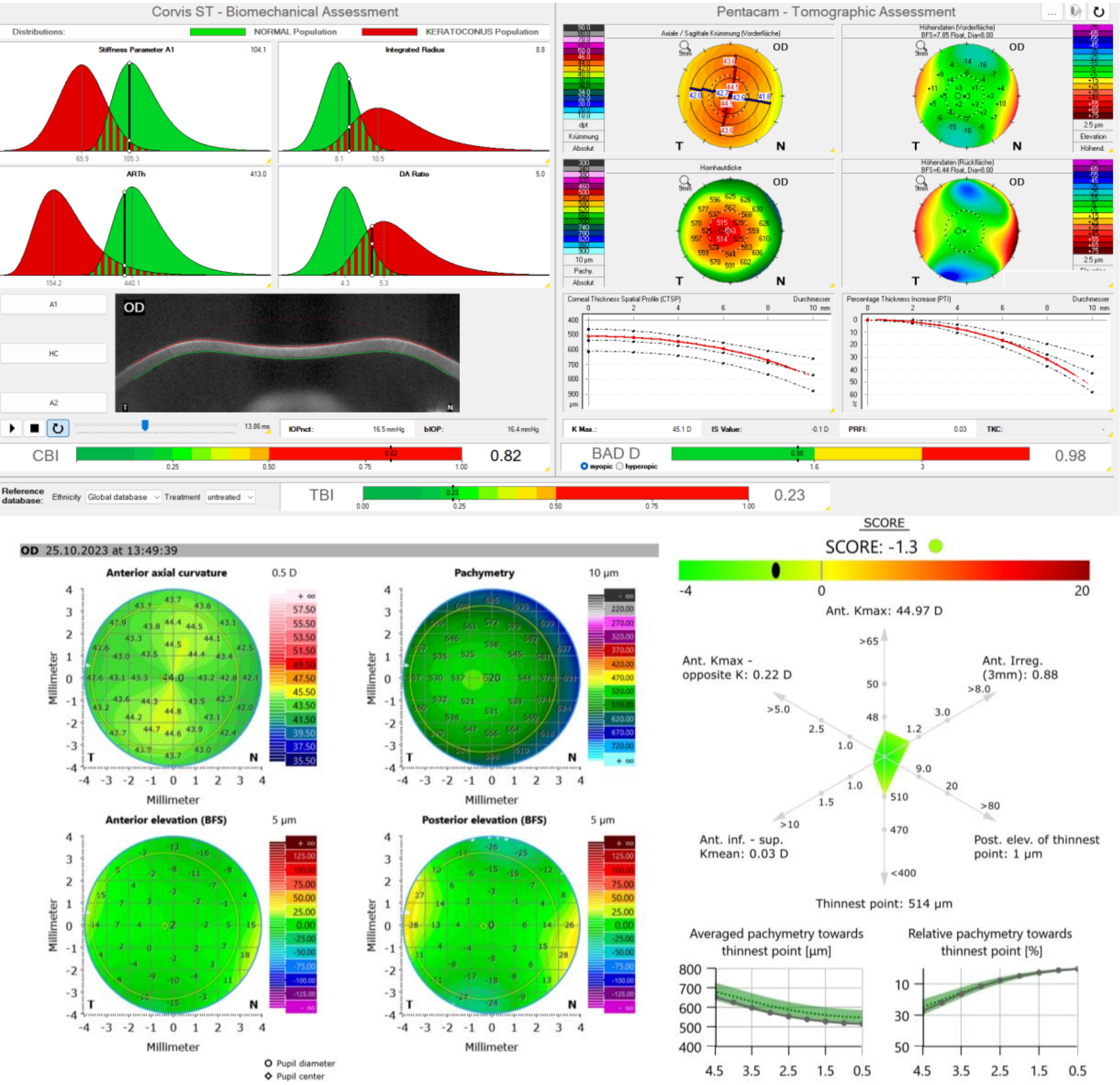

Corvis ST - Biomechanical Assessment
Pentacam - Tomographic Assessment
NORMAL Population
KERATOCONUS Population
Stiffness Parameter A1
Integrated Radius
ARTh
DA Ratio
OD
CBI
0.82
BAD D
0.98
TBI
0.23
OD 25.10.2023 at 13:49:39
Anterior axial curvature
Pachymetry
Anterior elevation (BFS)
Posterior elevation (BFS)
Millimeter
SCORE
SCORE: -1.3
Ant. Kmax: 44.97 D
Ant. Kmax - opposite K: 0.22 D
Ant. Irreg. (3mm): 0.88
Ant. inf. - sup. Kmean: 0.03 D
Post. elev. of thinnest point: 1 µm
Thinnest point: 514 µm
Averaged pachymetry towards thinnest point [µm]
Relative pachymetry towards thinnest point [%]
Pupil diameter
Pupil center

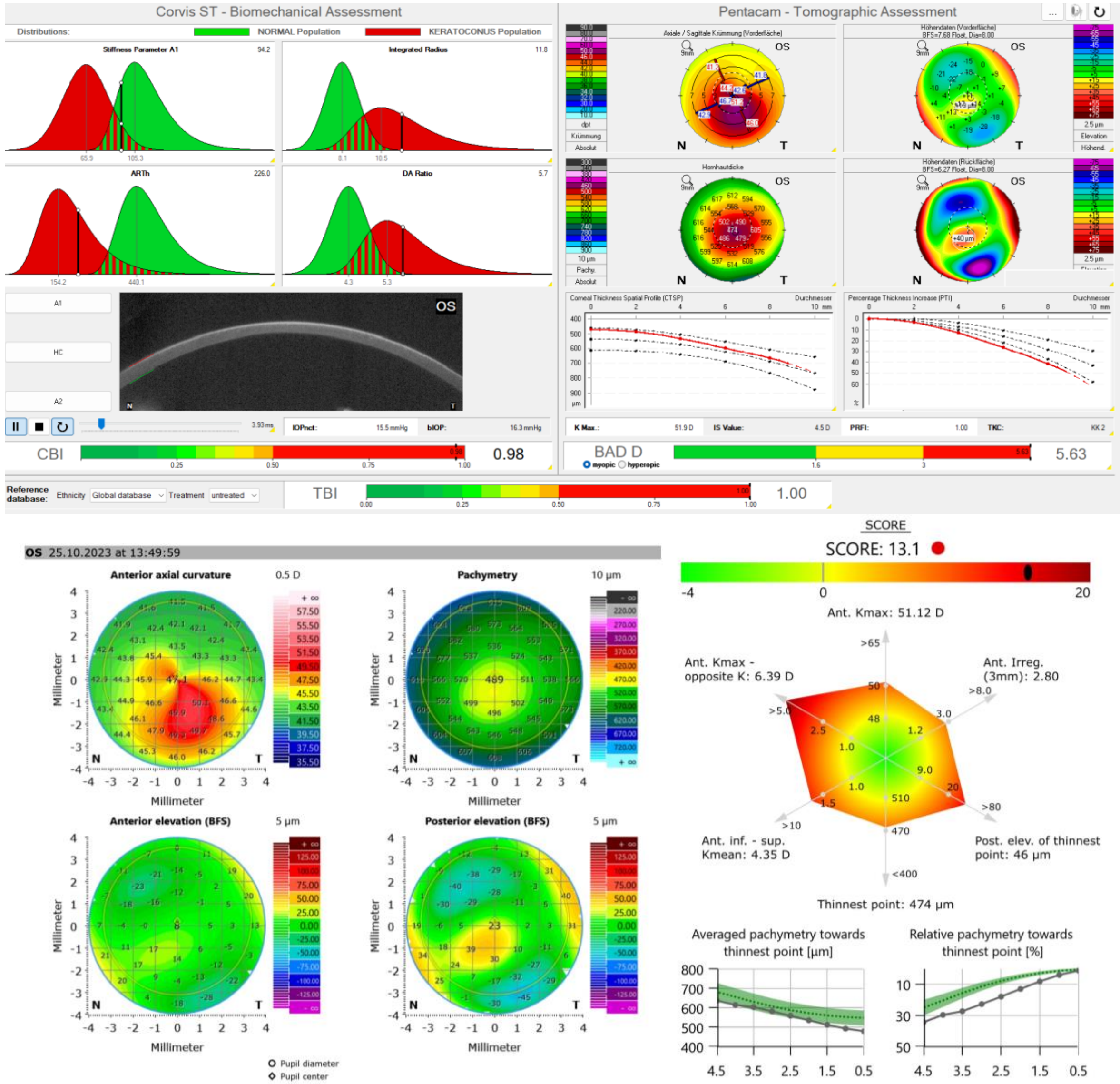

Corvis ST - Biomechanical Assessment
Pentacam - Tomographic Assessment
NORMAL Population
KERATOCONUS Population
Stiffness Parameter A1
Integrated Radius
ARTh
DA Ratio
OS
CBI
0.98
BAD D
5.63
TBI
1.00
OS 25.10.2023 at 13:49:59
Anterior axial curvature
Pachymetry
Anterior elevation (BFS)
Posterior elevation (BFS)
Millimeter
SCORE
SCORE: 13.1
Ant. Kmax: 51.12 D
Ant. Kmax - opposite K: 6.39 D
Ant. Irreg. (3mm): 2.80
Ant. inf. - sup. Kmean: 4.35 D
Post. elev. of thinnest point: 46 µm
Thinnest point: 474 µm
Averaged pachymetry towards thinnest point [µm]
Relative pachymetry towards thinnest point [%]
Pupil diameter
Pupil center

CASE #74

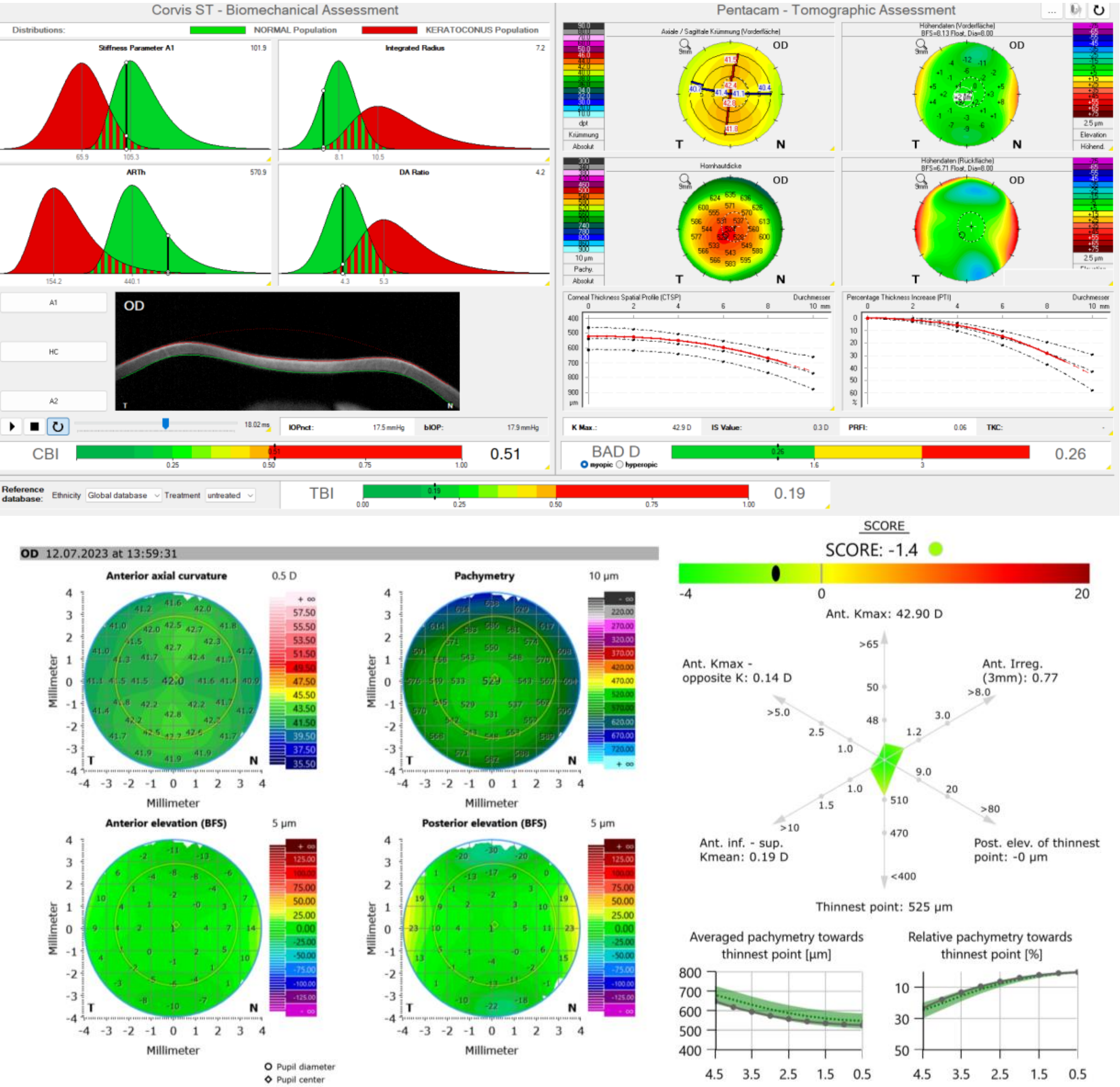

Corvis ST - Biomechanical Assessment
Pentacam - Tomographic Assessment
NORMAL Population
KERATOCONUS Population
OD
CBI 0.51
BAD D 0.26
TBI 0.19
SCORE
OD 12.07.2023 at 13:59:31
Anterior axial curvature
Pachymetry
Anterior elevation (BFS)
Posterior elevation (BFS)
Millimeter
SCORE: -1.4
Ant. Kmax: 42.90 D
Ant. Kmax - opposite K: 0.14 D
Ant. Irreg. (3mm): 0.77
Ant. inf. - sup. Kmean: 0.19 D
Post. elev. of thinnest point: -0 µm
Thinnest point: 525 µm
Averaged pachymetry towards thinnest point [µm]
Relative pachymetry towards thinnest point [%]
Pupil diameter
Pupil center

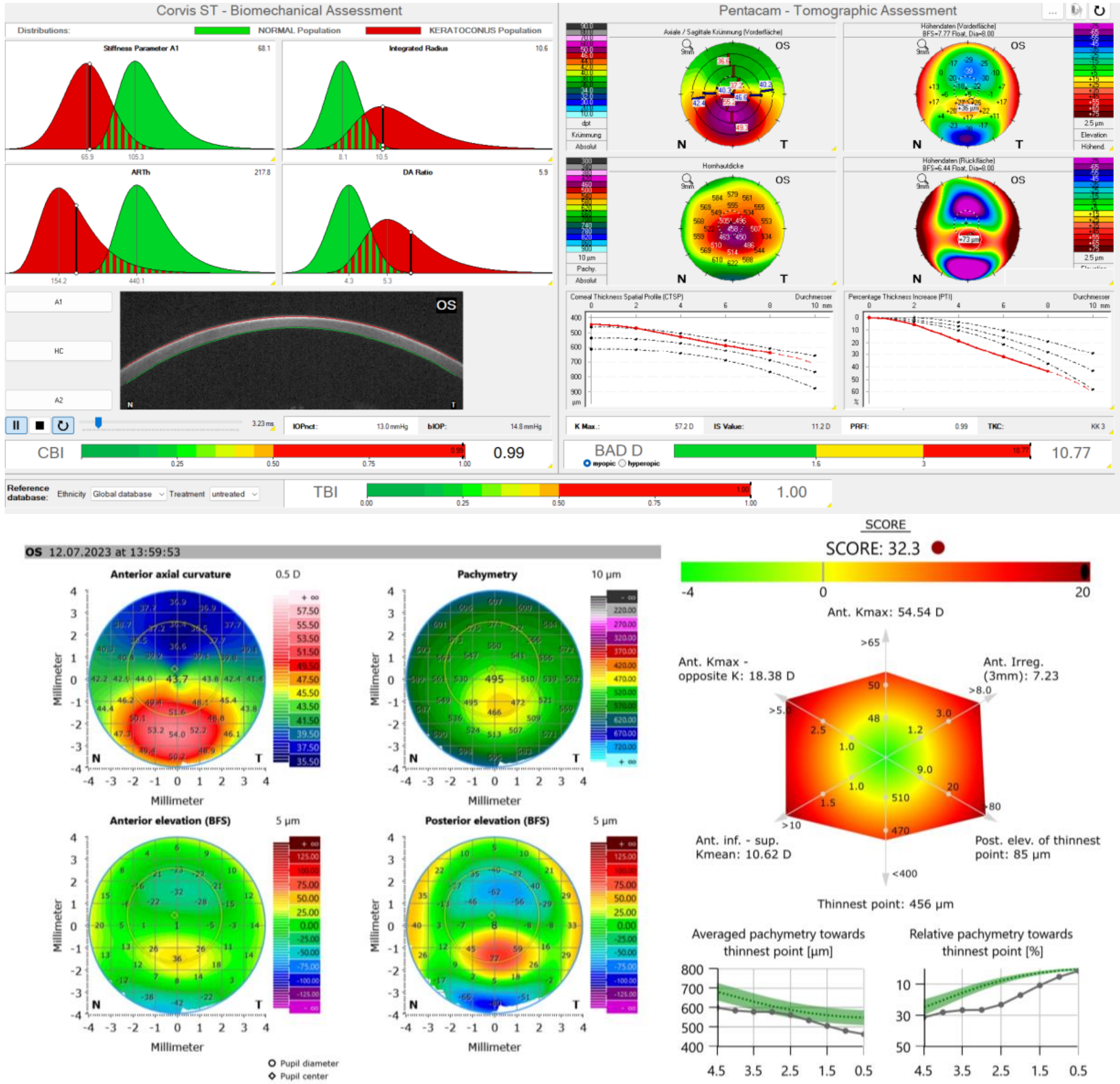

Corvis ST - Biomechanical Assessment
Pentacam - Tomographic Assessment
NORMAL Population
KERATOCONUS Population
OS
CBI 0.99
BAD D 10.77
TBI 1.00
SCORE
OS 12.07.2023 at 13:59:53
Anterior axial curvature
Pachymetry
Anterior elevation (BFS)
Posterior elevation (BFS)
Millimeter
SCORE: 32.3
Ant. Kmax: 54.54 D
Ant. Kmax - opposite K: 18.38 D
Ant. Irreg. (3mm): 7.23
Ant. inf. - sup. Kmean: 10.62 D
Post. elev. of thinnest point: 85 µm
Thinnest point: 456 µm
Averaged pachymetry towards thinnest point [µm]
Relative pachymetry towards thinnest point [%]
Pupil diameter
Pupil center

CASE #75

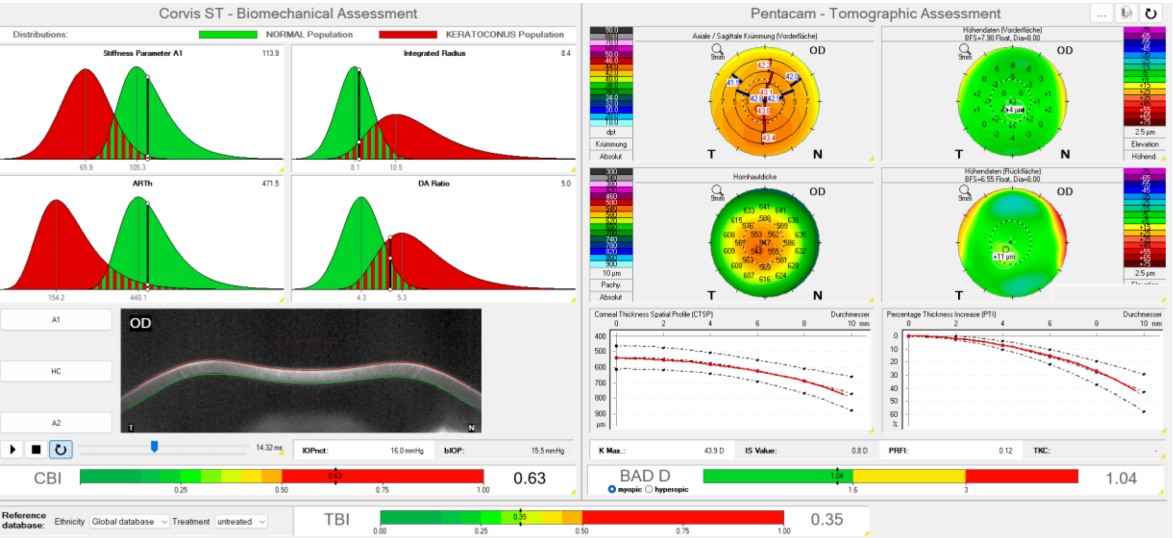
Corvis ST - Biomechanical Assessment
Pentacam - Tomographic Assessment
NORMAL Population
KERATOCONUS Population
Stiffness Parameter A1
Integrated Radius
ARTh
DA Ratio
OD
CBI 0.63
BAD D 1.04
TBI 0.35
Reference database: Ethnicity Global database Treatment untreated

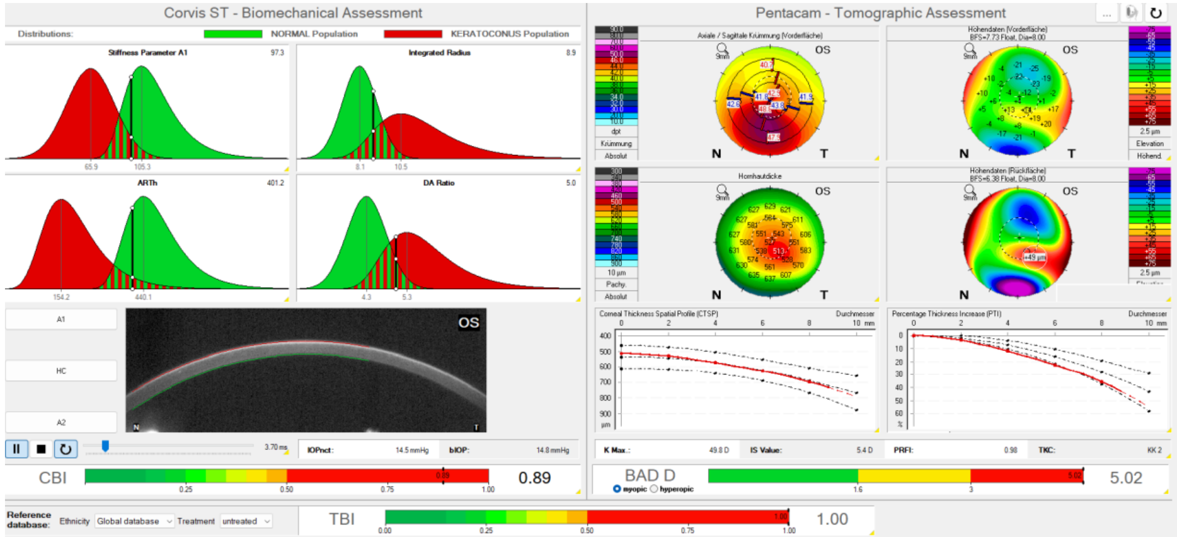
Corvis ST - Biomechanical Assessment
Pentacam - Tomographic Assessment
NORMAL Population
KERATOCONUS Population
Stiffness Parameter A1
Integrated Radius
ARTh
DA Ratio
OS
CBI 0.89
BAD D 5.02
TBI 1.00
Reference database: Ethnicity Global database Treatment untreated

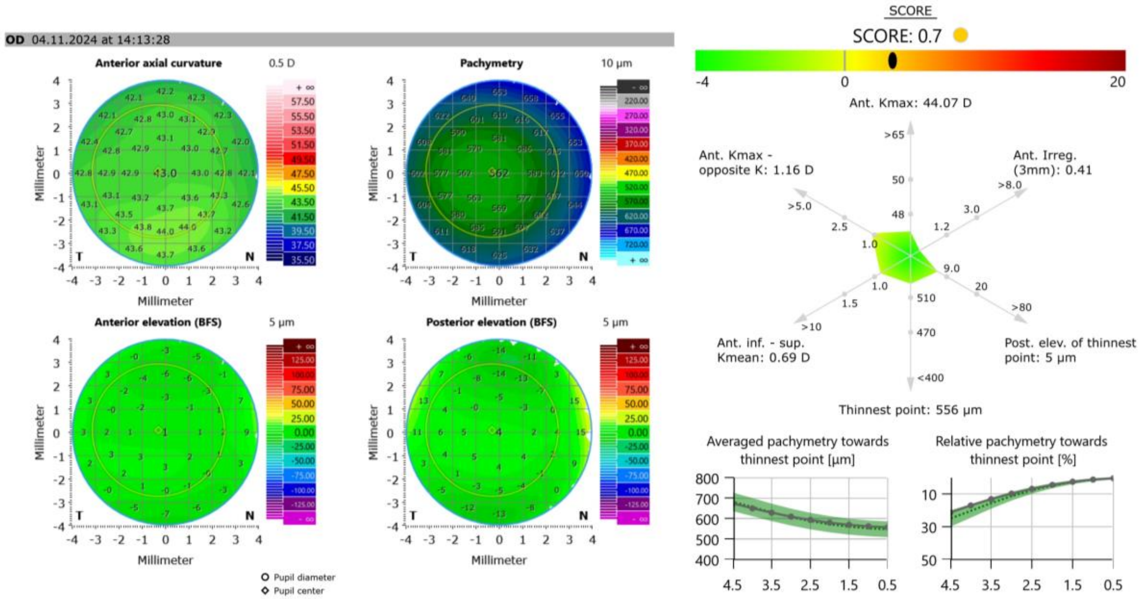
OD 04.11.2024 at 14:13:28
Anterior axial curvature
Pachymetry
Anterior elevation (BFS)
Posterior elevation (BFS)
Millimeter
Pupil diameter
Pupil center
SCORE
SCORE: 0.7
Ant. Kmax: 44.07 D
Ant. Kmax - opposite K: 1.16 D
Ant. Irreg. (3mm): 0.41
Ant. inf. - sup. Kmean: 0.69 D
Post. elev. of thinnest point: 5 µm
Thinnest point: 556 µm
Averaged pachymetry towards thinnest point [µm]
Relative pachymetry towards thinnest point [%]

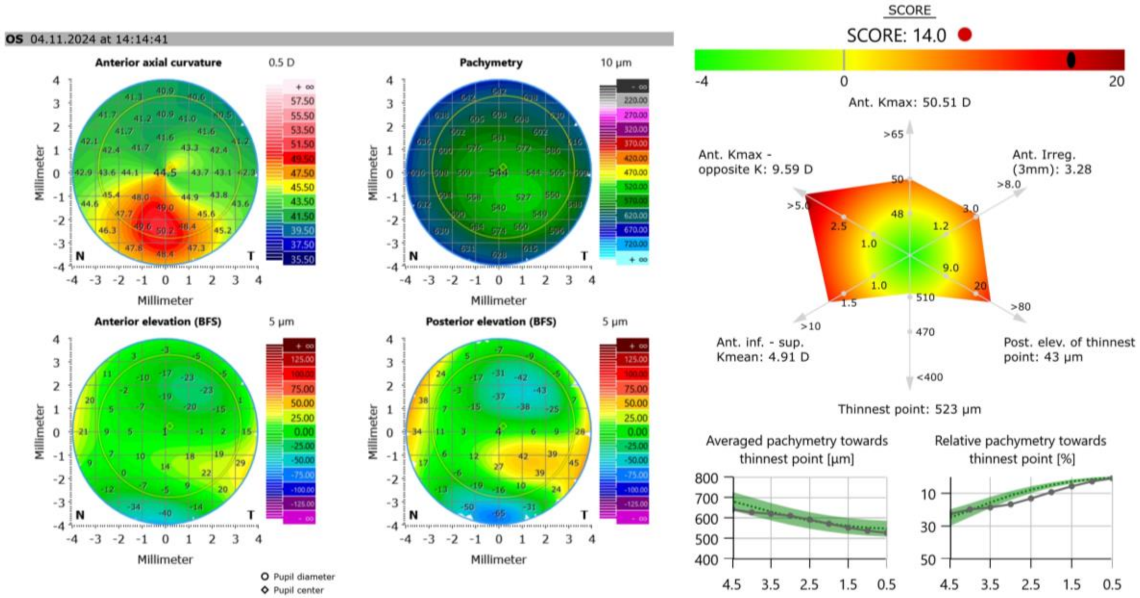
OS 04.11.2024 at 14:14:41
Anterior axial curvature
Pachymetry
Anterior elevation (BFS)
Posterior elevation (BFS)
Millimeter
Pupil diameter
Pupil center
SCORE
SCORE: 14.0
Ant. Kmax: 50.51 D
Ant. Kmax - opposite K: 9.59 D
Ant. Irreg. (3mm): 3.28
Ant. inf. - sup. Kmean: 4.91 D
Post. elev. of thinnest point: 43 µm
Thinnest point: 523 µm
Averaged pachymetry towards thinnest point [µm]
Relative pachymetry towards thinnest point [%]

CASE #76

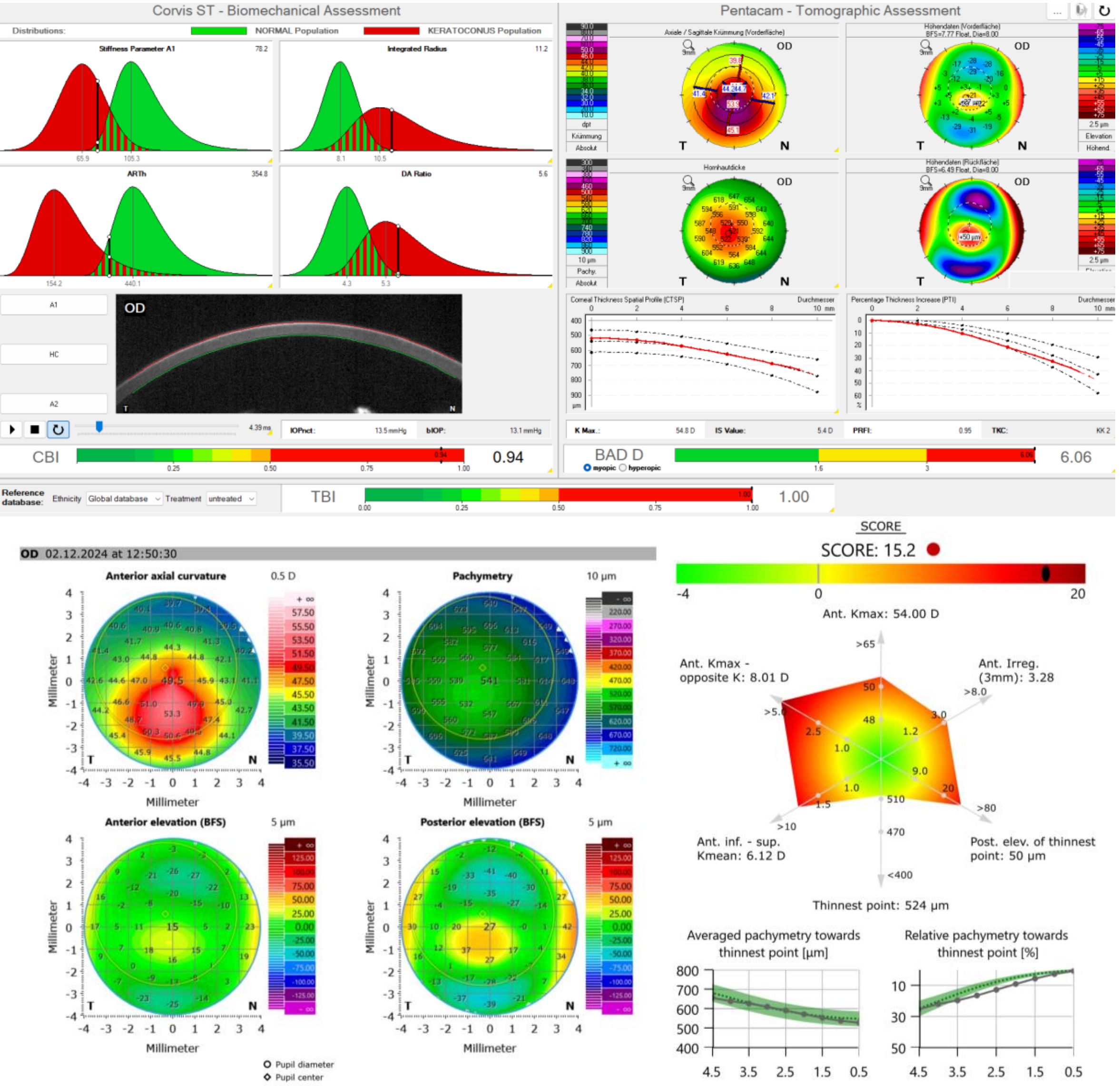

Corvis ST - Biomechanical Assessment
Pentacam - Tomographic Assessment
Distributions: NORMAL Population KERATOCONUS Population
Stiffness Parameter A1
Integrated Radius
ARTh
DA Ratio
OD
CBI 0.94
BAD D 6.06
TBI 1.00
Reference database: Ethnicity Global database Treatment untreated
OD 02.12.2024 at 12:50:30
Anterior axial curvature
Pachymetry
Anterior elevation (BFS)
Posterior elevation (BFS)
Millimeter
Pupil diameter
Pupil center
SCORE
SCORE: 15.2
Ant. Kmax: 54.00 D
Ant. Kmax - opposite K: 8.01 D
Ant. Irreg. (3mm): 3.28
Ant. inf. - sup. Kmean: 6.12 D
Post. elev. of thinnest point: 50 µm
Thinnest point: 524 µm
Averaged pachymetry towards thinnest point [µm]
Relative pachymetry towards thinnest point [%]

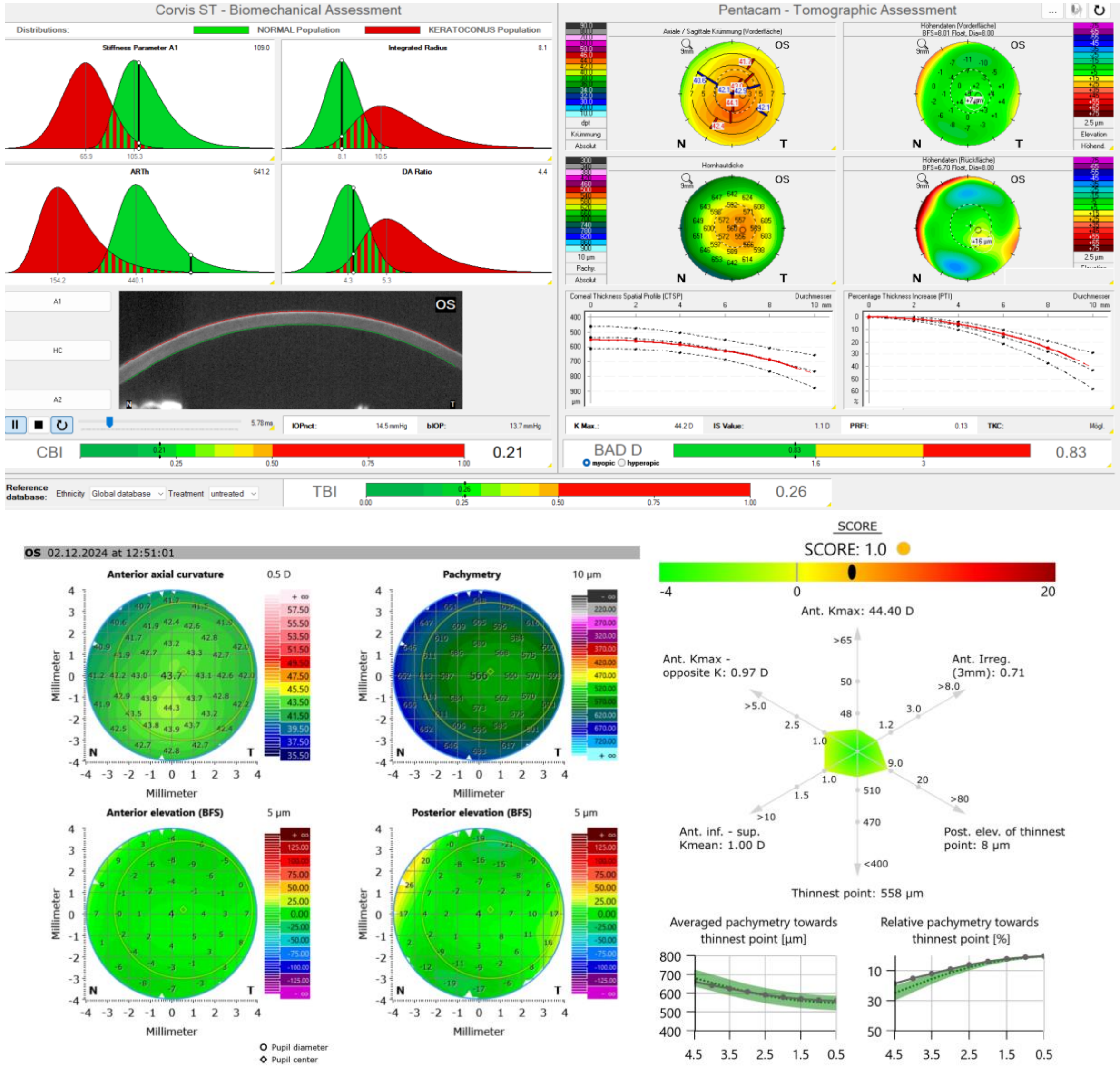

Corvis ST - Biomechanical Assessment
Pentacam - Tomographic Assessment
Distributions: NORMAL Population KERATOCONUS Population
Stiffness Parameter A1
Integrated Radius
ARTh
DA Ratio
OS
CBI 0.21
BAD D 0.83
TBI 0.26
Reference database: Ethnicity Global database Treatment untreated
OS 02.12.2024 at 12:51:01
Anterior axial curvature
Pachymetry
Anterior elevation (BFS)
Posterior elevation (BFS)
Millimeter
Pupil diameter
Pupil center
SCORE
SCORE: 1.0
Ant. Kmax: 44.40 D
Ant. Kmax - opposite K: 0.97 D
Ant. Irreg. (3mm): 0.71
Ant. inf. - sup. Kmean: 1.00 D
Post. elev. of thinnest point: 8 µm
Thinnest point: 558 µm
Averaged pachymetry towards thinnest point [µm]
Relative pachymetry towards thinnest point [%]

CASE #77

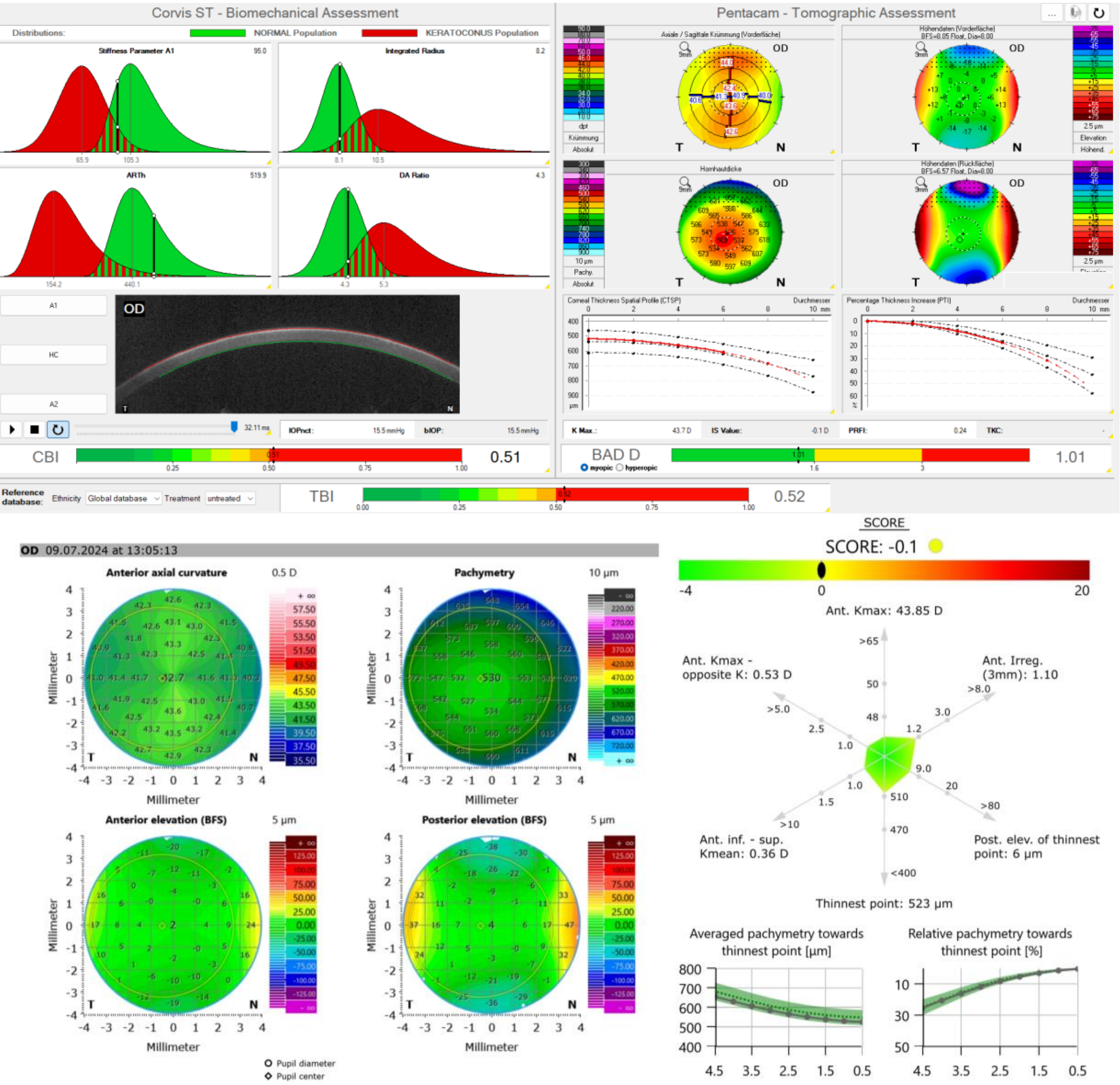

Corvis ST - Biomechanical Assessment
Pentacam - Tomographic Assessment
NORMAL Population
KERATOCONUS Population
Stiffness Parameter A1
Integrated Radius
ARTh
DA Ratio
OD
CBI
0.51
BAD D
1.01
TBI
0.52
OD 09.07.2024 at 13:05:13
Anterior axial curvature
Pachymetry
Anterior elevation (BFS)
Posterior elevation (BFS)
Millimeter
Pupil diameter
Pupil center
SCORE
SCORE: -0.1
Ant. Kmax: 43.85 D
Ant. Kmax - opposite K: 0.53 D
Ant. Irreg. (3mm): 1.10
Ant. inf. - sup. Kmean: 0.36 D
Post. elev. of thinnest point: 6 µm
Thinnest point: 523 µm
Averaged pachymetry towards thinnest point [µm]
Relative pachymetry towards thinnest point [%]

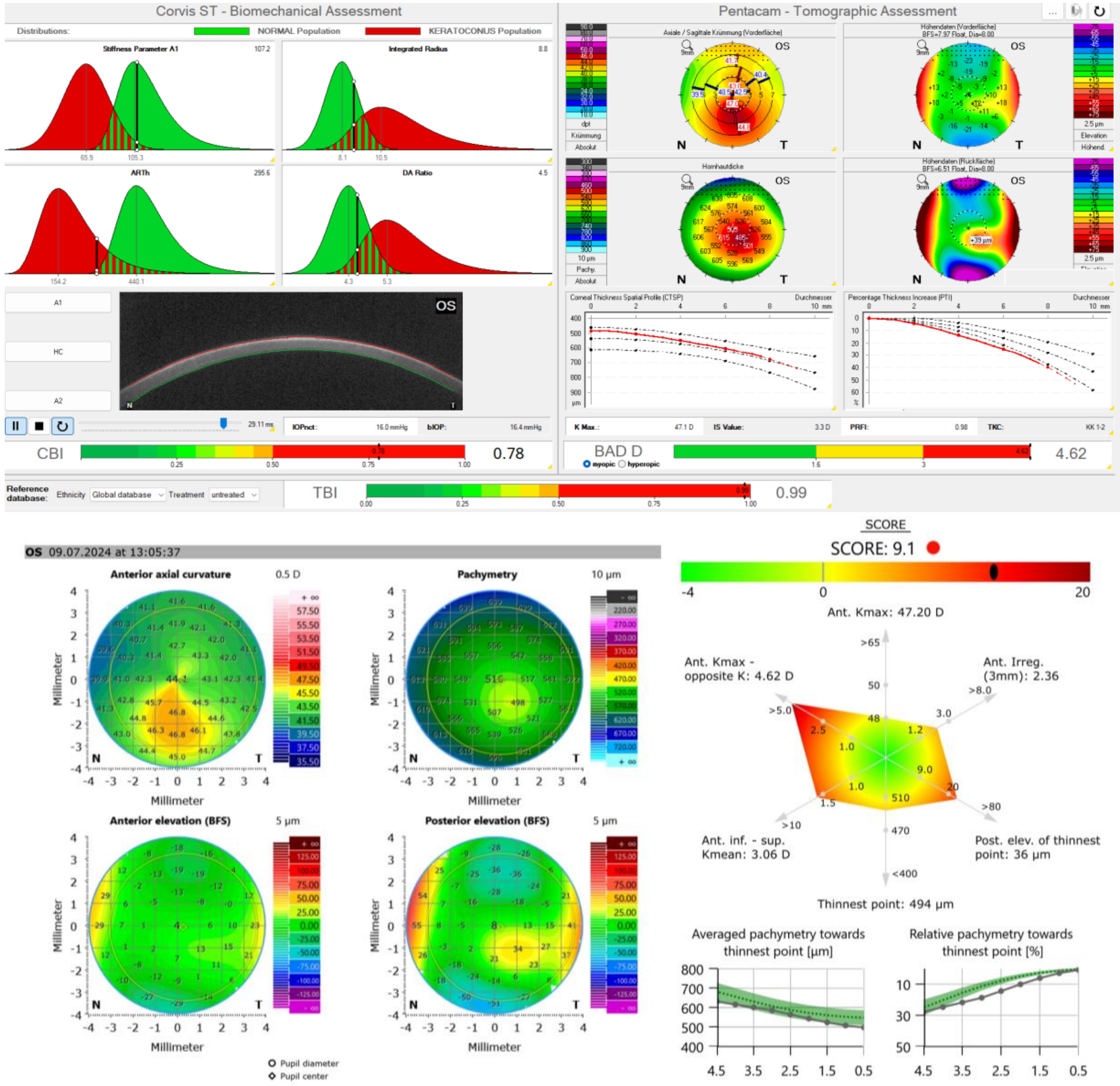

Corvis ST - Biomechanical Assessment
Pentacam - Tomographic Assessment
NORMAL Population
KERATOCONUS Population
Stiffness Parameter A1
Integrated Radius
ARTh
DA Ratio
OS
CBI
0.78
BAD D
4.62
TBI
0.99
OS 09.07.2024 at 13:05:37
Anterior axial curvature
Pachymetry
Anterior elevation (BFS)
Posterior elevation (BFS)
Millimeter
Pupil diameter
Pupil center
SCORE
SCORE: 9.1
Ant. Kmax: 47.20 D
Ant. Kmax - opposite K: 4.62 D
Ant. Irreg. (3mm): 2.36
Ant. inf. - sup. Kmean: 3.06 D
Post. elev. of thinnest point: 36 µm
Thinnest point: 494 µm
Averaged pachymetry towards thinnest point [µm]
Relative pachymetry towards thinnest point [%]

CASE #78

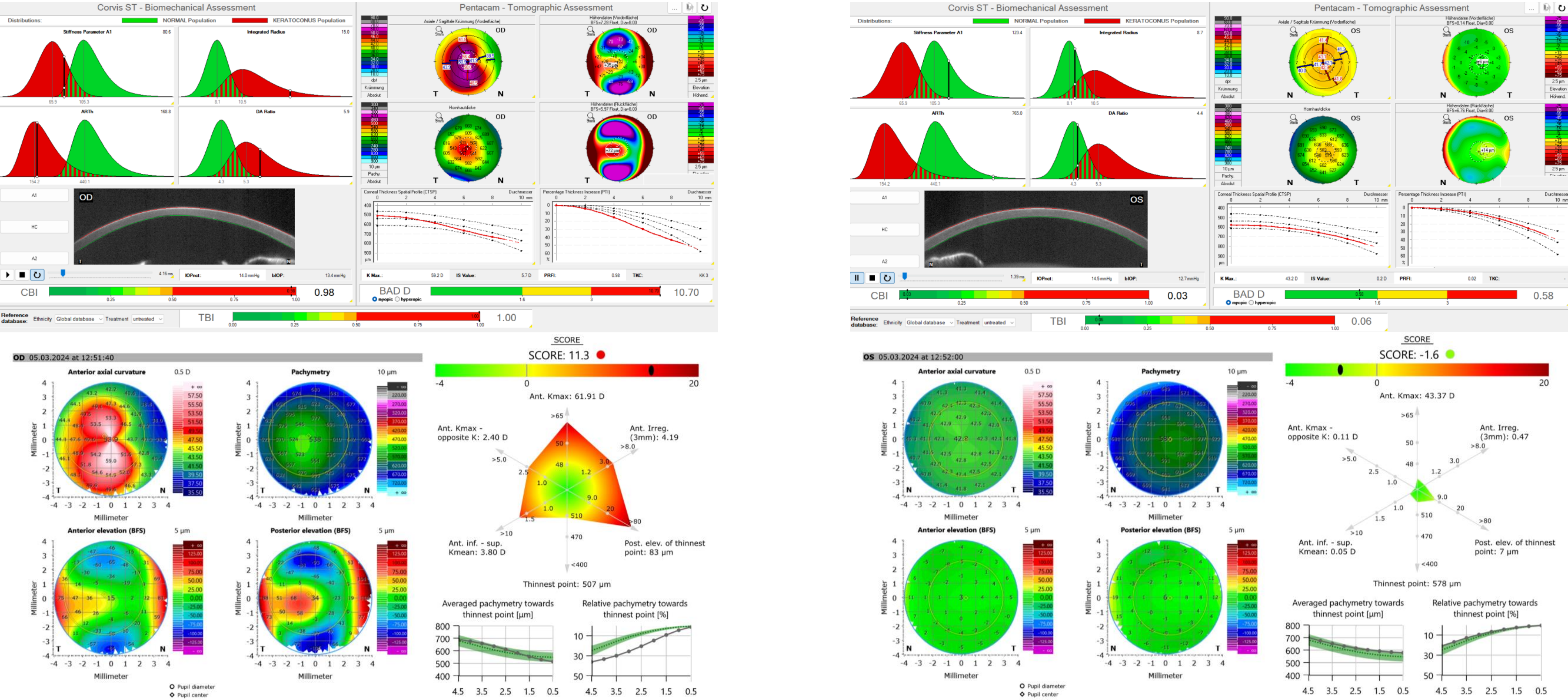

Corvis ST - Biomechanical Assessment
Pentacam - Tomographic Assessment
OD
CBI 0.98
BAD D 10.70
TBI 1.00
OD 05.03.2024 at 12:51:40
SCORE: 11.3
Ant. Kmax: 61.91 D
Ant. Kmax - opposite K: 2.40 D
Ant. Irreg. (3mm): 4.19
Ant. inf. - sup. Kmean: 3.80 D
Post. elev. of thinnest point: 83 µm
Thinnest point: 507 µm
Averaged pachymetry towards thinnest point [µm]
Relative pachymetry towards thinnest point [%]
Anterior axial curvature
Pachymetry
Anterior elevation (BFS)
Posterior elevation (BFS)
Millimeter
OS
CBI 0.03
BAD D 0.58
TBI 0.06
OS 05.03.2024 at 12:52:00
SCORE: -1.6
Ant. Kmax: 43.37 D
Ant. Kmax - opposite K: 0.11 D
Ant. Irreg. (3mm): 0.47
Ant. inf. - sup. Kmean: 0.05 D
Post. elev. of thinnest point: 7 µm
Thinnest point: 578 µm

CASE #79

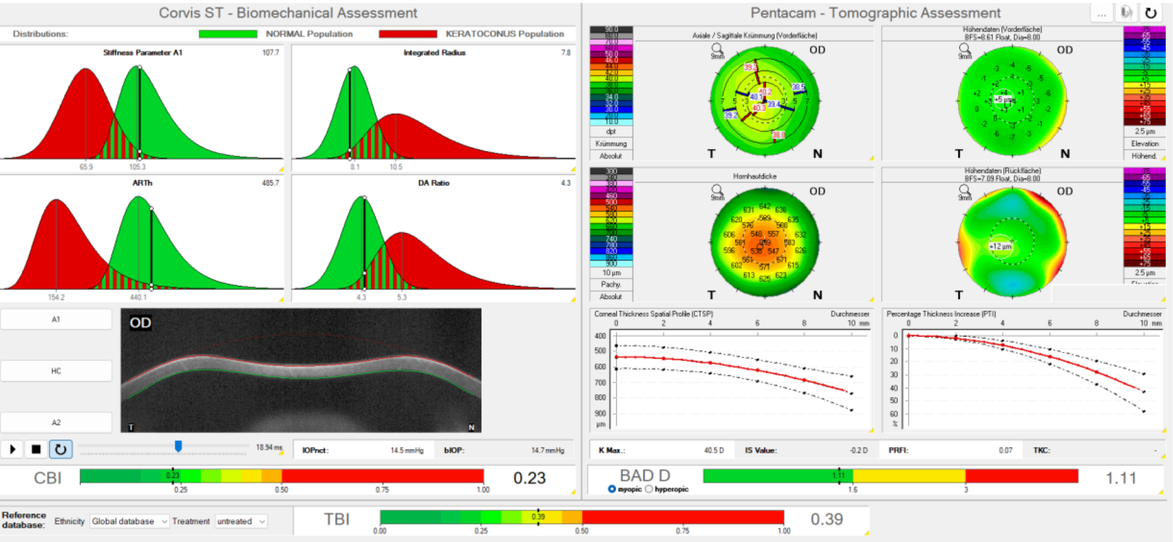
Corvis ST - Biomechanical Assessment
Pentacam - Tomographic Assessment
NORMAL Population
KERATOCONUS Population
OD
CBI
0.23
BAD D
1.11
TBI
0.39

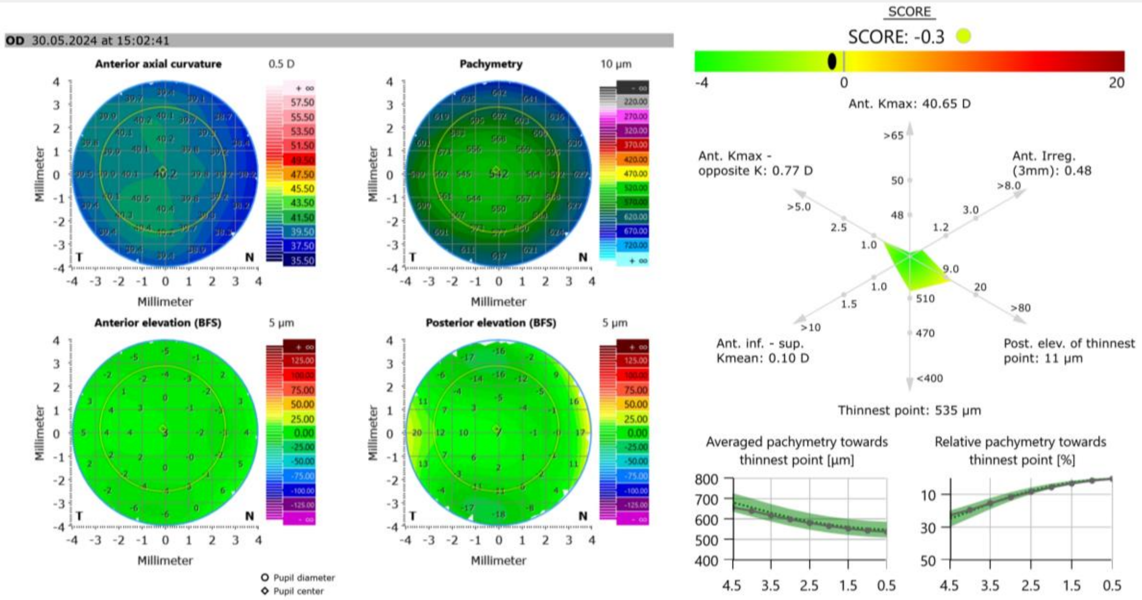
OD 30.05.2024 at 15:02:41
Anterior axial curvature
Pachymetry
Anterior elevation (BFS)
Posterior elevation (BFS)
Millimeter
SCORE
SCORE: -0.3
Ant. Kmax: 40.65 D
Ant. Kmax - opposite K: 0.77 D
Ant. Irreg. (3mm): 0.48
Ant. inf. - sup. Kmean: 0.10 D
Post. elev. of thinnest point: 11 µm
Thinnest point: 535 µm
Averaged pachymetry towards thinnest point [µm]
Relative pachymetry towards thinnest point [%]
Pupil diameter
Pupil center

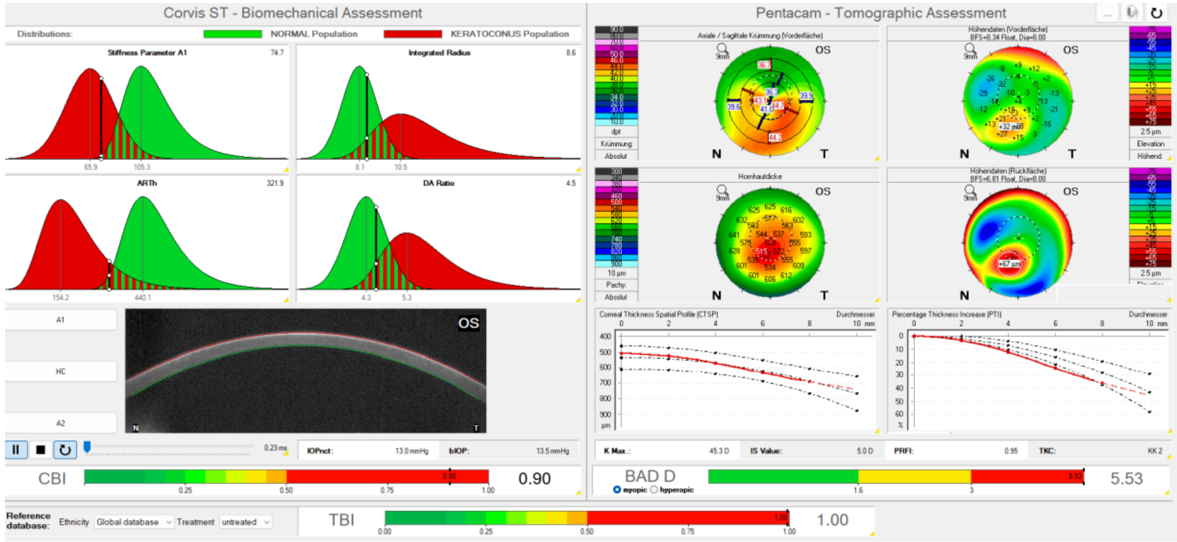
Corvis ST - Biomechanical Assessment
Pentacam - Tomographic Assessment
NORMAL Population
KERATOCONUS Population
OS
CBI
0.90
BAD D
5.53
TBI
1.00

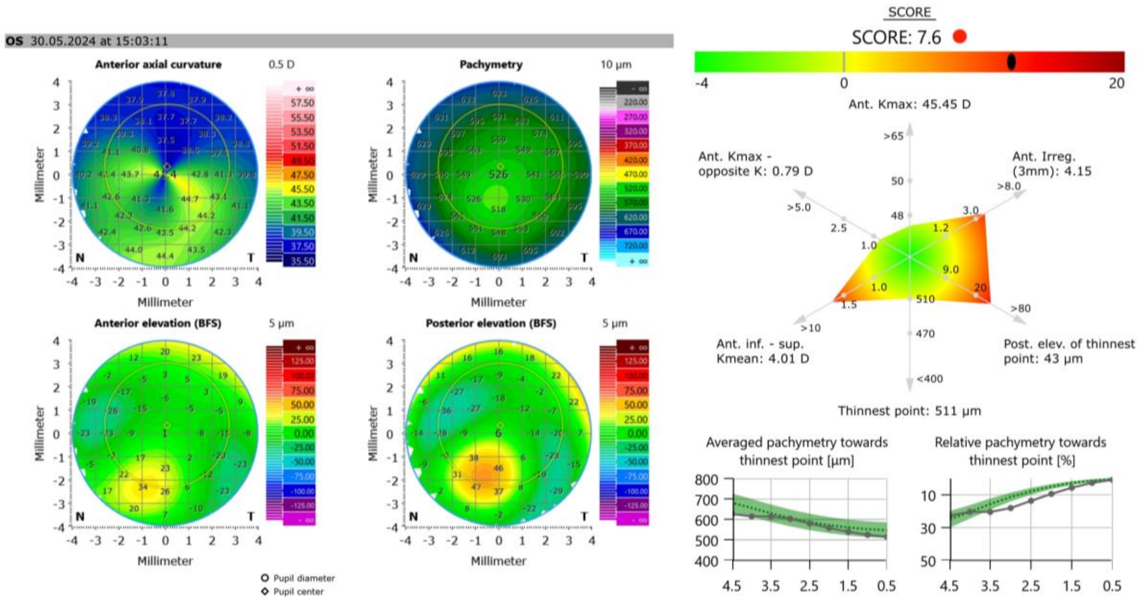
OS 30.05.2024 at 15:03:11
Anterior axial curvature
Pachymetry
Anterior elevation (BFS)
Posterior elevation (BFS)
Millimeter
SCORE
SCORE: 7.6
Ant. Kmax: 45.45 D
Ant. Kmax - opposite K: 0.79 D
Ant. Irreg. (3mm): 4.15
Ant. inf. - sup. Kmean: 4.01 D
Post. elev. of thinnest point: 43 µm
Thinnest point: 511 µm
Averaged pachymetry towards thinnest point [µm]
Relative pachymetry towards thinnest point [%]
Pupil diameter
Pupil center

CASE #80

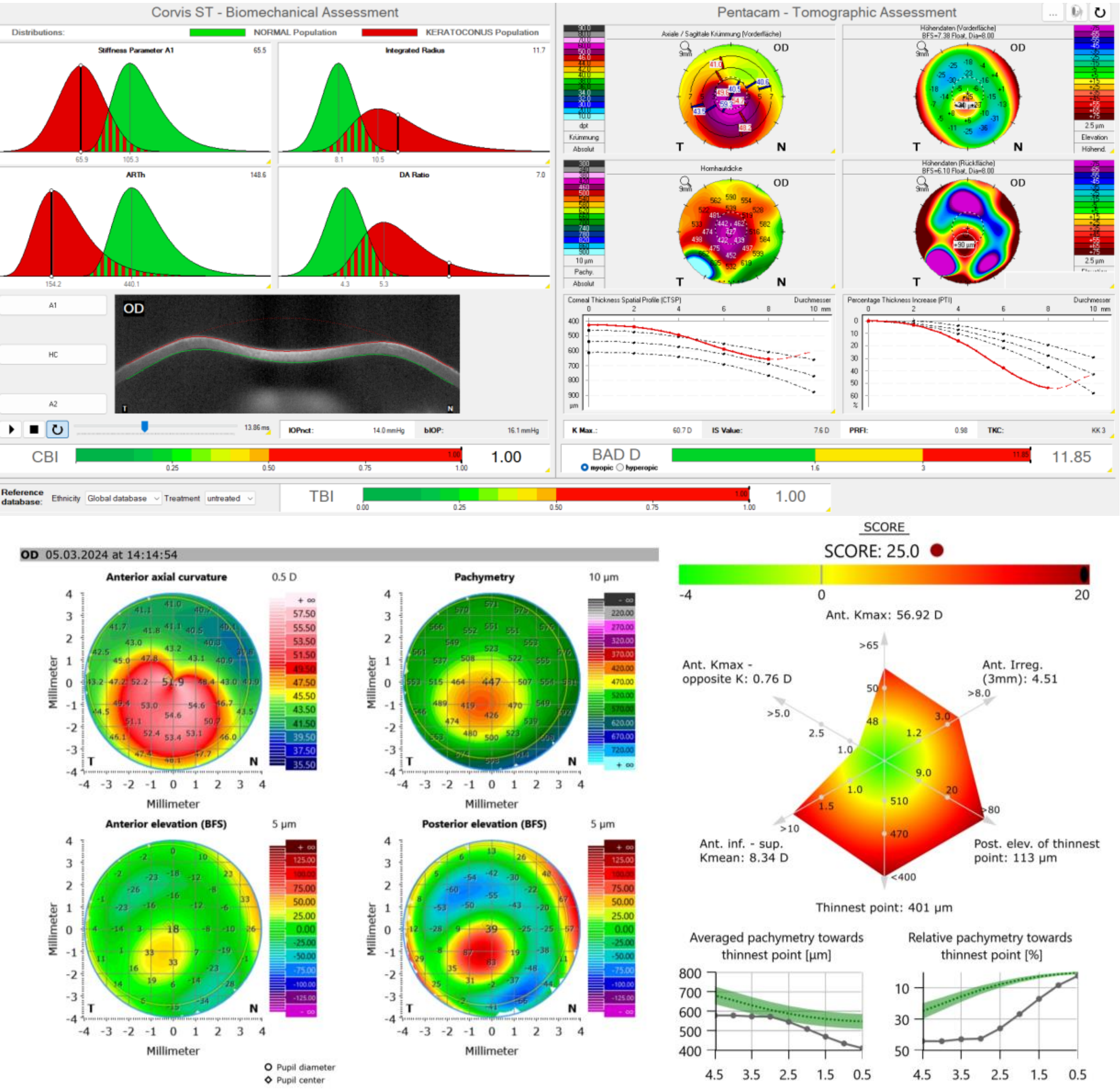

Corvis ST - Biomechanical Assessment
Pentacam - Tomographic Assessment
NORMAL Population
KERATOCONUS Population
Stiffness Parameter A1
Integrated Radius
ARTh
DA Ratio
OD
CBI
1.00
BAD D
11.85
TBI
1.00
OD 05.03.2024 at 14:14:54
Anterior axial curvature
Pachymetry
Anterior elevation (BFS)
Posterior elevation (BFS)
Millimeter
SCORE
SCORE: 25.0
Ant. Kmax: 56.92 D
Ant. Kmax - opposite K: 0.76 D
Ant. Irreg. (3mm): 4.51
Ant. inf. - sup. Kmean: 8.34 D
Post. elev. of thinnest point: 113 µm
Thinnest point: 401 µm
Averaged pachymetry towards thinnest point [µm]
Relative pachymetry towards thinnest point [%]
Pupil diameter
Pupil center

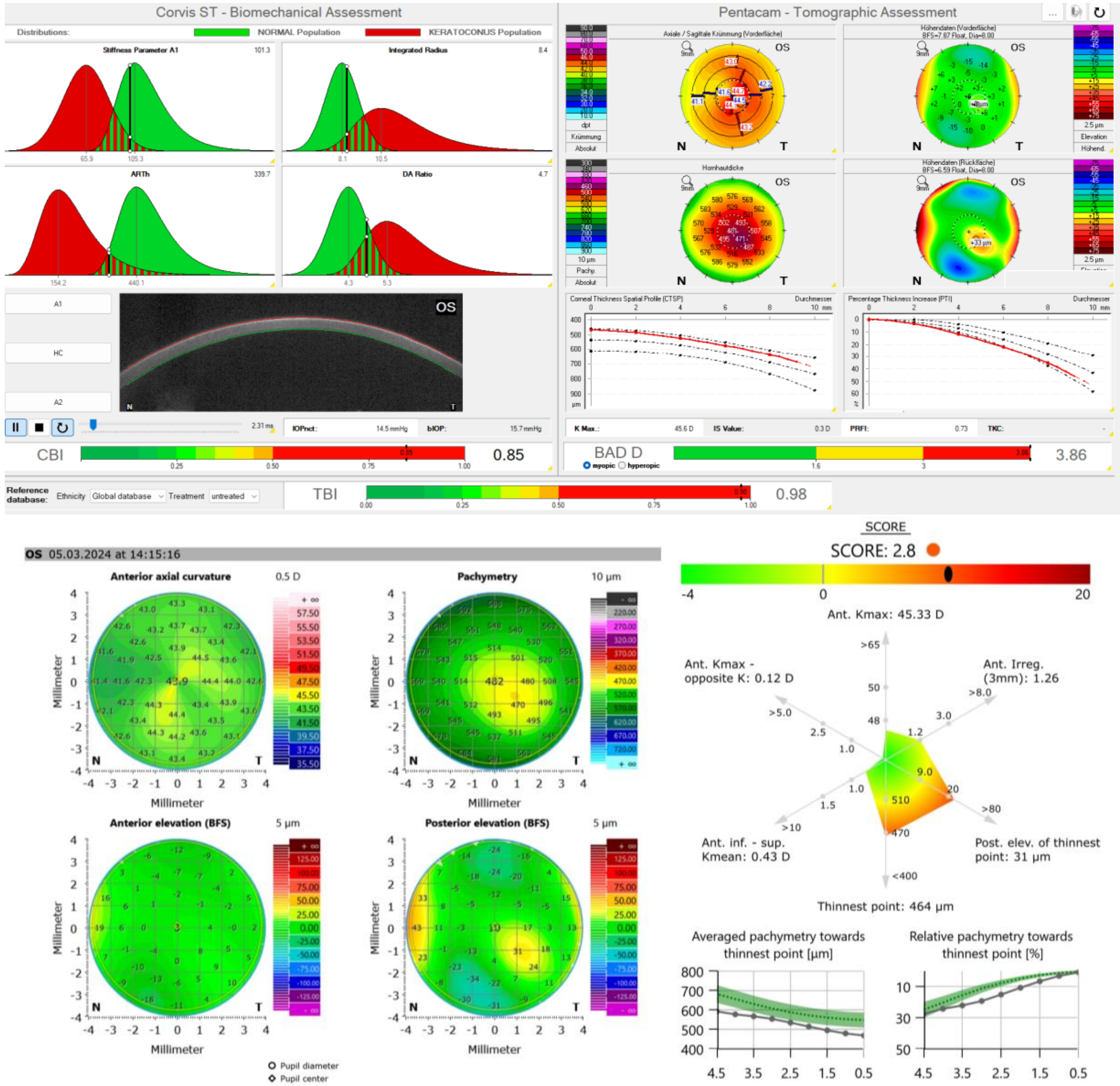

Corvis ST - Biomechanical Assessment
Pentacam - Tomographic Assessment
NORMAL Population
KERATOCONUS Population
Stiffness Parameter A1
Integrated Radius
ARTh
DA Ratio
OS
CBI
0.85
BAD D
3.86
TBI
0.98
OS 05.03.2024 at 14:15:16
Anterior axial curvature
Pachymetry
Anterior elevation (BFS)
Posterior elevation (BFS)
Millimeter
SCORE
SCORE: 2.8
Ant. Kmax: 45.33 D
Ant. Kmax - opposite K: 0.12 D
Ant. Irreg. (3mm): 1.26
Ant. inf. - sup. Kmean: 0.43 D
Post. elev. of thinnest point: 31 µm
Thinnest point: 464 µm
Averaged pachymetry towards thinnest point [µm]
Relative pachymetry towards thinnest point [%]
Pupil diameter
Pupil center

CASE #81

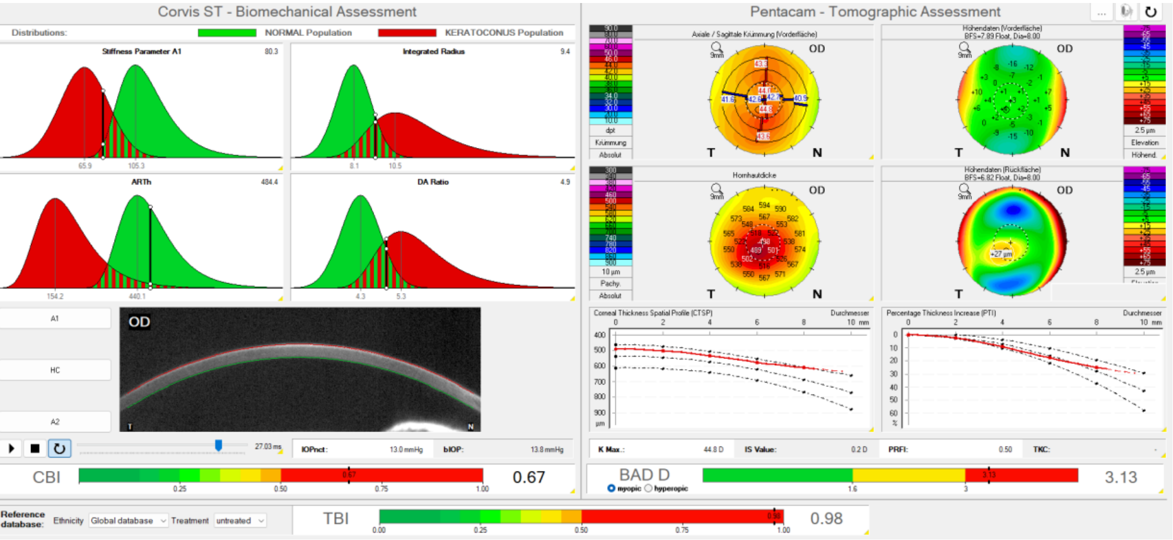
Corvis ST - Biomechanical Assessment
Pentacam - Tomographic Assessment
NORMAL Population
KERATOCONUS Population
Stiffness Parameter A1
Integrated Radius
ARTh
DA Ratio
OD
CBI 0.67
BAD D 3.13
TBI 0.98
K Max: 44.8 D
IS Value: 0.2 D
PRFI: 0.50
IOPnct: 13.0 mmHg
bIOP: 13.8 mmHg

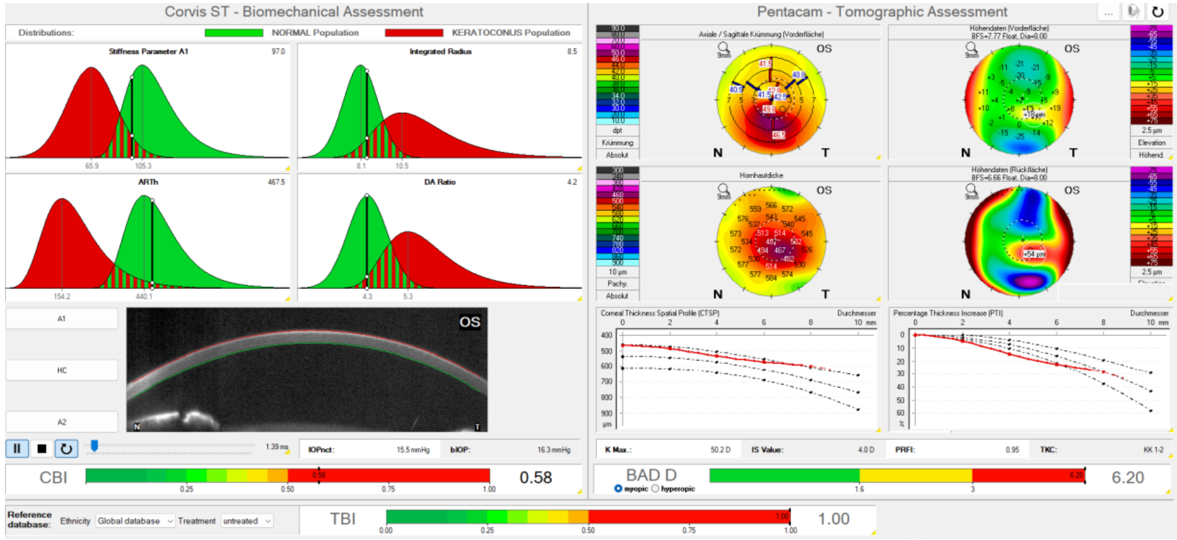
Corvis ST - Biomechanical Assessment
Pentacam - Tomographic Assessment
NORMAL Population
KERATOCONUS Population
Stiffness Parameter A1
Integrated Radius
ARTh
DA Ratio
OS
CBI 0.58
BAD D 6.20
TBI 1.00
K Max: 50.2 D
IS Value: 4.0 D
PRFI: 0.95
TKC: KK 1-2
IOPnct: 15.5 mmHg
bIOP: 16.3 mmHg

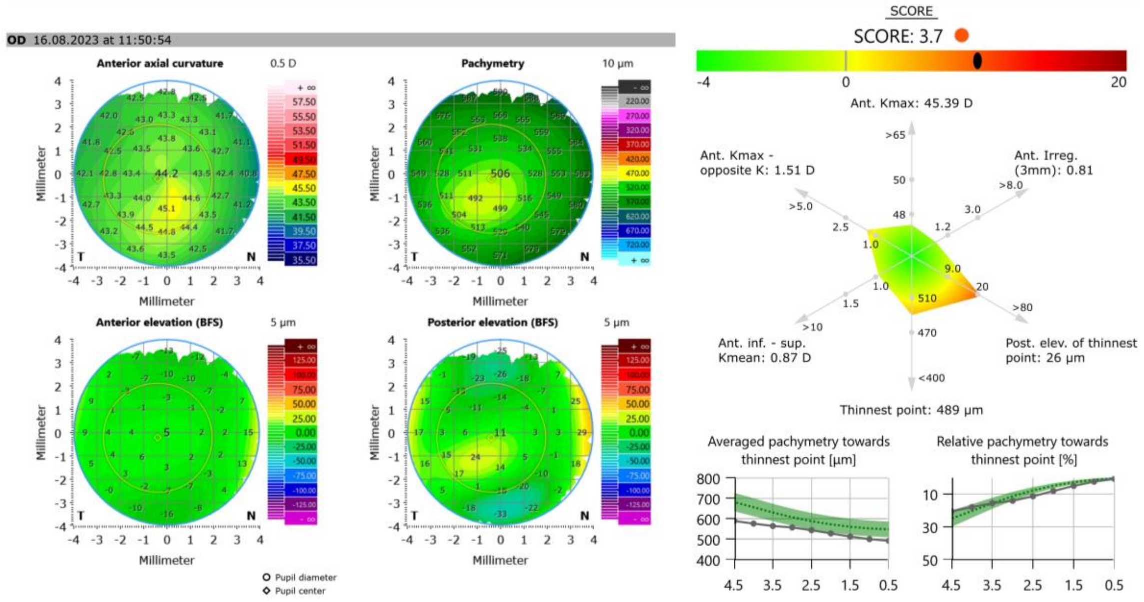
OD 16.08.2023 at 11:50:54
Anterior axial curvature
Pachymetry
Anterior elevation (BFS)
Posterior elevation (BFS)
Millimeter
SCORE
SCORE: 3.7
Ant. Kmax: 45.39 D
Ant. Kmax - opposite K: 1.51 D
Ant. Irreg. (3mm): 0.81
Ant. inf. - sup. Kmean: 0.87 D
Post. elev. of thinnest point: 26 µm
Thinnest point: 489 µm
Averaged pachymetry towards thinnest point [µm]
Relative pachymetry towards thinnest point [%]
Pupil diameter
Pupil center

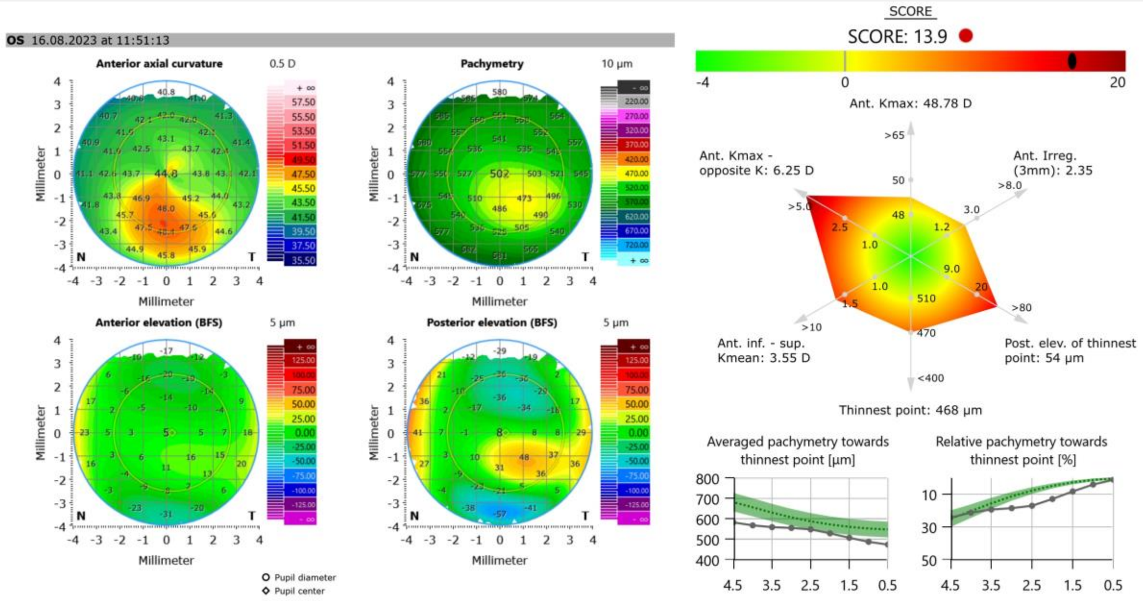
OS 16.08.2023 at 11:51:13
Anterior axial curvature
Pachymetry
Anterior elevation (BFS)
Posterior elevation (BFS)
Millimeter
SCORE
SCORE: 13.9
Ant. Kmax: 48.78 D
Ant. Kmax - opposite K: 6.25 D
Ant. Irreg. (3mm): 2.35
Ant. inf. - sup. Kmean: 3.55 D
Post. elev. of thinnest point: 54 µm
Thinnest point: 468 µm
Averaged pachymetry towards thinnest point [µm]
Relative pachymetry towards thinnest point [%]
Pupil diameter
Pupil center

CASE #82

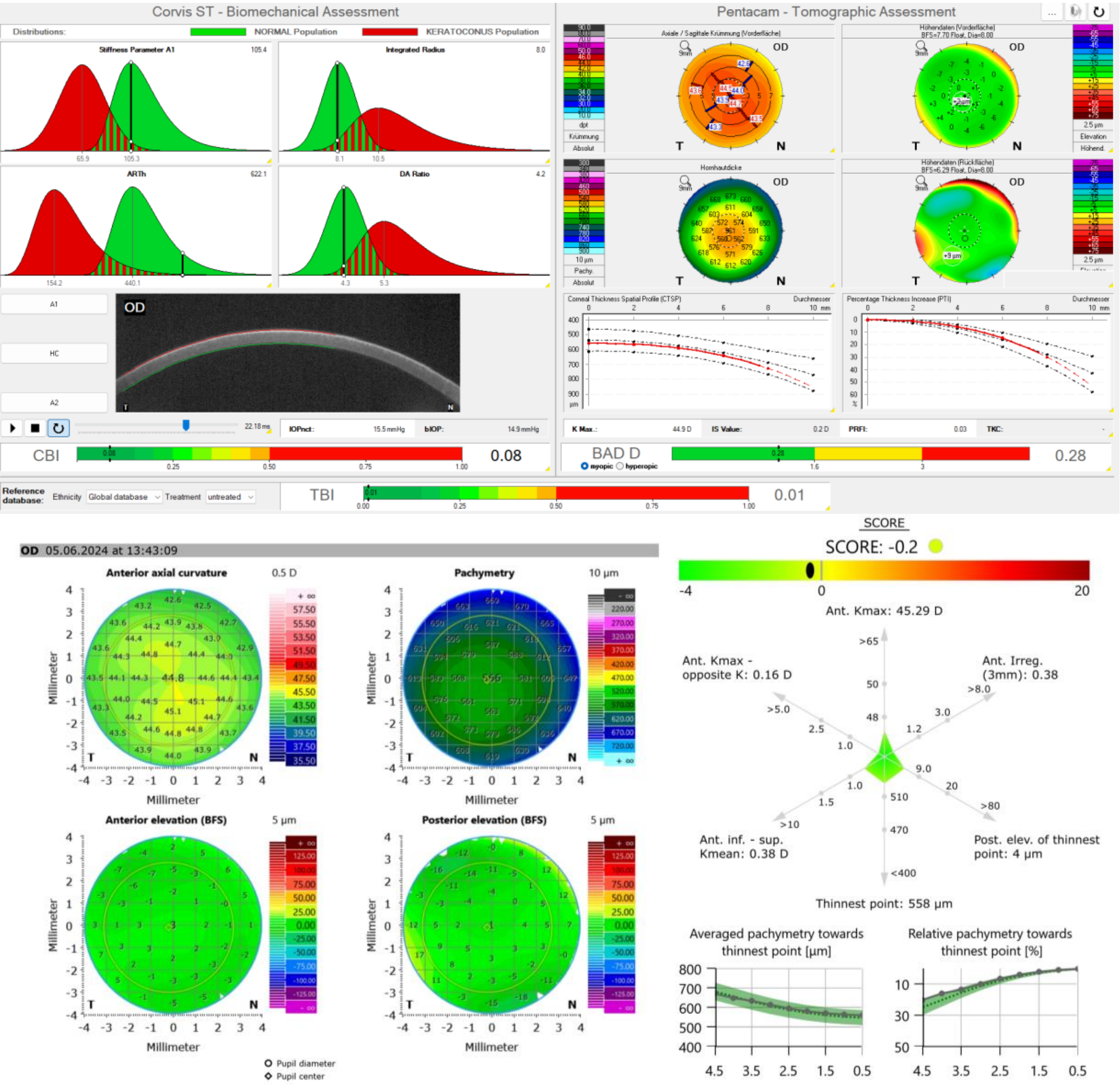

Corvis ST - Biomechanical Assessment
Pentacam - Tomographic Assessment
Distributions: NORMAL Population KERATOCONUS Population
Stiffness Parameter A1
Integrated Radius
ARTh
DA Ratio
OD
CBI 0.08
BAD D 0.28
TBI 0.01
Reference database: Ethnicity Global database Treatment untreated
OD 05.06.2024 at 13:43:09
Anterior axial curvature
Pachymetry
Anterior elevation (BFS)
Posterior elevation (BFS)
Millimeter
Pupil diameter
Pupil center
SCORE
SCORE: -0.2
Ant. Kmax: 45.29 D
Ant. Kmax - opposite K: 0.16 D
Ant. Irreg. (3mm): 0.38
Ant. inf. - sup. Kmean: 0.38 D
Post. elev. of thinnest point: 4 µm
Thinnest point: 558 µm
Averaged pachymetry towards thinnest point [µm]
Relative pachymetry towards thinnest point [%]

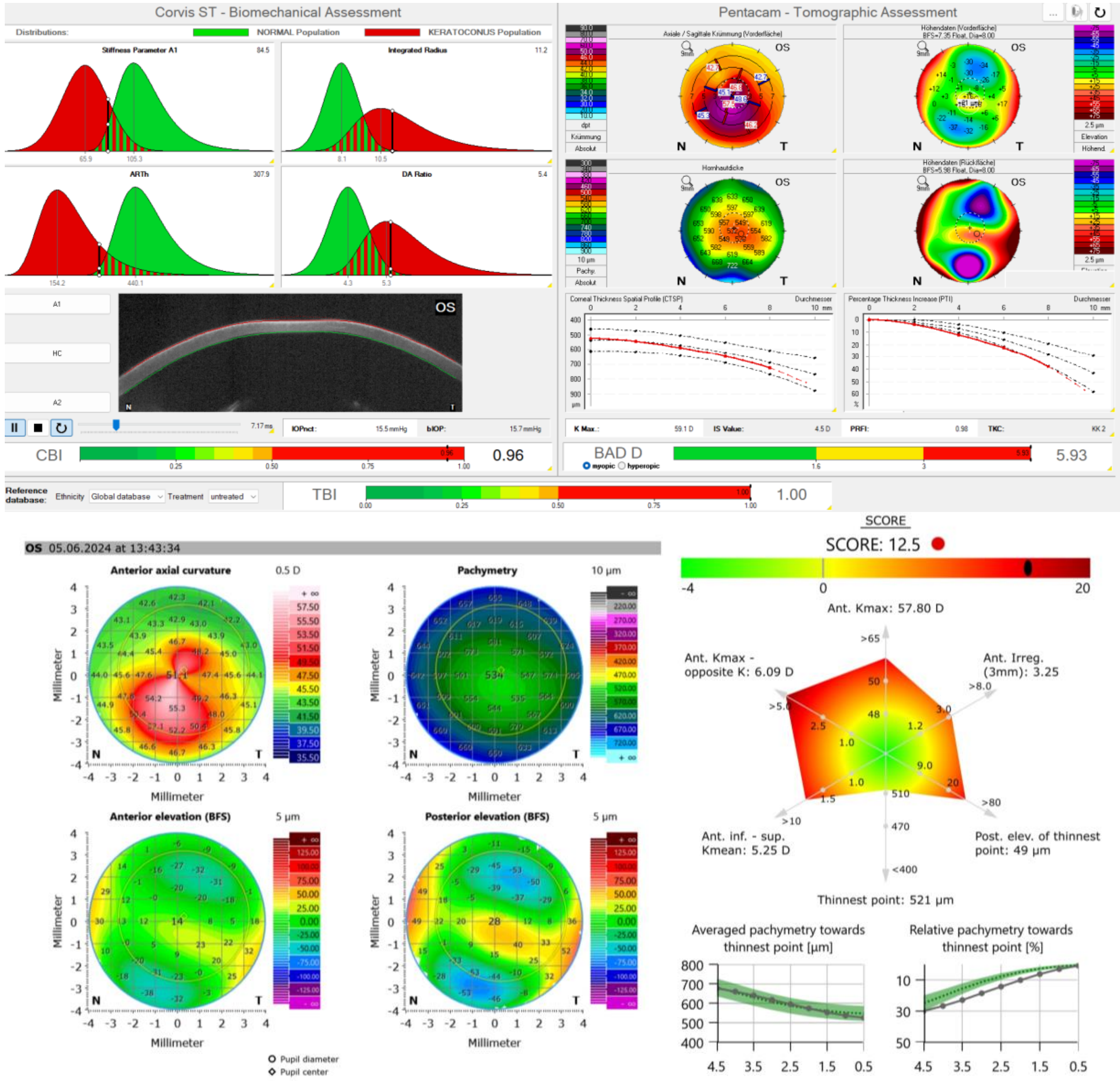

Corvis ST - Biomechanical Assessment
Pentacam - Tomographic Assessment
Distributions: NORMAL Population KERATOCONUS Population
Stiffness Parameter A1
Integrated Radius
ARTh
DA Ratio
OS
CBI 0.96
BAD D 5.93
TBI 1.00
Reference database: Ethnicity Global database Treatment untreated
OS 05.06.2024 at 13:43:34
Anterior axial curvature
Pachymetry
Anterior elevation (BFS)
Posterior elevation (BFS)
Millimeter
Pupil diameter
Pupil center
SCORE
SCORE: 12.5
Ant. Kmax: 57.80 D
Ant. Kmax - opposite K: 6.09 D
Ant. Irreg. (3mm): 3.25
Ant. inf. - sup. Kmean: 5.25 D
Post. elev. of thinnest point: 49 µm
Thinnest point: 521 µm
Averaged pachymetry towards thinnest point [µm]
Relative pachymetry towards thinnest point [%]

CASE #83

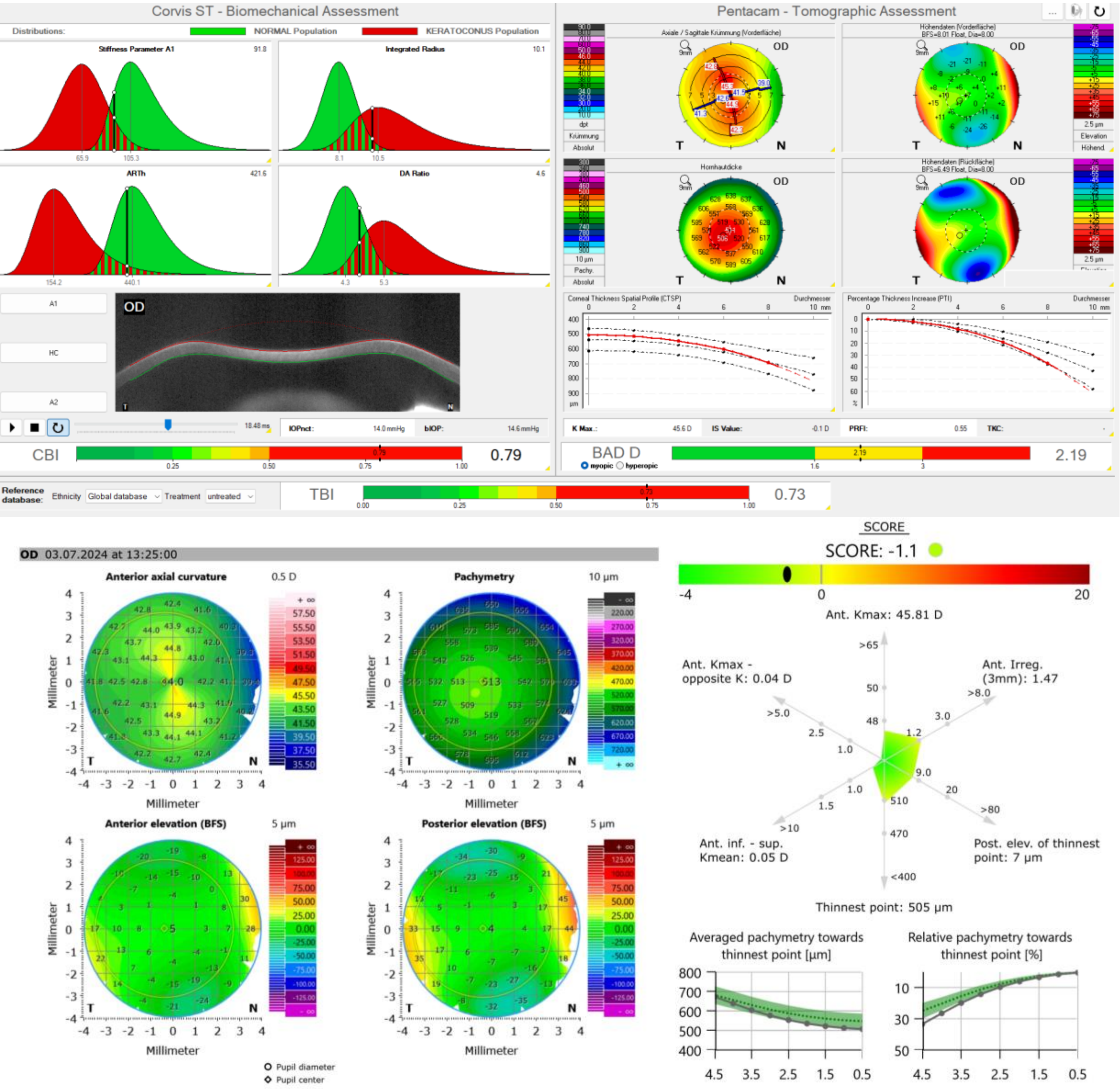

Corvis ST - Biomechanical Assessment
Pentacam - Tomographic Assessment
OD
CBI 0.79
BAD D 2.19
TBI 0.73
OD 03.07.2024 at 13:25:00
Anterior axial curvature
Pachymetry
Anterior elevation (BFS)
Posterior elevation (BFS)
Millimeter
SCORE
SCORE: -1.1
Ant. Kmax: 45.81 D
Ant. Kmax - opposite K: 0.04 D
Ant. Irreg. (3mm): 1.47
Ant. inf. - sup. Kmean: 0.05 D
Post. elev. of thinnest point: 7 µm
Thinnest point: 505 µm
Averaged pachymetry towards thinnest point [µm]
Relative pachymetry towards thinnest point [%]

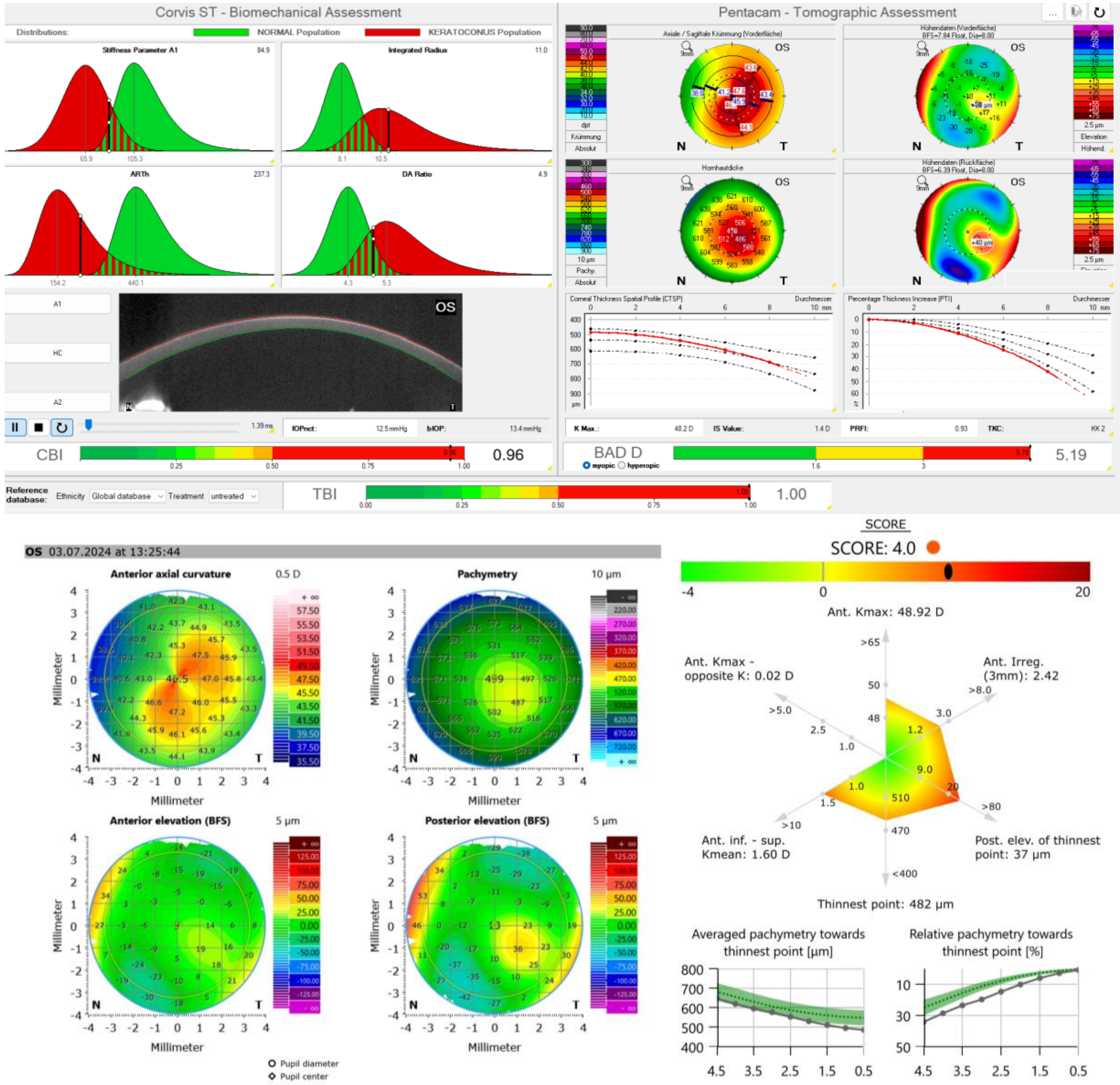

Corvis ST - Biomechanical Assessment
Pentacam - Tomographic Assessment
OS
CBI 0.96
BAD D 5.19
TBI 1.00
OS 03.07.2024 at 13:25:44
Anterior axial curvature
Pachymetry
Anterior elevation (BFS)
Posterior elevation (BFS)
Millimeter
SCORE
SCORE: 4.0
Ant. Kmax: 48.92 D
Ant. Kmax - opposite K: 0.02 D
Ant. Irreg. (3mm): 2.42
Ant. inf. - sup. Kmean: 1.60 D
Post. elev. of thinnest point: 37 µm
Thinnest point: 482 µm
Averaged pachymetry towards thinnest point [µm]
Relative pachymetry towards thinnest point [%]

CASE #84

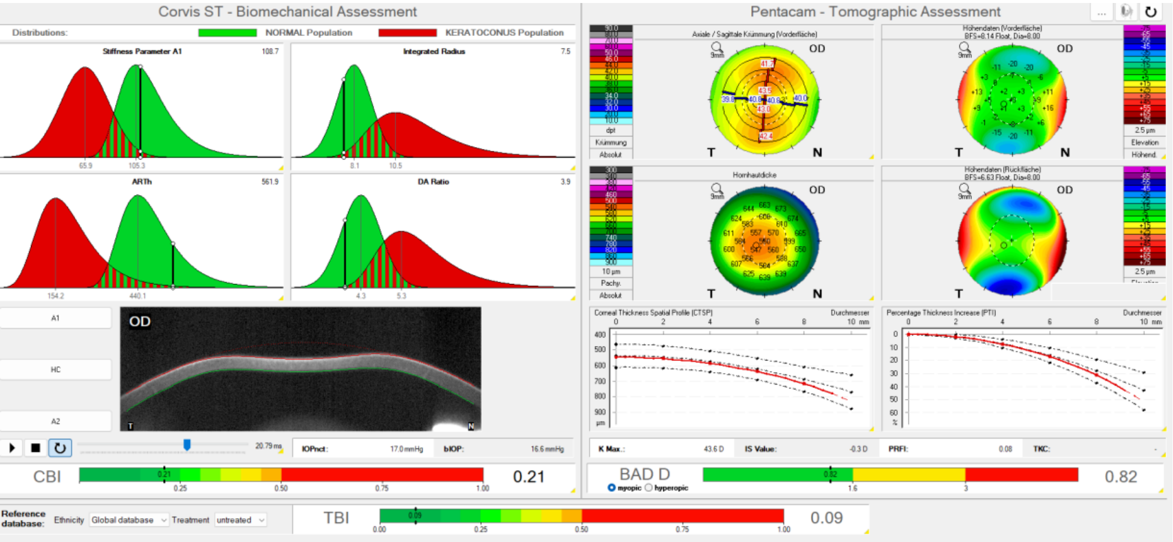
Corvis ST - Biomechanical Assessment
Pentacam - Tomographic Assessment
NORMAL Population
KERATOCONUS Population
Stiffness Parameter A1
Integrated Radius
ARTh
DA Ratio
OD
CBI
0.21
BAD D
0.82
TBI
0.09

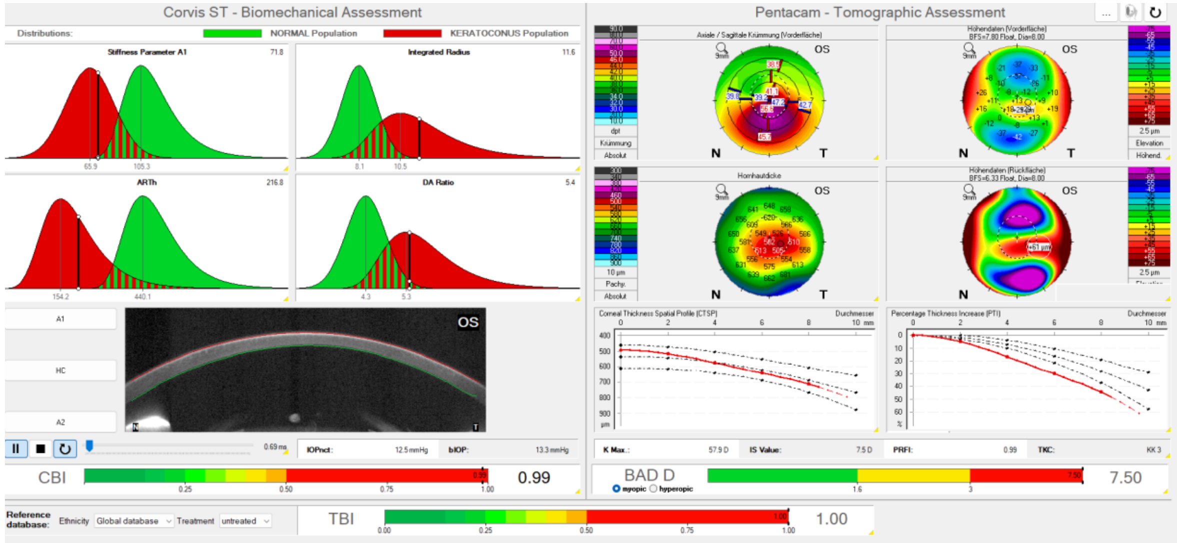
Corvis ST - Biomechanical Assessment
Pentacam - Tomographic Assessment
NORMAL Population
KERATOCONUS Population
Stiffness Parameter A1
Integrated Radius
ARTh
DA Ratio
OS
CBI
0.99
BAD D
7.50
TBI
1.00

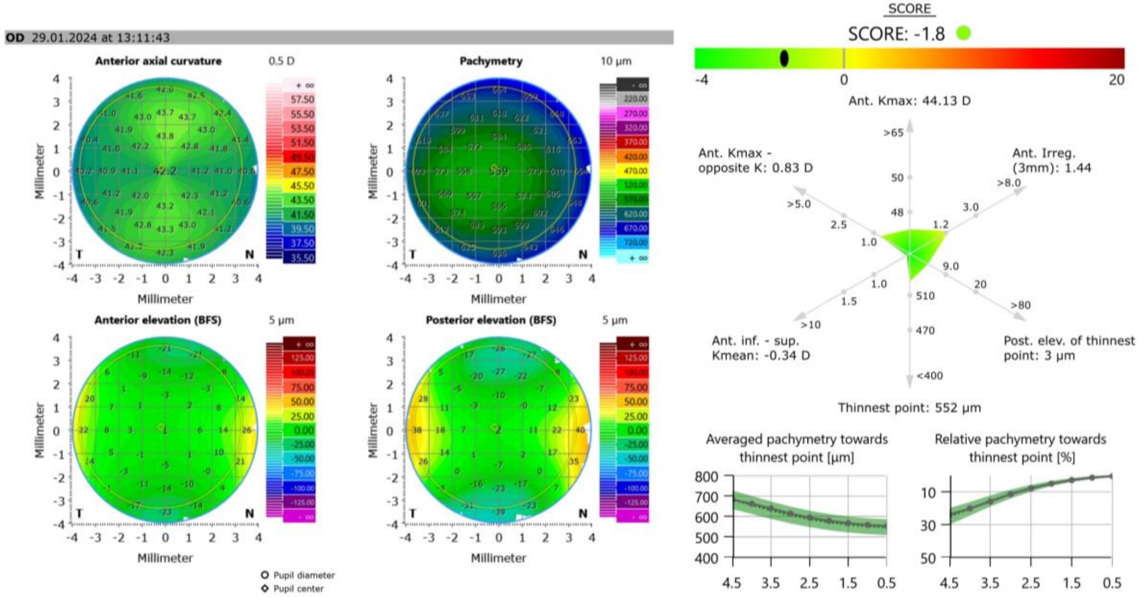
OD 29.01.2024 at 13:11:43
Anterior axial curvature
Pachymetry
Anterior elevation (BFS)
Posterior elevation (BFS)
Millimeter
SCORE
SCORE: -1.8
Ant. Kmax: 44.13 D
Ant. Kmax - opposite K: 0.83 D
Ant. Irreg. (3mm): 1.44
Ant. inf. - sup. Kmean: -0.34 D
Post. elev. of thinnest point: 3 µm
Thinnest point: 552 µm
Averaged pachymetry towards thinnest point [µm]
Relative pachymetry towards thinnest point [%]
Pupil diameter
Pupil center

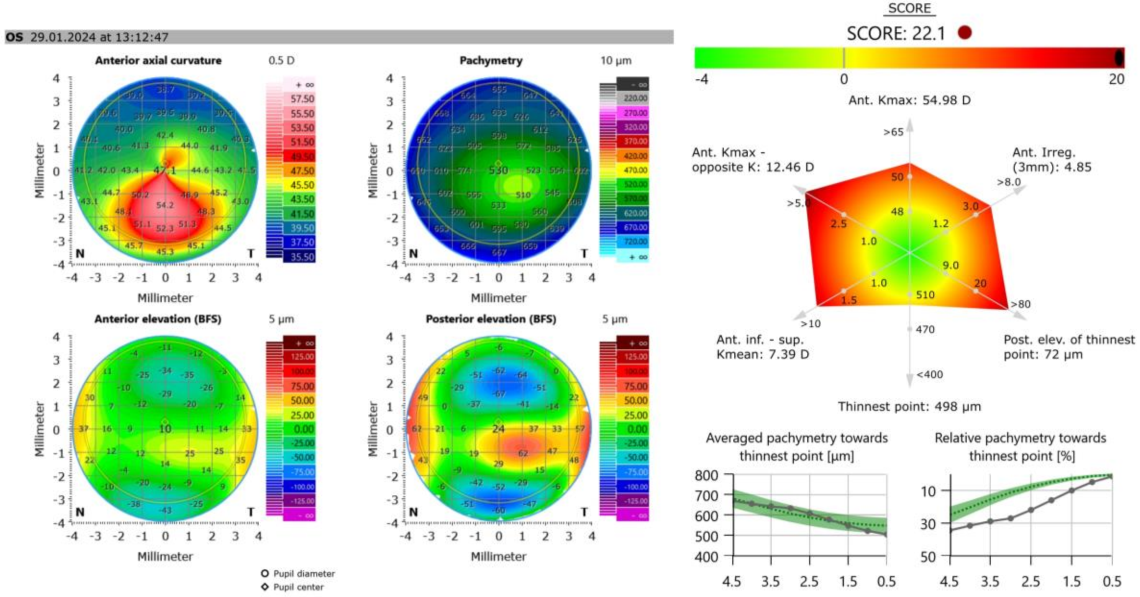
OS 29.01.2024 at 13:12:47
Anterior axial curvature
Pachymetry
Anterior elevation (BFS)
Posterior elevation (BFS)
Millimeter
SCORE
SCORE: 22.1
Ant. Kmax: 54.98 D
Ant. Kmax - opposite K: 12.46 D
Ant. Irreg. (3mm): 4.85
Ant. inf. - sup. Kmean: 7.39 D
Post. elev. of thinnest point: 72 µm
Thinnest point: 498 µm
Averaged pachymetry towards thinnest point [µm]
Relative pachymetry towards thinnest point [%]
Pupil diameter
Pupil center

CASE #85

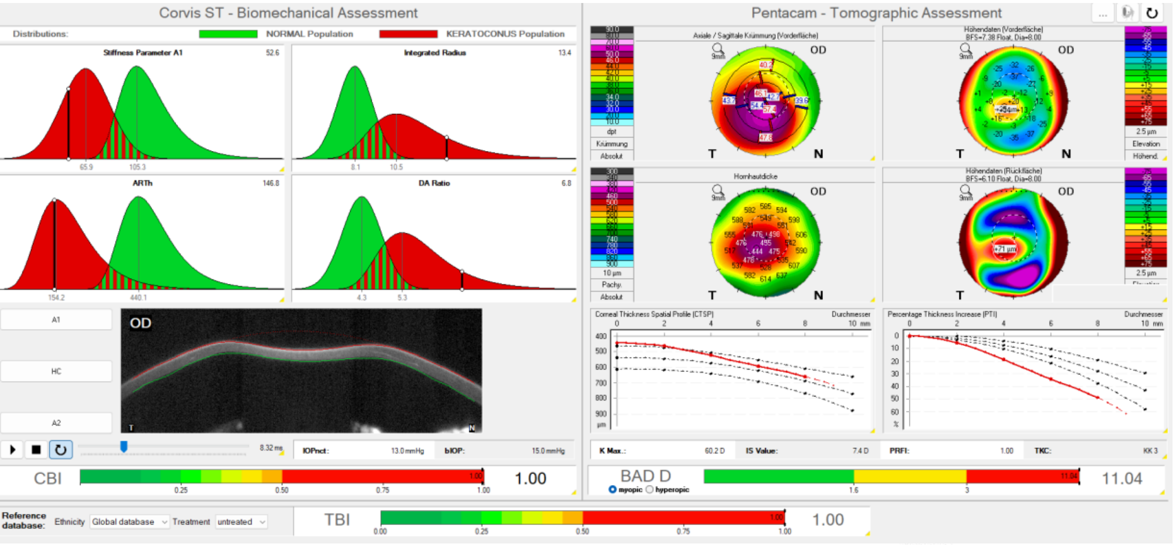

Corvis ST - Biomechanical Assessment
Pentacam - Tomographic Assessment
NORMAL Population
KERATOCONUS Population
Stiffness Parameter A1
Integrated Radius
ARTh
DA Ratio
OD
CBI
1.00
BAD D
11.04
TBI
1.00

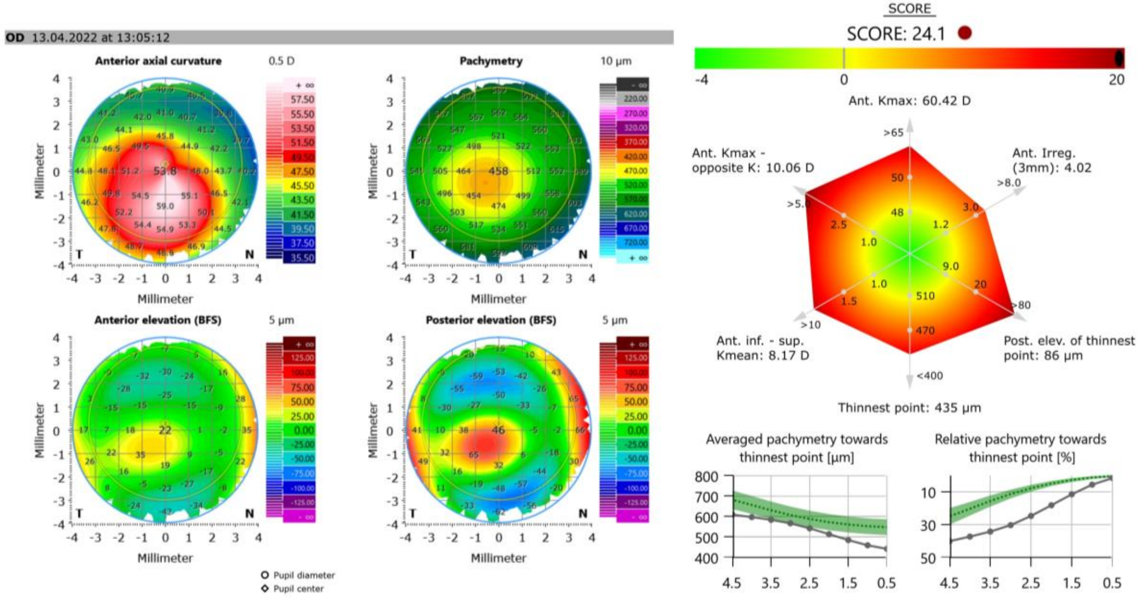

OD 13.04.2022 at 13:05:12
Anterior axial curvature
Pachymetry
Anterior elevation (BFS)
Posterior elevation (BFS)
Millimeter
SCORE
SCORE: 24.1
Ant. Kmax: 60.42 D
Ant. Kmax - opposite K: 10.06 D
Ant. Irreg. (3mm): 4.02
Ant. inf. - sup. Kmean: 8.17 D
Post. elev. of thinnest point: 86 µm
Thinnest point: 435 µm
Averaged pachymetry towards thinnest point [µm]
Relative pachymetry towards thinnest point [%]
Pupil diameter
Pupil center

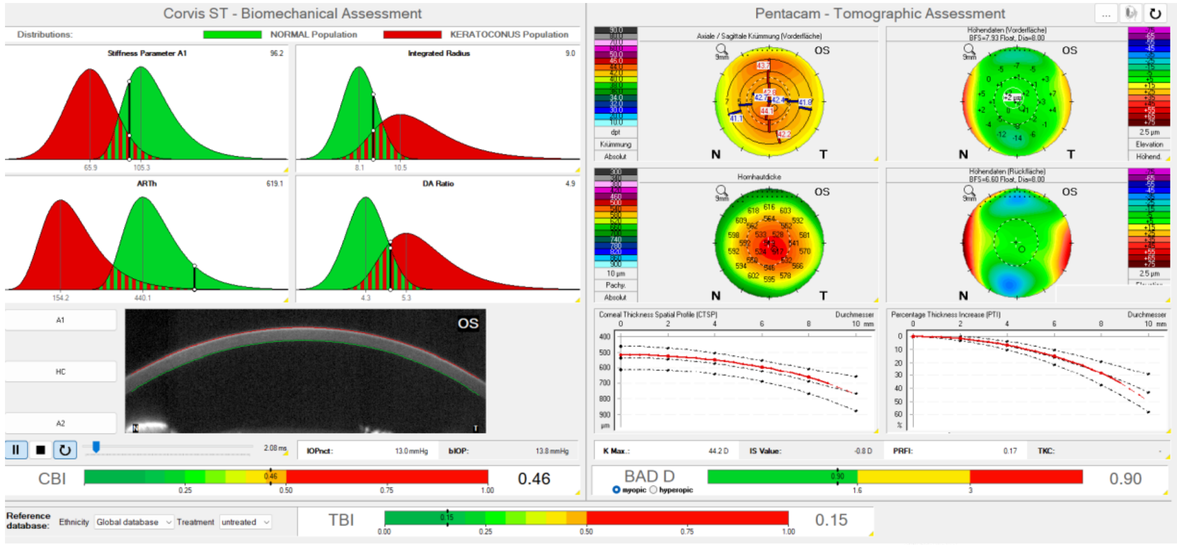

Corvis ST - Biomechanical Assessment
Pentacam - Tomographic Assessment
NORMAL Population
KERATOCONUS Population
Stiffness Parameter A1
Integrated Radius
ARTh
DA Ratio
OS
CBI
0.46
BAD D
0.90
TBI
0.15

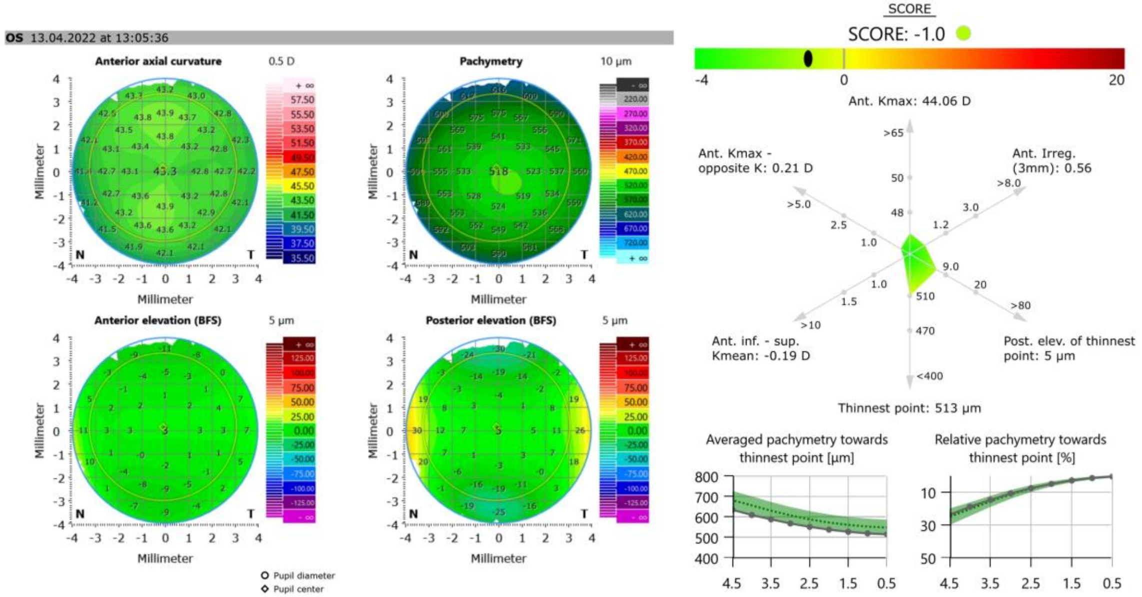

OS 13.04.2022 at 13:05:36
Anterior axial curvature
Pachymetry
Anterior elevation (BFS)
Posterior elevation (BFS)
Millimeter
SCORE
SCORE: -1.0
Ant. Kmax: 44.06 D
Ant. Kmax - opposite K: 0.21 D
Ant. Irreg. (3mm): 0.56
Ant. inf. - sup. Kmean: -0.19 D
Post. elev. of thinnest point: 5 µm
Thinnest point: 513 µm
Averaged pachymetry towards thinnest point [µm]
Relative pachymetry towards thinnest point [%]
Pupil diameter
Pupil center

CASE #86

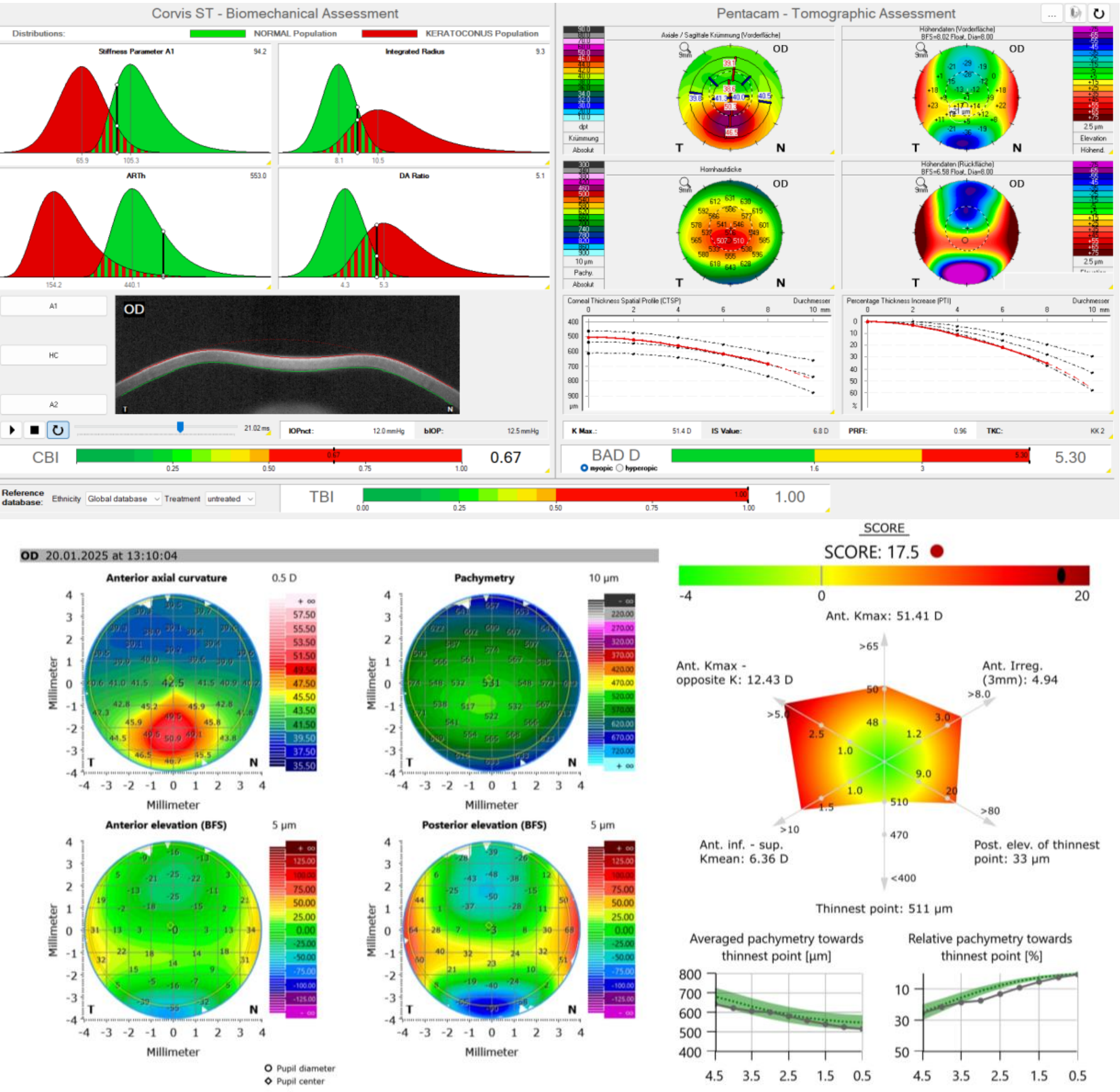

Corvis ST - Biomechanical Assessment
Pentacam - Tomographic Assessment
OD
CBI
0.67
BAD D
5.30
TBI
1.00
OD 20.01.2025 at 13:10:04
Anterior axial curvature
Pachymetry
Anterior elevation (BFS)
Posterior elevation (BFS)
Millimeter
SCORE
SCORE: 17.5
Ant. Kmax: 51.41 D
Ant. Kmax - opposite K: 12.43 D
Ant. Irreg. (3mm): 4.94
Ant. inf. - sup. Kmean: 6.36 D
Post. elev. of thinnest point: 33 µm
Thinnest point: 511 µm
Averaged pachymetry towards thinnest point [µm]
Relative pachymetry towards thinnest point [%]
Pupil diameter
Pupil center

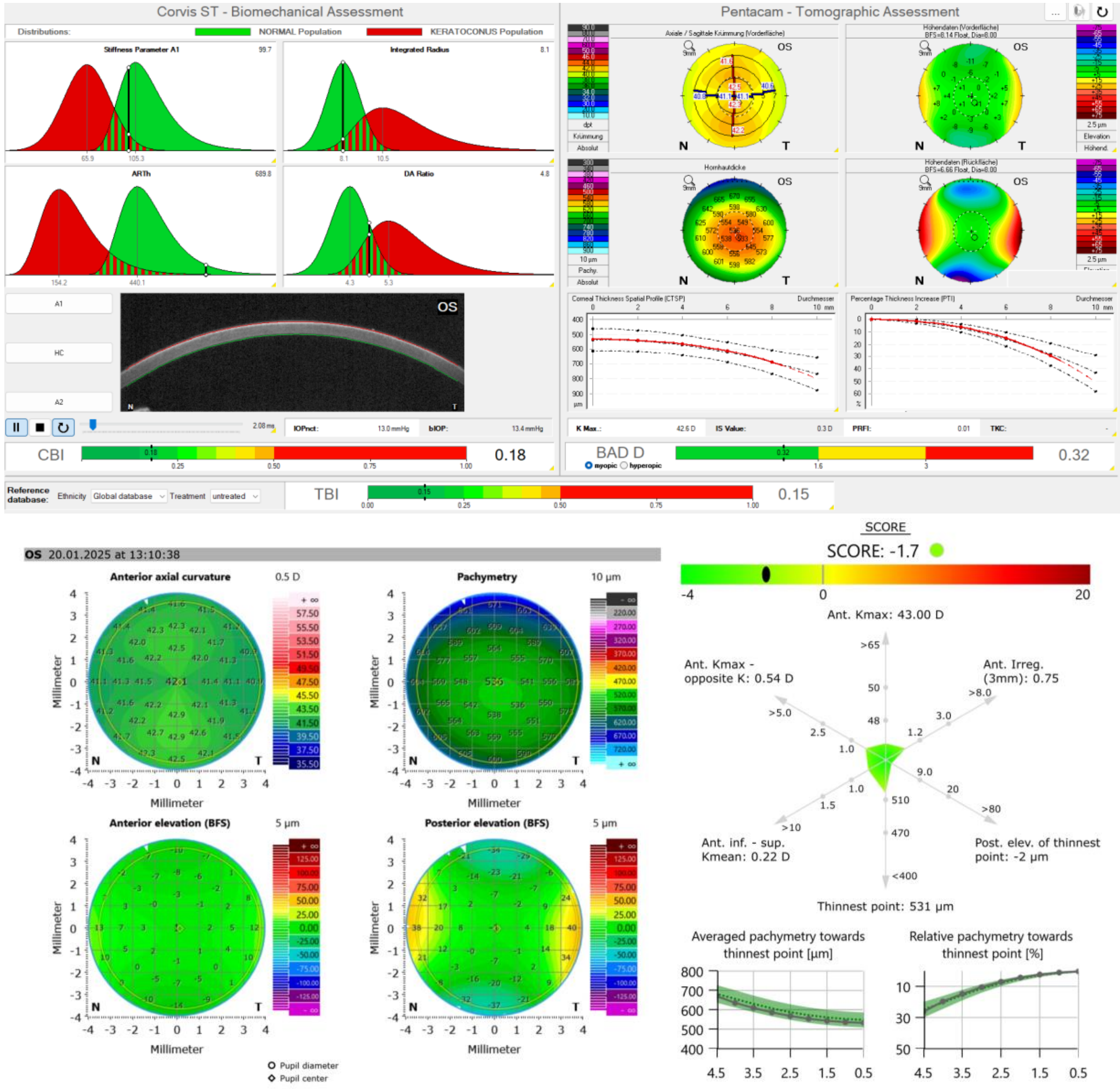

Corvis ST - Biomechanical Assessment
Pentacam - Tomographic Assessment
OS
CBI
0.18
BAD D
0.32
TBI
0.15
OS 20.01.2025 at 13:10:38
Anterior axial curvature
Pachymetry
Anterior elevation (BFS)
Posterior elevation (BFS)
Millimeter
SCORE
SCORE: -1.7
Ant. Kmax: 43.00 D
Ant. Kmax - opposite K: 0.54 D
Ant. Irreg. (3mm): 0.75
Ant. inf. - sup. Kmean: 0.22 D
Post. elev. of thinnest point: -2 µm
Thinnest point: 531 µm
Averaged pachymetry towards thinnest point [µm]
Relative pachymetry towards thinnest point [%]
Pupil diameter
Pupil center

CASE #87

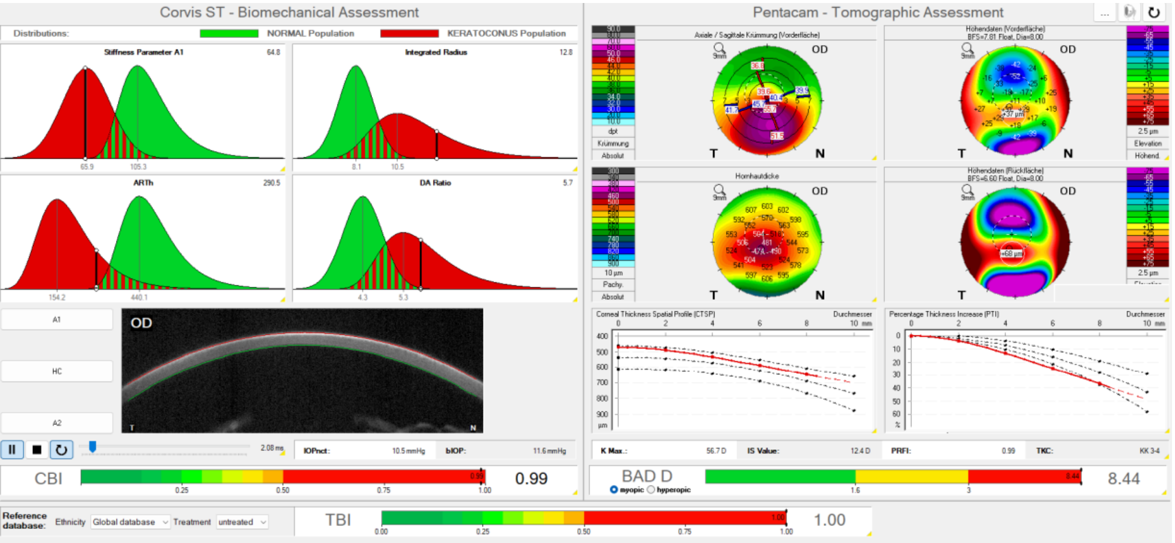

Corvis ST - Biomechanical Assessment
Pentacam - Tomographic Assessment
Distributions: NORMAL Population KERATOCONUS Population
Stiffness Parameter A1
Integrated Radius
ARTh
DA Ratio
OD
IOPnct: 10.5 mmHg
bIOP: 11.6 mmHg
K Max.: 56.7 D
IS Value: 12.4 D
PRFI: 0.99
TKC: KK 3-4
CBI 0.99
BAD D 8.44
myopic hyperopic
Reference database: Ethnicity Global database Treatment untreated
TBI 1.00

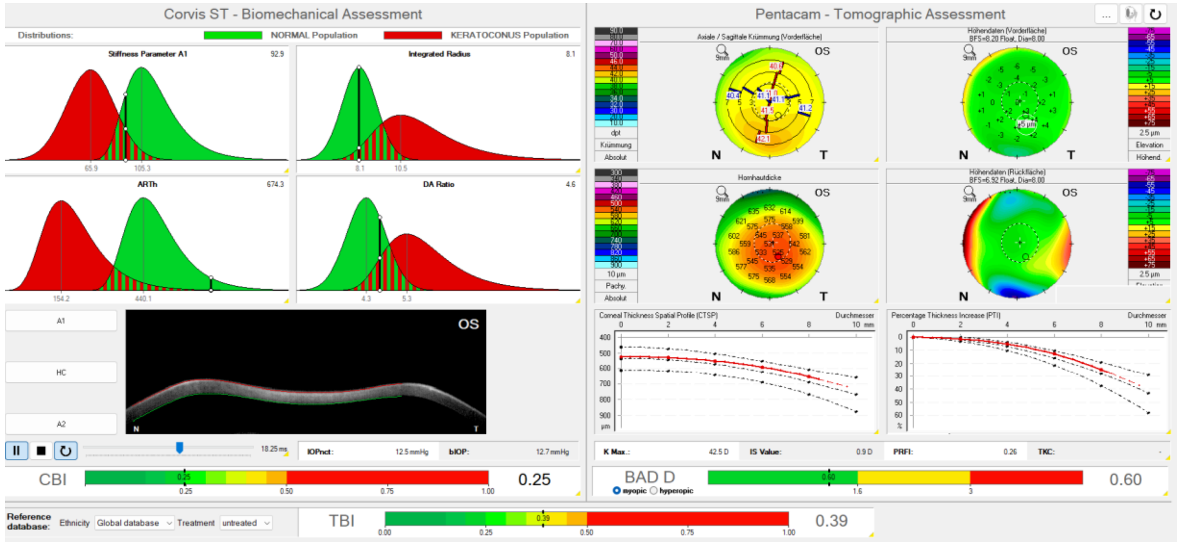

Corvis ST - Biomechanical Assessment
Pentacam - Tomographic Assessment
Distributions: NORMAL Population KERATOCONUS Population
Stiffness Parameter A1
Integrated Radius
ARTh
DA Ratio
OS
IOPnct: 12.5 mmHg
bIOP: 12.7 mmHg
K Max.: 42.5 D
IS Value: 0.9 D
PRFI: 0.26
TKC:
CBI 0.25
BAD D 0.60
myopic hyperopic
Reference database: Ethnicity Global database Treatment untreated
TBI 0.39

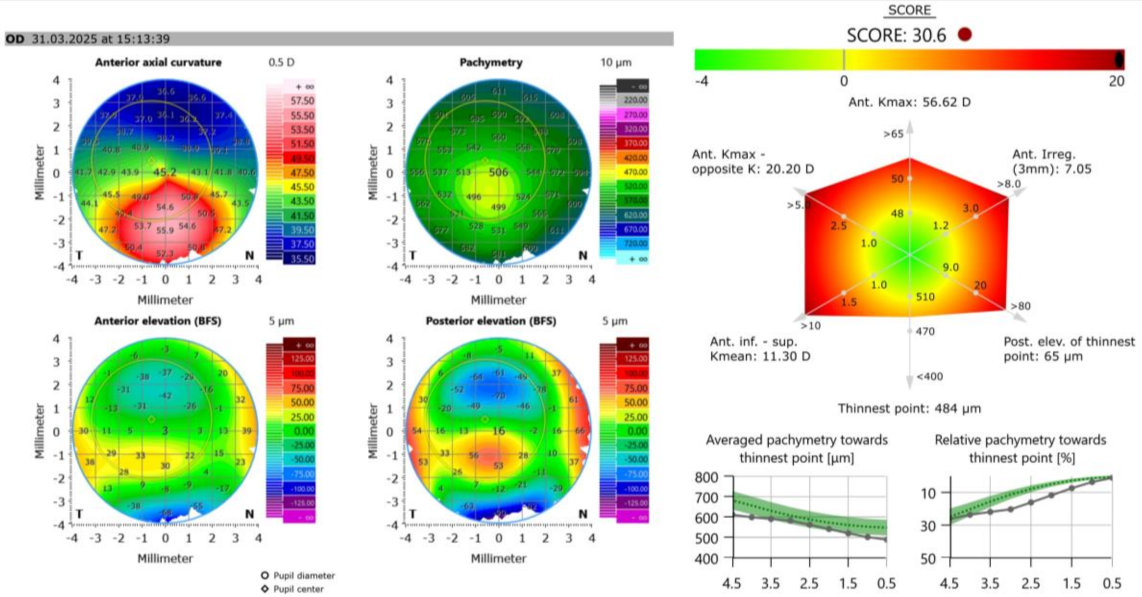

OD 31.03.2025 at 15:13:39
Anterior axial curvature
Pachymetry
Anterior elevation (BFS)
Posterior elevation (BFS)
Millimeter
Pupil diameter
Pupil center
SCORE
SCORE: 30.6
Ant. Kmax: 56.62 D
Ant. Kmax - opposite K: 20.20 D
Ant. Irreg. (3mm): 7.05
Ant. inf. - sup. Kmean: 11.30 D
Post. elev. of thinnest point: 65 µm
Thinnest point: 484 µm
Averaged pachymetry towards thinnest point [µm]
Relative pachymetry towards thinnest point [%]

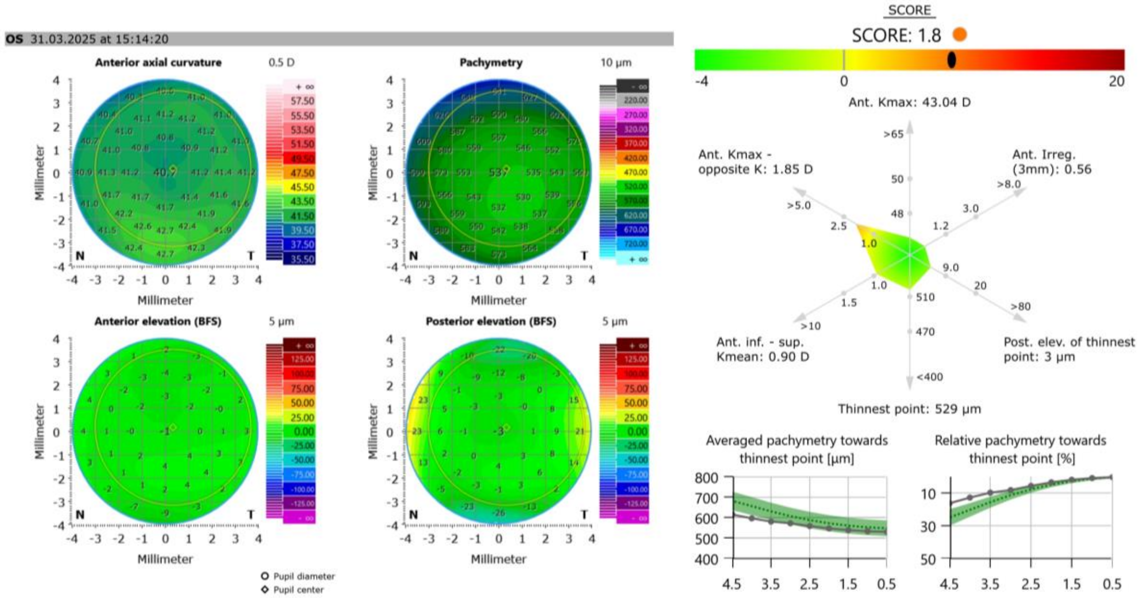

OS 31.03.2025 at 15:14:20
Anterior axial curvature
Pachymetry
Anterior elevation (BFS)
Posterior elevation (BFS)
Millimeter
Pupil diameter
Pupil center
SCORE
SCORE: 1.8
Ant. Kmax: 43.04 D
Ant. Kmax - opposite K: 1.85 D
Ant. Irreg. (3mm): 0.56
Ant. inf. - sup. Kmean: 0.90 D
Post. elev. of thinnest point: 3 µm
Thinnest point: 529 µm
Averaged pachymetry towards thinnest point [µm]
Relative pachymetry towards thinnest point [%]

CASE #88

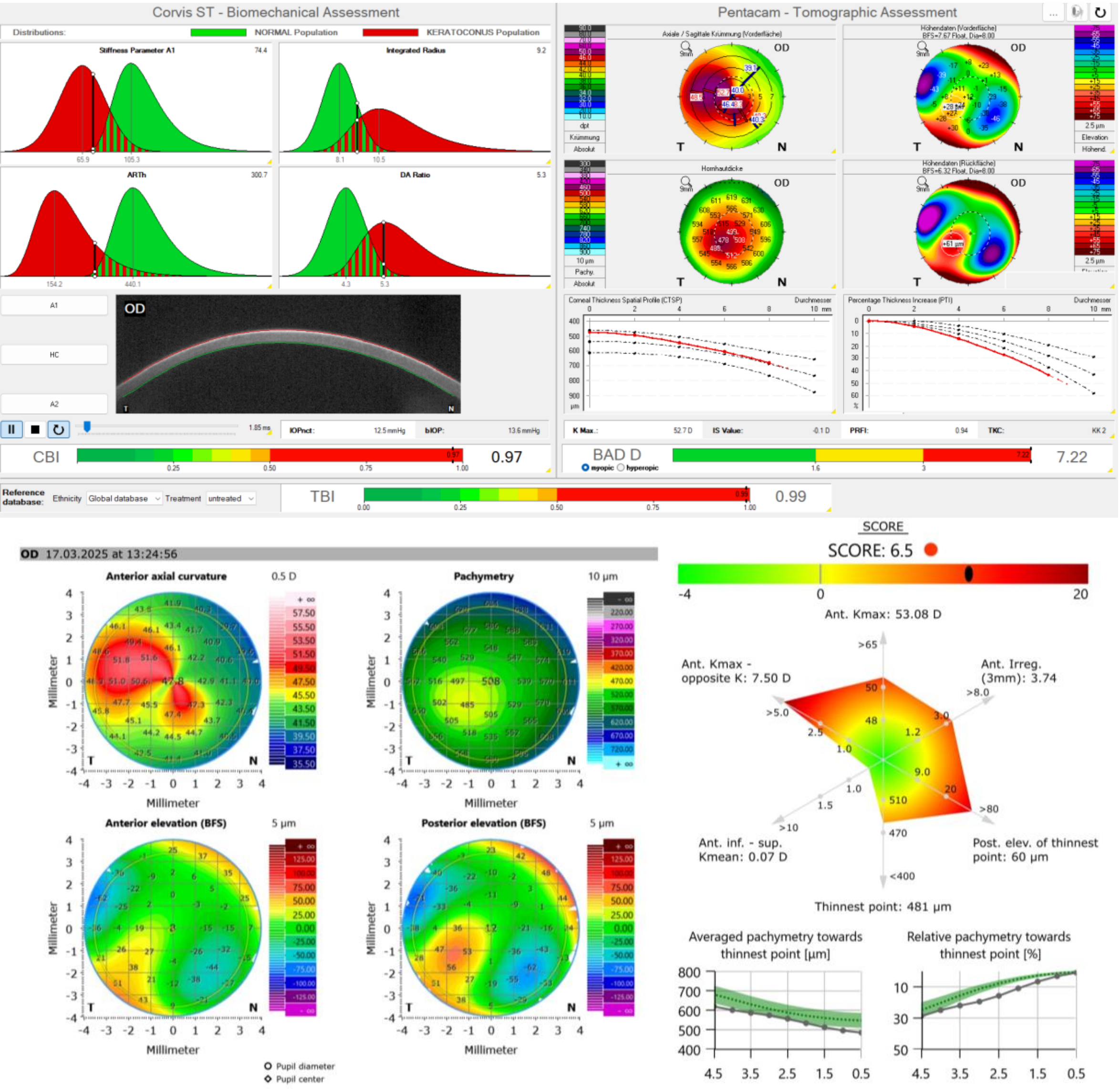
Corvis ST - Biomechanical Assessment
Pentacam - Tomographic Assessment
NORMAL Population
KERATOCONUS Population
Stiffness Parameter A1
Integrated Radius
ARTh
DA Ratio
OD
IOPnct: 12.5 mmHg
bIOP: 13.6 mmHg
K Max: 52.7 D
IS Value: -0.1 D
PRFI: 0.94
TKC: KK 2
CBI 0.97
BAD D 7.22
TBI 0.99
Reference database: Ethnicity Global database Treatment untreated
OD 17.03.2025 at 13:24:56
Anterior axial curvature 0.5 D
Pachymetry 10 µm
Anterior elevation (BFS) 5 µm
Posterior elevation (BFS) 5 µm
Millimeter
Pupil diameter
Pupil center
SCORE
SCORE: 6.5
Ant. Kmax: 53.08 D
Ant. Kmax - opposite K: 7.50 D
Ant. Irreg. (3mm): 3.74
Ant. inf. - sup. Kmean: 0.07 D
Post. elev. of thinnest point: 60 µm
Thinnest point: 481 µm
Averaged pachymetry towards thinnest point [µm]
Relative pachymetry towards thinnest point [%]

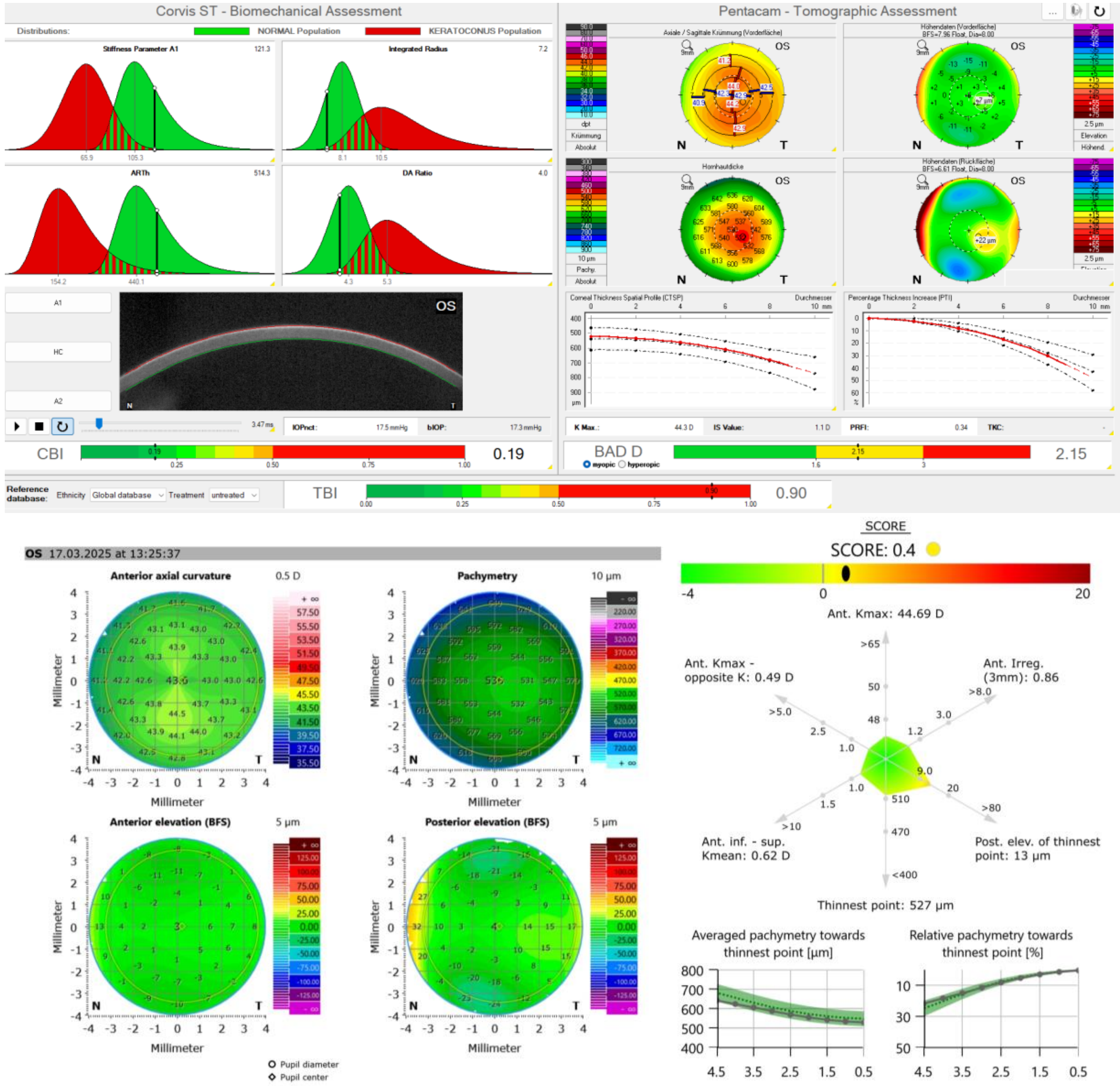
Corvis ST - Biomechanical Assessment
Pentacam - Tomographic Assessment
NORMAL Population
KERATOCONUS Population
Stiffness Parameter A1
Integrated Radius
ARTh
DA Ratio
OS
IOPnct: 17.5 mmHg
bIOP: 17.3 mmHg
K Max: 44.3 D
IS Value: 1.1 D
PRFI: 0.34
TKC:
CBI 0.19
BAD D 2.15
TBI 0.90
Reference database: Ethnicity Global database Treatment untreated
OS 17.03.2025 at 13:25:37
Anterior axial curvature 0.5 D
Pachymetry 10 µm
Anterior elevation (BFS) 5 µm
Posterior elevation (BFS) 5 µm
Millimeter
Pupil diameter
Pupil center
SCORE
SCORE: 0.4
Ant. Kmax: 44.69 D
Ant. Kmax - opposite K: 0.49 D
Ant. Irreg. (3mm): 0.86
Ant. inf. - sup. Kmean: 0.62 D
Post. elev. of thinnest point: 13 µm
Thinnest point: 527 µm
Averaged pachymetry towards thinnest point [µm]
Relative pachymetry towards thinnest point [%]

CASE #89

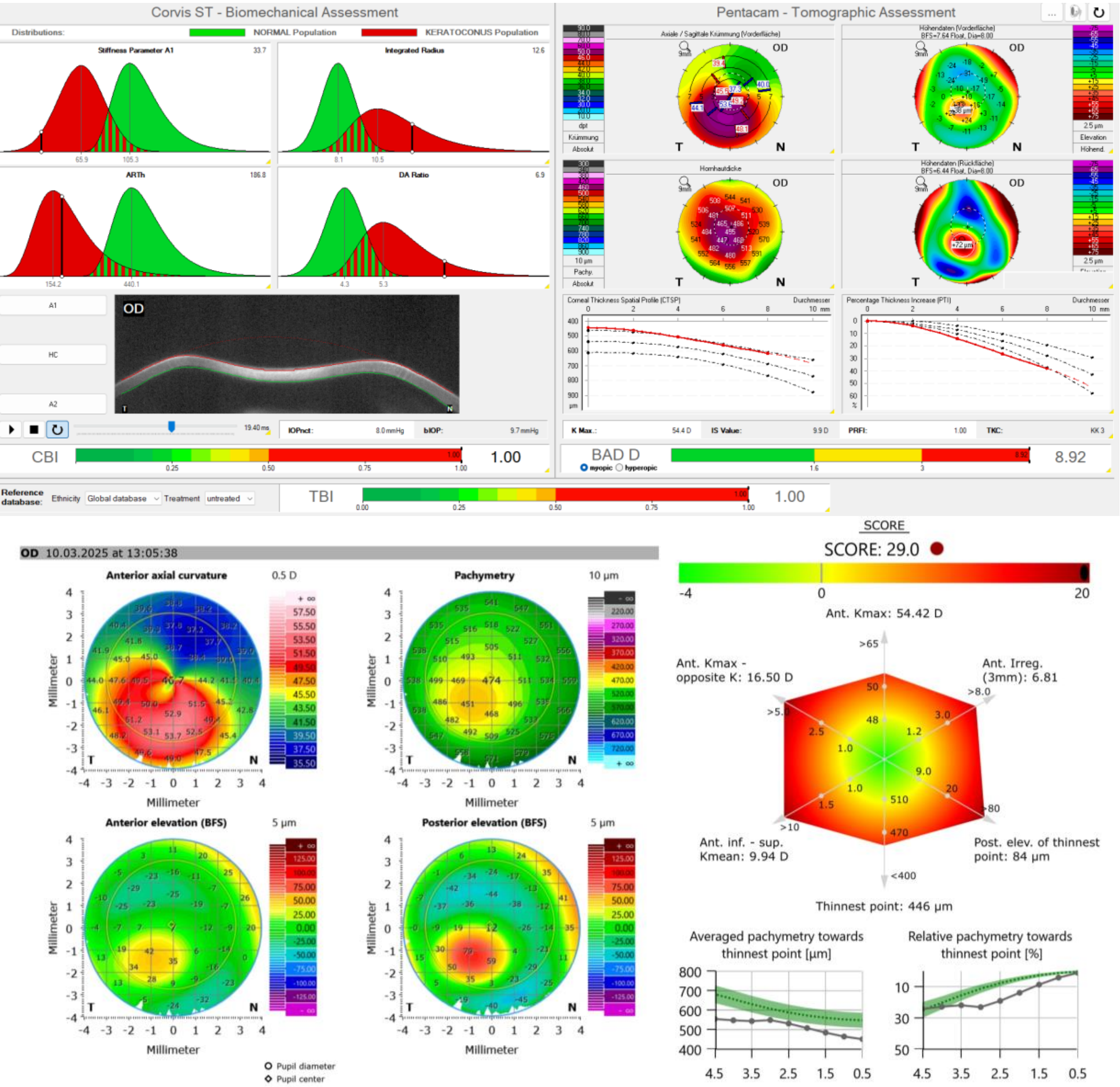
Corvis ST - Biomechanical Assessment
Pentacam - Tomographic Assessment
OD
CBI 1.00
BAD D 8.92
TBI 1.00
OD 10.03.2025 at 13:05:38
Anterior axial curvature
Pachymetry
Anterior elevation (BFS)
Posterior elevation (BFS)
SCORE
SCORE: 29.0
Ant. Kmax: 54.42 D
Ant. Kmax - opposite K: 16.50 D
Ant. Irreg. (3mm): 6.81
Ant. inf. - sup. Kmean: 9.94 D
Post. elev. of thinnest point: 84 µm
Thinnest point: 446 µm
Averaged pachymetry towards thinnest point [µm]
Relative pachymetry towards thinnest point [%]
Pupil diameter
Pupil center

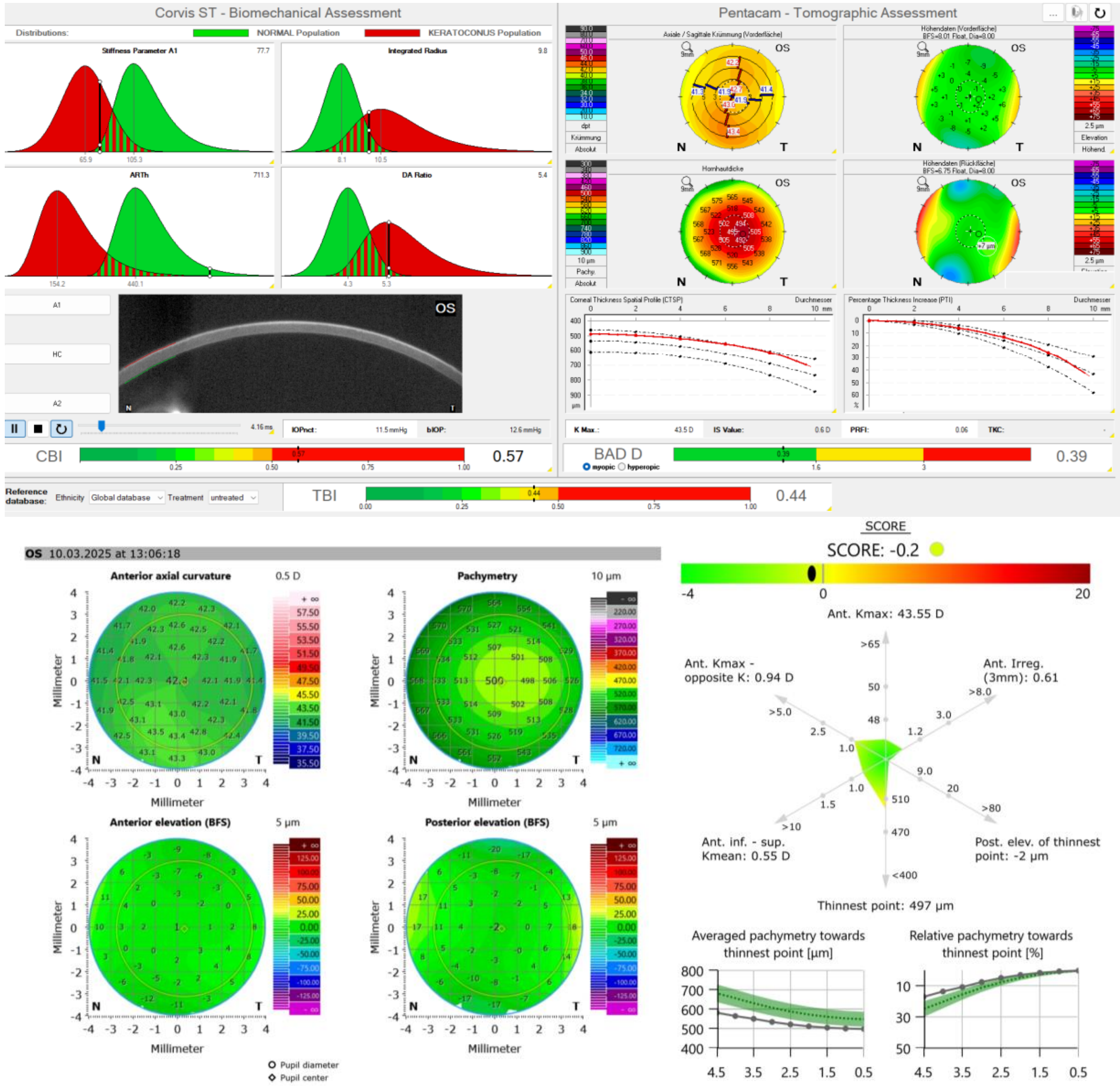
Corvis ST - Biomechanical Assessment
Pentacam - Tomographic Assessment
OS
CBI 0.57
BAD D 0.39
TBI 0.44
OS 10.03.2025 at 13:06:18
Anterior axial curvature
Pachymetry
Anterior elevation (BFS)
Posterior elevation (BFS)
SCORE
SCORE: -0.2
Ant. Kmax: 43.55 D
Ant. Kmax - opposite K: 0.94 D
Ant. Irreg. (3mm): 0.61
Ant. inf. - sup. Kmean: 0.55 D
Post. elev. of thinnest point: -2 µm
Thinnest point: 497 µm
Averaged pachymetry towards thinnest point [µm]
Relative pachymetry towards thinnest point [%]
Pupil diameter
Pupil center

CASE #90

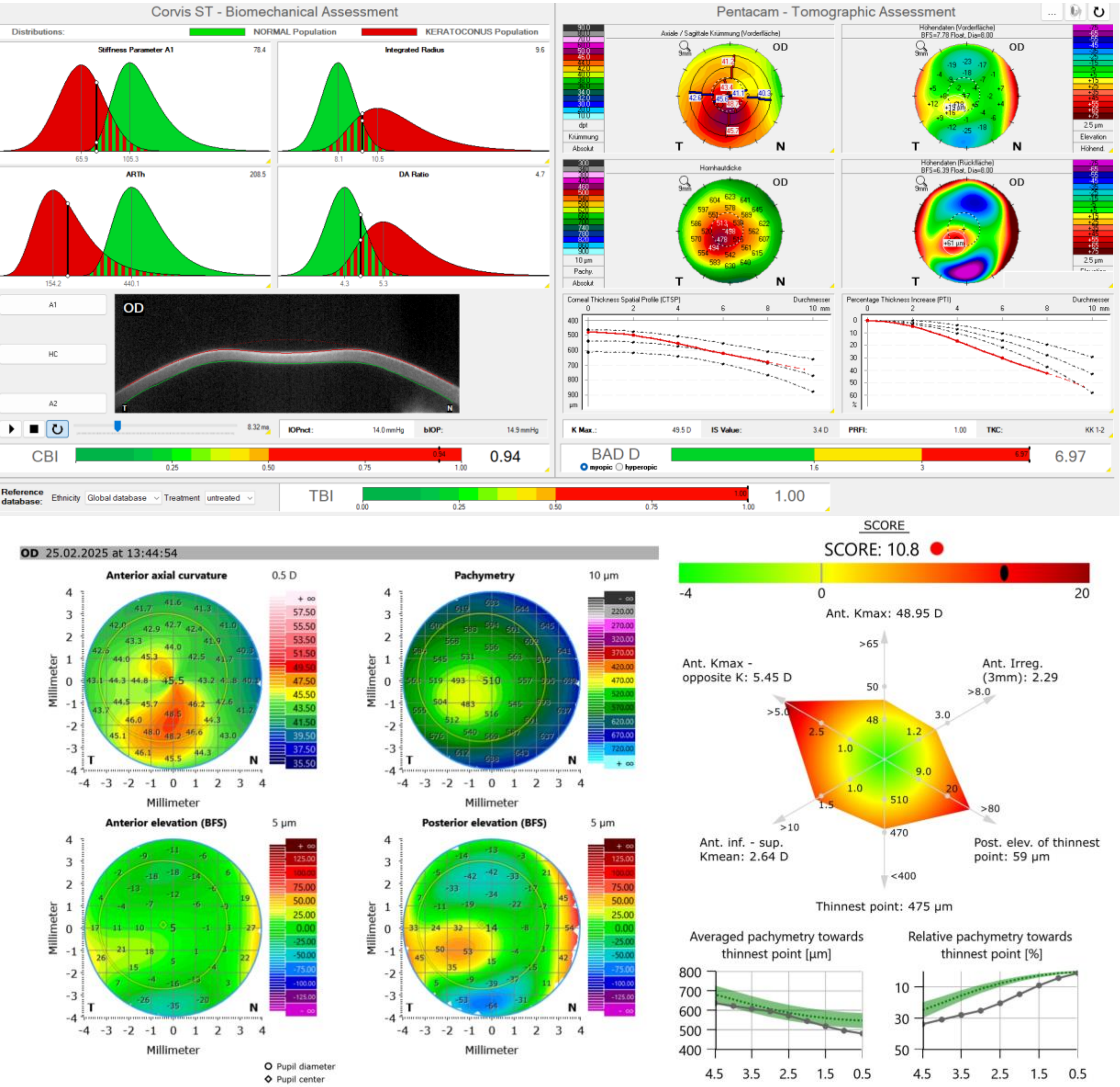

Corvis ST - Biomechanical Assessment
Pentacam - Tomographic Assessment
NORMAL Population
KERATOCONUS Population
OD
CBI 0.94
BAD D 6.97
TBI 1.00
SCORE
OD 25.02.2025 at 13:44:54
Anterior axial curvature
Pachymetry
Anterior elevation (BFS)
Posterior elevation (BFS)
Millimeter
SCORE: 10.8
Ant. Kmax: 48.95 D
Ant. Kmax - opposite K: 5.45 D
Ant. Irreg. (3mm): 2.29
Ant. inf. - sup. Kmean: 2.64 D
Post. elev. of thinnest point: 59 µm
Thinnest point: 475 µm
Averaged pachymetry towards thinnest point [µm]
Relative pachymetry towards thinnest point [%]
Pupil diameter
Pupil center

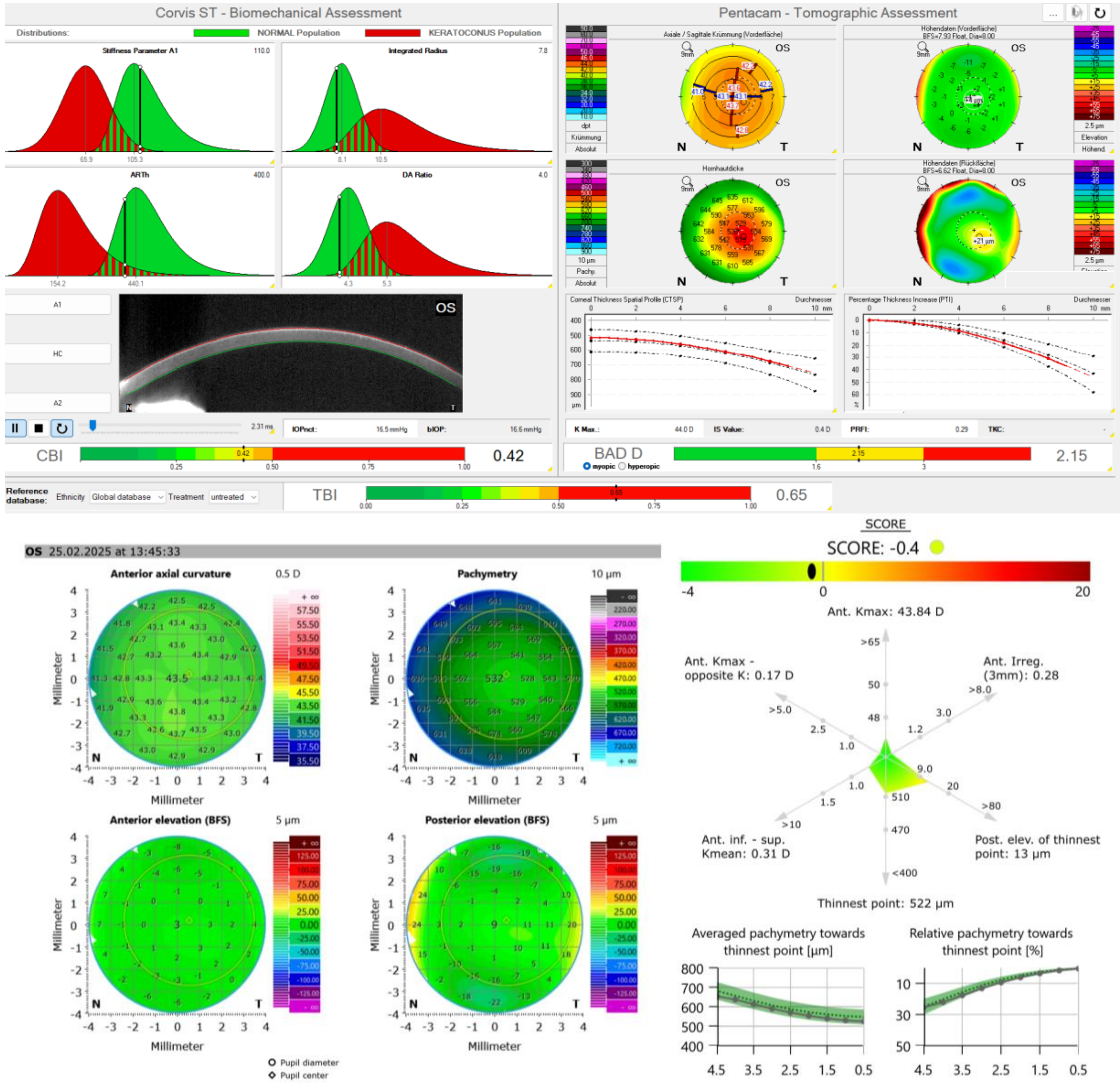

Corvis ST - Biomechanical Assessment
Pentacam - Tomographic Assessment
NORMAL Population
KERATOCONUS Population
OS
CBI 0.42
BAD D 2.15
TBI 0.65
SCORE
OS 25.02.2025 at 13:45:33
Anterior axial curvature
Pachymetry
Anterior elevation (BFS)
Posterior elevation (BFS)
Millimeter
SCORE: -0.4
Ant. Kmax: 43.84 D
Ant. Kmax - opposite K: 0.17 D
Ant. Irreg. (3mm): 0.28
Ant. inf. - sup. Kmean: 0.31 D
Post. elev. of thinnest point: 13 µm
Thinnest point: 522 µm
Averaged pachymetry towards thinnest point [µm]
Relative pachymetry towards thinnest point [%]
Pupil diameter
Pupil center

CASE #91

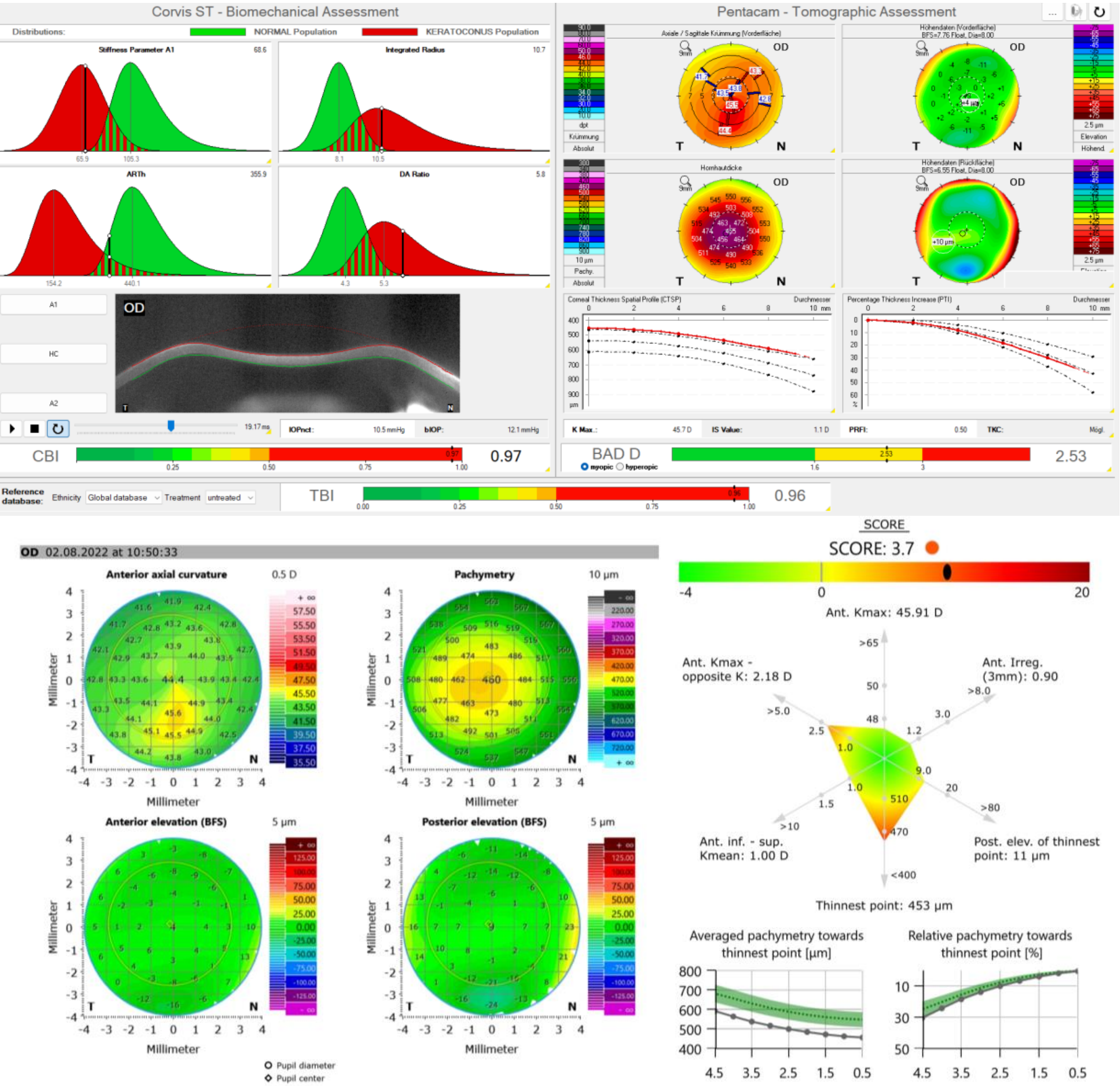
Corvis ST - Biomechanical Assessment
Pentacam - Tomographic Assessment
NORMAL Population
KERATOCONUS Population
Stiffness Parameter A1
Integrated Radius
ARTh
DA Ratio
OD
CBI
0.97
BAD D
2.53
TBI
0.96
OD 02.08.2022 at 10:50:33
Anterior axial curvature
Pachymetry
Anterior elevation (BFS)
Posterior elevation (BFS)
Millimeter
SCORE
SCORE: 3.7
Ant. Kmax: 45.91 D
Ant. Kmax - opposite K: 2.18 D
Ant. Irreg. (3mm): 0.90
Ant. inf. - sup. Kmean: 1.00 D
Post. elev. of thinnest point: 11 µm
Thinnest point: 453 µm
Averaged pachymetry towards thinnest point [µm]
Relative pachymetry towards thinnest point [%]
Pupil diameter
Pupil center

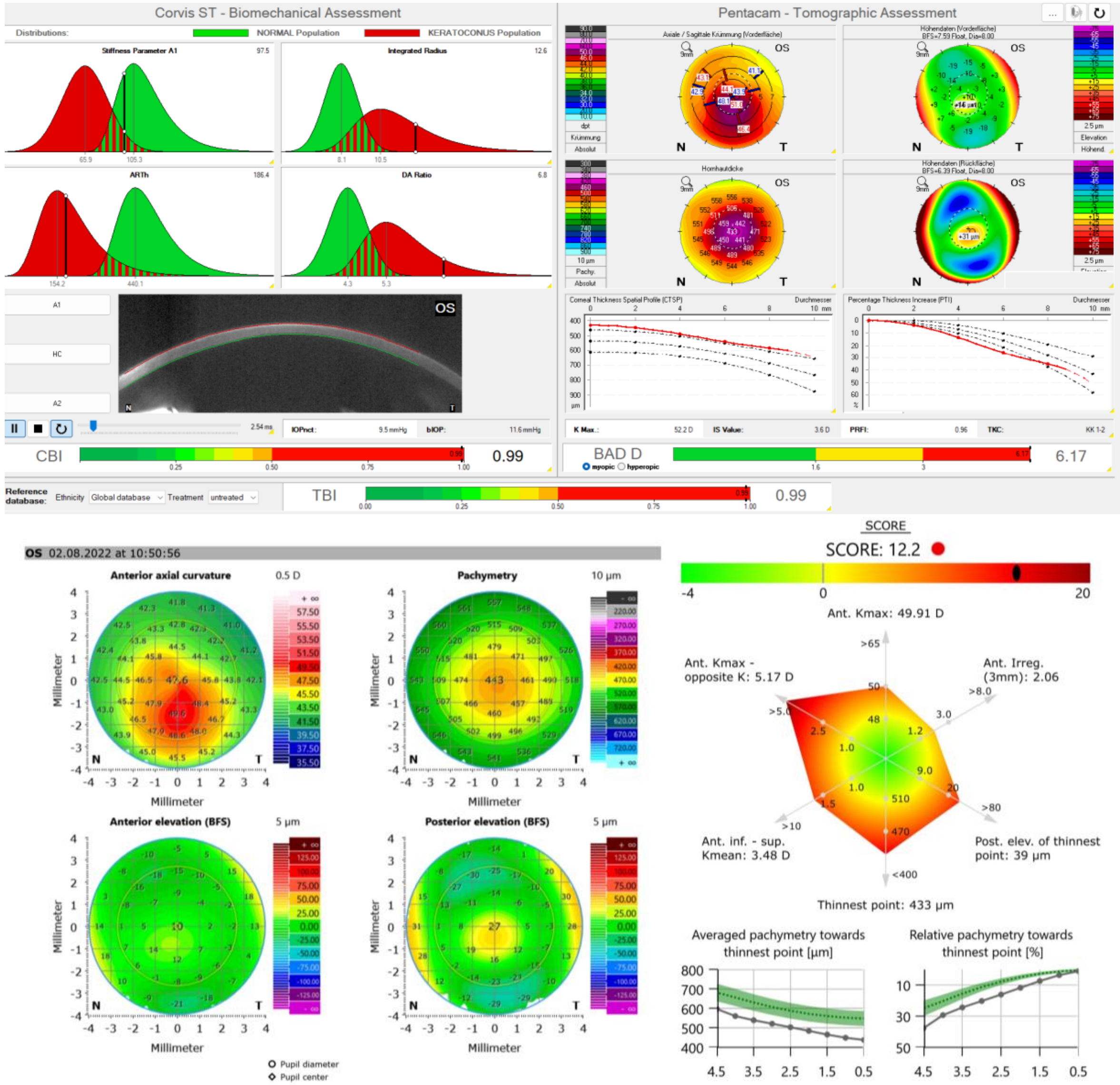
Corvis ST - Biomechanical Assessment
Pentacam - Tomographic Assessment
NORMAL Population
KERATOCONUS Population
Stiffness Parameter A1
Integrated Radius
ARTh
DA Ratio
OS
CBI
0.99
BAD D
6.17
TBI
0.99
OS 02.08.2022 at 10:50:56
Anterior axial curvature
Pachymetry
Anterior elevation (BFS)
Posterior elevation (BFS)
Millimeter
SCORE
SCORE: 12.2
Ant. Kmax: 49.91 D
Ant. Kmax - opposite K: 5.17 D
Ant. Irreg. (3mm): 2.06
Ant. inf. - sup. Kmean: 3.48 D
Post. elev. of thinnest point: 39 µm
Thinnest point: 433 µm
Averaged pachymetry towards thinnest point [µm]
Relative pachymetry towards thinnest point [%]
Pupil diameter
Pupil center
