# Supplementary material for: Comparative Diagnostic Efficacy of Swept-Source OCT and Scheimpflug Imaging in Clinically Unaffected Eyes of Very Asymmetric Ectasia
Source: Ophthalmol Sci. 2026 Jun 15;6(8):101285. doi: 10.1016/j.xops.2026.101285 (PMC13383214; doi:10.1016/j.xops.2026.101285)
Supplement: Supplement 2 [file mmc2.pdf]

Supplement 2 Data obtained from of the SS-OCT device presented in mean, standard deviation (SD), minimum, and maximum.

	group	Mean	SD	Minimum	Maximum
Inferior – superior K mean (D)	Healthy	-0.0568	0.481	-1.42	1.31
	bilateral KC	5.881	3.1635	-0.11	15.09
	VAE-NES	0.398	0.3975	-0.65	1.23
	VAE-E	5.909	3.0486	0.07	14.78
Anterior irregularity [3 mm] (D)	Healthy	0.93	0.5913	0.17	2.93
	bilateral KC	3.542	1.5795	0.13	9.16
	VAE-NES	0.746	0.2993	0.23	1.68
	VAE-E	3.796	1.5394	1.67	8.51
Kmax (D)	Healthy	44.852	1.5067	40.87	49.15
	bilateral KC	53.56	5.9881	41.77	88.88
	VAE-NES	44.532	1.3126	40.82	47.57
	VAE-E	53.429	4.2108	45.33	64.55
Kmax − opposite K (D)	Healthy	0.668	0.5874	0	2.75
	bilateral KC	8.605	5.4032	0.01	25.73
	VAE-NES	0.921	0.8997	0	6.43
	VAE-E	8.155	5.4043	0.02	22.19
simK flat (D)	Healthy	42.726	1.3562	39.77	45.73
	bilateral KC	45.307	3.7483	37.24	68.92
	VAE-NES	42.786	1.2672	39.33	45.41
	VAE-E	45.062	2.8652	38.7	53.53
simK steep (D)	Healthy	44.469	1.4795	40.63	47.24
	bilateral KC	48.726	4.3472	40.3	75.11
	VAE-NES	43.968	1.3103	40.49	46.64
	VAE-E	48.652	3.3752	43.02	57.33
CCT (μm)	Healthy	549.868	30.5518	480	683
	bilateral KC	484.958	49.254	354	932
	VAE-NES	526.945	31.1535	467	627
	VAE-E	493	37.0208	404	571
(PIT1) Relative pachyme try towards thinnest point 0.5 mm ring	Healthy	0.311	0.05321	0.2	0.5
	bilateral KC	1.073	0.5178	0.2	4.7
	VAE-NES	0.347	0.1058	0.2	0.8
	VAE-E	1.108	0.452	0.5	2.7
(PIT2) Relative pachyme try towards thinnest point 1.0 mm ring	Healthy	1.21	0.1967	0.6	1.8
	bilateral KC	4.066	1.9428	0.9	17.2
	VAE-NES	1.34	0.3696	0.7	2.9
	VAE-E	4.167	1.6821	1.8	10

(PIT3) Relative pachymetry towards thinnest point 1.5 mm ring	Healthy	2.727	0.4178	1.6	4
	bilateral KC	8.488	3.8727	2	34.3
	VAE-NES	2.986	0.7568	1.6	6.2
	VAE-E	8.701	3.3688	4	20.1
(PIT4) Relative pachymetry towards thinnest point 2.0 mm ring	Healthy	4.888	0.7305	3.2	7
	bilateral KC	13.672	5.8763	3.7	52.2
	VAE-NES	5.251	1.2037	3	10.2
	VAE-E	14.024	5.1649	6.8	30.5
(PIT5) Relative pachymetry towards thinnest point 2.5 mm ring	Healthy	7.725	1.1115	5.5	10.9
	bilateral KC	18.96	7.6128	5.9	67.5
	VAE-NES	8.132	1.6439	4.9	14.3
	VAE-E	19.408	6.6833	10	39.1
(PIT6) Relative pachymetry towards thinnest point 3.0 mm ring	Healthy	11.243	1.5721	7.9	15.7
	bilateral KC	23.556	8.8836	8.4	78.1
	VAE-NES	11.582	2.0927	7.5	19.1
	VAE-E	23.967	7.6672	13.4	49.3
(PIT7) Relative pachymetry towards thinnest point 3.5 mm ring	Healthy	15.449	2.1031	10.8	22
	bilateral KC	26.838	9.7205	9.5	83.9
	VAE-NES	15.449	2.6124	9.7	25.6
	VAE-E	26.979	8.27	14.4	54.3
(PIT8) Relative pachymetry towards thinnest point 4.0 mm ring	Healthy	20.182	2.7805	13.9	30.2
	bilateral KC	29.728	10.0165	11.3	88.1
	VAE-NES	19.729	3.4115	11.8	32.8
	VAE-E	29.717	8.5636	16.7	61.1
(PIT9) Relative pachymetry towards thinnest point 4.5 mm ring	Healthy	24.675	3.4662	16.8	36.8
	bilateral KC	33.446	10.562	12.4	95.4
	VAE-NES	24.345	4.1556	14.1	39.4
	VAE-E	33.439	9.0665	20.7	68.2
Posterior elevation of thinnest point (μm)	Healthy	5.711	3.8592	-8	21
	bilateral KC	61.094	32.1638	-11	240
	VAE-NES	7.912	6.1439	-5	30
	VAE-E	62.613	27.3806	19	144
TCT (μm)	Healthy	546.566	30.4342	477	681
	bilateral KC	466.844	43.6281	333	701
	VAE-NES	522.176	31.0231	464	614
	VAE-E	474.04	37.5274	391	542
SCORE	Healthy	-1.5	0.8115	-3.4	0.8
	bilateral KC	17.769	9.6441	1.149	51.1
	VAE-NES	0.322	1.2474	-2.309	3.414

	VAE-E	17.511	9.2144	3.103	46.746

CCT, central corneal thickness; E, ectasia; K, keratometry; Kmax, maximum keratometry; KC, bilateral keratoconus; PostKmax Y, vertical position of maximum posterior keratometry; NES, non-ectatic signs; Pr/Ar, the ratio of posterior to anterior corneal radius; PTI(X), percentage thickness increase at X mm from the thinnest pachymetry; SCORE, Screening Corneal Objective Risk of Ectasia; SimK, simulated keratometry; TCT, thinnest corneal thickness; VAE, very asymmetric ectasia.
