# Supplementary material for: Comparative Diagnostic Efficacy of Swept-Source OCT and Scheimpflug Imaging in Clinically Unaffected Eyes of Very Asymmetric Ectasia
Source: Ophthalmol Sci. 2026 Jun 15;6(8):101285. doi: 10.1016/j.xops.2026.101285 (PMC13383214; doi:10.1016/j.xops.2026.101285)
Supplement: Supplement 3 [file mmc3.pdf]

Supplement 3 Data obtained from Scheimpflug imaging devices presented in mean, standard deviation (SD), minimum, and maximum.

	group	Mean	SD	Minimum	Maximum
I-S value (D)	Healthy	-0.07	0.61	-1.83	1.35
	bilateral KC	6.33	3.33	-0.63	16.97
	VAE-NES	0.44	0.52	-0.89	1.33
	VAE-E	6.08	3.09	-0.13	15.36
KISA%	Healthy	6	8	0	53
	bilateral KC	1745	4362	5	56127
	VAE-NES	12	13	0	54
	VAE-E	1466	2630	7	12097
RPI Avg.	Healthy	0.99	0.10	0.79	1.32
	bilateral KC	1.96	0.71	0.00	7.31
	VAE-NES	1.07	0.19	0.75	2.12
	VAE-E	1.94	0.58	0.98	4.55
ART Max.	Healthy	453	65	308	675
	bilateral KC	178	69	0	598
	VAE-NES	385	83	186	635
	VAE-E	178	59	57	469
BAD D	Healthy	1.00	0.54	-0.33	2.56
	bilateral KC	8.10	3.97	0.33	31.12
	VAE-NES	1.48	0.76	0.07	3.86
	VAE-E	7.81	3.20	3.02	17.74
PRFI	Healthy	0.09	0.10	0.00	0.56
	bilateral KC	0.96	0.08	0.28	1.00
	VAE-NES	0.27	0.22	0.01	0.74
	VAE-E	0.98	0.03	0.87	1.00
TBI	Healthy	0.16	0.19	0.00	0.78
	bilateral KC	0.99	0.06	0.00	1.00
	VAE-NES	0.57	0.32	0.00	1.00
	VAE-E	1.00	0.00	0.99	1.00
CBI	Healthy	0.22	0.18	0.00	0.77
	bilateral KC	0.91	0.20	0.02	1.00
	VAE-NES	0.50	0.29	0.00	0.98
	VAE-E	0.94	0.12	0.34	1.00

ART max, Ambrosio relational thickness; BAD D, Belin/Ambrosio total deviation value; CBI, Corvis biomechanical index; E, ectasia; KISA%, keratoconus percentage index score; KC, bilateral keratoconus; I-S, the inferior-superior asymmetry value; MCT, minimal corneal thickness; NES, non-ectatic signs; PRFI, Pentacam random forest index; RPI Avg, averaged pachymetric progression. TBI, tomographical and biomechanical index (version 2); VAE, very asymmetric ectasia.
