# Supplementary material for: Comparative Diagnostic Efficacy of Swept-Source OCT and Scheimpflug Imaging in Clinically Unaffected Eyes of Very Asymmetric Ectasia
Source: Ophthalmol Sci. 2026 Jun 15;6(8):101285. doi: 10.1016/j.xops.2026.101285 (PMC13383214; doi:10.1016/j.xops.2026.101285)
Supplement: Supplement 4 [file mmc4.pdf]

Supplement 4 Receiver operating characteristic (ROC) analysis of machine learning and artificial intelligence parameters from SS-OCT and Scheimpflug imaging systems

Parameter	Group	AUC	YI	Cut-off	Sn (%)	Sp (%)	+LR	-LR
Kmax − opposite K (D)	NE vs. all ectasia	0.892 (0.867 - 0.914)	0.7129	>2.47	71.73	99.56	163.54	0.28
	NE vs. VAE-NES	0.590 (0.534 - 0.645)	0.1852	>0.9	43.96	74.56	1.73	0.75
Inferior – superior K mean (D)	NE vs. all ectasia	0.952 (0.933 - 0.966)	0.8090	>0.73	83.97	96.93	27.35	0.17
	NE vs. VAE-NES	0.767 (0.717 - 0.813)	0.4139	>0.15	74.73	66.67	2.24	0.38
Anterior irregularity [3 mm] (D)	NE vs. all ectasia	0.866 (0.838 - 0.890)	0.6211	>1.84	70.89	91.23	8.08	0.32
	NE vs. VAE-NES	0.551 (0.495 - 0.607)	0.1818	≤1.17	92.31	25.88	1.25	0.33
	NE vs. all ectasia	0.921 (0.899 - 0.940)	0.7950	>16	80.38	99.12	91.63	0.20

Posterior elevation of thinnest point (µm)	NE vs. VAE-NES	0.612 (0.556 - 0.666)	0.1918	>7	47.25	71.93	1.68	0.73
PostKmax Y (mm)	NE vs. all ectasia	0.941 (0.921 - 0.958)	0.8810	≤0.34	94.70	93.39	14.33	0.057
	NE vs. VAE-NES	0.819 (0.772 - 0.860)	0.6765	≤0.67	77.78	89.87	7.68	0.25
PTI2	NE vs. all ectasia	0.916 (0.893 - 0.936)	0.7992	>1.7	80.80	99.12	92.11	0.19
	NE vs. VAE-NES	0.598 (0.542 to 0.652)	0.2071	>1.3	41.76	78.95	1.98	0.74
Pr/Ar	NE vs. all ectasia	0.674 (0.638 - 0.708)	0.3811	≤0.81	44.28	93.83	7.18	0.59
	NE vs. VAE-NES	0.654 (0.599 - 0.707)	0.2403	>0.84	52.22	71.81	1.85	0.67
I-S value (D)	NE vs. all ectasia	0.945 (0.925 - 0.961)	0.8049	>1.02	83.12	97.37	31.59	0.17

	NE vs. VAE-NES	0.732 (0.680 - 0.780)	0.3743	>0.22	68.13	69.30	2.22	0.46
KISA%	NE vs. all ectasia	0.925 (0.903 - 0.943)	0.7708	>26.233	80.59	96.49	22.97	0.20
	NE vs. VAE-NES	0.639 (0.584 - 0.692)	0.2951	>6.01	57.14	72.37	2.07	0.59
RPI Avg.	NE vs. all ectasia	0.915 (0.892 - 0.935)	0.7801	>1.2	79.32	98.68	60.29	0.21
	NE vs. VAE-NES	0.631 (0.576 - 0.684)	0.2228	>1.02	58.24	64.04	1.62	0.65
ART Max.	NE vs. all ectasia	0.947 (0.928 - 0.962)	0.8218	≤343	84.81	97.37	32.23	0.16
	NE vs. VAE-NES	0.753 (0.702 - 0.799)	0.4227	≤424	76.92	65.35	2.22	0.35

ART max, Ambrosio relational thickness; AUC, area under curve; NE, normal eyes; +LR, positive likelihood-ratio; -LR, negative likelihood-ratio; NES, non-ectatic signs; KISA%, keratoconus percentage index score; I-S, the inferior-superior asymmetry value; PostKmax Y, vertical position of maximum posterior keratometry; Pr/Ar, the ratio of posterior to anterior corneal radius; PTI2, percentage thickness increase at 2 mm from the thinnest pachymetry; RPI Avg, averaged pachymetric progression; Sn, sensitivity; Sp, specificity; VAE, very asymmetric ectasia; YI, Youden-Index.
